# Base-catalyzed aryl halide isomerization enables the 4-selective substitution of 3-bromopyridines†

**DOI:** 10.1039/d0sc02689a

**Published:** 2020-09-09

**Authors:** Thomas R. Puleo, Jeffrey S. Bandar

**Affiliations:** Department of Chemistry, Colorado State University Fort Collins Colorado 80523 USA jeff.bandar@colostate.edu

## Abstract

The base-catalyzed isomerization of simple aryl halides is presented and utilized to achieve the 4-selective etherification, hydroxylation and amination of 3-bromopyridines. Mechanistic studies support isomerization of 3-bromopyridines to 4-bromopyridines proceeds *via* pyridyne intermediates and that 4-substitution selectivity is driven by a facile aromatic substitution reaction. Useful features of a tandem aryl halide isomerization/selective interception approach to aromatic functionalization are demonstrated. Example benefits include the use of readily available and stable 3-bromopyridines in place of less available and stable 4-halogenated congeners and the ability to converge mixtures of 3- and 5-bromopyridines to a single 4-substituted product.

## Abstract 2

The base-catalyzed isomerization of aryl halides is described and applied to the tandem isomerization/4-substitution of 3-bromopyridines as a strategy for achieving unconventional selectivity in nucleophilic aromatic substitution reactions.

## Graphical abstract

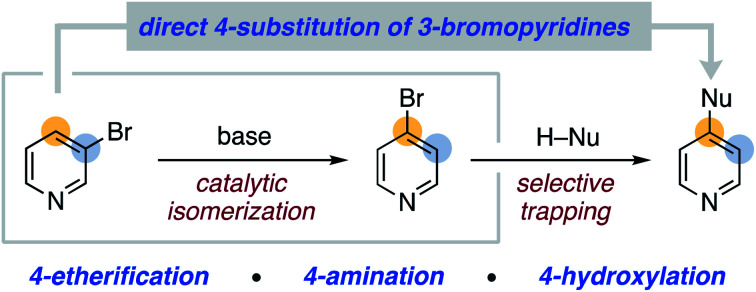

## Introduction

**Scheme 1:**
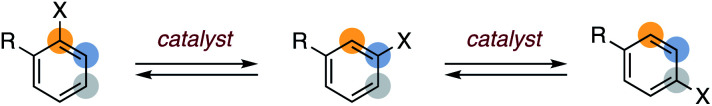
Concept of catalytic aryl halide isomerization.

The synthetic value of aryl halides derives from their thoroughly studied reactivity that allows reliable and predictable access to functionalized aromatic compounds.^1^ The utility of these widely available substrates can be significantly increased as new reactivity modes are discovered and applied. In this regard, catalytic aryl halide isomerization drew our attention as a relatively underdeveloped yet potentially useful process (Scheme 1).^2^

The rearrangement of halogenated arenes under basic conditions has been extensively studied in the context of “halogen dance” chemistry.^3^ Early studies by Bunnett on the base-catalyzed isomerization of 1,2,4-tribromobenzene into 1,3,5-tribromobenzene revealed rearrangement occurs *via* intermolecular halogen transfer, resulting in regioisomeric mixtures and disproportionated side products.^4^ This catalytic rearrangement requires an acidic arene that can generate electrophilic halogen transfer intermediates (*e.g.* tetrabromobenzenes); thus, isomerization is observed for tribromobenzenes but not for simple aryl halides.^5^ This insight guided decades of development of modern “halogen dance” methodology, wherein stoichiometric and irreversible metalation of haloarenes can lead to rearrangement through intermolecular metal–halogen transposition.^6^ In addition to typically requiring stoichiometric lithium bases under cryogenic conditions, a synthetically useful dance requires a thermodynamic gradient in order to drive a selective rearrangement.^3^ In this regard, important achievements have been made in identifying specific classes of metalated haloarenes that rearrange as a strategy for electrophilic functionalization.^7^

**Fig. 1:**
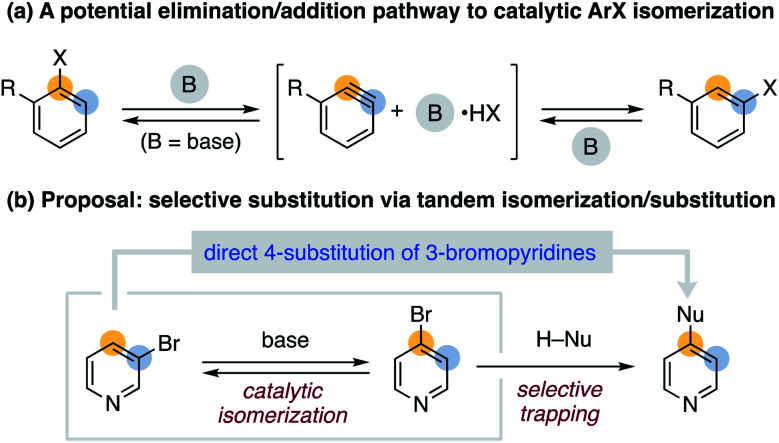
A general approach to aryl halide isomerization and its application to a new selective substitution reaction.

We sought to identify more mild and general conditions for aryl halide isomerization as an entry to developing new arene functionalization methods. Inspired by sporadic reports of rearranged aryl halide side products^8^ in reactions involving aryne^9,10^ intermediates, we hypothesized that non-nucleophilic bases could enable reversible HX elimination/addition as an additional isomerization pathway (Fig. 1a). We further proposed that pairing isomerization with a tandem substitution reaction could provide a driving force for nontraditional selectivity in aromatic substitution reactions (Fig. 1b).^11^ We herein describe initial studies on a general approach to catalytic aryl halide isomerization and demonstrate its utility as a new route to 4-functionalized pyridines.^12^

## Results and discussion

**Scheme 2:**
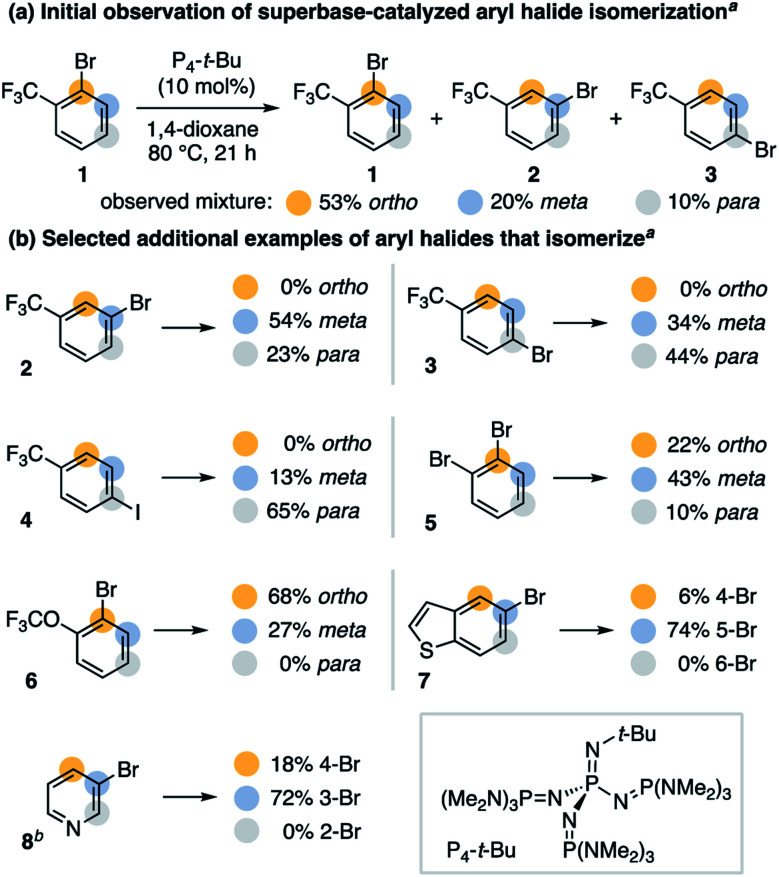
P_4_-*t*-Bu-catalyzed aryl halide isomerization. ^*a*^ Yields determined by ^1^H NMR spectroscopy; the mass balance is less than 100% with no observed haloarene side products; conditions for (b) are as shown in (a). ^*b*^ Reaction performed in cyclohexane for 14 h.

We speculated bases that reversibly deprotonate aryl C–H bonds may create conditions capable of isomerizing aryl halides.^13^ This led us to investigate the non-nucleophilic organic superbase P_4_-*t*-Bu (p*K*_BH^+^_ 30.2 in DMSO) as a potential isomerization catalyst.^14^ In 1,4-dioxane, we discovered P_4_-*t*-Bu catalyzes the isomerization of 2-bromobenzotrifluoride (**1**) into all possible regioisomers (Scheme 2a). Under these conditions, 3-bromobenzotrifluoride (**2**) and 4-bromobenzotrifluoride (**3**) interconvert but do not form 2-bromobenzotrifluoride (Scheme 2b). No protodehalogenated or polyhalogenated side products are observed, and we note isomerization occurs to a lesser extent for 4-iodobenzotrifluoride (**4**).^15^ A variety of other bromoarenes (**5**, **6** and **7**) also isomerize, including the formation of 4-bromopyridine from 3-bromopyridine (**8**).^16^ Although further studies on the scope of this process are ongoing, these observations suggest P_4_-*t*-Bu-catalyzed aryl halide isomerization is a general and reversible process.

**Scheme 3:**
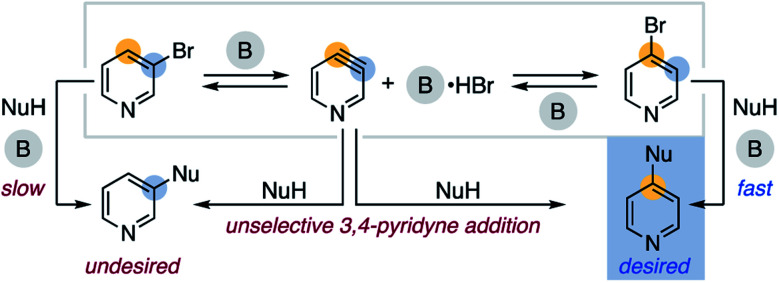
Proposed pathway for the 4-selective substitution of 3-bromopyridine (B = base, NuH = nucleophile).

As a broader objective, we questioned if aryl halide isomerization could be utilized to address current challenges in aromatic functionalization. As a halogen migrates around an arene, we reasoned that differing electronic properties of isomeric C–X bonds could provide a source to differentiate interconverting isomers and drive an overall selective transformation.^17^ A mechanistic outline for the application of this concept to the 4-substitution of 3-bromopyridines is shown in Scheme 3. This pathway exploits the inherent preference for 4-bromopyridines to undergo nucleophilic aromatic substitution (S_N_Ar) over 3-bromopyridines.^18^ However, a likely challenge is avoiding nucleophilic addition to the proposed 3,4-pyridyne intermediate, as this could decrease the desired reaction's yield and regioselectivity.^19^ Successful development of this protocol would offer an attractive route to 4-functionalized pyridines from 3-bromopyridines, which are more commercially available^20^ and stable^21^ than 4-halogenated congeners. This method would also complement other recently developed methods for 4-selective nucleophilic pyridine C–H functionalization, including McNally's powerful heterocyclic phosphonium salt approach.^22,23^

As the proposed process requires stoichiometric base, we first investigated the use of hydroxide bases as more practical reagents for promoting aryl halide isomerization. Hydroxide bases are known to generate and be compatible with aryne intermediates, and we identified that 3-bromopyridines isomerize in the presence of 18-crown-6-ligated KOH in *N*,*N*-dimethylacetamide (see ESI†).^24^ Using these conditions, we then employed two separate strategies for optimizing a 4-selective etherification reaction of 3-bromopyridine (Table 1). When 1 equivalent of 3-bromopyridine (**8**) reacts with 4 equivalents of alcohol **9**, a 2.4 : 1 ratio of 4:3-substituted product (**10** : **11**) is obtained in 54% overall yield (entry 1). This ratio is comparable to reported selectivities for nucleophilic additions to 3,4-pyridyne, which typically range from 1 : 1 to 3 : 1 for 4 : 3-addition selectivity.^25^ The 4-selectivity increases as higher ratios of pyridine : alcohol (**8** : **9**) are used, a result perhaps explainable by less alcohol intercepting a 3,4-pyridyne intermediate (entries 2–5). Based on this observation, we hypothesized that added bromide salts may enable more efficient isomerization and prevent undesired side reactions.^26^ When 50 mol% KBr is added to a reaction using a 1.5 : 1 pyridine : alcohol (**8** : **9**) ratio, the yield increases to 76% with >14 : 1 4-selectivity compared to 67% yield and 8.6 : 1 4-selectivity in the absence of bromide salt (entries 6–10).

**Table 1:** Optimization of 4-selective etherification of 3-bromopyridine^a^

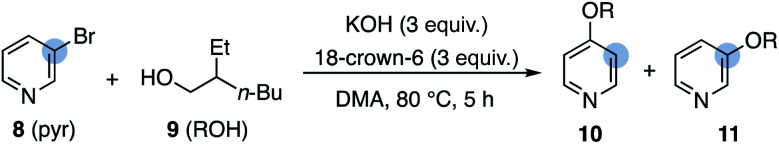							
Effect of pyridine : alcohol ratio				Effect of bromide salt additive^b^			
Entry	**8** : **9**	Yield	**10** : **11**	Entry	KBr	Yield	**10** : **11**
1	1 : 4	54%	2.4	6	0%	67%	8.6
2	1 : 2	65%	5.5	7	10%	69%	8.9
3	1 : 1	64%	8.1	8	20%	73%	11.1
4	2 : 1	95%	12.6	9	50%	76%	14.2
5	4 : 1	90%	11.9	10	100%	77%	14.2

a Yields and selectivities determined by ^1^H NMR spectroscopy of the crude reaction mixtures; yields represent total amount of both isomeric products **10** and **11**.

b 2.0 equiv. of KOH used with a 1 : 1 ratio of **8** : **9**.

**Scheme 4:**
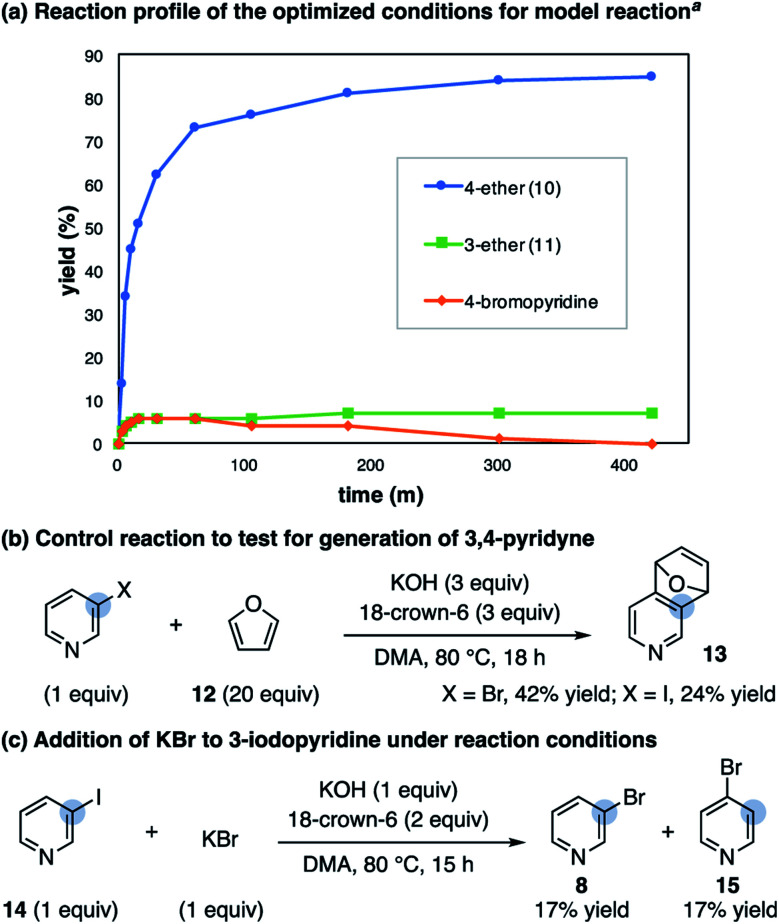
Mechanistic studies on the 4-selective etherification of 3-bromopyridine. ^*a*^ Conditions as shown in Table 1 using 1.5 : 1 ratio of **8** : **9** with 50 mol% KBr additive; see ESI† for details.

A reaction profile with the optimized conditions shows the rapid formation of a low concentration of 4-bromopyridine (approximately 5%) that decreases as the reaction reaches completion (Scheme 4a). Subjection of the 3-substituted ether product (**11**) to the reaction conditions does not result in mass balance loss, indicating the high 4-selectivity is not a result of selective decomposition or product rearrangement.^27^ To test for the generation of 3,4-pyridyne under these conditions, when the alcohol is replaced with an excess of furan (**12**) the corresponding cycloadduct **13** forms in 42% yield (Scheme 4b).^28^ We also subjected 3-iodopyridine (**14**) to the reaction conditions in the absence of alcohol; in the presence of furan (**12**) cycloadduct **13** forms and in the presence of KBr a mixture of 3- and 4-bromopyridine form (**8** and **15**, Scheme 4c). The observed mixture of 3- and 4-bromopyridine supports the proposal of bromide addition to 3,4-pyridyne. Overall, these results are consistent with an isomerization pathway *via* 3,4-pyridyne and 4-substitution selectivity driven by a facile S_N_Ar reaction.

A substrate scope for the 4-selective etherification of 3-bromopyridines is provided in Table 2.^29^ A range of primary and secondary alcohols are first shown using 1.5 equiv. of simple bromopyridines. Sterically hindered alcohols (**16**) and those containing amino groups (**17**, **23**, and **24**), a terminal alkene (**18**) and a protected sugar (**22**) are suitable nucleophiles. Pyridines methylated in all positions react in high yield and selectivity (**19**, **20**, **21** and **24**), indicating that steric hindrance and acidic C–H bonds are tolerated on the arene. Pyridine biaryl substrates also selectively couple in the 4-position (**25** and **26**). Both 2-alkoxy (**23**) and 2-amino (**28**) substituents on the pyridine are tolerated, although we note the high selectivity observed for these substrates could originate from alcohol addition to a distorted 3,4-pyridyne intermediate.^19^ Pyridine substrates with more electron-withdrawing groups undergo direct 3-substitution under the current reaction conditions (*e.g.* 3-bromo-2-(trifluoromethyl)pyridine).^30^

**Table 2:** Scope of the 4-etherification of 3-bromopyridines^a^

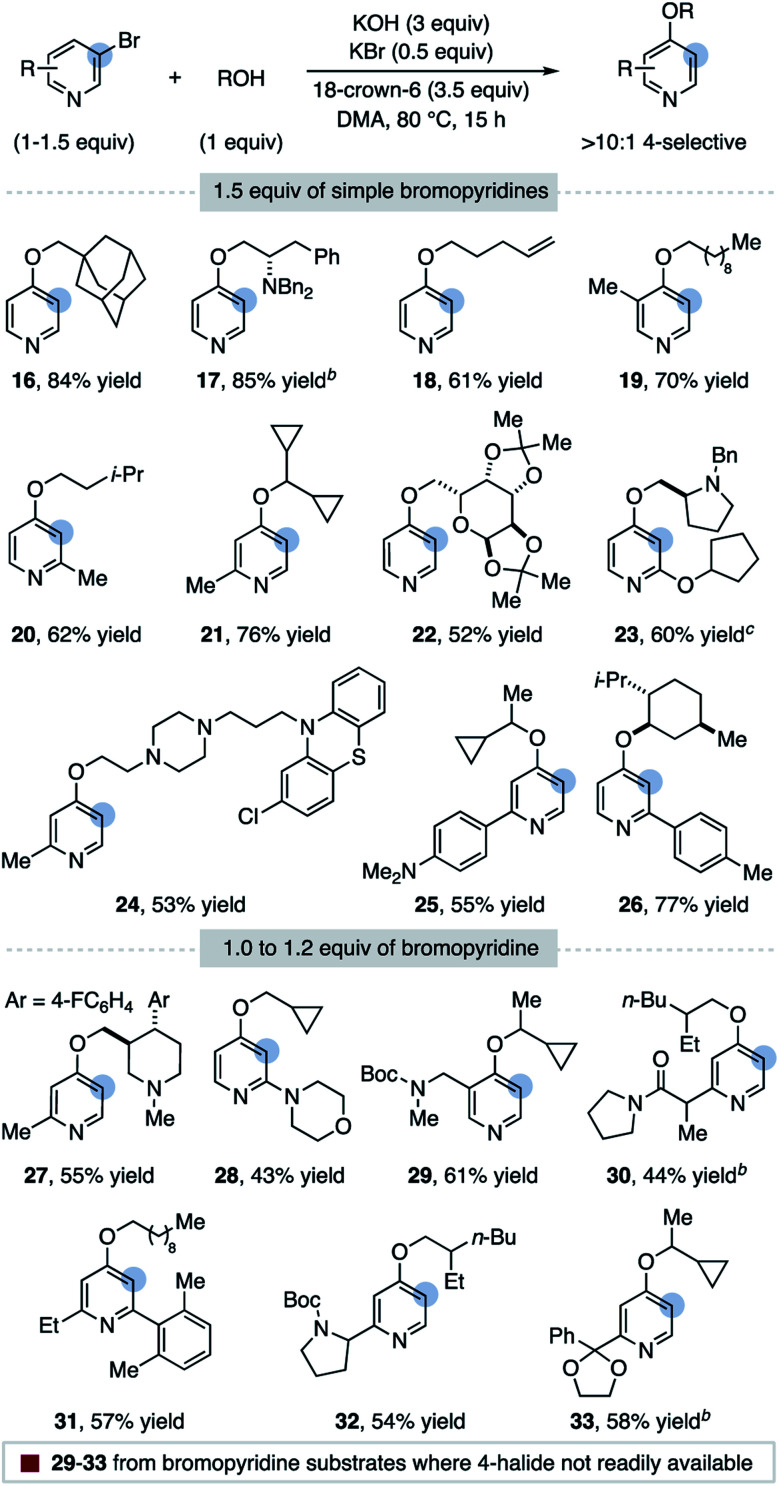

a Yields are of purified 4-ether product; regioselectivities determined by ^1^H NMR spectroscopy of the crude reaction mixture.

b Selectivity > 6 : 1; see ESI for details.

c P_4_-*t*-Bu (1.3 equiv.) used as base in place of KOH.

An advantage of this functionalization strategy is demonstrated with substrates **29–33** in Table 2, where the bromopyridine substrate is obtained from commercial pyridines while a 4-halogenated isomer is either not available or significantly more expensive (see ESI† for a discussion). Using 1–1.2 equiv. of these bromopyridines, 4-substituted products featuring a carbamate (**29**), an acidic amide (**30**), a 2,6-disubstituted pyridine (**31**), a nicotine isomer (**32**) and a ketal (**33**) can be rapidly accessed.

**Scheme 5:**
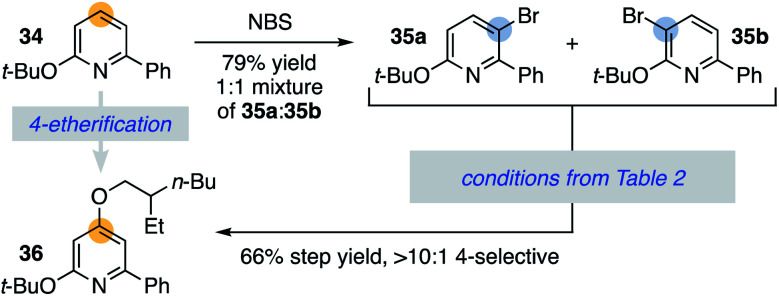
4-selective etherification of a 2,6-disubstituted pyridine.

This strategy can also utilize an arene's innate halogenation position as an entry to functionalizing more difficult to access C–H bonds. This is demonstrated in Scheme 5, where the 2,6-disubstituted pyridine **34** undergoes facile but unselective bromination in the 3- and 5-positions (**35a** and **35b**). Application of conditions from Table 2 provides access to the 4-ether **36** through convergence of the regioisomeric bromopyridine mixture, highlighting an additional benefit of this methodology.^31^

**Scheme 6:**
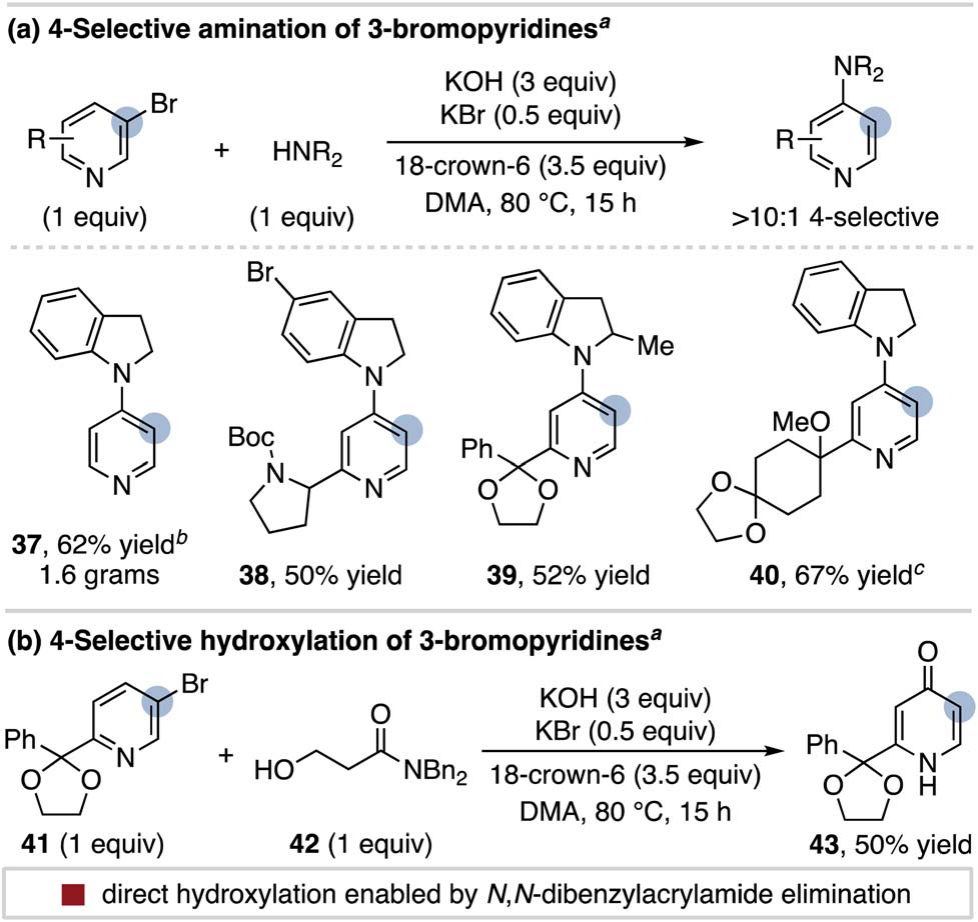
The 4-substitution of 3-bromopyridines with additional nucleophiles. ^*a*^ Isolated yield of purified 4-substituted products; selectivities determined by ^1^H NMR spectroscopy of crude reaction mixtures; ^*b*^ 1.5 equiv. of 3-bromopyridine used; ^*c*^ selectivity 9 : 1.

We next examined if other nucleophiles can participate in the 4-selective substitution of 3-bromopyridines. We found that indolines are effective coupling partners and this route provides straightforward access to 4-aminopyridines from readily available 3-bromopyridines (**37–40**, Scheme 6a). The 4-amination of 3-bromopyridine with indoline proceeds on gram scale with excellent selectivity (**37**). A single isomer of the 5-bromoindoline product **38** is obtained, demonstrating the chemoselectivity of aryl halide isomerization.

It is interesting to note that 4-hydroxypyridine side products are not typically observed for the reactions in Table 2 even though KOH is used as a base.^32^ To develop a 4-hydroxylation protocol, we instead hypothesized tandem isomerization/substitution could be further sequenced with a base-promoted elimination step. This is demonstrated in Scheme 6b, where the use of β-hydroxyamide **42** as a nucleophile directly delivers the 4-hydroxylated product **43** in 50% yield with >10 : 1 selectivity. We speculate this reaction proceeds through the standard 4-substitution pathway followed by a facile base-promoted acrylamide elimination reaction.^33^

## Conclusions

This work demonstrates base-catalyzed aryl halide isomerization can be paired with S_N_Ar reactivity to achieve unconventional substitution selectivity. In contrast, established “halogen dance” methodology relies on the controlled rearrangement of specific classes of stoichiometrically metalated haloarenes prior to treatment with electrophiles.^3^ Thus, tandem isomerization/selective interception may be a complementary and general strategy for achieving nontraditional selectivities in aryl halide functionalization chemistry.

## Conflicts of interest

There are no conflicts to declare.

## Supplementary Material

SC-011-D0SC02689A-s001
