# Supplementary material for: Base-catalyzed aryl halide isomerization enables the 4-selective substitution of 3-bromopyridines
Source: Chem Sci. 2020 Sep 9;11(38):10517–22. doi: 10.1039/d0sc02689a (PMC8162412; doi:10.1039/d0sc02689a)
Supplement: SC-011-D0SC02689A-s001 [file SC-011-D0SC02689A-s001.pdf]

Electronic Supplementary Material (ESI) for Chemical Science.
This journal is © The Royal Society of Chemistry 2020

Supporting Information

Base-Catalyzed Aryl Halide Isomerization Enables the 4-Selective Substitution of 3-Bromopyridines

Thomas R. Puleo and Jeffrey S. Bandar

Department of Chemistry, Colorado State University
Fort Collins, Colorado 80523, United States
Email: jeff.bandar@colostate.edu

Table of Contents

I.	General Information	S2
II.	Base-Catalyzed Isomerization of Aryl Halides	S3
III.	Optimization of the 4-Selective Etherification of 3-Bromopyridine	S12
IV.	Description of Mechanistic Studies on 3-Bromopyridine Isomerization	S19
V.	General Procedures for the 4-Selective Substitution of 3/5-Bromopyridines	S31
VI.	Characterization Data of 4-Substituted Pyridines	S38
VII.	Convergence of 3- and 5-Bromopyridine Isomers	S46
VIII.	Preparative Scale 4-Selective Amination of 3-Bromopyridine	S49
IX.	Preparation of 3- or 5-Bromopyridine Starting Materials	S50
X.	References	S58
XI.	Copies of NMR Spectra	S59

I. General Information

General Reagent Information: All reactions were performed under a N_2 atmosphere unless otherwise noted. Anhydrous *N,N*-dimethylacetamide (DMAc) (Catalog # 375230010) was purchased from Sigma Aldrich in a Sure/Seal® bottle stored over 4 Å molecular sieves and used as received. Potassium hydroxide (KOH) was purchased from Millipore Sigma (Catalog # 8143530100) as an 85% purity powder and used as purchased. Potassium bromide (KBr) (Catalog # A16339) was purchased from Alfa Aesar in 99.8% purity. Prior to use, KBr was dried *in vacuo* at 100 °C for 24 h. 1,4,7,10,13,16-Hexaoxacyclooctadecane (18-crown-6) was purchased from Chem-Impex (Catalog # 03901) and used as purchased. Anhydrous 2-ethyl-1-hexanol was purchased from Sigma-Aldrich (Catalog # 538051) and stored over 4 Å molecular sieves. 3-Bromopyridine was purchased from Chem-Impex (Catalog # 27044) and used as purchased. KOH, KBr, and 18-crown-6 were all stored at room temperature (rt) inside a N_2 filled glovebox and used immediately if brought outside the glovebox. Tetrahydrofuran, dichloromethane, and toluene were deoxygenated and dried by passage over packed columns of neutral alumina and copper (II) oxide under positive pressure of N_2. NaH was purchased from Acros as a 60% dispersion in mineral oil and was stored in a desiccator with $CaSO_4$ as the desiccant. 1-*tert*-Butyl-4,4,4-tris(dimethylamino)-2,2-bis[tris(dimethylamino)-phosphoranylidenamino]-2λ^5,4λ^5-catenadi(phosphazene) (P_4-*t*-Bu) was purchased from Millipore Sigma as a 0.8 M solution in hexanes and was stored in a -30 °C freezer inside a N_2 filled glovebox. Before use, the P_4-*t*-Bu solution was allowed to warm to rt and homogenize if any solid was evident. All other solvents and reagents were purchased from Millipore Sigma, Combi-Blocks, TCI, Acros Organics, Matrix Scientific, Alfa-Aesar, or Synthonix and used as received unless otherwise noted. Flash Chromatography was performed on 40-63 µm silica gel (SiliaFlash® F60 from Silicycle). Preparative thin-layer chromatography (PTLC) was performed on silica gel 60Å F254 plates (20 x 20 cm, 1000 µm, SiliaPlate from Silicycle, #TLG-R10011B-341) and visualized with UV light (254 nm).

General Analytical Information: All reported compounds were characterized by ^{1}H, ^{13}C, and ^{19}F (if applicable) NMR spectroscopy, FTIR spectroscopy and mass spectrometry. Melting point analysis was conducted if the compound was solid. ^{1}H NMR, ^{13}C NMR, and ^{19}F NMR spectra were obtained on a Bruker Advanced NEO or Varian Inova 400 spectrometer. ^{1}H NMR data is reported as follows: chemical shift (δ ppm), multiplicity (s = singlet, br s = broad singlet, d = doublet, t = triplet, q = quartet, dd = doublet of doublets, td = triplet of doublets, m = multiplet), coupling constant (Hz), and integration. ^{13}C NMR data is reported as follows: chemical shift (δ ppm), multiplicity (if applicable, d = doublet), and coupling constant (Hz). All ^{1}H NMR signals are reported as chemical shifts (*δ* ppm) relative to residual $CHCl_3$, DMSO, or MeOH at 7.26 ppm, 2.50 ppm, or 3.34 ppm, respectively. ^{13}C NMR signals are reported as chemical shifts (*δ* ppm) relative to $CDCl_3$, CD_3OD, or DMSO-d_6 at 77.23 ppm, 49.86 ppm, or 39.52 ppm, respectively. ^{19}F NMR signals are reported as chemical shifts relative to added α,α,α-trifluorotoluene internal standard calibrated to -63.72 ppm. High resolution mass spectra (HRMS) were recorded on an Agilent 6210 TOF interfaced to a DART 100 source provided by Colorado State University Central Instrumentation Facility and recalibrated using *O*-(2-aminopropyl)-*O*'-(2-methoxyethyl)polypropylene glycol M_n ~600 (Jeffamine® M-600) standard. IR spectra were recorded using a Thermo Scientific Nicolet iS-50 FTIR Spectrometer and reported as frequency of absorption (cm^{-1}). Melting point analyses were conducted using a Mel-Temp capillary melting

point apparatus. The specific rotation of chiral molecules was measured using a Rudolph Research Analytical Autopol III polarimeter. Thin-layer chromatography analysis was performed on silica gel 60Å F_{254} plates (250 μm, SiliaPlate from Silicycle, #TLG-R10014B-323) and interpreted using UV light (254 nm).

Note on nomenclature: The names provided for the structures below were obtained from ChemDraw Professional 16.0.

II. Base-Catalyzed Isomerization of Aryl Halides

General Procedure: An oven-dried 4 mL vial (ThermoFisher, C4015-1) was charged with a magnetic stir bar. Inside a N_2 filled glovebox, the appropriate aryl bromide/iodide (0.1 mmol, 1.0 equiv), 1,4-dioxane (0.4 mL, 0.25 M), and P_4-*t*-Bu (13 μl, 0.1 equiv) were added to the vial in successive order. The vial was sealed with a PTFE-lined screw cap (ThermoFisher, C4015-1A), removed from the glovebox, and placed into a preheated 80 °C aluminum reaction block. The reaction solution was stirred at 80 °C for 21 h. Upon completion of the given reaction time, the reaction solution was cooled to rt. 1,3,5-Trimethoxybenzene (TMB) internal standard was measured into the solution and ^{1}H NMR spectroscopy (400 MHz, $CDCl_3$) of the crude solution was used to determine the percentage of each aryl halide isomer in solution. The identity of each isomer within the spectrum was determined by ^{1}H NMR analysis of an authentic sample of the isomer. The figures below provide an overlay of the ^{1}H NMR spectra of the authentic regioisomers on the top, and the ^{1}H NMR spectrum of the crude reaction solutions on the bottom. ***Note***: the signal at δ = 6.08 ppm corresponds to the 1,3,5-trimethoxybenzene internal standard.

a)

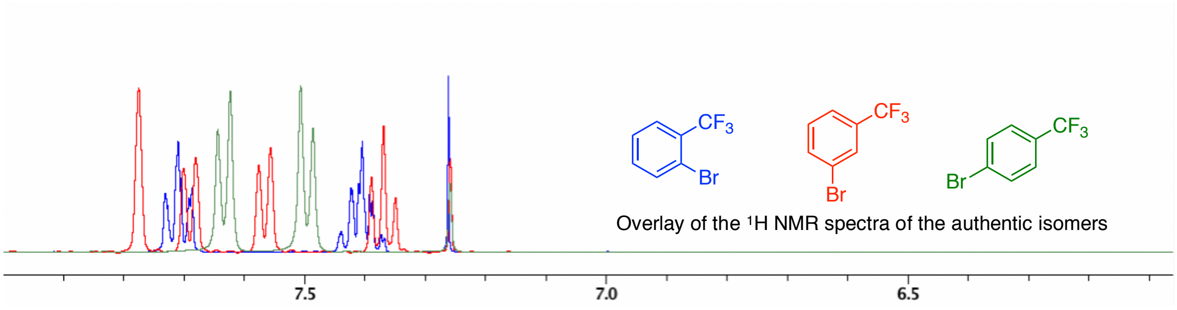

b)

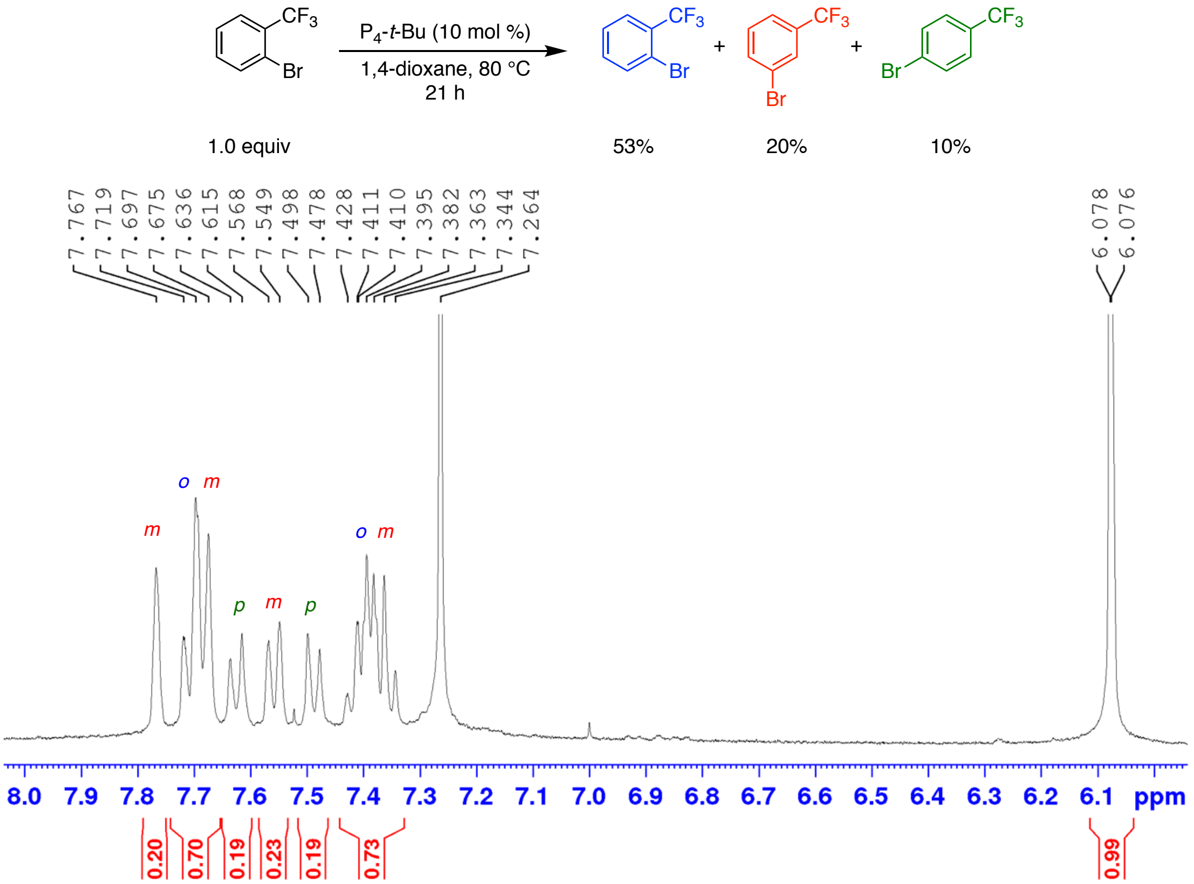

Figure S1: (**a**) Overlay of the ^{1}H NMR spectra of the authentic isomers of 1-bromo-2-(trifluoromethyl)benzene, 1-bromo-3-(trifluoromethyl)benzene and 1-bromo-4-(trifluoromethyl)benzene. (**b**) ^{1}H NMR spectrum of the crude reaction solution of 1-bromo-2-(trifluoromethyl)benzene base-catalyzed isomerization; the reaction was run according to the general procedure; 5.5 mg (0.033 mmol) of TMB was added to the reaction solution: 53% 1-bromo-2-(trifluoromethyl)benzene, 20% 1-bromo-3-(trifluoromethyl)benzene, 10% 1-bromo-4-(trifluoromethyl)benzene.

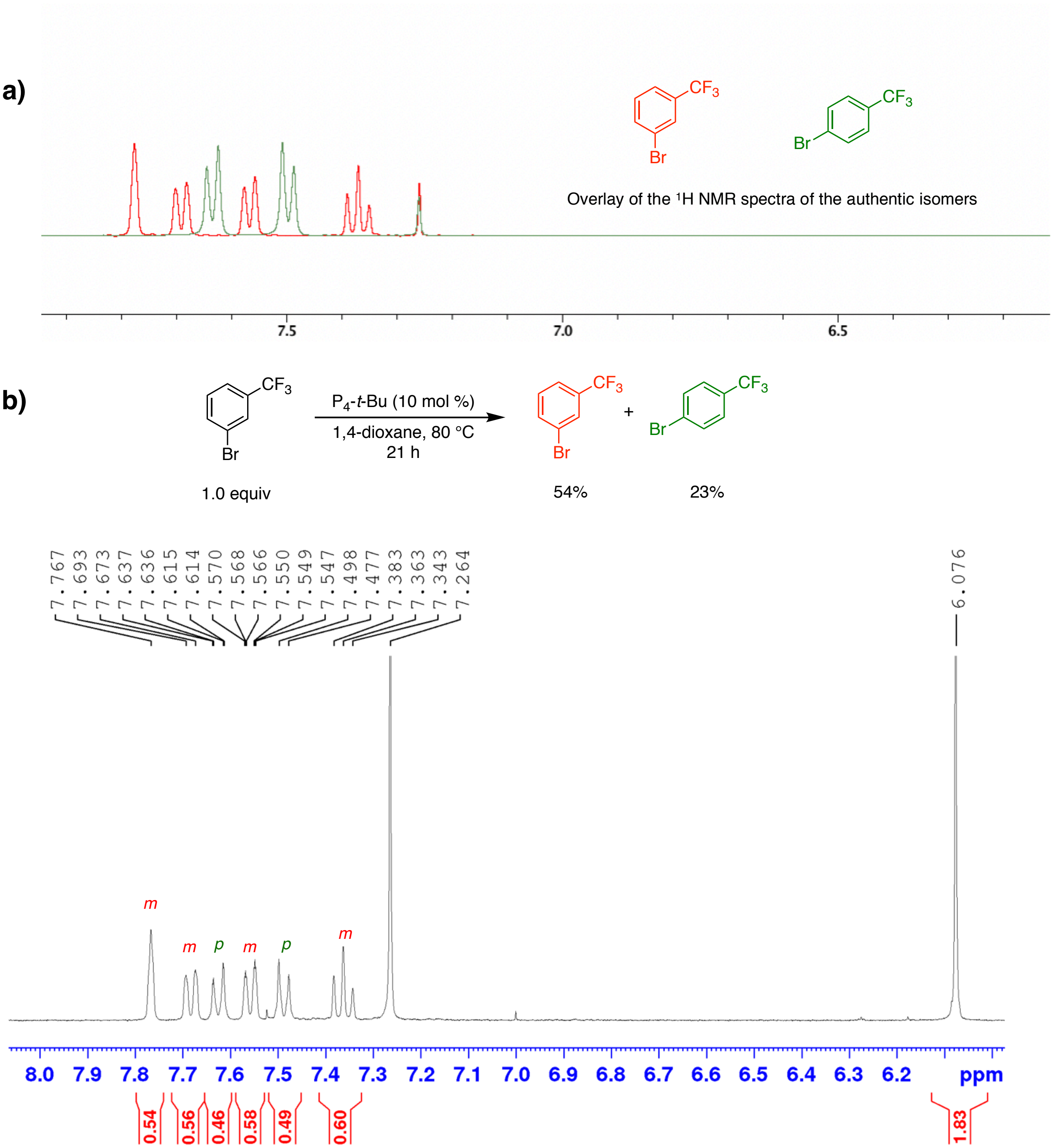

Figure S2: (**a**) Overlay of the ^{1}H NMR spectra of the authentic isomers of 1-bromo-3-(trifluoromethyl)benzene and 1-bromo-4-(trifluoromethyl)benzene. (**b**) ^{1}H NMR spectrum of the crude reaction solution of 1-bromo-3-(trifluoromethyl)benzene base-catalyzed isomerization; the reaction was run according to the general procedure; 10.3 mg (0.061 mmol) of TMB was added to the reaction solution: 54% 1-bromo-3-(trifluoromethyl)benzene and 23% 1-bromo-4-(trifluoromethyl)benzene.

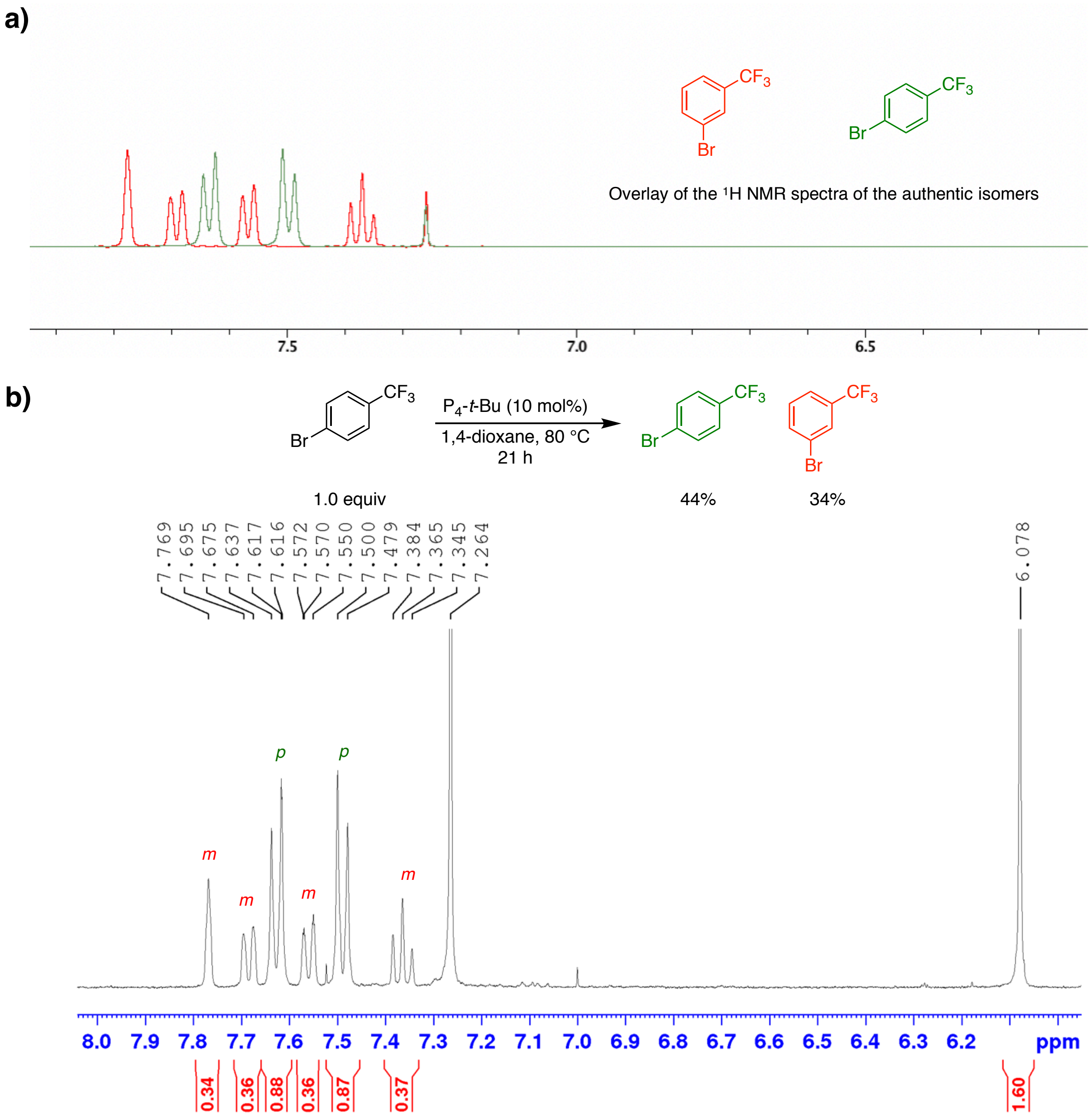

Figure S3: (**a**) Overlay of the ^{1}H NMR spectra of the authentic isomers of 1-bromo-4-(trifluoromethyl)benzene and 1-bromo-3-(trifluoromethyl)benzene. (**b**) ^{1}H NMR spectrum of the crude reaction solution of 1-bromo-4-(trifluoromethyl)benzene base-catalyzed isomerization; the reaction was run according to the general procedure; 8.9 mg (0.053 mmol) of TMB was added to the reaction solution: 44% 1-bromo-4-(trifluoromethyl)benzene and 34% 1-bromo-3-(trifluoromethyl)benzene.

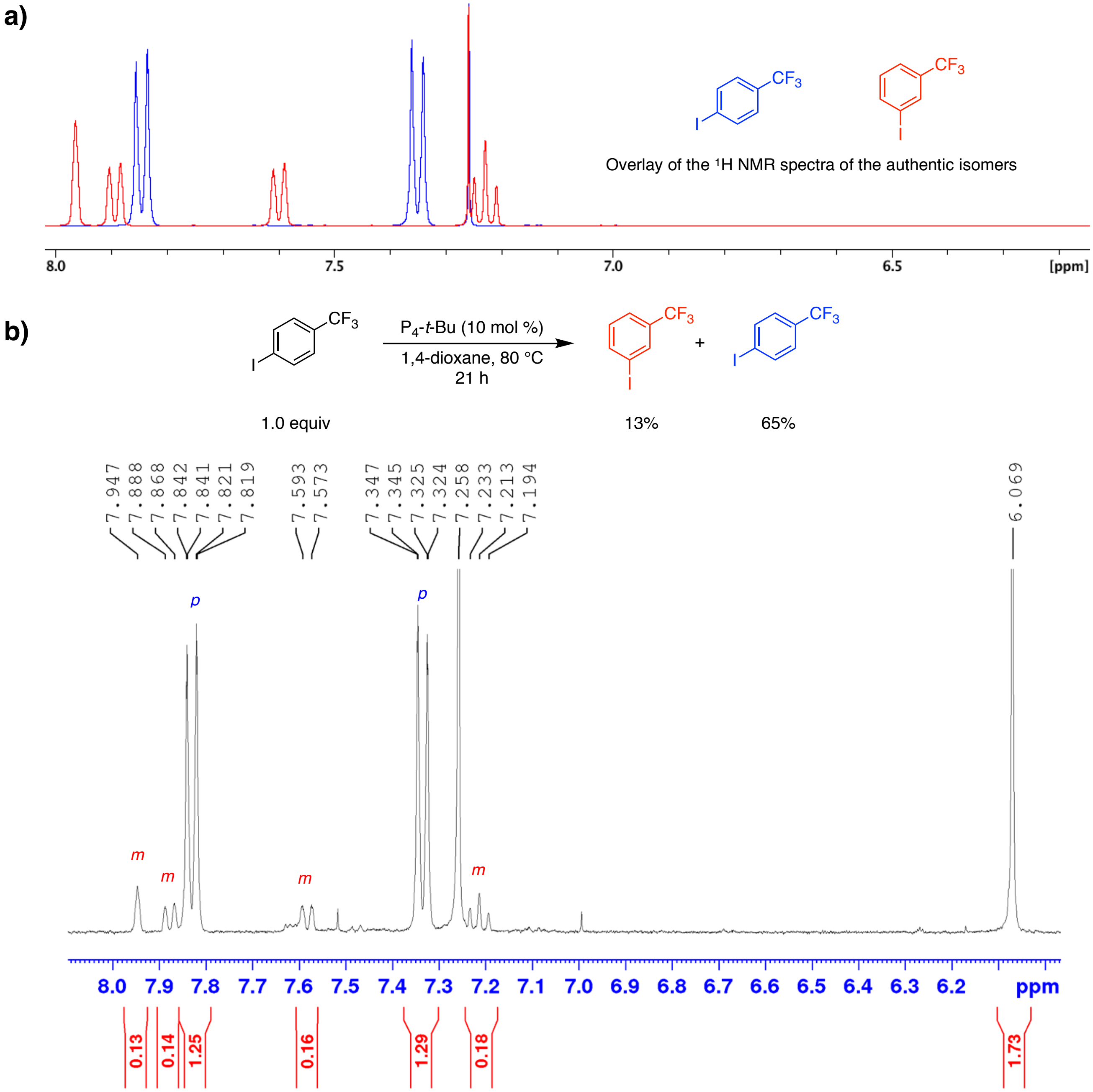

Figure S4: (**a**) Overlay of the ^{1}H NMR spectra of the authentic isomers of 1-iodo-4-(trifluoromethyl)benzene and 1-iodo-3-(trifluoromethyl)benzene. (**b**) ^{1}H NMR spectrum of the crude reaction solution of 1-iodo-4-(trifluoromethyl)benzene base-catalyzed isomerization; the reaction was run according to the general procedure; 9.7 mg (0.058 mmol) of TMB was added to the reaction solution: 65% 1-iodo-4-(trifluoromethyl)benzene and 13% 1-iodo-3-(trifluoromethyl)benzene.

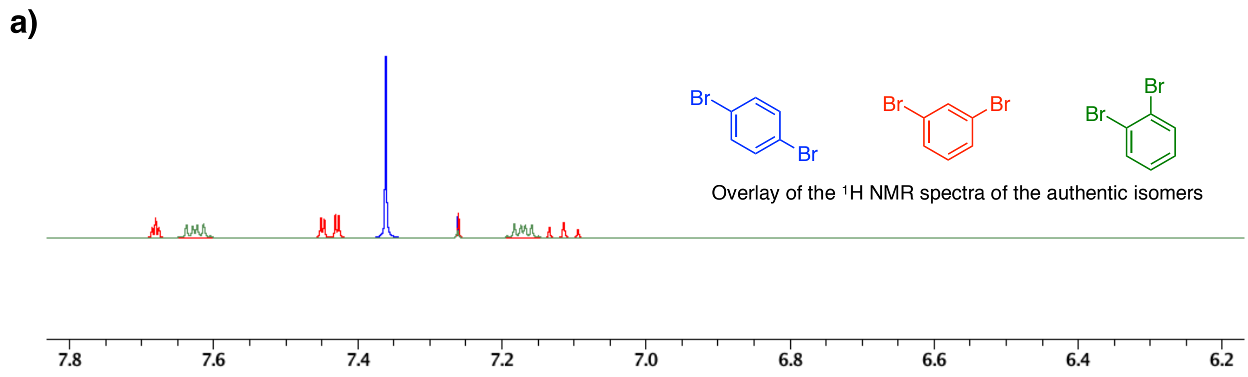

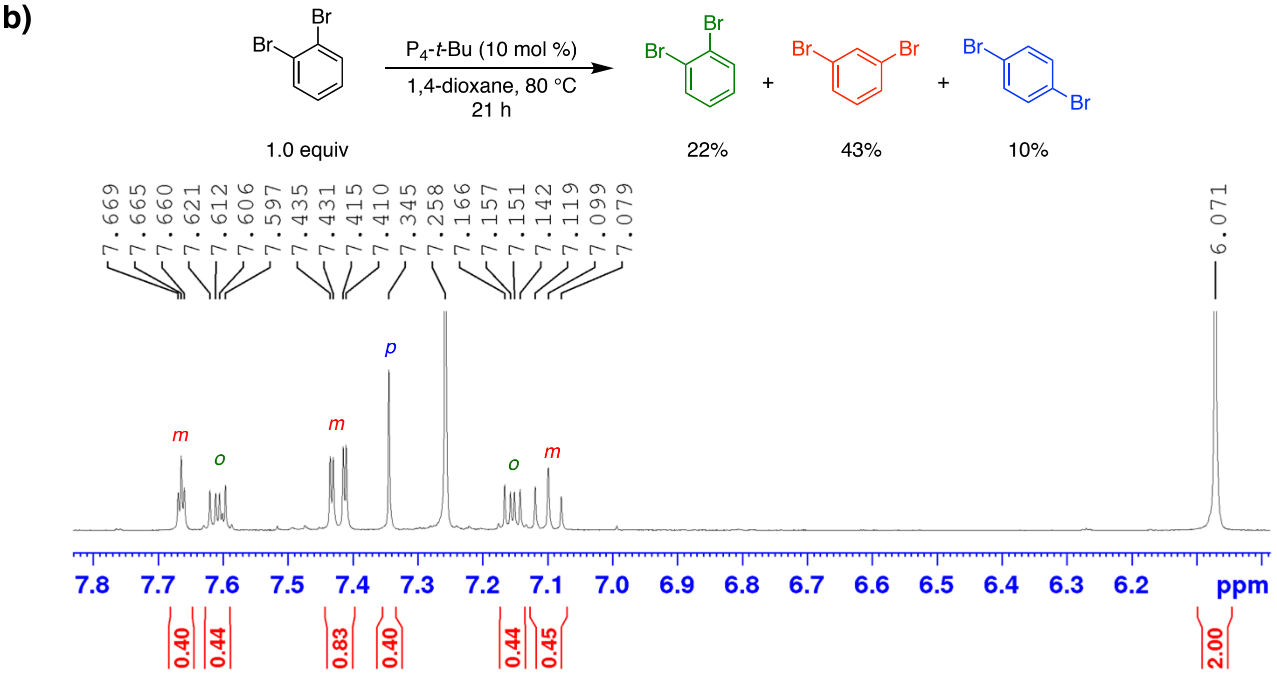

Figure S5: (**a**) Overlay of the ^{1}H NMR spectra of the authentic isomers of 1,2-dibromobenzene, 1,3-dibromobenzene, and 1,4-dibromobenzene. (**b**) ^{1}H NMR spectrum of the crude reaction solution of 1,2-dibromobenzene base-catalyzed isomerization; the reaction was run according to the general procedure; 11.2 mg (0.067 mmol) of TMB was added to the reaction solution: 22% 1,2-dibromobenzene, 43% 1,3-dibromobenzene, 10% 1,4-dibromobenzene.

a)

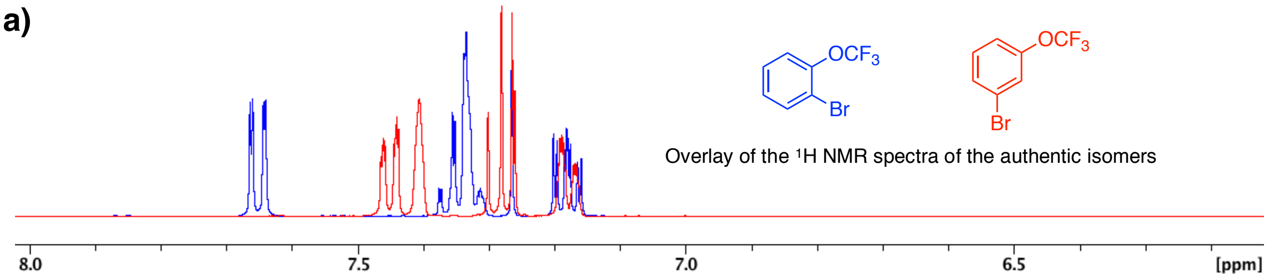

b)

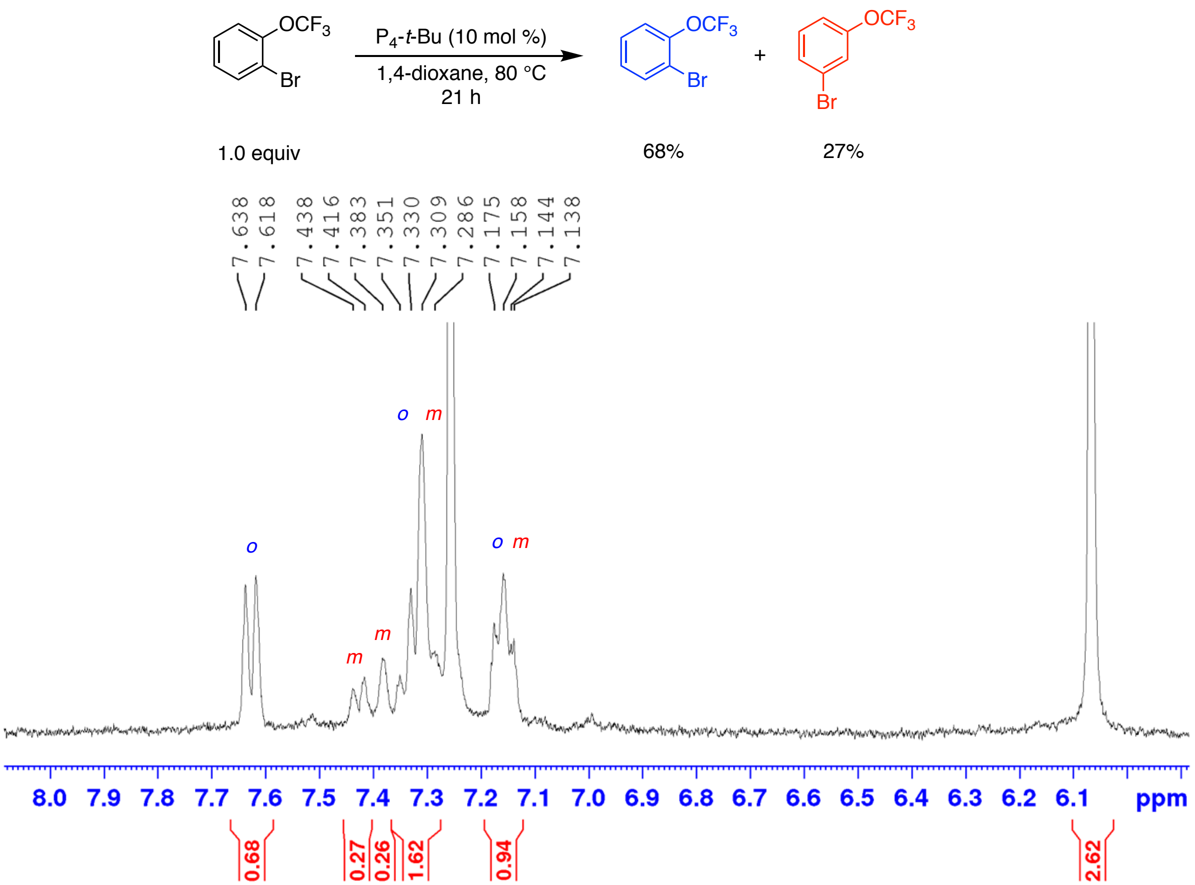

Figure S6: (**a**) Overlay of the ^{1}H NMR spectra of the authentic isomers of 1-bromo-2-(trifluoromethoxy)benzene and 1-bromo-3-(trifluoromethoxy)benzene. (**b**) ^{1}H NMR spectrum of the crude reaction solution of 1-bromo-2-(trifluoromethoxy)benzene base-catalyzed isomerization; the reaction was run according to the general procedure; 14.7 mg (0.087 mmol) of TMB was added to the reaction solution: 68% 1-bromo-2-(trifluoromethoxy)benzene and 27% 1-bromo-3-(trifluoromethoxy)benzene.

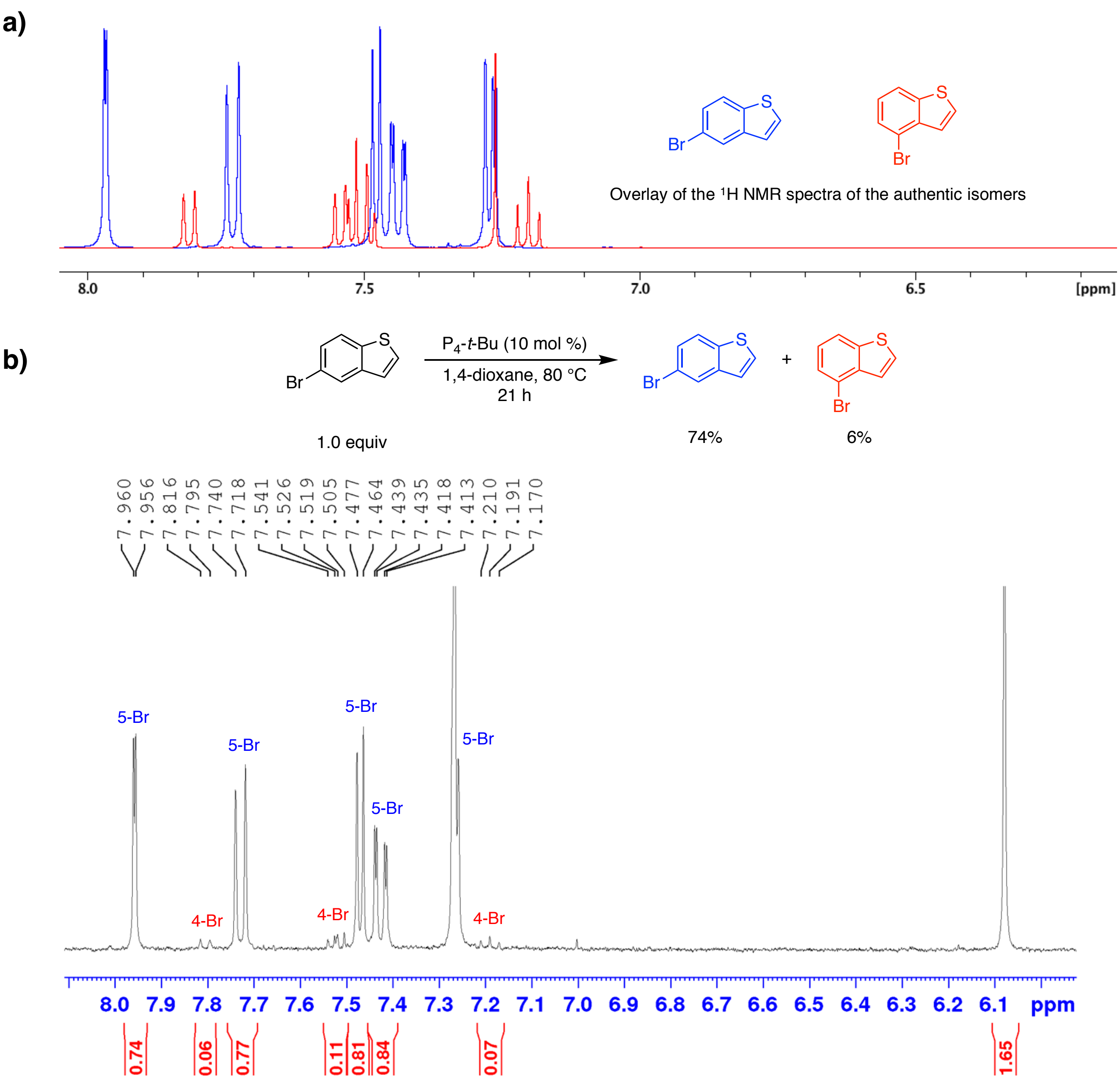

Figure S7: (**a**) Overlay of the ^{1}H NMR spectra of the authentic isomers of 5-bromobenzo[*b*]thiophene and 4-bromobenzo[*b*]thiophene. (**b**) ^{1}H NMR spectrum of the crude reaction solution of 5-bromobenzo[*b*]thiophene base-catalyzed isomerization; the reaction was run according to the general procedure using 38 µl (0.03 mmol, 0.3 equiv) of P_4-*t*-Bu; 9.3 mg (0.055 mmol) of TMB was added to the reaction solution: 74% 5-bromobenzo[*b*]thiophene and 6% 4-bromobenzo[*b*]thiophene.

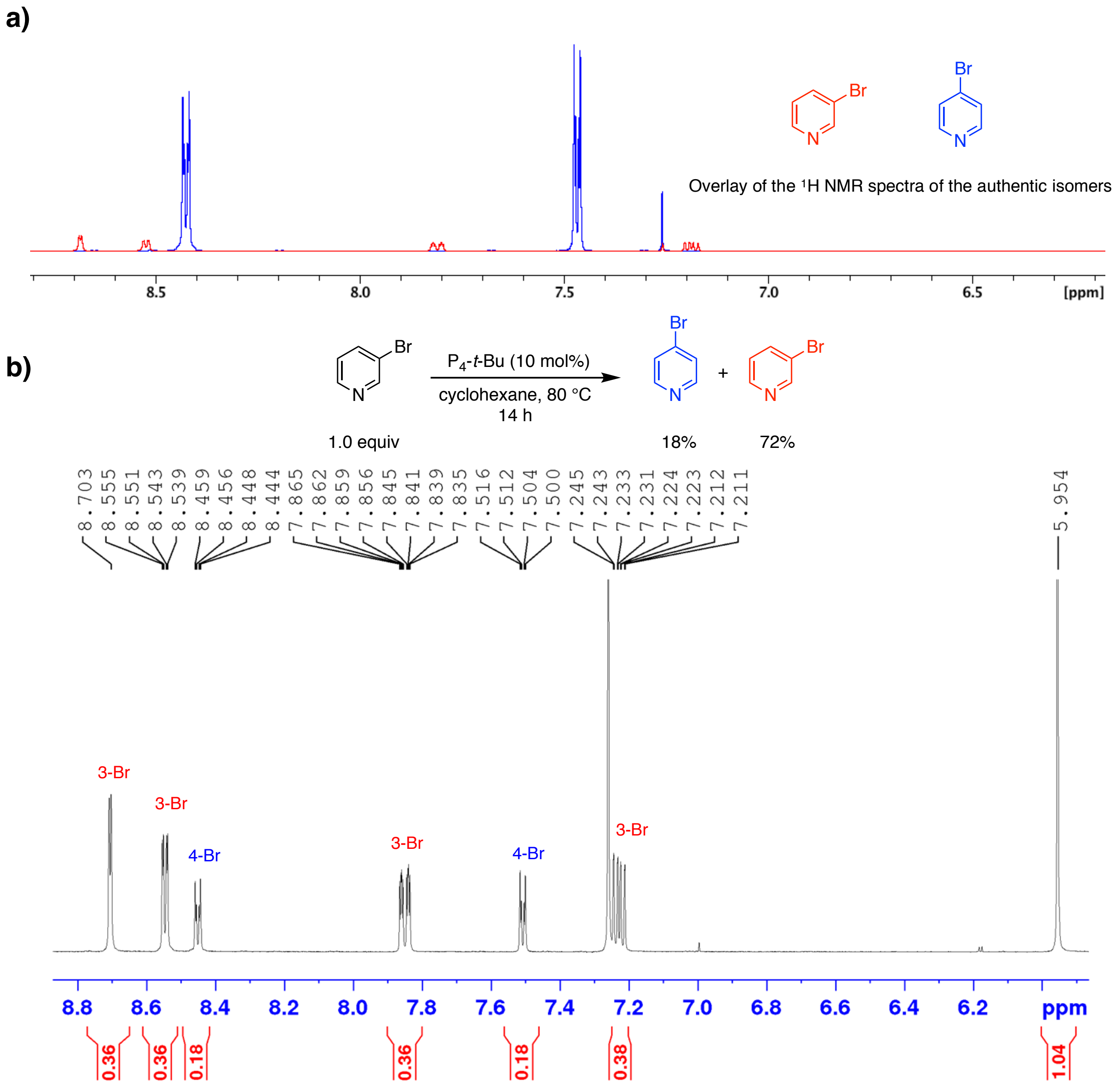

Figure S8: (**a**) ^{1}H NMR spectra of the authentic isomers of 3-bromopyridine and 4-bromopyridine. (**b**) ^{1}H NMR spectrum of the crude reaction solution of 3-bromopyridine base-catalyzed isomerization; the reaction was run according to the general procedure with stirring for 14 h at 80 °C in cyclohexane solvent (0.2 mL, 0.5 M), the reaction was quenched with acetic acid (ca. 250 µl) and 11 µl (0.10 mmol) of 1,1,2,2-tetrachloroethane (δ = 5.95 ppm) was added as internal standard to the reaction solution: 72% 3-bromopyridine and 18% 4-bromopyridine.

III. Optimization of the 4-Selective Etherification of 3-Bromopyridine

(a) Evaluation of changes in optimal base, solvent, temperature, and concentration

Preliminary experiments varying base and solvent suggested KOH with 18-crown-6 in DMAc could promote a 4-selective etherification reaction between 3-bromopyridine and 2-ethyl-1-hexanol. The optimal conditions were identified using pyridine:alcohol stoichiometry studies and KBr additive experiments that are described below in Section IIIb and IIIc, respectively. The optimized conditions are provided in the table below in comparison to specific changes of reagents or conditions used.

General procedure for condition variation: Inside a N_2 filled glovebox, a 4 mL vial (ThermoFisher, C4015-1) was charged with a magnetic stir, 3-bromopyridine (1.0 – 1.5 equiv), 2-ethyl-1-hexanol (13.0 mg, 0.1 mmol, 1.0 equiv), solvent (see Table **S1** below), KBr (6.0 mg, 0.05 mmol, 0.5 equiv.), 18-crown-6 (92.5 mg, 0.35 mmol, 3.5 equiv), and base (see Table **S1** below) in successive order. The vial was sealed with a PTFE-lined screw cap (ThermoFisher, C4015-1A), removed from the glovebox, and placed into a preheated 80 °C aluminum reaction block. The solution was stirred for 15 h at 80 °C and then allowed to cool to rt. 1,3,5-Trimethoxybenzene internal standard was then measured into the crude solution. A small aliquot was then removed and injected into a NMR tube and constituted in $CDCl_3$ (0.6 mL). ^1H NMR spectroscopy (400 MHz, $CDCl_3$) was used to determine the 4:3 selectivity and yield of each reaction using the process described in Section IIIb.

standard conditions:

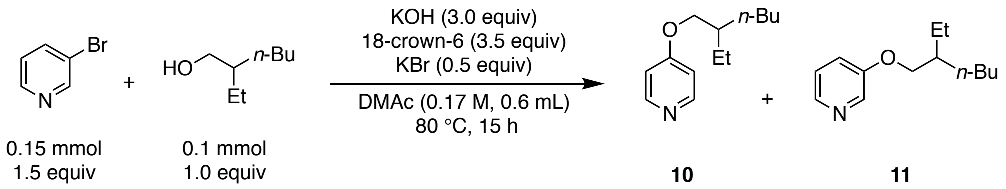

Entry	Change from the "standard conditions"	yield of 10	4:3 selectivity (10:11)
1	none	88%	>10:1
2	3.0 equiv NaOH used instead of KOH	0%	-
3	3.0 equiv CsOH · H_2O used instead of KOH	86%	>10:1
4	2.0 equiv of KOH/18-crown-6	81%	10:1
5	1.2 equiv P_4-*t*-Bu at rt instead of KOH at 80 °C	62%	4.4:1
6[a]	2.0 equiv KO-*t*-Bu/18-crown-6 used instead of KOH	62%	10:1
7[b]	0.5 M instead of 0.17 M in DMAc	84%	7.1:1
8[b]	1.0M instead of 0.17 M in DMAc	77%	4.5:1
9	99.8% KOH Pellets used instead of 85% KOH	86%	>10:1
10	Reaction run at 40 °C instead of 80 °C	70%	8.8:1
11	Reaction run open to air	66%	10:1
12	DME used as solvent instead of DMAc	28%	1.6:1
13	PhMe used as solvent instead of DMAc	20%	1.1:1
14	no 18-crown-6	40%	4.0:1
15	3.5 equiv DME instead of 18-crown-6	44%	5.5:1
16	3.5 equiv of diglyme instead of 18-crown-6	27%	3.4:1
17	3.5 equiv of tetraglyme instead of 18-crown-6	22%	5.5:1
18	3.5 equiv of *N*,*N*,*N*,*N*-tetramethylethylenediamine instead of 18-crown-6	58%	2.6:1

Table S1: Evaluation of the base, solvent, temperature, and concentration for the 4-selective etherification of 3-bromopyridine. [a]An 18% yield of 4-(*tert*-butoxy)pyridine was observed; this undesired side product was difficult to separate from the desired ether using silica gel chromatography. [b]Large quantities of solid precipitated from the reaction solution inhibiting adequate mixing.

(b) Effect of 3-bromopyridine and alcohol equivalency on substitution selectivity

Purpose: This experiment was performed to evaluate how the equivalency ratio of 3-bromopyridine:alcohol impacts the yield and 4-selectivity of the model substitution reaction.

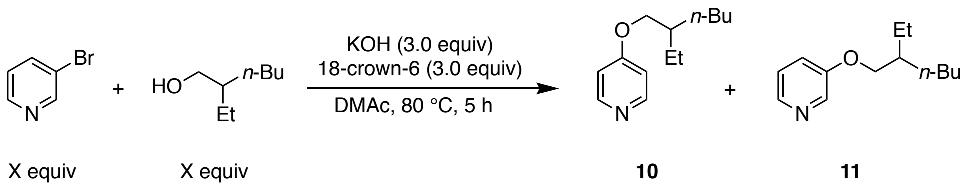

Procedure: An oven-dried 4 mL vial (ThermoFisher, C4015-1) was charged with a magnetic stir bar. Inside a N_2 filled glovebox, the vial was charged with appropriate quantity of 3-bromopyridine (see Table **S2**), the appropriate quantity of 2-ethyl-1-hexanol (see Table **S2**), DMAc (1.5 mL, 0.17 M in the limiting reagent), 18-crown-6 (198.2 mg, 0.75 mmol, 3.0 equiv), and KOH (42.1 mg, 0.75 mmol, 3.0 equiv) in successive order. The vials were sealed with a PTFE-lined screw cap (ThermoFisher, C4015-1A), removed from the glovebox, and placed into a preheated 80 °C aluminum reaction block. The reaction solutions were stirred at 80 °C for 5 h, at which point the solutions were removed from the aluminum reaction block and allowed to cool to rt. 1,3,5-Trimethoxybenzene internal standard was then added to each solution, and the yield of **10** and **11** under each condition was determined via analysis of the crude solutions by ^{1}H NMR spectroscopy (400 MHz, $CDCl_3$).

Evaluation of ^{1}H NMR yield and 4:3 substitution selectivity: Authentic samples of **10** and **11** were synthesized in order to accurately determine the 4:3 selectivity. The ^{1}H NMR spectra of the crude solutions of entries **1** and **6** from Table **S2** are provided in Figure **S9** and **S10**, respectively, to provide an example for how the yields of **10** and **11** were determined.

Entry	Equivalents of 3-bromopyridine	Equivalents of 2-ethyl-1-hexanol	yield of 10	yield of 11	4:3 selectivity (10:11)
**1**	1.0	4.0	38%	16%	2.4:1
**2**	1.0	3.0	52%	14%	3.7:1
**3**	1.0	2.0	55%	10%	5.5:1
**4**	1.0	1.0	57%	7%	8.1:1
**5**	2.0	1.0	88%	7%	12.6:1
**6**	3.0	1.0	90%	7%	12.9:1
**7**	4.0	1.0	83%	7%	11.9:1

Table S2: Results of the study on the effect of the equivalents ratio of 3-bromopyridine to 2-ethyl-1-hexanol on the selectivity and yield of the title reaction. **Note**: Under the conditions of entries **5, 6,** and **7** an appreciable yield of 4-bromopyridine (≈ 25% yield) was observed.

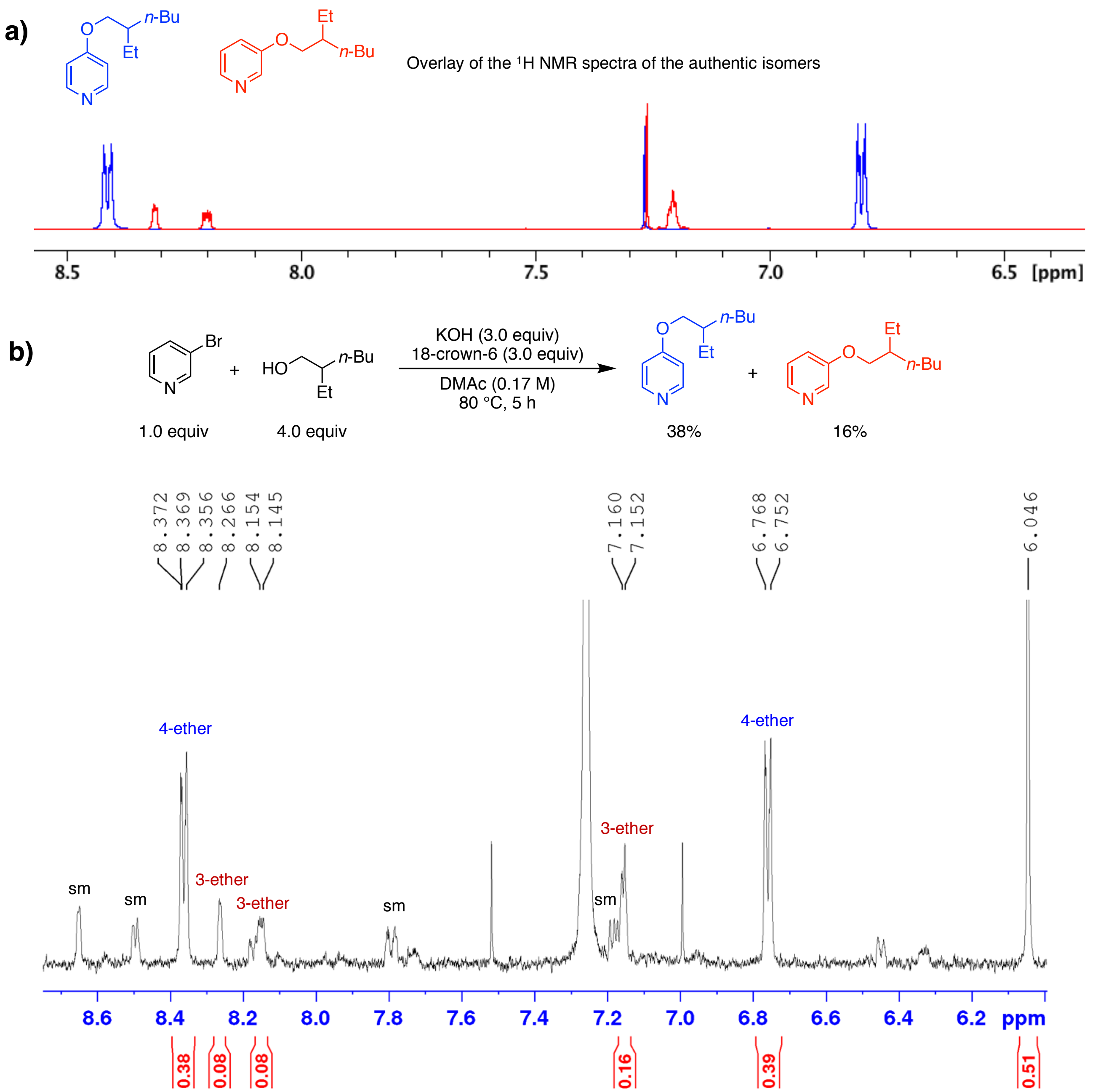

Figure S9: (**a**) Overlay of the ^{1}H NMR spectra of authentic 4-((2-ethylhexyl)oxy)pyridine and 3-((2-ethylhexyl)oxy)pyridine. (**b**) ^{1}H NMR spectrum of the crude reaction solution from entry **1** in Table **S2**. The reaction was run according to the general procedure; 14.2 mg (0.084 mmol) of 1,3,5-trimethoxybenzene used as internal standard: 38% yield of 4-((2-ethylhexyl)oxy)pyridine and 16% yield of 3-((2-ethylhexyl)oxy)pyridine (2.4:1 selectivity); sm = starting material.

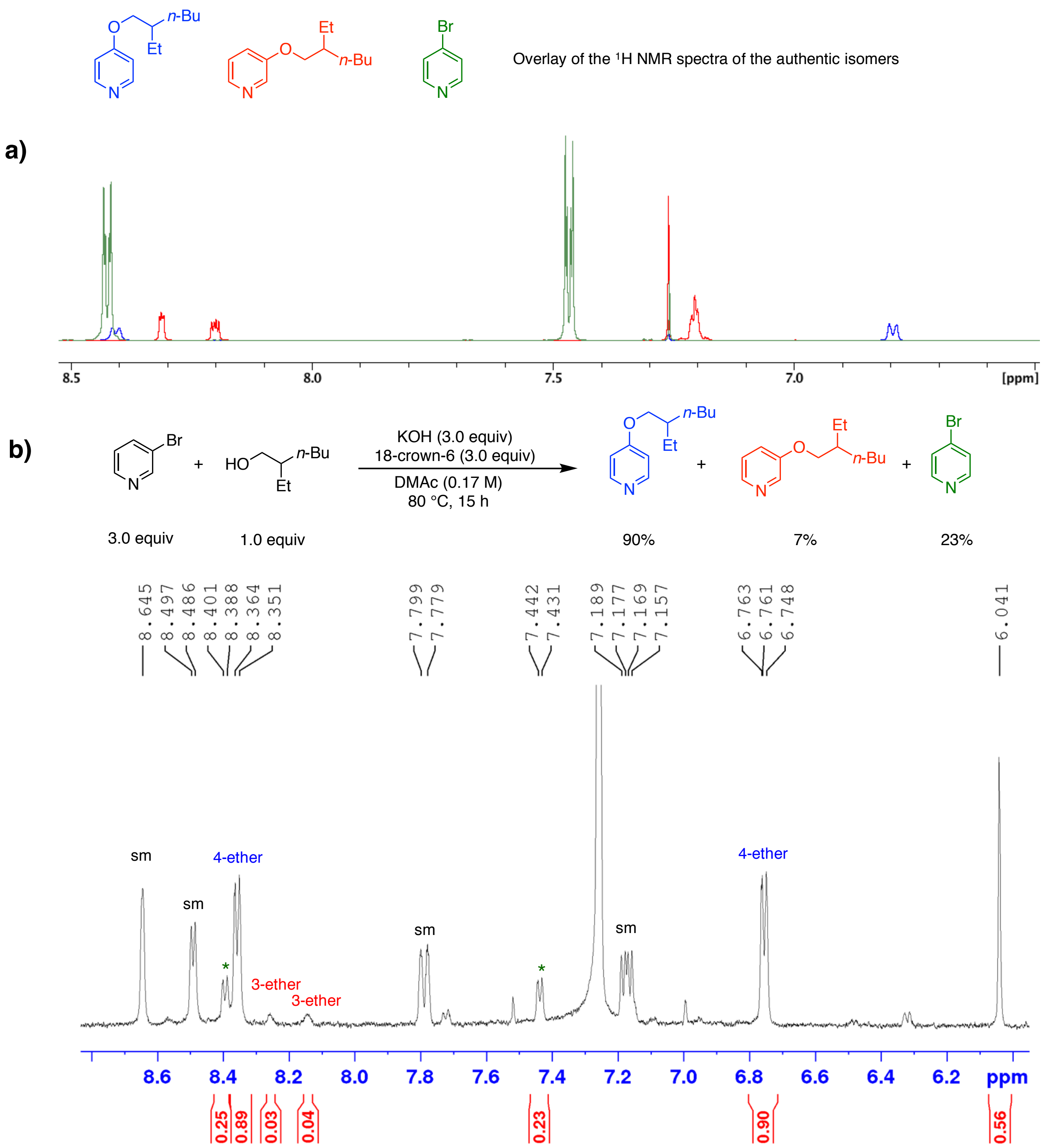

Figure S10: (**a**) Overlay of the ^{1}H NMR spectra of authentic 4-((2-ethylhexyl)oxy)pyridine and 3-((2-ethylhexyl)oxy)pyridine. (**b**) ^{1}H NMR spectrum of the crude reaction solution from entry **6** in Table **S2**. The reaction was run according to the general procedure; 15.8 mg (0.094 mmol) of 1,3,5-trimethoxybenzene used as internal standard: 90% yield of 4-((2-ethylhexyl)oxy)pyridine and 7% yield of 3-((2-ethylhexyl)oxy)pyridine (>10:1 selectivity) with 23% yield of 4-bromopyridine (marked with green asterisk); sm = starting material.

(c) Effect of KBr on the product yield, selectivity, and mass balance

Purpose: This experiment was performed to evaluate how added KBr impacts the yield and 4-selectivity of the model substitution reaction. Note that these reactions were run with 2.0 equiv of KOH and the experiments in Section IIIb were run with 3.0 equiv of KOH.

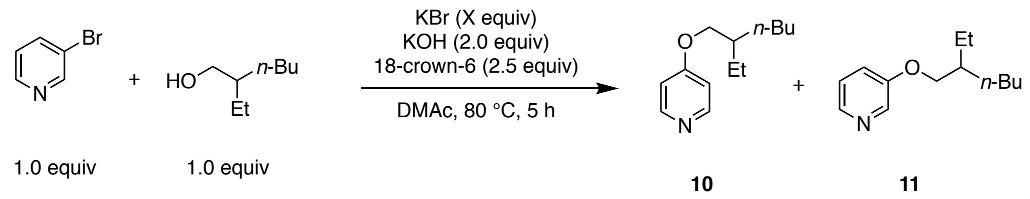

Procedure: An oven-dried 4 mL vial (ThermoFisher, C4015-1) was charged with a magnetic stir bar. Inside a N_2 filled glovebox, the vial was charged with 3-bromopyridine (39.5 mg, 0.25 mmol, 1.0 equiv), 2-ethyl-1-hexanol (32.6 mg, 0.25 mmol, 1.0 equiv), DMAc (1.5 mL, 0.17 M), 18-crown-6 (165.2 mg, 0.625 mmol, 2.5 equiv), the appropriate quantity of KBr (see Table **S3**), and KOH (28.1 mg, 0.50 mmol, 2.0 equiv) in successive order. The vials were sealed with a PTFE-lined screw cap (ThermoFisher, C4015-1A), removed from the glovebox, and placed into a preheated 80 °C aluminum reaction block. The reaction solutions were stirred at 80 °C for 5 h, at which point the reaction vials were removed from the aluminum reaction block and allowed to cool to rt. 1,3,5-Trimethoxybenzene internal standard was then added to each solution, and the yield of **10** and **11** under each condition was determined via analysis of the crude solutions by ^{1}H NMR spectroscopy (400 MHz, $CDCl_3$).

Entry	Equivalents of KBr	yield of 10	yield of 11	4:3 selectivity (10:11)	Mass balance
**1**	0	60%	7%	8.6:1	72%
**2**	0.1	62%	7%	8.9:1	75%
**3**	0.2	67%	6%	11.1:1	78%
**4**	0.3	65%	5%	13.0:1	78%
**5**	0.4	72%	5%	14.4:1	84%
**6**	0.5	71%	5%	14.2:1	81%
**7**[a]	1.0	72%	5%	14.4:1	81%

Table S3: Results of the effect of added KBr on the product yield, selectivity, and mass balance for the model reaction. [a]3.0 equiv of 18-crown-6 (198.2 mg, 0.75 mmol) used.

Results: A significant increase in yield of the 4-ether product and 4:3 selectivity were observed as the equivalents of KBr increased up to 0.5 equiv (see Table **S3**).

IV. Description of Mechanistic Studies on 3-Bromopyridine Isomerization

(a) Furan cycloaddition experiment under optimized basic conditions

Purpose: A known trap for 3,4-pyridyne is a cycloaddition reaction with furan.[1] We included an excess of furan under the reaction conditions to observe if the [4+2] cycloaddition adduct (**13** below) is formed.

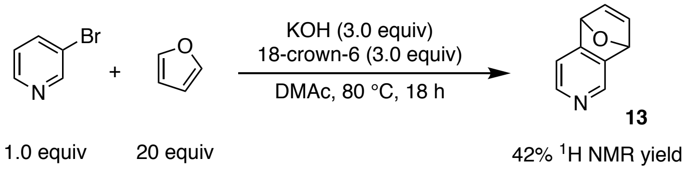

Procedure: An oven-dried 4 mL vial (ThermoFisher, C4015-1) was charged with a magnetic stir bar. Inside a N_2 filled glovebox, the vial was charged with 3-bromopyridine (15.8 mg, 0.1 mmol, 1.0 equiv), furan (146 µl, 2.0 mmol, 20 equiv), DMAc (0.6 mL, 0.17 M) 18-crown-6 (79.3 mg, 0.3 mmol, 3.0 equiv), and KOH (16.8 mg, 0.3 mmol, 3.0 equiv) in successive order. The vial was sealed with a PTFE-lined screw cap (ThermoFisher, C4015-1A), removed from the glovebox, and placed into a preheated 80 °C aluminum reaction block. The solution was stirred at 80 °C for 18 h, at which point the reaction vial was removed from the aluminum reaction block and allowed to cool to rt. 1,3,5-Trimethoxybenzene internal standard (8.9 mg, 0.053 mmol) was then added to the solution, and the yield of **13** was determined by ^{1}H NMR spectroscopy (400 MHz, $CDCl_3$, 42% yield). The observed ^{1}H NMR and ^{13}C NMR signals are consistent with literature reports for **13**.[1]
^{1}H NMR (400 MHz, $CDCl_3$) δ 8.45 (s, 1H), δ 8.29 (d, J = 4.6 Hz, 1H), 7.24 (d, J = 4.6 Hz, 1H), 7.06 (dd, J = 1.8 Hz, 5.6 Hz, 1H), 6.99 (dd, J = 1.8 Hz, 5.6 Hz, 1H), 5.82 (m, 1H), 5.73 (m, 1H).
^{13}C NMR (101 MHz, $CDCl_3$) δ 159.2, 147.8, 144.5, 143.8, 142.3, 140.2, 116.5, 82.1, 81.0.

(b) Effect of the addition of KCl and KI on product yield and selectivity

Purpose: After observing that KBr improved the product yield and selectivity for the model etherification reaction (see Table **S3**), we next investigated how addition of potassium iodide (KI) and potassium chloride (KCl) impacts the yield and selectivity of the reaction.

Procedure: An oven-dried 4 mL vial (ThermoFisher, C4015-1) was charged with a magnetic stir bar. Inside a N_2 filled glovebox, the vial was charged with 3-bromopyridine (39.5 mg, 0.25 mmol, 1.0 equiv), 2-ethyl-1-hexanol (32.6 mg, 0.25 mmol, 1.0 equiv), DMAc (1.5 mL, 0.25 M), 18-crown-6 (165.2 mg, 0.625 mmol, 3.5 equiv), and KCl (18.7 mg, 0.25 mmol, 1.0 equiv), KBr (29.8 mg, 0.25 mmol, 1.0 equiv) or KI (41.5 mg, 0.25 mmol, 1.0 equiv) in successive order. KOH (42.1 mg, 0.75 mmol, 3.0 equiv) was then added. The vials were sealed with a PTFE-lined screw cap (ThermoFisher, C4015-1A), removed from the glovebox, and placed into a preheated 80 °C aluminum reaction block. The reaction solutions were stirred at 80 °C for 15 h, at which point they were removed from the aluminum reaction block and allowed to cool to rt. 1,3,5-

Trimethoxybenzene internal standard was then added to each solution, and the yield of **10** and **11** under each condition was determined via analysis of the crude solutions by ^{1}H NMR spectroscopy (400 MHz, $CDCl_3$).

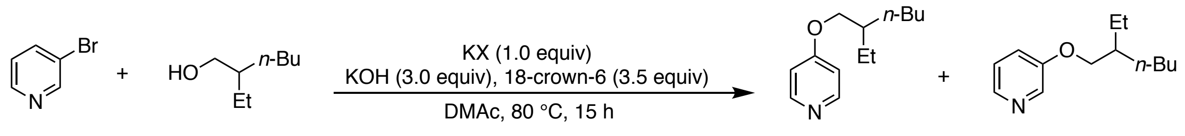

1.0 equiv 1.0 equiv **10** **11**

KX	yield of 10	yield of 11	4:3 selectivity (10:11)
KCl	60%	7%	8.6:1
KBr	80%	7%	>10:1
KI	57%	7%	8.1:1
no KX	57%	7%	8.3:1

Table S4: Results of the effect of halide salt addition on the 4-selectivity and product yield.

Results: Addition of KCl or KI had a negligible effect on the yield and 4-selectivity of the reaction. The yield and 4-selectivity under either condition was comparable to a control in which no halide salt was included. Addition of KBr to the reaction improved the yield of the 4-alkoxylated product and the 4-selectivity of the reaction.

(c) Determination of the reaction profile

Purpose: This experiment was performed to analyze components of the reaction solution at different time points to observe the formation and consumption of potential reaction intermediates.

Procedure: An oven-dried 25 mL round bottom flask was charged with a magnetic stir bar, 3-bromopyridine (237 mg, 1.5 mmol, 1.5 equiv), and dibenzyl ether (99.2 mg, 0.5 mmol, 0.5 equiv, used as internal standard). The flask was then brought into a N_2 filled glovebox and 2-ethyl-1-hexanol (130.2 mg, 1 mmol, 1.0 equiv), DMAc (6.0 mL, 0.17 M), KBr (59.5 mg, 0.5 mmol, 0.5 equiv), 18-crown-6 (1.19g, 4.5 mmol, 4.5 equiv), and KOH (224.4 mg, 4.0 mmol, 4.0 equiv) were added to the flask in successive order. The flask was sealed with a Fisherbrand® red septum stopper (Cat. No. FB57875) and the edges of the septum were lined with Parafilm®. The flask was put under positive pressure using a balloon filled with N_2, and then placed into a preheated 80 °C silicon oil bath. At the given time intervals (see Table **S5**), a small aliquot (ca. 25 µl) of the reaction solution was removed using a syringe, injected into an NMR tube, and constituted in $CDCl_3$ (0.6 mL). The mass balance, selectivity, and amount of 4-bromopyridine, **10**, and **11** was then

determined via ^{1}H NMR (400 MHz, $CDCl_3$) using the dibenzyl ether (which is inert under the reaction conditions) as the internal standard.

Time (min)	4-bromopyridine (%)	10 (%)	11 (%)	4:3 ratio (10:11)
2	3%	14%	3%	4.7:1
5	4%	34%	4%	8.5:1
10	5%	45%	5%	9.0:1
15	6%	51%	5%	10.2:1
30	6%	62%	6%	10.3:1
60	6%	73%	6%	12.2:1
105	4%	76%	6%	12.7:1
180	4%	81%	7%	11.6:1
300	1%	84%	7%	12.0:1
420	0%	85%	7%	12.1:1

Table S5: Results of the reaction profile on the 4-selective etherification of 3-bromopyridine.

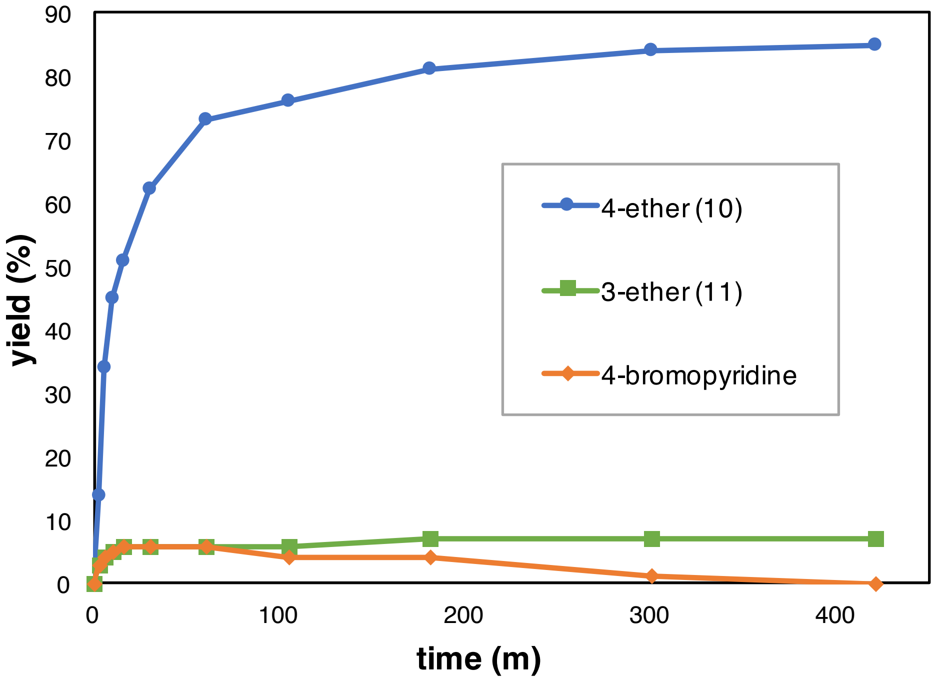

Figure S11. Reaction profile obtained for the model etherification reaction of 3-bromopyridine.

Result: Upon monitoring the reaction over time, we observed a buildup of 4-bromopyridine that is ultimately consumed by the end of the reaction. No other discernable intermediates were observed.

(d) Conversion of 3-iodopyridine to 4- and 3-bromopyridine with KBr additive

Purpose: A possible explanation for the positive effect of KBr on the yield and selectivity of the etherification reaction is addition of bromide to a 3,4-pyridyne intermediate. We sought to investigate if there is a yield of 3- or 4-bromopyridine when 3-iodopyridine is stirred under the basic reaction conditions in the presence of KBr.

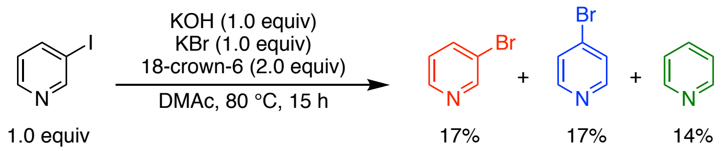

Procedure: An oven-dried 4 mL vial (ThermoFisher, C4015-1) was charged with a magnetic stir bar. Inside a N_2 filled glovebox, the vial was charged with 3-iodopyridine (20.5 mg, 0.10 mmol, 1.0 equiv), DMAc (1.5 mL, 0.25 M), 18-crown-6 (52.8 mg, 0.20 mmol, 2.0 equiv), KBr (11.9 mg, 0.10 mmol, 1.0 equiv), and KOH (5.6 mg, 0.10 mmol, 1.0 equiv) in successive order. The vials were sealed with a PTFE-lined screw cap (ThermoFisher, C4015-1A), removed from the glovebox, and placed into a preheated 80 °C aluminum reaction block. The reaction solutions were stirred at 80 °C for 15 h, at which point they were removed from the aluminum reaction block and allowed

to cool to rt. 1,3,5-Trimethoxybenzene internal standard (15.0 mg, 0.089 mmol) was then added to the solution, and the yield of 3-bromopyridine and 4-bromopyridine was determined via analysis of the crude solution by ^{1}H NMR spectroscopy (400 MHz, $CDCl_3$).

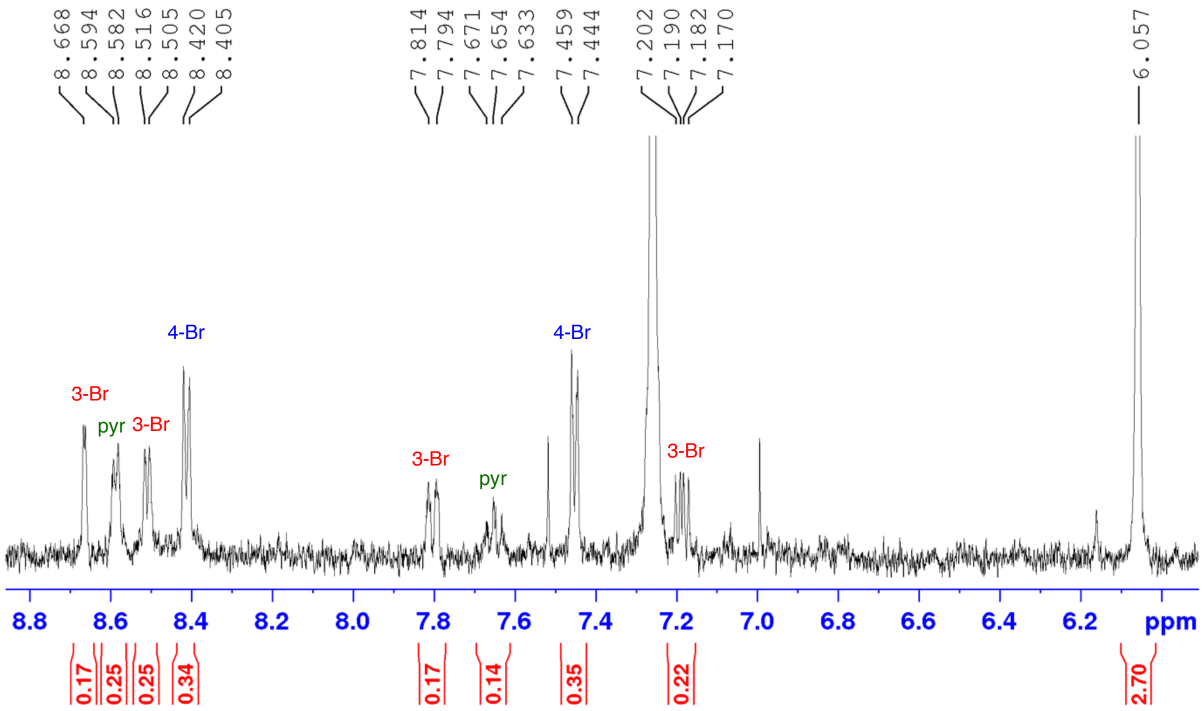

Figure S12: ^{1}H NMR spectrum of the crude reaction solution of the base-catalyzed substitution of 3-iodopyridine with KBr; 17% yield of 3-bromopyridine (marked in red), 17% yield of 4-bromopyridine (marked in blue), and 14% yield of pyridine (marked in green). **Note:** To support the identity that corresponds to each ^{1}H NMR signal above, we mixed authentic 3-bromopyridine, 4-bromopyridine, 3-iodopyridine, and 4-iodopyridine into 4 separate ^{1}H NMR samples of the crude solution. Only in the cases of 3-iodopyridine and 4-iodopyridine were new signals observed.

Supplementary Experiments and Notes: To corroborate that 3,4-pyridyne is generated under the reaction conditions for 3-iodopyridine, we performed a trapping experiment with furan analogous to Section IVa. Additionally, we stirred 3-iodopyridine under the conditions above with exclusion of KOH; under these conditions we observed no conversion of 3-iodopyridine.

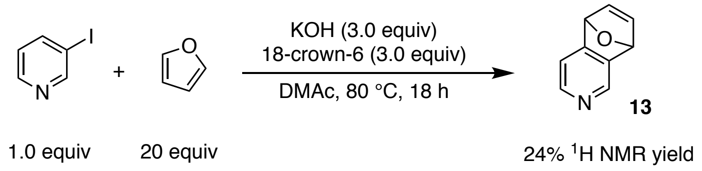

Procedure: An oven-dried 4 mL vial (ThermoFisher, C4015-1) was charged with a magnetic stir bar. Inside a N_2 filled glovebox, the vial was charged with 3-iodopyridine (20.5 mg, 0.1 mmol, 1.0 equiv), furan (146 µl, 2.0 mmol, 20 equiv), DMAc (0.6 mL, 0.17 M) 18-crown-6 (79.3 mg, 0.3 mmol, 3.0 equiv), and KOH (16.8 mg, 0.3 mmol, 3.0 equiv) in successive order. The vial was

sealed with a PTFE-lined screw cap (ThermoFisher, C4015-1A), removed from the glovebox, and placed into a preheated 80 °C aluminum reaction block. The solution was stirred at 80 °C for 18 h, at which point the solution was removed from the aluminum reaction block and allowed to cool to rt. 1,3,5-Trimethoxybenzene internal standard (8.8 mg, 0.052 mmol) was then added to the solution, and the yield of **13** was determined by ^{1}H NMR spectroscopy (400 MHz, $CDCl_3$, 24% yield). The ^{1}H NMR of **13** is consistent with that in Section IVa.

(d) Observation of the isomerization of 3-bromopyridine substrates from Table 1

Purpose: To demonstrate that 3-bromopyridines from Table **1** isomerize to 4-bromopyridines under the optimal reaction conditions, we subjected several substrates to the basic conditions in the absence of alcohol nucleophile.

Procedure: An oven-dried 4 mL vial (ThermoFisher, C4015-1) was charged with a magnetic stir bar. Inside a N_2 filled glovebox, the vial was charged with the appropriate 3- or 5-bromopyridine substrate (0.1 mmol, 1.0 equiv), DMAc (0.6 mL, 0.17 M), KBr (6.0 mg, 0.05 mmol, 0.5 equiv), 18-crown-6 (1.0-1.5 equiv), and KOH (5.6 mg, 0.1 mmol, 0.1 equiv) in successive order. The vial was sealed with a PTFE lined screw cap (ThermoFisher, C4015-1A), removed from the glovebox, and placed into a preheated 80 °C aluminum reaction block. The reaction solutions were stirred at 80 °C for 4 h. The reaction solutions were removed from the aluminum reaction block and allowed to cool to rt. 1,3,5-Trimethoxybenzene internal standard was then measured into each solution, and the yield of the starting material and the corresponding 4-bromopyridine was determined via analysis of the crude solutions by ^{1}H NMR spectroscopy (400 MHz, $CDCl_3$). **Note**: the structure of the corresponding 4-bromopyridine was confirmed by ^{1}H NMR analysis of authentic samples of the appropriate 4-bromopyridine or by direct isolation and ^{1}H NMR characterization of the 4-bromopyridine from the reaction solution. An overlay of the ^{1}H NMR spectra of the authentic 3-bromopyridine and 4-bromopyridine isomer for each substrate is provided above the ^{1}H NMR spectrum of each crude reaction solution.

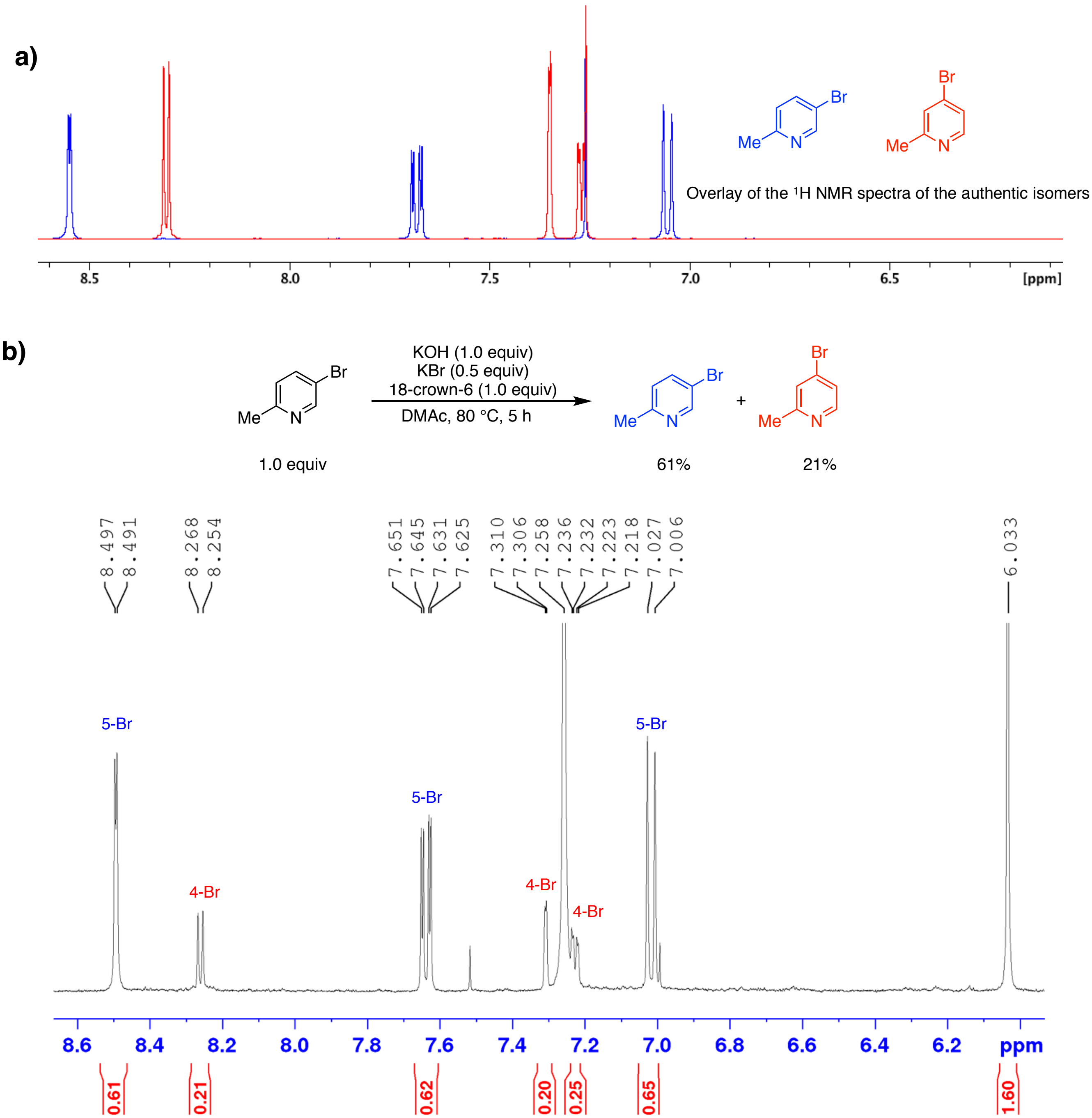

Figure S13: (**a**) ^{1}H NMR spectra of the authentic isomers of 5-bromo-2-methylpyridine and 4-bromo-2-methylpyridine. (**b**) ^{1}H NMR spectrum of the crude reaction solution of 5-bromo-2-methylpyridine base-catalyzed isomerization. The reaction was run according to the general procedure on 0.25 mmol scale using 43.0 mg (0.25 mmol) of 5-bromo-2-methylpyridine; 22.3 mg (0.133 mmol) of 1,3,5-trimethoxybenzene was added as internal standard to the reaction solution: 61% 2-methyl-5-bromopyridine and 21% 2-methyl-4-bromopyridine.

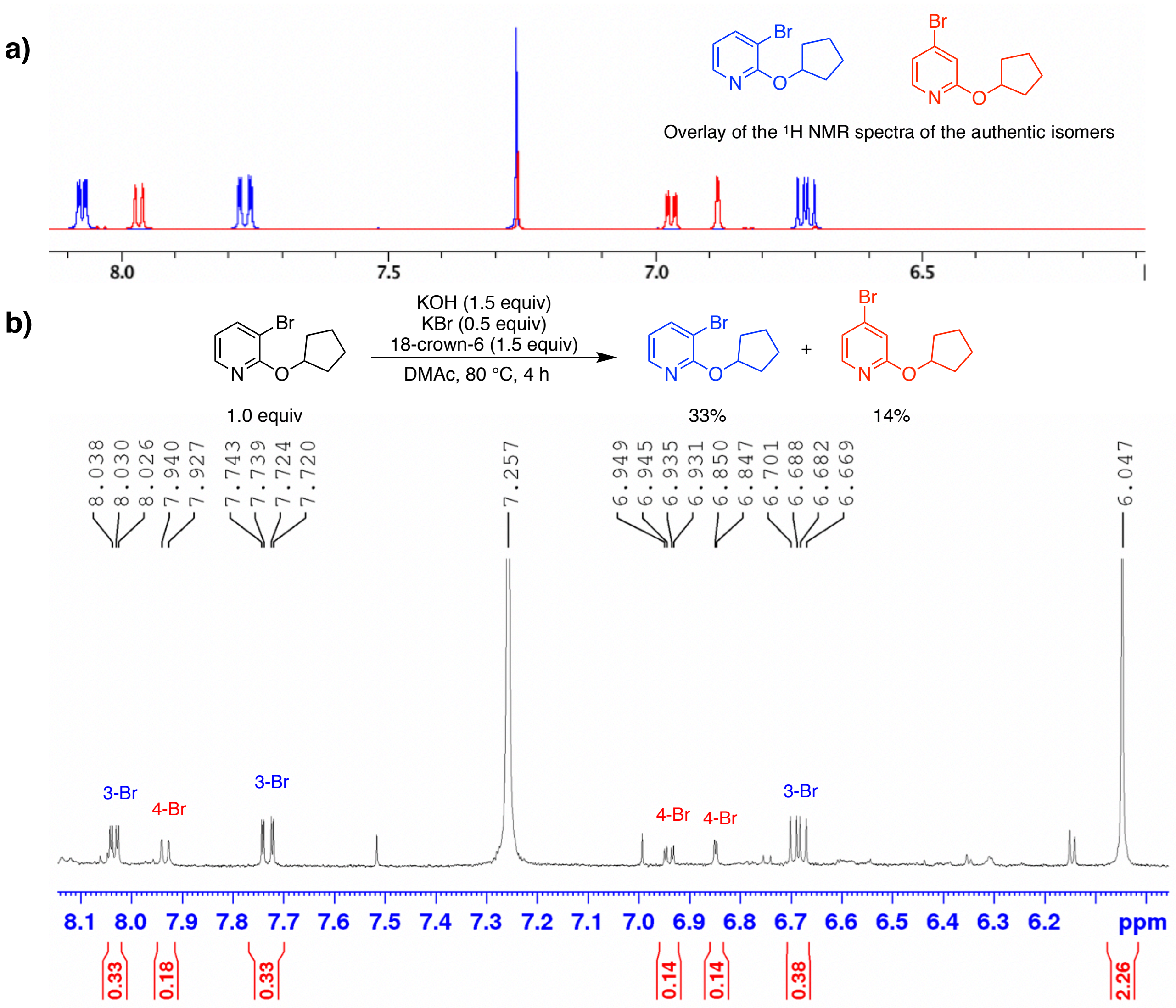

Figure S14: (**a**) ^{1}H NMR spectra of the authentic isomers of 3-bromo-2-(cyclopentyloxy)pyridine and 4-bromo-2-(cyclopentyloxy)pyridine. (**b**) ^{1}H NMR spectrum of the crude reaction solution of 3-bromo-2-(cyclopentyloxy)pyridine base-catalyzed isomerization; the reaction was run according to the general procedure using 1.5 equiv of KOH (8.4 mg, 0.15 mmol) and 1.5 equiv of 18-crown-6 (39.6 mg, 0.15 mmol); 12.7 mg (0.076 mmol) of 1,3,5-trimethoxybenzene was added as internal standard: 33% 3-bromo-2-(cyclopentyloxy)pyridine and 14% 4-bromo-2-(cyclopentyloxy)pyridine.

a)

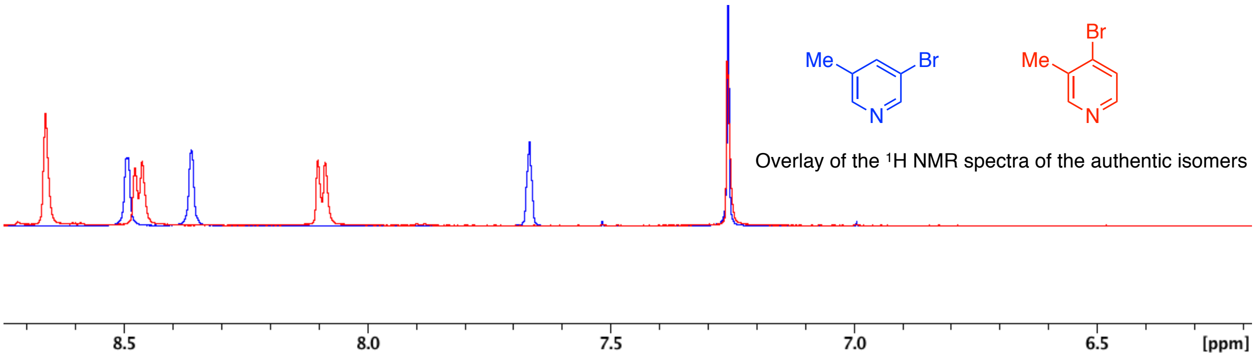
Me
Br
N
Me
Br
N
Overlay of the ¹H NMR spectra of the authentic isomers
8.5
8.0
7.5
7.0
6.5
[ppm]

b)

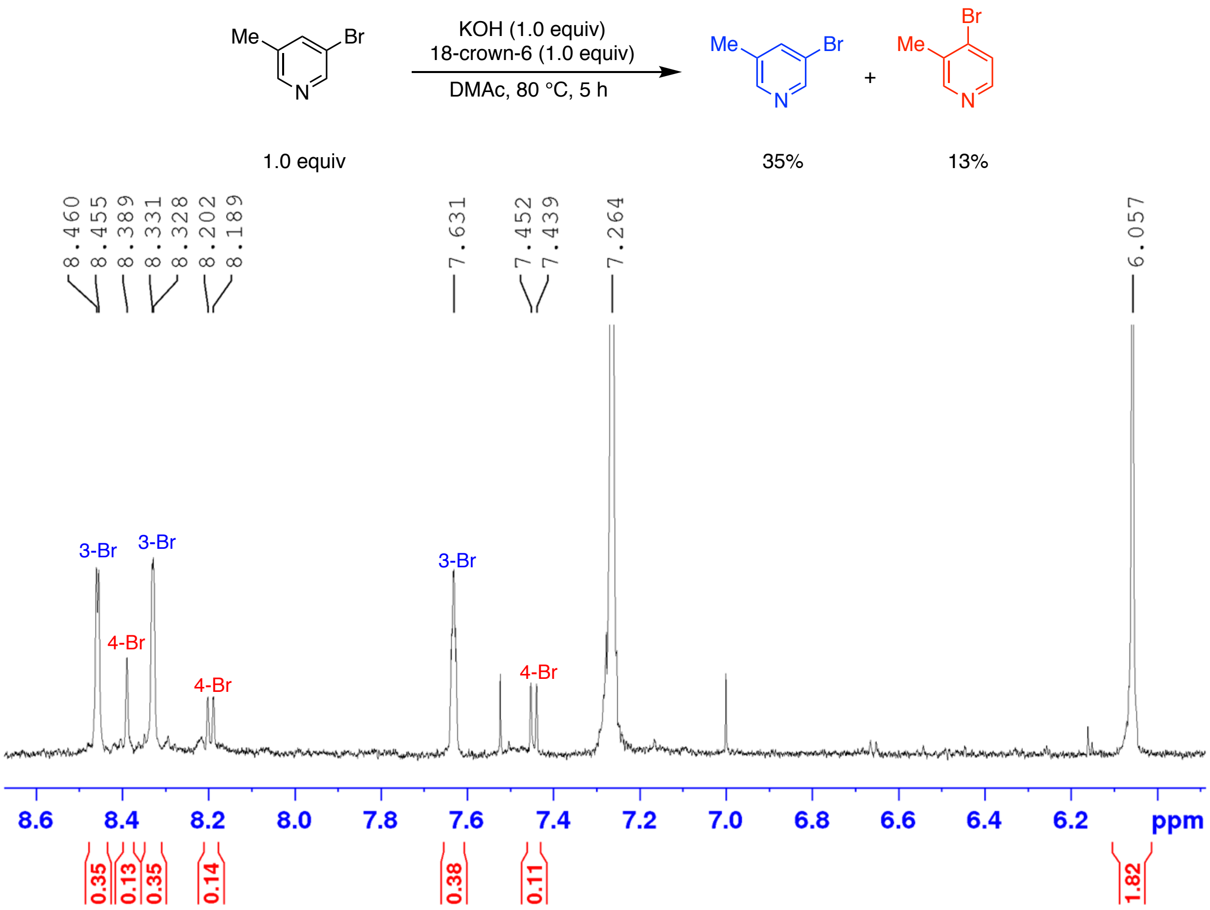
Me
Br
N
1.0 equiv
KOH (1.0 equiv)
18-crown-6 (1.0 equiv)
DMAc, 80 °C, 5 h
Me
Br
N
35%
+
Me
Br
N
13%
8.460
8.455
8.389
8.331
8.328
8.202
8.189
7.631
7.452
7.439
7.264
6.057
3-Br
3-Br
4-Br
4-Br
3-Br
4-Br
8.6
8.4
8.2
8.0
7.8
7.6
7.4
7.2
7.0
6.8
6.6
6.4
6.2
ppm
0.35
0.13
0.35
0.14
0.38
0.11
1.82

c)

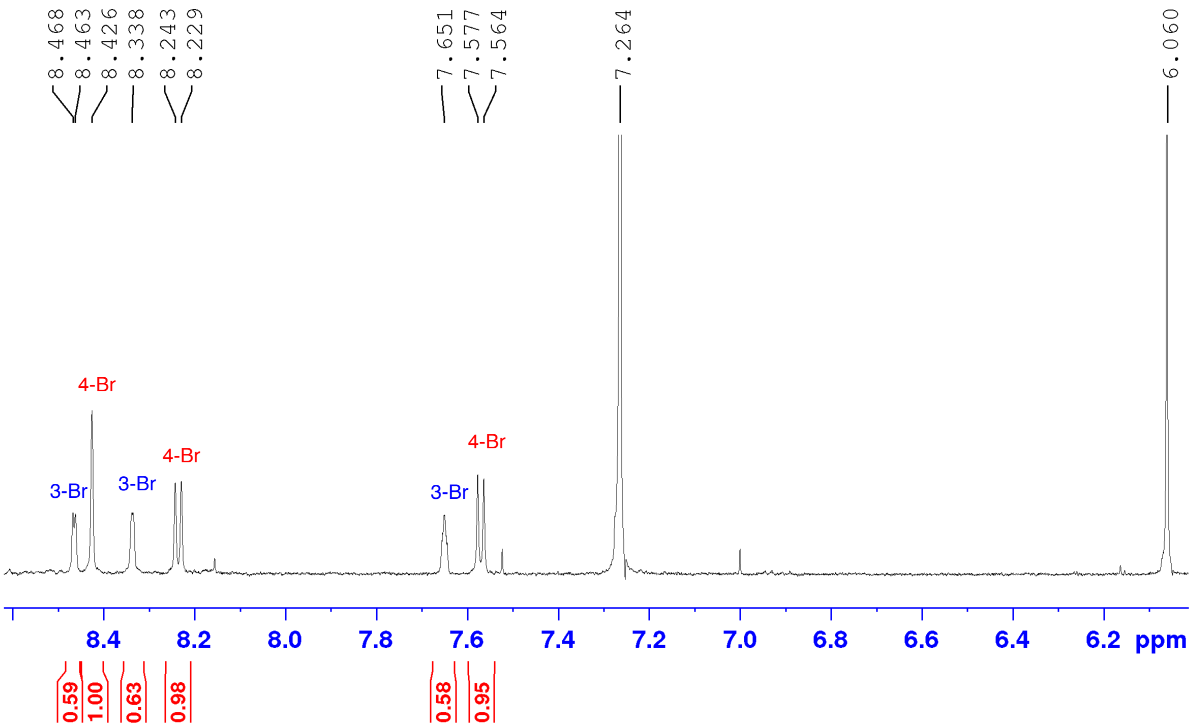

Figure S15: (**a**) ^{1}H NMR spectra of the authentic isomers of 3-bromo-5-methylpyridine and 4-bromo-3-methylpyridine. (**b**) ^{1}H NMR spectrum of the crude reaction solution of 3-bromo-5-methylpyridine base-catalyzed isomerization; the reaction was run according to the general procedure; 10.2 mg (0.061 mmol) of 1,3,5-trimethoxybenzene was added as internal standard to the reaction solution: 35% 3-bromo-5-methylpyridine and 13% 4-bromo-3-methylpyridine. **(c)** ^{1}H NMR spectrum of the crude reaction solution of 3-bromo-5-methylpyridine base-catalyzed isomerization with a small quantity of authentic 4-bromo-3-methylpyridine spiked into it. We note that the spectrum of 4-bromo-3-methylpyridine from (**a**) above does not overlay onto the ^{1}H NMR spectrum of the crude solution. When authentic 4-bromo-3-methylpyridine was spiked into the ^{1}H NMR sample of the crude solution, we observed an increase in the signals we labeled as corresponding to the 4-bromo-3-methylpyridine isomer. This is shown in spectrum (c).

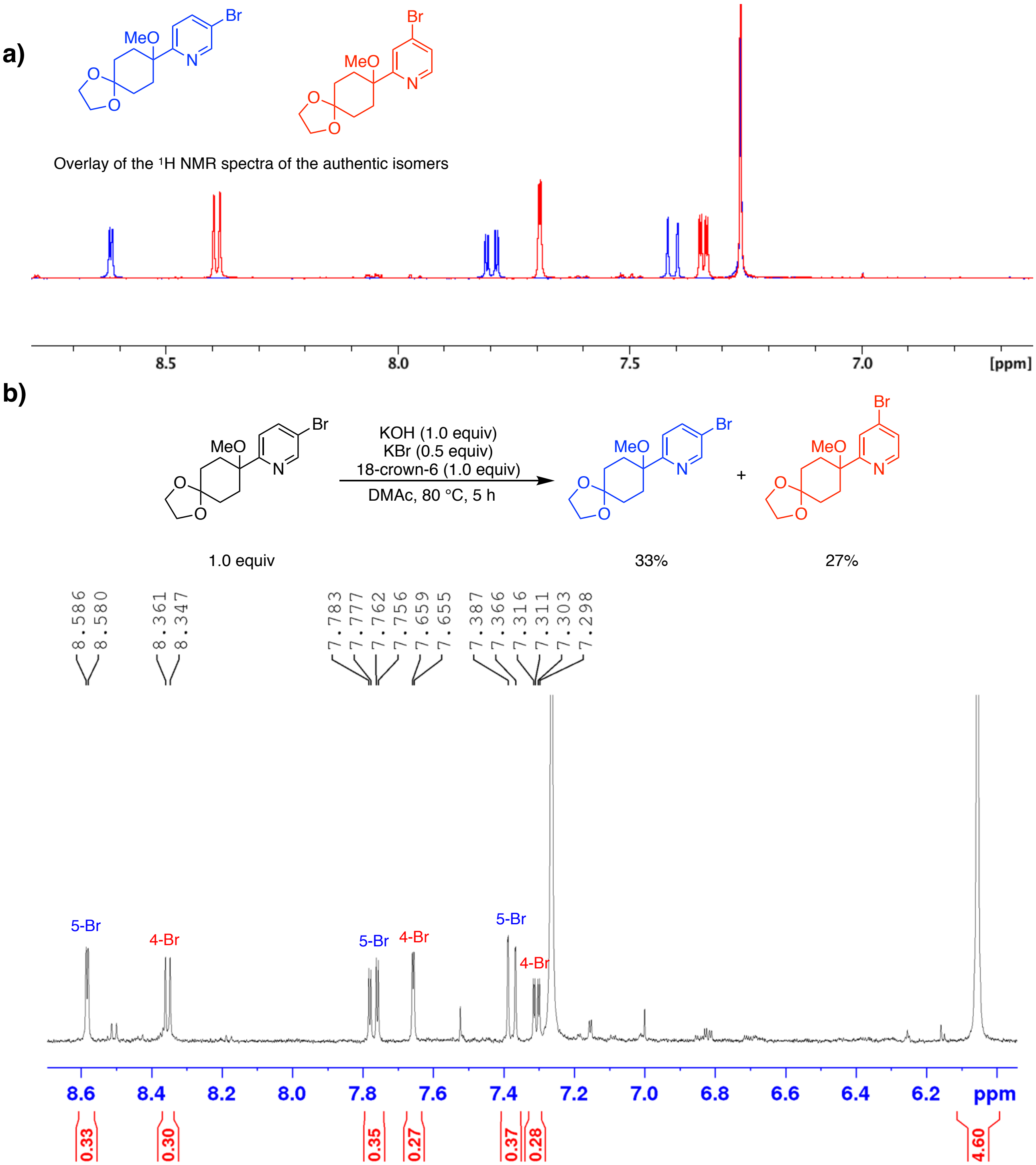

Figure S16: (**a**) ^{1}H NMR spectra of the authentic isomers of 5-bromo-2-(8-methoxy-1,4-dioxaspiro[4.5]decan-8-yl)pyridine and 4-bromo-2-(8-methoxy-1,4-dioxaspiro[4.5]decan-8-yl)pyridine. (**b**) ^{1}H NMR spectrum of the crude reaction solution of 5-bromo-2-(8-methoxy-1,4-dioxaspiro[4.5]decan-8-yl)pyridine base-catalyzed isomerization; the reaction was run according to the general procedure; 25.6 mg (0.152 mmol) of 1,3,5-trimethoxybenzene was added as internal standard to the reaction solution: 33% 5-bromo-2-(8-methoxy-1,4-dioxaspiro[4.5]decan-8-yl)pyridine and 27% 4-bromo-2-(8-methoxy-1,4-dioxaspiro[4.5]decan-8-yl)pyridine.

(e) Control test for the decomposition of 3-((2-ethylhexyl)oxy)pyridine (11) under reaction conditions

Purpose: To determine if the 3-alkoxypyridines are stable under the reaction conditions in Table 1.

Procedure: An oven-dried 4 mL vial (ThermoFisher, C4015-1) was charged with a magnetic stir bar. Inside a N_2 filled glovebox, the vial was charged with 3-((2-ethylhexyl)oxy)pyridine (20.8 mg, 0.1 mmol, 1.0 equiv), 2-ethyl-1-hexanol (3.3 mg, 0.025 mmol, 0.25 equiv), DMAc (0.6 mL, 0.17 M), KBr (6.0 mg, 0.05 mmol, 0.5 equiv), 18-crown-6 (26.4 mg, 0.1 mmol, 1.0 equiv), and KOH (5.6 mg, 0.1 mmol, 1.0 equiv) in successive order. The vial was sealed with a PTFE-lined screw cap (ThermoFisher, C4015-1A), removed from the glovebox, and placed into a preheated 80 °C aluminum reaction block. The reaction solution was stirred at 80 °C for 15 h. The solution was removed from the aluminum reaction block and allowed to cool to rt. 1,3,5-Trimethoxybenzene internal standard (13.9 mg, 0.083 mmol) was then added to the solution, and the yield of 3-((2-ethylhexyl)oxy)pyridine was determined via analysis of the crude solution by ^{1}H NMR spectroscopy (400 MHz, $CDCl_3$).

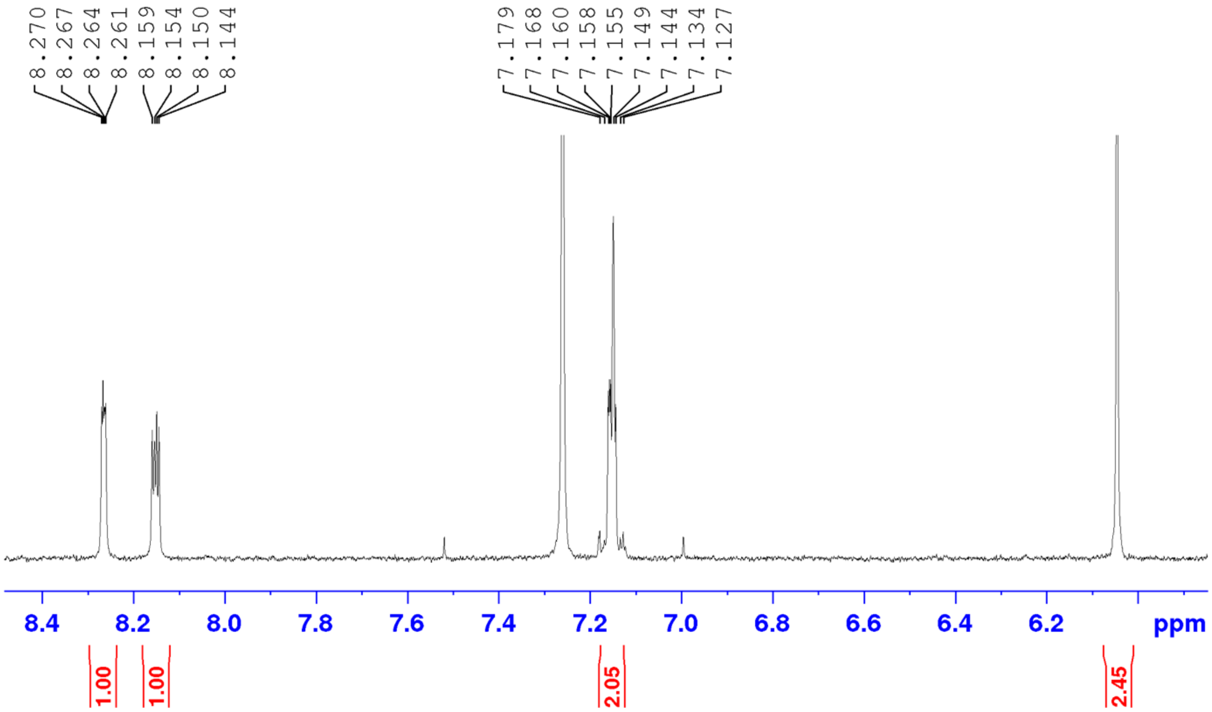

Figure S17: ^{1}H NMR spectrum for the subjection of **11** to the standard reaction conditions. 13.9 mg (0.083 mmol) of 1,3,5-trimethoxybenzene was added to reaction solution as internal standard. 100% of **11** can be accounted for in the ^{1}H NMR spectrum with no observable decomposition products.

V. General Procedure for the 4-Selective Substitution of 3/5-Bromopyridines

General procedure for 1 mmol scale reactions: KOH, KBr, and 18-crown-6 were stored in a N_2 filled glovebox at rt and used immediately upon removal from the glovebox. Outside of the glovebox, an oven-dried 25 or 50 mL round bottom flask was charged with a magnetic stir bar, 18-crown-6 (925.1 mg, 3.5 mmol, 3.5 equiv), KBr (59.5 mg, 0.5 mmol, 0.5 equiv), and KOH (168.3 mg, 3.0 mmol, 3.0 equiv). The flask was then sealed with a Fisherbrand® red septum stopper (Cat. No. FB57875) and the edges of the septum were then sealed with Parafilm®. The flask was evacuated and backfilled three times with N_2 and left under positive pressure using either a N_2 balloon or a Schlenk line. DMAc (4.0 mL) was then added via syringe under N_2 and the solution was stirred at rt (***Note***: there is usually some insoluble solid that remains in the bottom of the flask). The 3/5-bromopyridine substrate (1.0-1.5 mmol, 1.0-1.5 equiv) and the alcohol substrate (1.0 mmol, 1.0 equiv) were measured into a separate vessel and constituted in DMAc (2.0 mL). This substrate solution was then sparged with N_2 for 3 min and then transferred into the reaction flask via a syringe under N_2. The reaction solution was then placed into a preheated 80 °C silicon oil bath and stirred for 15 h. The solution was then allowed to cool to rt, and 1,3,5-trimethoxybenzene internal standard was measured into the solution. ^{1}H NMR spectroscopy of the crude reaction solution was used to determine the selectivity of the reaction (see discussion below). The solution was then poured into a 250 mL separatory funnel containing water (60 mL). The product was extracted with ethyl acetate (3 x 40 mL) and the organic layers were dried over Na_2SO_4. The Na_2SO_4 was filtered off and the organic layer was concentrated *in vacuo*. All substrates were purified by silica gel chromatography using the given conditions. **Note**: in some cases, residual DMAc co-eluted with the desired product; this can be removed by extended drying *in vacuo*. The results below are reported in the format: (mass of isolated product, percent isolated yield, 4:3 substitution selectivity of crude reaction mixture).

Determining the selectivity of the reaction: The selectivity of each reaction was determined by analyzing the crude reaction solutions by ^{1}H NMR spectroscopy and determining the yield of both 4- and 3-isomeric products. If the identity of the 3-isomeric product was ambiguous in the ^{1}H NMR spectrum, an authentic sample was synthesized, or the 3-isomeric product was purified from the reaction mixture to ensure the correct peaks were used for the analysis. Below are examples of our process to determine the selectivity of the reaction. The top portion of the figure is an overlay of the authentic 3-substituted and 4-substituted isomers. The bottom portion of the figure shows the ^{1}H NMR spectrum of the crude reaction solution and the yield of both the 4-substituted isomer and the 3-substituted isomer relative to 1,3,5-trimethoxybenzene (TMB) internal standard.

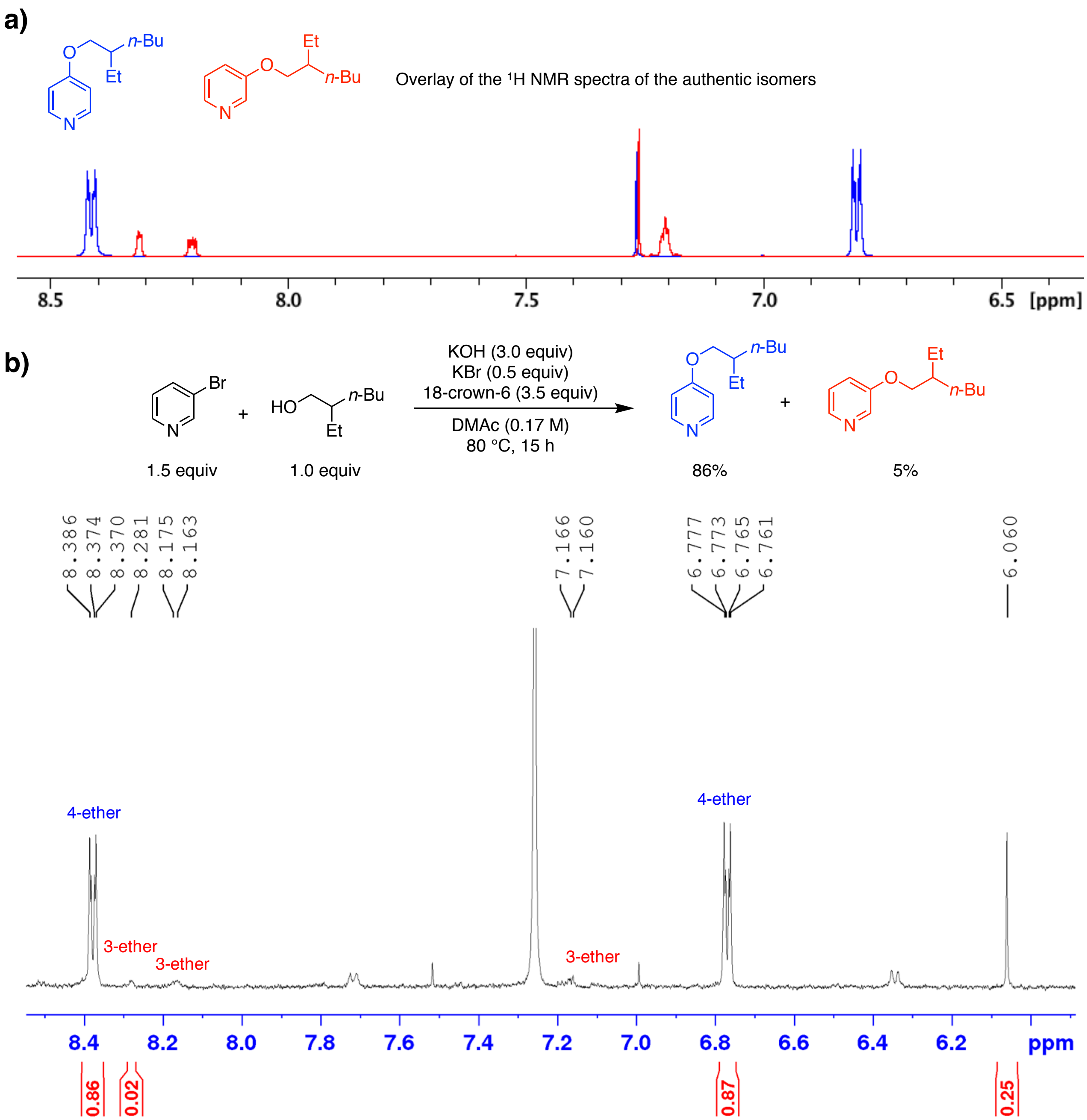

Figure S18: (**a**) Overlay of the ^{1}H NMR spectra of 4-((2-ethylhexyl)oxy)pyridine (blue) and 3-((2-ethylhexyl)oxy)pyridine (red). (**b**) ^{1}H NMR spectrum of the crude reaction solution of **10** used to determine the 4-selectivity. The reaction was run according to the general procedure; 27.5 mg (0.164 mmol) of TMB was added as internal standard: 86% yield of 4-((2-ethylhexyl)oxy)pyridine and 5% yield of 3-((2-ethylhexyl)oxy)pyridine (>10:1 selectivity).

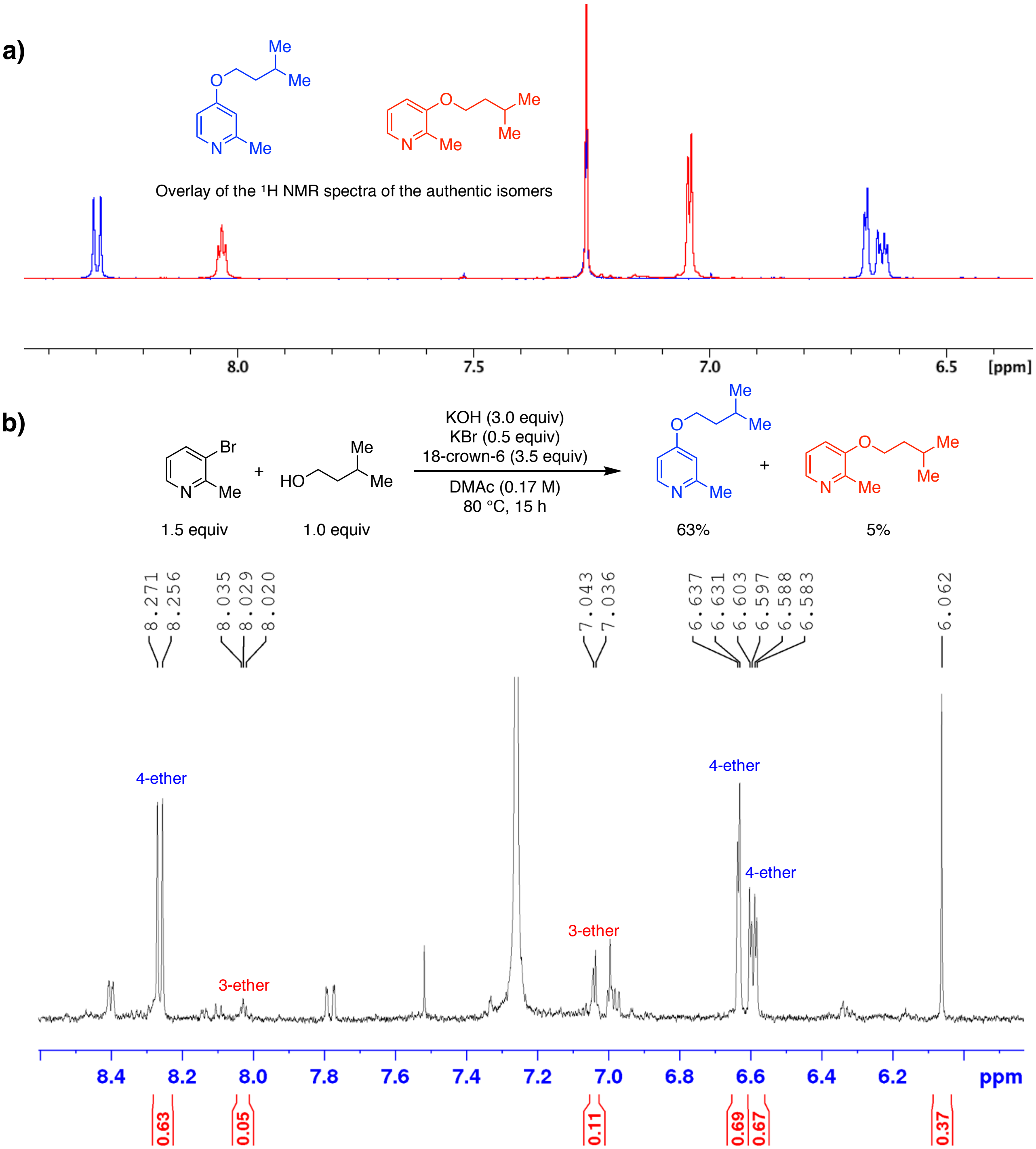

Figure S19: (**a**) Overlay of the ^{1}H NMR spectra of 4-(isopentyloxy)-2-methylpyridine (blue) and 3-(isopentyloxy)-2-methylpyridine (red). (**b**) ^{1}H NMR spectrum of the crude reaction solution of **20** used to determine the 4-selectivity. Reaction run according to the general procedure; 20.8 mg (0.124 mmol) of TMB was added as internal standard: 63% yield of 4-(isopentyloxy)-2-methylpyridine and 5% yield of 3-(isopentyloxy)-2-methylpyridine (>10:1 selectivity).

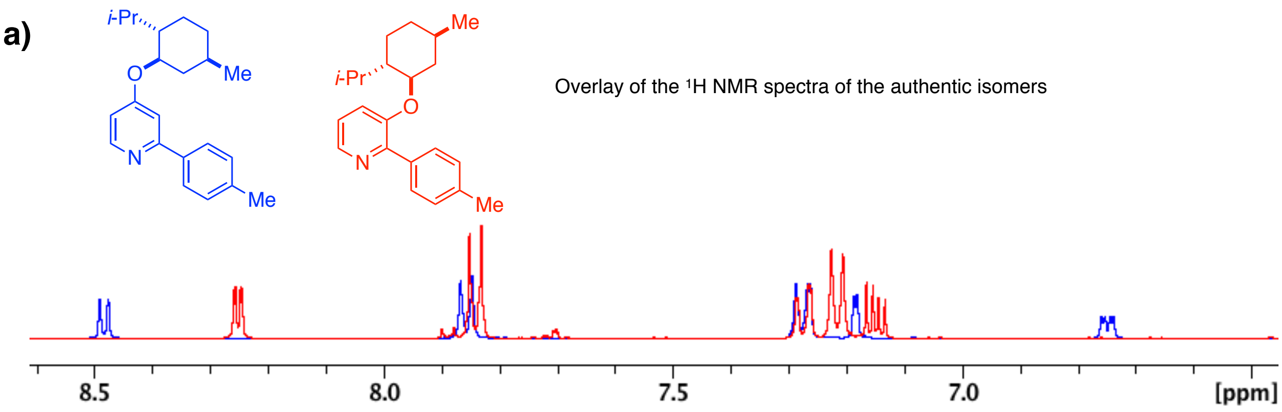

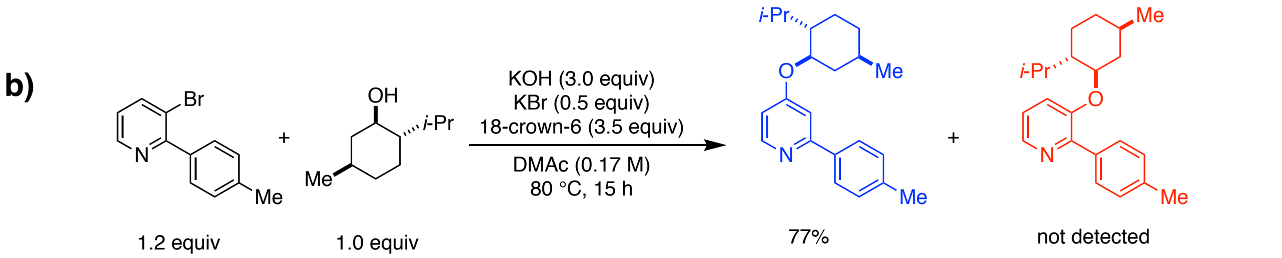

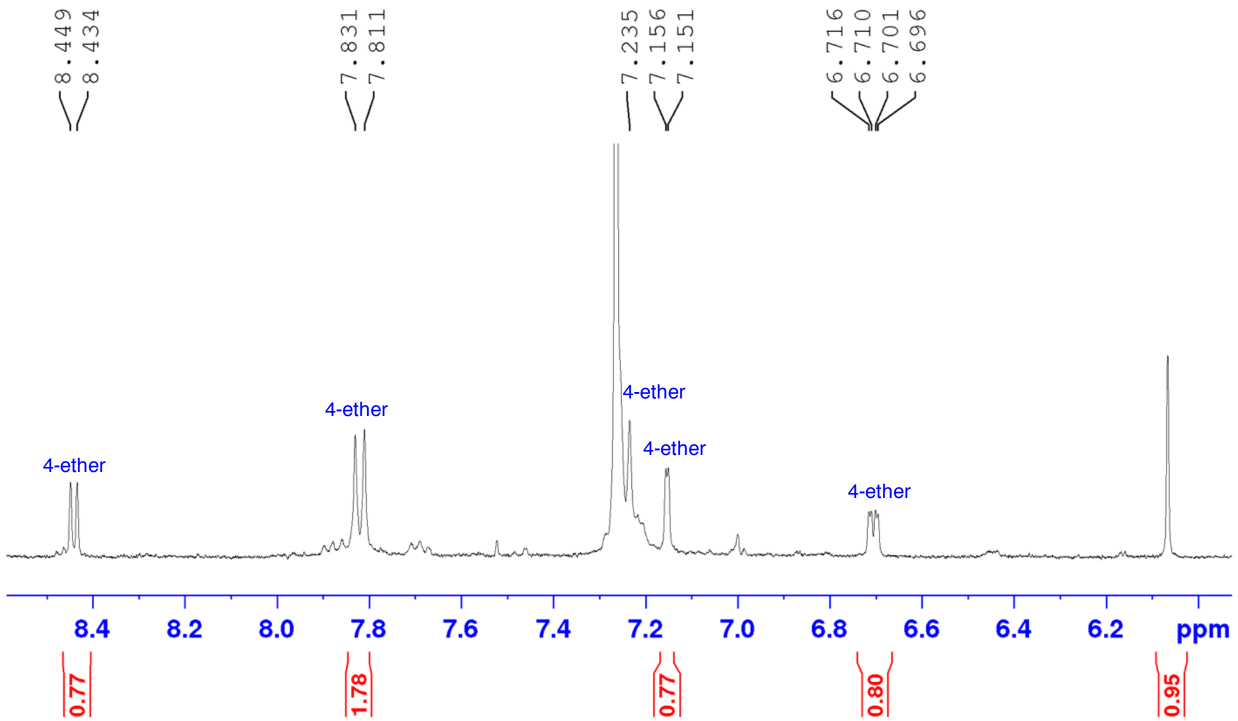

Figure S20: **(a)** Overlay of the ¹H NMR spectra of authentic 4-(((1*R*,2*S*,4*S*)-2-isopropyl-4-methylcyclohexyl)oxy)-2-(*p*-tolyl)pyridine (blue) and 3-(((1*R*,2*S*,4*S*)-2-isopropyl-4-methylcyclohexyl)oxy)-2-(*p*-tolyl)pyridine (red). **(b)** ¹H NMR spectrum of the crude reaction solution of **26**. Reaction run according to the general procedure on a 0.5 mmol scale; 26.7 mg (0.159 mmol) of TMB was added as internal standard: 77% yield of 4-(((1*R*,2*S*,4*S*)-2-isopropyl-

4-methylcyclohexyl)oxy)-2-(*p*-tolyl)pyridine and no observed yield of 3-(((1*R*,2*S*,4*S*)-2-isopropyl-4-methylcyclohexyl)oxy)-2-(*p*-tolyl)pyridine (>10:1 selectivity).

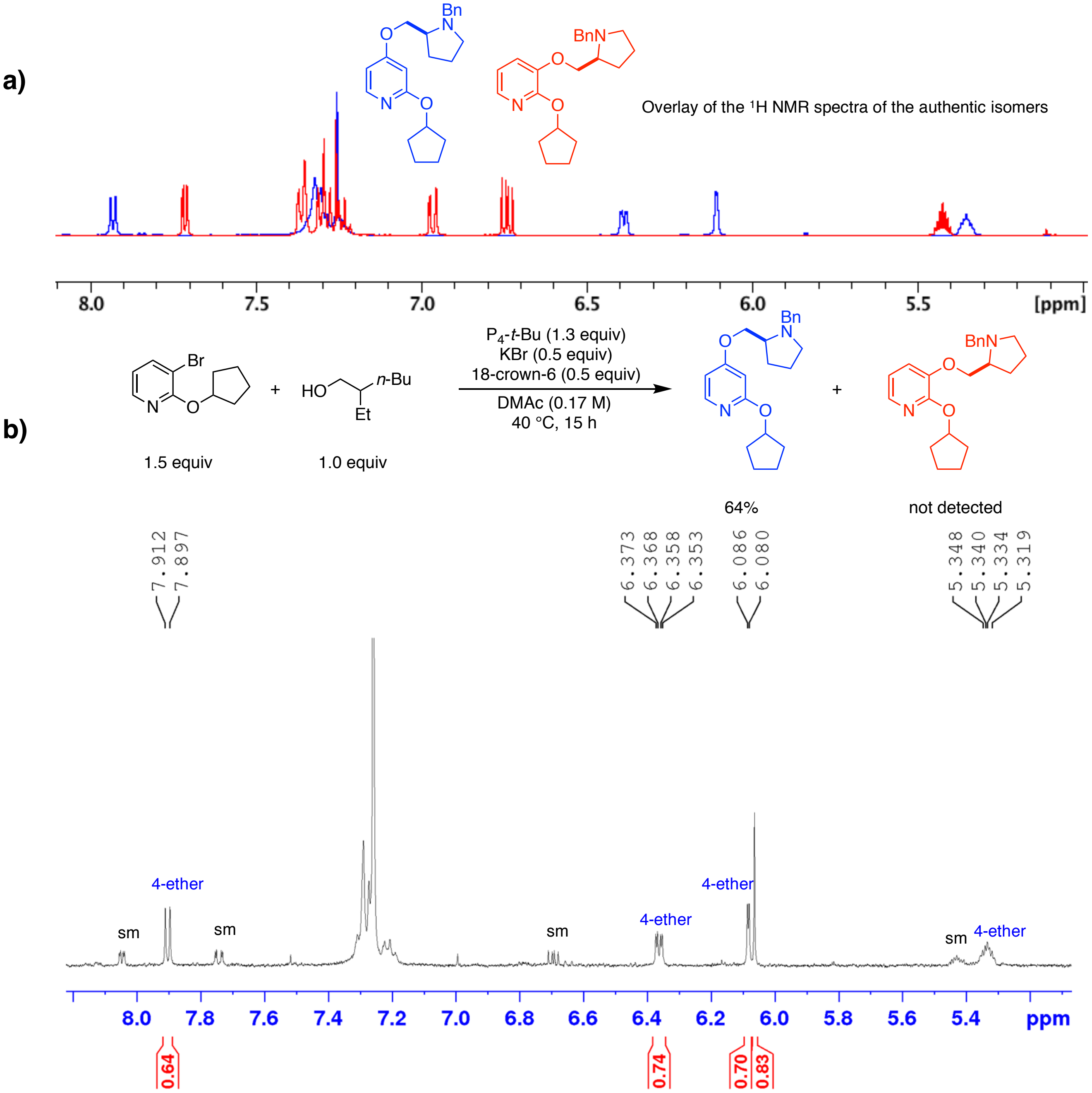

Figure S21: **(a)** Overlay of the ^{1}H NMR spectra of authentic (*S*)-4-((1-benzylpyrrolidin-2-yl)methoxy)-2-(cyclopentyloxy)pyridine (blue) and (*S*)-3-((1-benzylpyrrolidin-2-yl)methoxy)-2-(cyclopentyloxy)pyridine (red). **(b)** ^{1}H NMR spectrum of the crude reaction solution of **23**. Reaction run according to a modified procedure on a 0.25 mmol scale (see Section VI); 11.7 mg (0.070 mmol) of TMB was added as internal standard: 64% yield of (*S*)-4-((1-benzylpyrrolidin-2-

yl)methoxy)-2-(cyclopentyloxy)pyridine and no detected yield of (*S*)-3-((1-benzylpyrrolidin-2-yl)methoxy)-2-(cyclopentyloxy)pyridine (>10:1 selectivity); sm = starting material.

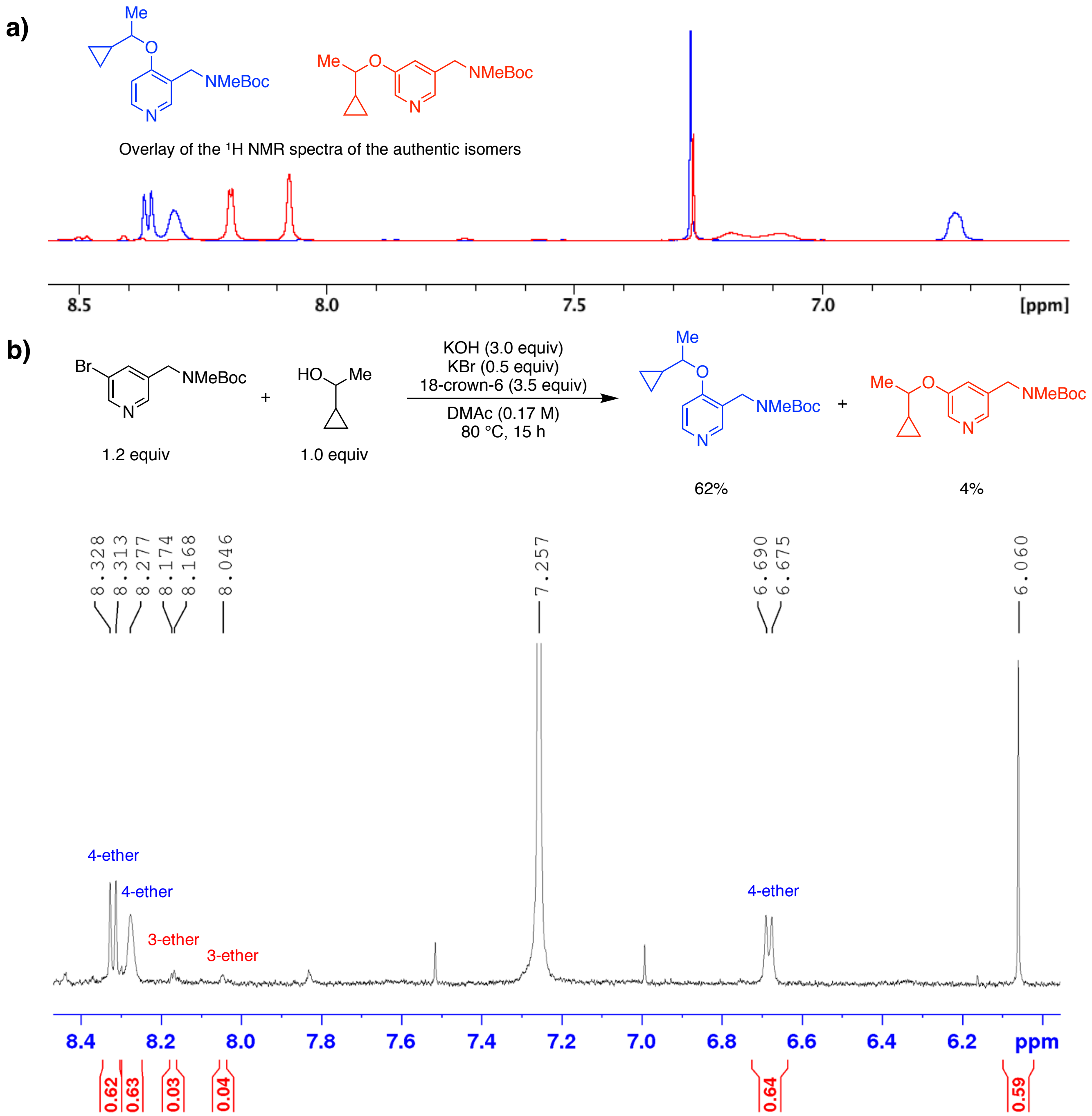

Figure S22: **(a)** Overlay of the ^{1}H NMR spectra of authentic *tert*-butyl ((4-(1-cyclopropylethoxy)pyridin-3-yl)methyl)(methyl)carbamate (blue) and *tert*-butyl ((5-(1-cyclopropylethoxy)pyridin-3-yl)methyl)(methyl)carbamate (red, broad signals due to rotamers). **(b)** ^{1}H NMR spectrum of the crude reaction solution of **29**. Reaction run according to the general procedure; 33.3 mg (0.198 mmol) of TMB was added as internal standard: 62% yield of *tert*-butyl ((4-(1-cyclopropylethoxy)pyridin-3-yl)methyl)(methyl)carbamate (blue) and 4% yield of *tert*-butyl ((5-(1-cyclopropylethoxy)pyridin-3-yl)methyl)(methyl)carbamate (>10:1 selectivity).

a)

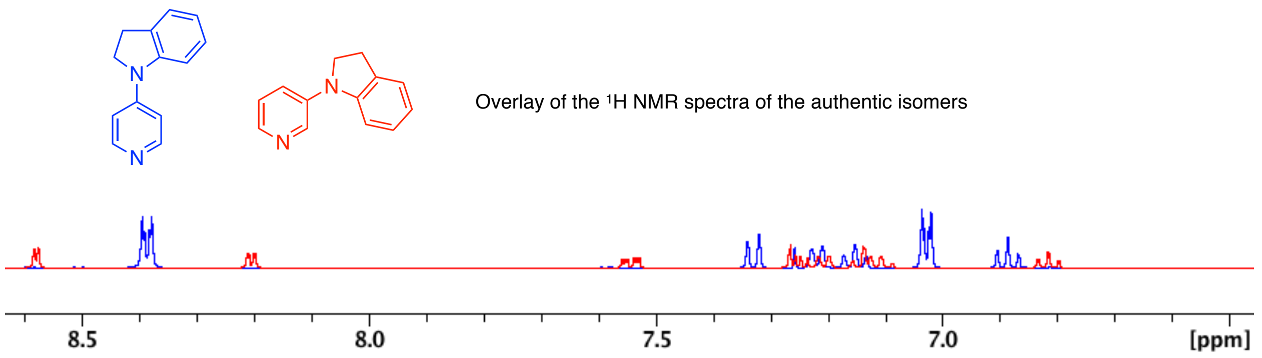

b)

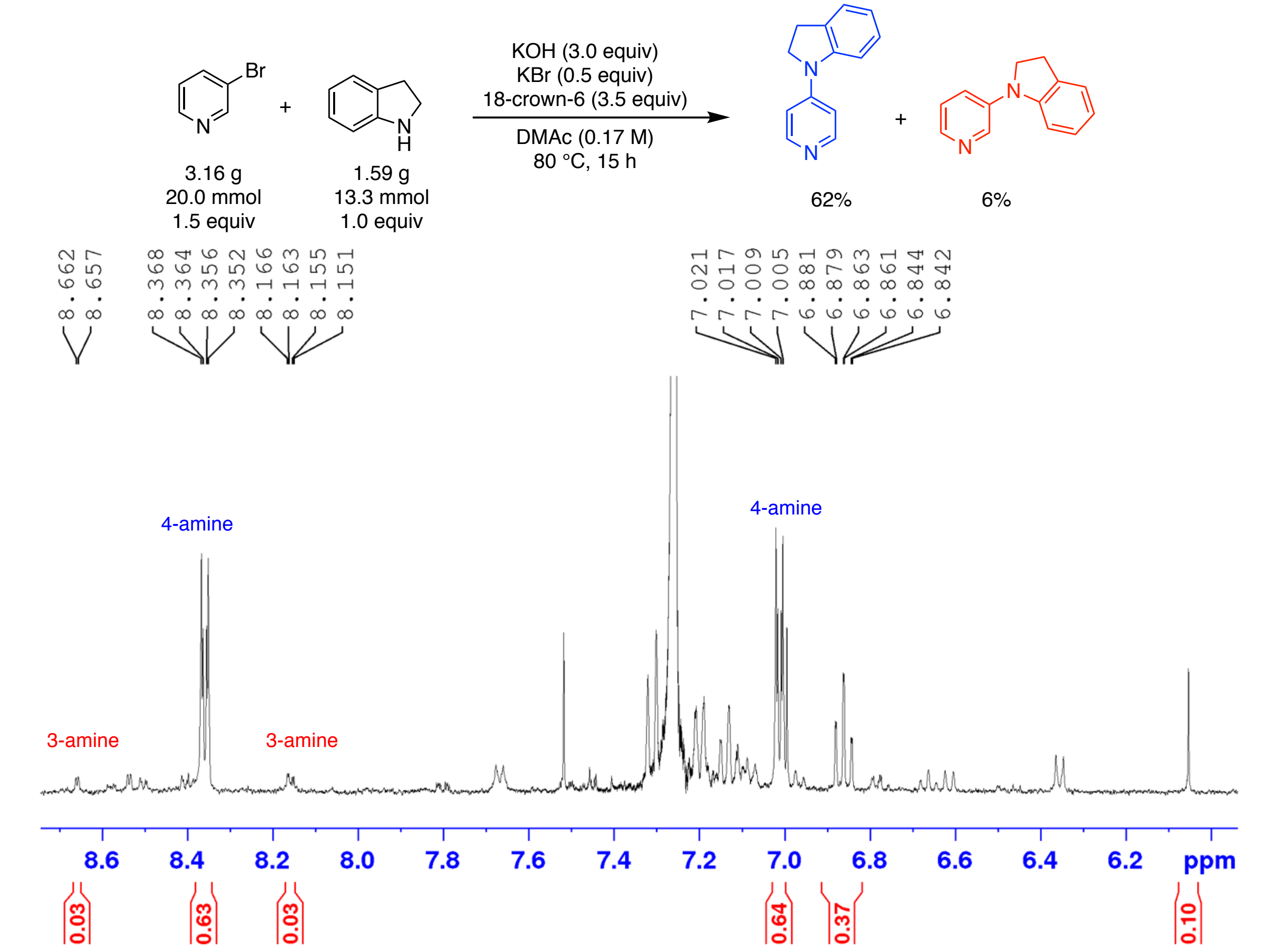

Figure S23: **(a)** Overlay of the ¹H NMR spectra of authentic 1-(pyridin-4-yl)indoline (blue) and 1-(pyridin-3-yl)indoline (red). **(b)** ¹H NMR spectrum of the crude reaction solution of **37**. Reaction run according to the procedure described in Section VIII on a 13.3 mmol scale; 139.5 mg (0.829 mmol) of TMB was added as internal standard. 62% yield of 1-(pyridin-4-yl)indoline (blue) and 6% yield of 1-(pyridin-3-yl)indoline (>10:1 selectivity).

VI. Characterization Data of 4-Substituted Pyridines

4-((2-ethylhexyl)oxy)pyridine (10)

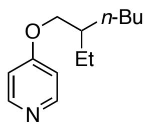

The title product was prepared according to the general procedure using 3-bromopyridine (237.0 mg, 1.5 mmol, 1.5 equiv) and 2-ethyl-1-hexanol (130.2 mg, 1.0 mmol, 1.0 equiv). Silica gel chromatography (50% EtOAc/hexanes) yielded the title compound as a light yellow oil (173.7 mg, 84% yield, >10:1 selectivity). **^{1}H NMR** (400 MHz, $CDCl_3$) δ 8.41 (d, J = 6.2 Hz, 2H), δ 6.80 (d, J = 6.2 Hz, 2H), 3.89 (m, 2H), 1.74 (m, 1H), 1.25-1.55 (m, 8H), 0.89-0.94 (m, 6H). **^{13}C NMR** (101 MHz, $CDCl_3$) δ 165.7, 151.3, 110.7, 70.7, 39.5, 30.8, 29.4, 24.1, 23.3, 14.4, 11.4. **HRMS** (DART) $[M+H]^+$ calcd. for $[C_{13}H_{21}NO]^+$ 208.1696, 208.1709 found. **IR** (neat, cm^{-1}) 2958, 2928, 1590, 1500, 1283, 1210, 1024.

4-((adamantan-1-yl)methoxy)pyridine (16)

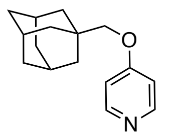

The title product was prepared according to the general procedure using 3-bromopyridine (237.0 mg, 1.5 mmol, 1.5 equiv) and 1-adamantanemethanol (166.3 mg, 1.0 mmol, 1.0 equiv). Silica gel chromatography (40% EtOAc/hexanes) yielded the title compound as an off-white powder (204.3 mg, 84% yield, >10:1 selectivity). **Melting Point**: 75 °C – 77 °C. **^{1}H NMR** (400 MHz, $CDCl_3$) δ 8.39 (m, 2H), 6.79 (m, 2H), 3.53 (s, 2H), 2.02 (m, 3H), 1.64-1.77 (m, 12H). **^{13}C NMR** (101 MHz, $CDCl_3$) δ 165.9, 151.2, 110.7, 78.3, 39.7, 37.4, 34.0, 28.4. **HRMS** (DART) $[M+H]^+$ calcd. for $[C_{16}H_{22}NO]^+$ 244.1696, 244.1703 found. **IR** (neat, cm^{-1}) 2900, 2848, 1590, 1457, 1283, 1212, 1019.

(*S*)-*N*,*N*-dibenzyl-1-phenyl-3-(pyridin-4-yloxy)propan-2-amine (17)

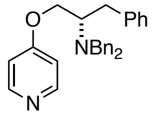

The title product was prepared according to the general procedure using 3-bromopyridine (237.0 mg, 1.5 mmol, 1.5 equiv) and (*S*)-2-(dibenzylamino)-3-phenylpropan-1-ol (331.5 mg, 1.0 mmol, 1.0 equiv). Silica gel chromatography (50% EtOAc/hexanes) yielded the title compound as a viscous yellow oil (346.8 mg, 85% yield, approximately 7:1 selectivity). **^{1}H NMR** (400 MHz, $CDCl_3$) δ 8.36 (m, 2H), 7.17-7.28 (m, 13H), 7.05 (m, 2H), 6.68 (m, 2H), 4.08 (dd, J = 6.3 Hz, 10.0 Hz, 1H), 4.00 (dd, J = 4.4 Hz, 10.0 Hz, 1H), 3.76-3.84 (m, 4H), 3.33 (m, 1H), 3.07 (dd, J = 6.2 Hz, 13.7 Hz, 1H), 2.85 (dd, J = 8.5 Hz, 13.7 Hz, 1H). **^{13}C NMR** (101 MHz, $CDCl_3$) δ 165.1, 151.2, 140.1, 139.7, 129.6, 128.9, 128.8, 128.6, 127.3, 126.6, 110.6, 67.9, 58.5, 54.8, 34.4. **HRMS** (DART) $[M+H]^+$ calcd. for $[C_{28}H_{29}N_2O]^+$ 409.2274, 409.2304 found. **IR** (neat, cm^{-1}) 3026, 2801, 1590, 1494, 1453, 1282, 1026. **Specific Rotation**: $[\alpha]_D^{23}$ = -44.3°.

4-(pent-4-en-1-yloxy)pyridine (18)

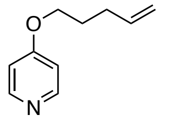

The title product was prepared according to the general procedure using 3-bromopyridine (237.0 mg, 1.5 mmol, 1.5 equiv) and 4-penten-1-ol (86.1 mg, 1.0 mmol, 1.0 equiv). Silica gel chromatography (45% to 50% EtOAc/hexanes) yielded the title compound as a light yellow oil (99.0 mg, 61% yield, >10:1 selectivity). **^{1}H NMR** (400 MHz, $CDCl_3$) δ 8.41 (m, 2H), 6.79 (m, 2H), 5.83 (m, 1H), 5.00-5.08 (m, 2H), 4.01 (t, J = 6.4 Hz, 2H), 2.24 (m, 2H), 1.90 (m, 2H). **^{13}C NMR** (101 MHz, $CDCl_3$) δ 165.3, 151.3, 137.7, 115.9, 110.6, 67.3, 30.2, 28.3. **HRMS** (DART) $[M+H]^+$ calcd. for $[C_{10}H_{14}NO]^+$ 164.1070, 164.1073 found. **IR** (neat, cm^{-1}) 2942, 1591, 1501, 1283, 1210, 1010.

4-(decyloxy)-3-methylpyridine (19)

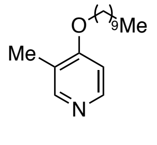

The title product was prepared according to the general procedure using 3-bromo-5-methylpyridine (258.0 mg, 1.5 mmol, 1.5 equiv) and decan-1-ol (158.3 mg, 1.0 mmol, 1.0 equiv). Silica gel chromatography (50% EtOAc/hexanes) yielded the title compound as a light yellow oil (174.6 mg, 70% yield, >10:1 selectivity). **^{1}H NMR** (400 MHz, $CDCl_3$) δ 8.31 (d, J = 5.6 Hz, 1H), 8.24 (s, 1H), 6.70 (d, J = 5.6 Hz, 1H), 4.00 (t, J = 6.5 Hz, 2H), 2.17 (s, 3H), 1.82 (m, 2H), 1.47 (m, 2H), 1.22-1.40 (m, 12H), 0.88 (t, J = 7.0 Hz, 3H). **^{13}C NMR** (101 MHz, $CDCl_3$) δ 163.7, 151.0, 149.4, 122.7, 106.5, 68.2, 32.2, 29.9, 29.9, 29.6, 29.3, 26.3, 23.0, 14.4, 13.4. **HRMS** (DART) $[M+H]^+$ calcd. for $[C_{16}H_{28}NO]^+$ 250.2165, 250.2173 found. **IR** (neat, cm^{-1}) 2923, 2854, 1589, 1501, 1467, 1283, 1186.

4-(isopentyloxy)-2-methylpyridine (20)

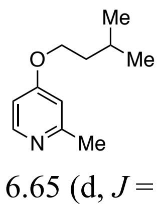

The title product was prepared according to the general procedure using 3-bromo-2-methylpyridine (258.0 mg, 1.5 mmol, 1.5 equiv) and isoamyl alcohol (88.2 mg, 1.0 mmol, 1.0 equiv). Silica gel chromatography (40% EtOAc/hexanes to 50% EtOAc/hexanes) yielded the title compound as a light yellow oil (111.5 mg, 62% yield, >10:1 selectivity). **^{1}H NMR** (400 MHz, $CDCl_3$) δ 8.29 (d, J = 5.8 Hz, 1H), 6.65 (d, J = 2.4 Hz, 1H), 6.62 (dd, J = 2.4 Hz, 5.8 Hz, 1H), 4.01 (t, J = 6.6 Hz, 2H), 2.50 (s, 3H), 1.81 (m, 1H), 1.67 (m, 2H), 0.96 (d, J = 6.6 Hz, 6H). **^{13}C NMR** (101 MHz, $CDCl_3$) δ 165.8, 160.2, 150.5, 109.7, 107.9, 66.5, 37.9, 25.3, 24.9, 22.8. **HRMS** (DART) $[M+H]^+$ calcd. for $[C_{11}H_{18}NO]^+$ 180.1383, 180.1390 found. **IR** (neat, cm^{-1}) 2956, 1595, 1568, 1474, 1284, 1023.

4-(dicyclopropylmethoxy)-2-methylpyridine (21)

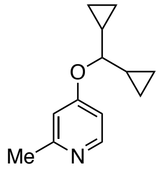

The title product was prepared according to the general procedure using 5-bromo-2-methylpyridine (258.0 mg, 1.5 mmol, 1.5 equiv) and dicyclopropylmethanol (112.2 mg, 1.0 mmol, 1.0 equiv). Silica gel chromatography (50% EtOAc/hexanes) yielded the title compound as a light yellow oil (153.9 mg, 76% yield, >10:1 selectivity). **^{1}H NMR** (400 MHz, $CDCl_3$) δ 8.26 (d, J = 5.8 Hz, 1H), 6.65 (d, J = 2.4 Hz, 1H), 6.60 (dd, J = 2.4 Hz, 5.8 Hz, 1H), 3.66 (t, J = 6.8 Hz, 1H), 2.47 (s, 3H), 1.14 (m, 2H), 0.50-0.58 (m, 4H), 0.32-0.46 (m, 4H). **^{13}C NMR** (101 MHz, $CDCl_3$) δ 165.8, 160.2, 150.5, 111.0, 108.9, 83.5, 24.9, 14.7, 2.8, 2.6. **HRMS** (DART) $[M+H]^+$ calcd. for $[C_{13}H_{18}NO]^+$ 204.1383, 204.1398 found. **IR** (neat, cm^{-1}) 3084, 3008, 1593, 1564, 1281, 1172, 972.

4-(((3a*R*,5*R*,5a*S*,8a*S*,8b*R*)-2,2,7,7-tetramethyltetrahydro-5*H*-bis([1,3]dioxolo)[4,5-*b*:4',5'-*d*]pyran-5-yl)methoxy)pyridine (22)

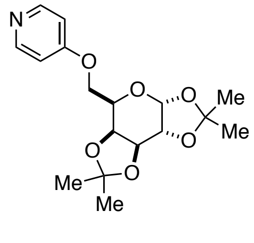

The title product was prepared according to the general procedure on a 0.5 mmol scale using 3-bromopyridine (118.5 mg, 0.75 mmol, 1.5 equiv) and ((3a*R*,5*R*,5a*S*,8a*S*,8b*R*)-2,2,7,7-tetramethyltetrahydro-5*H*-bis([1,3]dioxolo)[4,5-*b*:4',5'-*d*]pyran-5-yl)methanol (130.2 mg, 0.5 mmol, 1.0 equiv). Silica gel chromatography (60% EtOAc/hexanes) yielded the title compound as a white solid (87.5 mg, 52% yield, >10:1 selectivity). **Melting Point**: 111 °C – 114 °C. **^{1}H NMR** (400 MHz, $CDCl_3$) δ 8.43 (d, J = 6.3 Hz, 2H), 6.88 (d, J = 6.3 Hz, 2H), 5.56 (d, J = 5.0 Hz, 1H), 4.66 (dd, J = 2.5 Hz, 8.0 Hz, 1H), 4.33-4.36 (m, 2H), 4.15-4.25 (m, 3H), 1.53 (s, 3H), 1.47 (s, 3H), 1.35 (s, 3H), 1.34 (s, 3H). **^{13}C NMR** (101 MHz, $CDCl_3$) δ 165.4, 150.9, 110.9, 110.0, 109.2, 96.7, 71.2, 71.0, 70.9, 67.1, 66.4, 26.4,

26.3, 25.2, 24.8. **HRMS** (DART) $[M+H]^+$ calcd. for $[C_{17}H_{24}NO_6]^+$ 338.1598, 338.1620 found. **IR** (neat, cm^{-1}) 2938, 2905, 1598, 1381, 1283, 1115, 999. **Specific Rotation**: $[\alpha]_D^{23}$ = -52.5°.

(*S*)-4-((1-benzylpyrrolidin-2-yl)methoxy)-2-(cyclopentyloxy)pyridine (23)

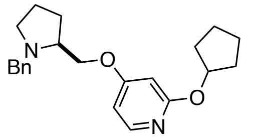

The title product was prepared using a modified procedure. An oven-dried 25 mL round bottom flask was charged with a magnetic stir bar, 3-bromo-2-(cyclopentyloxy)pyridine (90.8 mg, 0.375 mmol, 1.5 equiv), and (*S*)-(1-benzylpyrrolidin-2-yl)methanol (47.8 mg, 0.25 mmol, 1.0 equiv). The flask was then brought into a N_2 filled glovebox and charged with DMAc (1.5 mL, 0.17 M), KBr (14.9 mg, 0.125 mmol, 0.5 equiv), 18-crown-6 (33.0 mg, 0.125 mmol, 0.5 equiv), and P_4-*t*-Bu (407 µl of a 0.8 M solution in hexanes, 0.325 mmol, 1.3 equiv) in that respective order. The flask was then sealed with a Fisherbrand® red septum stopper (Cat. No. FB57875) and the edges of the septum were then sealed with Parafilm®. The flask was then removed from the glovebox, placed into a preheated 40 °C silicon oil bath and stirred for 15 h. The aqueous workup and purification procedures are in accord with the general procedure. Silica gel chromatography (35% EtOAc/hexanes) yielded the title compound as a brown oil (52.4 mg, 60% yield, >10:1 selectivity). **^{1}H NMR** (400 MHz, $CDCl_3$) δ 7.93 (d, J = 5.9 Hz, 1H), 7.28-7.38 (m, 4H), 7.25 (m, 1H), 6.39 (dd, J = 1.8 Hz, 5.9 Hz, 1H), 6.11 (d, J = 1.8 Hz, 1H), 5.35 (m, 1H), 4.08 (m, 1H), 3.93 (m, 1H), 3.83 (m, 1H), 3.54 (m, 1H), 2.92-3.08 (m, 2H), 2.32 (m, 1H), 1.86-2.07 (m, 3H), 1.68-1.85 (m, 7H), 1.56-1.66 (m, 2H). **^{13}C NMR** (101 MHz, $CDCl_3$) δ 167.4, 165.9, 147.9, 129.3, 128.6, 127.4, 106.2, 95.4, 78.2, 71.4, 62.3, 60.0, 54.9, 33.2, 28.9, 24.2, 23.3. **HRMS** (DART) $[M+H]^+$ calcd. for $[C_{22}H_{29}N_2O_2]^+$ 353.2224, 353.2237 found. **IR** (neat, cm^{-1}) 2959, 2871, 1597, 1419, 1165, 1028. **Specific Rotation**: $[\alpha]_D^{23}$ = +9.6°.

2-chloro-10-(3-(4-(2-((2-methylpyridin-4-yl)oxy)ethyl)piperazin-1-yl)propyl)-10*H*-phenothiazine (24)

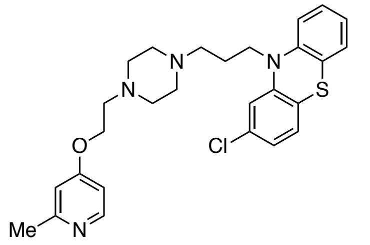

The title product was synthesized according to the general procedure on a 0.5 mmol scale using 5-bromo-2-methylpyridine (129.0 mg, 0.75 mmol, 1.5 equiv) and perphenazine (202.0 mg, 0.50 mmol, 1.0 equiv). The crude organic residue obtained after the aqueous workup was dried *in vacuo* for 24 h. The desired product was purified via chromatography on NEt_3-neutralized silica gel (1% $MeOH/CH_2Cl_2$ to 1.5% $MeOH/CH_2Cl_2$). A small quantity of triethylammonium salt was present in the product after purification. This was removed by diluting the product in EtOAc (50 mL) and washing with H_2O (2 x 50 mL) and with brine (1 x 50 mL). The organic layer was subsequently dried over Na_2SO_4 and concentrated *in vacuo* to afford the purified product as a light yellow oil (130.6 mg, 53% yield, >10:1 selectivity). **^{1}H NMR** (400 MHz, $CDCl_3$) δ 8.29 (d, J = 5.8 Hz, 1H), 7.10-7.16 (m, 2H), 7.01 (d, J = 8.1 Hz, 1H), 6.84-6.94 (m, 4H), 6.66 (d, J = 2.4 Hz, 1H), 6.62 (dd, J = 2.4 Hz, 5.8 Hz, 1H), 4.10 (t, J = 5.8 Hz, 2H), 3.90 (t, J = 6.8 Hz, 2H), 2.79 (t, J = 5.8 Hz, 2H), 2.52-2.67 (m, 4H), 2.37-2.52 (m, 9H), 1.94 (m, 2H). **^{13}C NMR** (101 MHz, $CDCl_3$) δ 165.4, 160.3, 150.6, 146.8, 144.8, 133.5, 128.2, 127.8, 127.7, 125.1,123.8, 123.2, 122.6, 116.2, 116.1, 109.8, 108.0, 66.0, 57.1, 55.8, 53.9, 53.5, 45.6, 24.9, 24.5. **HRMS** (DART) $[M+H]^+$ calcd. for $[C_{27}H_{32}ClN_4OS]^+$ 495.1980, 495.1984 found. **IR** (neat, cm^{-1}) 2939, 2811, 1594, 1566, 1456, 1282, 1039.

4-(4-(1-cyclopropylethoxy)pyridin-2-yl)-*N*,*N*-dimethylaniline (25)

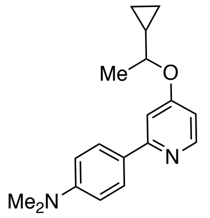

The title product was prepared according to the general procedure on a 0.5 mmol scale using 4-(5-bromopyridin-2-yl)-*N*,*N*-dimethylaniline (207.2 mg, 0.75 mmol, 1.5 equiv) and 1-cyclopropylethanol (43.1 mg, 0.5 mmol, 1.0 equiv). Silica gel chromatography (50% EtOAc/hexanes) yielded the title compound as a light yellow oil (77.0 mg, 55% yield, >10:1 selectivity). **^{1}H NMR** (400 MHz, $CDCl_3$) δ 8.43 (d, J = 5.9 Hz, 1H), 7.89 (d, J = 9.0 Hz, 2H), 7.13 (d, J = 2.4 Hz, 1H), 6.78 (d, J = 9.0 Hz, 2H), 6.64 (dd, J = 2.4 Hz, 5.9 Hz, 1H), 4.03 (m, 1H), 3.02 (s, 6H), 1.42 (d, J = 6.1 Hz, 3H), 1.17 (m, 1H), 0.61 (m, 2H), 0.44 (m, 1H), 0.33 (m, 1H). **^{13}C NMR** (101 MHz, $CDCl_3$) δ 165.4, 159.5, 151.4, 150.7, 128.1, 127.4, 112.5, 108.5, 107.0, 77.8, 40.7, 19.5, 16.9, 3.9, 2.5. **HRMS** (DART) $[M+H]^+$ calcd. for $[C_{18}H_{23}N_2O]^+$ 283.1805, 283.1811 found. **IR** (neat, cm^{-1}) 2976, 1894, 1587, 1526, 1468, 1192, 1064.

4-(((1*R*,2*S*,5*R*)-2-isopropyl-5-methylcyclohexyl)oxy)-2-(*p*-tolyl)pyridine (26)

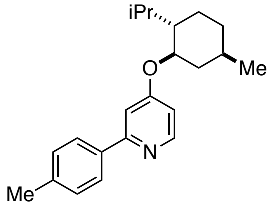

The title product was prepared according to the general procedure on a 0.5 mmol scale using 3-bromo-2-(*p*-tolyl)pyridine (248.1 mg, 0.75 mmol, 1.5 equiv) and (1*R*,2*S*,5*R*)-2-isopropyl-5-methylcyclohexan-1-ol (78.2 mg, 0.5 mmol, 1.0 equiv). Silica gel chromatography (10% EtOAc/hexanes) yielded the title compound as a white solid (124.3 mg, 77% yield, >10:1 selectivity). **Melting Point**: 201 °C – 205 °C **^{1}H NMR** (400 MHz, $CDCl_3$) δ 8.49 (d, J = 5.8 Hz, 1H), 7.86 (d, J = 8.2 Hz, 2H), 7.28 (d, J = 8.2 Hz, 2H), 7.18 (d, J = 3.0 Hz, 1H), 6.75 (dd, J = 3.0 Hz, 5.8 Hz, 1H), 4.21 (td, J = 4.2 Hz, 10.6 Hz, 1H), 2.40 (s, 3H), 2.14 (m, 2H), 1.76 (m, 2H), 1.47-1.60 (m, 2H), 1.03-1.19 (m, 2H), 0.90-1.00 (m, 7H), 0.77 (d, J = 7.0 Hz, 3H). **^{13}C NMR** (101 MHz, $CDCl_3$) δ 165.9, 159.3, 150.7, 139.5, 136.5, 129.8, 127.2, 109.0, 108.3, 77.8, 48.1, 40.2, 34.6, 31.7, 26.5, 24.1, 22.4, 21.6, 21.0, 17.0. **HRMS** (DART) $[M+H]^+$ calcd. for $[C_{22}H_{30}NO]^+$ 324.2322, 323.2325 found. **IR** (neat, cm^{-1}) 2924.45, 2602.04, 1594.40, 1473.69, 1397. 27, 1198.60. **Specific Rotation**: $[\alpha]_D^{23}$ = -2.9°.

4-(((3*S*,4*R*)-4-(4-fluorophenyl)-1-methylpiperidin-3-yl)methoxy)-2-methylpyridine (27)

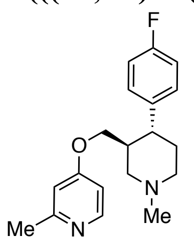

The title product was prepared according to the general procedure using 5-bromo-2-methylpyridine (206.4 mg, 1.2 mmol, 1.2 equiv) and (3*S*, 4*R*)-4-(4-fluorophenyl)-3-hydroxymethyl-1-methylpiperidine (331.5 mg, 1.0 mmol, 1.0 equiv). Chromatography on NEt_3-neutralized silica gel (100% CH_2Cl_2 – 5% $MeOH/CH_2Cl_2$) yielded the title compound as a light brown oil (172.4 mg, 55% yield, >10:1 selectivity). **^{1}H NMR** (400 MHz, $CDCl_3$) δ 8.22 (d, J = 5.7 Hz, 1H), 7.15 (m, 2H), 6.97 (m, 2H), 6.48 (d, J = 2.2 Hz, 1H), 6.44 (dd, J = 2.2 Hz, 5.7 Hz, 1H), 3.67 (dd, J = 2.9 Hz, 9.7 Hz, 1H), 3.55 (dd, J = 6.7 Hz, 9.7 Hz, 1H), 3.21 (m, 1H), 3.03 (m, 1H), 2.27-2.58 (m, 8H), 2.04-2.19 (m, 2H), 1.97 (m, 1H), 1.84 (m, 1H). **^{13}C NMR** (101 MHz, $CDCl_3$) δ 165.4, 162.0 (d, J = 245.8 Hz) , 160.3, 150.5, 139.3 (d, J = 3.1 Hz), 129.1 (d, J = 7.8 Hz), 115.9 (d, J = 21.2 Hz), 109.5, 107.8, 68.4, 59.4, 56.3, 46.5, 43.5, 41.9, 34.2, 24.8. **^{19}F NMR** (376 MHz, $CDCl_3$) δ 116.69. **HRMS** (DART) $[M+H]^+$ calcd. for $[C_{19}H_{24}FN_2O]^+$ 315.1867, 315.1876 found. **IR** (neat, cm^{-1}) 2938.27, 2795.74, 1598.89, 1509.84, 1283.33, 1221.45, 1177.06. **Specific Rotation**: $[\alpha]_D^{23}$ = -38.2°.

4-(4-(cyclopropylmethoxy)pyridin-2-yl)morpholine (28)

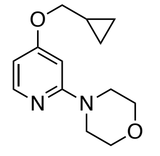

The title product was prepared according to the general procedure using 4-(3-bromopyridin-2-yl)morpholine (291.7 mg, 1.2 mmol, 1.2 equiv) and cyclopropylmethanol (72.1 mg, 1.0 mmol, 1.0 equiv). Silica gel chromatography (40% EtOAc/hexanes) yielded the title compound as a light yellow oil (122.1 mg, 43% yield, 10:1 selectivity). **^{1}H NMR** (400 MHz, $CDCl_3$) δ 8.03 (d, J = 5.8 Hz, 1H), 6.28 (dd, J = 2.6 Hz, 5.8 Hz, 1H), 6.10 (d, J = 2.6 Hz, 1H), 3.80-3.83 (m, 6H), 3.47 (t, J = 5.0 Hz, 4H), 1.26 (m, 1H), 0.66 (m, 2H), 0.35 (m, 2H). **^{13}C NMR** (101 MHz, $CDCl_3$) δ 167.0, 161.8, 149.3, 102.1, 92.8, 72.7, 67.0, 46.1, 10.3, 3.5. **HRMS** (DART) $[M+H]^+$ calcd. for $[C_{13}H_{19}N_2O_2]^+$ 235.1441, 235.1461 found. **IR** (neat, cm^{-1}) 2963, 2859, 1595, 1553, 1441, 1202, 1118.

***tert*-butyl ((4-(1-cyclopropylethoxy)pyridin-3-yl)methyl)(methyl)carbamate (29)**

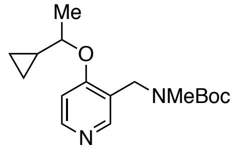

The title product was prepared according to the general procedure using *tert*-butyl ((5-bromopyridin-3-yl)methyl)(methyl)carbamate (343.2 mg, 1.2 mmol, 1.2 equiv) and 1-cyclopropylethanol (86.1 mg, 1.0 mmol, 1.0 equiv). Silica gel chromatography (60% EtOAc/hexanes) yielded the title compound as a light yellow oil (179.0 mg, 61% yield, >10:1 selectivity). **^{1}H NMR** (400 MHz, $CDCl_3$) signal broadening due to mixture of rotamers δ 8.34 (d, J = 5.8 Hz, 1H), 8.29 (br s, 1H), 6.70 (d, J = 5.8 Hz, 1H), 4.41 (m, 2H), 4.01 (m, 1H), 2.75-2.99 (m, 3H), 1.41-1.53 (m, 9H), 1.38 (m, 3H), 1.13 (m, 1H), 0.56 (m, 2H), 0.37 (m, 1H), 0.29 (m, 1H). **^{13}C NMR** (101 MHz, $CDCl_3$) (mixture of rotamers) δ 162.6, 156.2, 150.9, 150.5, 150.3, 123.1, 122.9, 107.7, 80.2, 79.9, 78.1, 77.8, 46.0, 45.2, 35.2, 34.7, 28.8, 19.6, 16.9, 3.8, 2.5. **HRMS** (DART) $[M+H]^+$ calcd. for $[C_{17}H_{27}N_2O_3]^+$ 307.2016, 307.2036 found. **IR** (neat, cm^{-1}) 2976, 1688, 1588, 1490, 1390, 1140, 1048.

2-(4-((2-ethylhexyl)oxy)pyridin-2-yl)-1-(pyrrolidin-1-yl)propan-1-one (30)

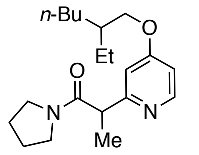

The title product was prepared using a modified procedure. A 4.0 mL oven-dried vial (ThermoFisher, C4015-1) was charged with a magnetic stir bar and 2-(5-bromopyridin-2-yl)-1-(pyrrolidin-1-yl)propan-1-one (84.6 mg, 0.3 mmol, 1.2 equiv). The vial was then brought into a N_2 filled glovebox and charged with 2-ethyl-1-hexanol (32.6 mg, 0.25 mmol, 1.0 equiv), DMAc (1.5 mL, 0.17 M), KBr (14.9 mg, 0.125 mmol, 0.5 equiv), 18-crown-6 (231.3 mg, 0.875 mmol, 3.5 equiv), and KOH (42.1 mg, 0.75 mmol, 3.0 equiv) in that respective order. The vial was sealed with a PTFE-lined screw cap (ThermoFisher, C4015-1A), removed from the glovebox, and placed into a preheated 80 °C aluminum reaction block. The reaction solution was stirred at 80 °C for 15 h. The solution was then allowed to cool to rt and was quenched with H_2O (ca. 1.0 mL) and diluted with EtOAc (30 mL). The organic layer was washed with H_2O (2 x 30 mL) and brine (1 x 20 mL). The organic layer was then dried over Na_2SO_4 and concentrated *in vacuo*. The crude residue was then loaded onto a preparatory TLC plate and developed with 1% MeOH/CH_2Cl_2 (R_f = 0.7). The band was scrapped off of the plate and the silica was washed with 100 mL 5% MeOH/CH_2Cl_2 to extract the product. The volatiles were removed *in vacuo*. The product remained impure and was therefore loaded onto another preparatory TLC plate neutralized with NEt_3 and developed with 50% EtOAc/hexanes (R_f = 0.4). The band was scrapped off the plate and the silica gel was washed with 100 mL of 5% MeOH/CH_2Cl_2. The volatiles were removed *in vacuo* to yield the title compound as a clear oil (32.0 mg, 44% yield, approximately 9:1 selectivity). **^{1}H NMR** (400 MHz, $CDCl_3$) δ 8.27 (d, J = 5.8 Hz, 1H), 6.89 (d, J = 2.4 Hz, 1H), 6.65 (dd, J = 2.4 Hz, 5.8 Hz, 1H), 3.99 (q, J =

7.0 Hz, 1H), 3.85 (m, 2H), 3.51 (m, 2H), 3.39 (m, 2H), 1.90 (m, 1H), 1.75-1.84 (m, 3H), 1.69 (m, 1H), 1.48 (d, *J* = 7.0 Hz, 3H), 1.34-1.45 (m, 3H), 1.21-1.34 (m, 5H), 0.81-0.95 (m, 6H). **^{13}C NMR** (101 MHz, $CDCl_3$) *δ* 171.8, 166.6, 163.4, 150.2, 109.4, 107.7, 70.7, 47.8, 46.9, 46.3, 39.5, 39.5, 30.7, 30.7, 29.4, 26.3, 24.6, 24.0, 24.0, 23.3, 18.9, 14.4, 11.4 (Note: chemical shifts characteristic of diastereomers can be observed in the aliphatic region of the ^{13}C NMR spectrum for this product as racemic alcohol and pyridine substrates were used in its synthesis). **HRMS** (DART) $[M+H]^+$ calcd. for $[C_{20}H_{33}N_2O_2]^+$ 333.2537, 333.2539 found. **IR** (neat, cm^{-1}) 2929, 2872, 1640, 2592, 1430, 1301, 1172.

2-(2,6-dimethylphenyl)-6-ethyl-4-(nonyloxy)pyridine (31)

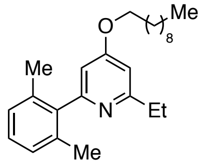

The title product was prepared according to the general procedure inside an N_2 filled glovebox. A 4.0 mL oven-dried vial (ThermoFisher, C4015-1) was charged with a magnetic stir bar and 3-bromo-6-(2,6-dimethylphenyl)-2-ethylpyridine (72.3 mg, 0.25 mmol, 1.0 equiv). The vial was then brought into a N_2 filled glovebox and charged with 1-decanol (39.6 mg, 0.25 mmol, 1.0 equiv), DMAc (1.5 mL, 0.17 M), KBr (14.9 mg, 0.125 mmol, 0.5 equiv), 18-crown-6 (231.3 mg, 0.875 mmol, 3.5 equiv), and KOH (42.1 mg, 0.75 mmol, 3.0 equiv) in that respective order. The vial was sealed with a PTFE-lined screw cap (ThermoFisher, C4015-1A), removed from the glovebox, and placed into a preheated 80 °C aluminum reaction block and stirred for 15 h. The solution was allowed to cool to rt and then loaded directly onto a silica gel column and eluted (1% acetone/hexanes to 2% acetone/hexanes) to yield the purified title compound as a clear oil (51.9 mg, 57% yield, >10:1 selectivity). **^{1}H NMR** (400 MHz, $CDCl_3$) *δ* 7.16 (m, 1H), 7.08 (m, 2H), 6.66 (d, *J* = 2.3 Hz, 1H), 6.57 (d, *J* = 2.3 Hz, 1H), 4.00 (t, *J* = 6.6 Hz, 2H), 2.81 (q, *J* = 7.6 Hz, 2H), 2.08 (s, 6H), 1.80 (m, 2H), 1.46 (m, 2H), 1.22-1.38 (m, 15H), 0.89 (m, 3H). **^{13}C NMR** (101 MHz, $CDCl_3$) *δ* 166.3, 165.4, 161.0, 141.3, 136.1, 127.9, 127.8, 108.0, 106.8, 68.2, 32.2, 32.1, 29.9, 29.7, 29.6, 29.3, 26.3, 23.0, 20.5, 14.8, 14.4. **HRMS** (DART) $[M+H]^+$ calcd. for $[C_{25}H_{38}NO]^+$ 368.2948, 368.2981 found. **IR** (neat, cm^{-1}) 2923, 2854, 1589, 1347, 1172, 1037.

***tert*-butyl 2-(4-((2-ethylhexyl)oxy)pyridin-2-yl)pyrrolidine-1-carboxylate (32)**

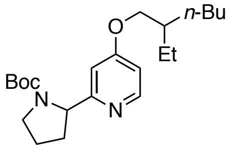

The title product was prepared according to the general procedure using *tert*-butyl 2-(5-bromopyridin-2-yl)pyrrolidine-1-carboxylate (326.1 mg, 1.0 mmol, 1.0 equiv) and 2-ethyl-1-hexanol (130.2 mg, 1.0 mmol, 1.0 equiv). Silica gel chromatography (40% EtOAc/hexanes) yielded the title compound as a clear oil (204.3 mg, 54% yield, >10:1 selectivity). **^{1}H NMR** (400 MHz, $CDCl_3$) Note: broad signals are observed; *δ* 8.32 (d, *J* = 5.6 Hz, 1H), 6.58-6.70 (m, 2H), 4.87 (m, 1H), 3.84 (m, 2H), 3.43-3.70 (m, 2H), 2.32 (m, 1H), 2.01 (m, 1H), 1.79-1.93 (m, 2H), 1.70 (m, 1H), 1.35-1.55 (m, 7H), 1.26-1.34 (m, 4H), 1.17-1.26 (m, 6H), 0.85-0.96 (m, 6H). **^{13}C NMR** (101 MHz, CD_3OD) (mixture of rotamers) *δ* 168.9, 167.3, 166.3, 157.1, 157.0, 151.9, 151.8, 110.7, 108.9, 108.7, 82.0, 81.8, 72.4, 64.8, 64.2, 49.1, 41.3, 41.3, 36.2, 35.2, 32.4, 32.4, 31.0, 29.6, 29.3, 25.8, 25.7, 25.5, 25.1, 24.9, 15.3, 12.3. **HRMS** (DART) $[M+H]^+$ calcd. for $[C_{22}H_{37}N_2O_3]^+$ 377.2799, 377.2818 found. **IR** (neat, cm^{-1}) 2960, 2929, 1693, 1594, 1389, 1162, 1114.

4-(1-cyclopropylethoxy)-2-(2-phenyl-1,3-dioxolan-2-yl)pyridine (33)

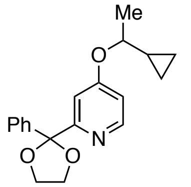

The title product was prepared according to the general procedure using 5-bromo-2-(2-phenyl-1,3-dioxolan-2-yl)pyridine (305.0 mg, 1.0 mmol, 1.0 equiv) and 1-cyclopropylethanol (86.1 mg, 1.0 mmol, 1.0 equiv). Silica gel chromatography (40% EtOAc/hexanes) yielded the title compound as a white solid (181.1 mg, 58% yield, approximately 6:1 selectivity). **Melting Point**: 69 °C – 72 °C. **^{1}H NMR** (400 MHz, $CDCl_3$) δ 8.39 (d, J = 5.7 Hz, 1H), 7.62 (m, 2H), 7.25-7.37 (m, 3H), 7.15 (d, J = 2.5 Hz, 1H), 6.63 (dd, J = 2.5 Hz, 5.7 Hz, 1H), 4.10 (m, 4H), 3.96 (m, 1H), 1.36 (d, J = 6.1 Hz, 3H), 1.12 (m, 1H), 0.58 (m, 2H), 0.39 (m, 1H), 0.30 (m, 1H). **^{13}C NMR** (101 MHz, $CDCl_3$) δ 165.2, 162.3, 151.2, 141.5, 128.4, 128.4, 126.4, 110.2, 108.9, 108.2, 77.8, 65.5, 19.4, 16.8, 3.9, 2.5. **HRMS** (DART) $[M+H]^+$ calcd. for $[C_{19}H_{22}NO_3]^+$ 312.1594, 312.1607 found. **IR** (neat, cm^{-1}) 2973, 2884, 1595, 1561, 1470, 1301, 1072.

***tert*-butyl 2-(4-(5-bromoindolin-1-yl)pyridin-2-yl)pyrrolidine-1-carboxylate (38)**

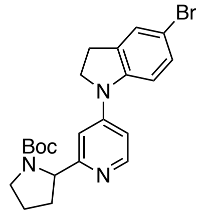

The title product was prepared according to the general procedure using *tert*-butyl 2-(5-bromopyridin-2-yl)pyrrolidine-1-carboxylate (326.1 mg, 1.0 mmol, 1.0 equiv) and 5-bromoindoline (198.1 mg, 1.0 mmol, 1.0 equiv). Silica gel chromatography (40% EtOAc/hexanes – 100% EtOAc) yielded the title compound as a brown oil (220.8 mg, 50% yield, >10:1 selectivity). **^{1}H NMR** (400 MHz, $CDCl_3$) Note: broad signals are observed; δ 8.33 (d, J = 5.6 Hz, 1H), 7.08-7.32 (m, 3H), δ 6.71-7.00 (m, 2H), δ 4.90 (m, 1H), δ 3.88-4.06 (m, 2H), δ 3.44-3.71 (m, 2H), 3.13 (m, 2H), 2.33 (m, 1H), 1.96-2.18 (m, 1H), 1.79-1.95 (m, 2H), 1.47 (s, 4H), 1.24 (s, 5H). **^{13}C NMR** (101 MHz, $CDCl_3$) (mixture of rotamers) δ 164.9, 163.3, 155.0, 154.9, 150.2, 150.0, 144.0, 134.8, 130.2, 130.2, 128.7, 128.6, 113.1, 112.8, 111.9, 111.8, 108.8, 108.6, 106.8, 106.1, 79.6, 63.2, 62.5, 51.5, 47.8, 47.4, 34.4, 33.1, 28.8, 28.6, 27.9, 24.1, 23.5. **HRMS** (DART) $[M+H]^+$ calcd. for $[C_{22}H_{27}BrN_3O_2]^+$ 444.1281 and , 444.1288 found. **IR** (neat, cm^{-1}) 2973, 2875, 1686, 1584, 1485, 1388, 1158.

2-methyl-1-(2-(2-phenyl-1,3-dioxolan-2-yl)pyridin-4-yl)indoline (39)

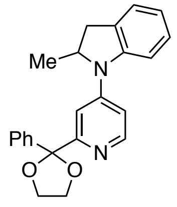

The title product was prepared according to the general procedure using 5-bromo-2-(2-phenyl-1,3-dioxolan-2-yl)pyridine (305.0 mg, 1.0 mmol, 1.0 equiv) and 2-methylindoline (133.1 mg, 1.0 mmol, 1.0 equiv). Silica gel chromatography (40% EtOAc/hexanes – 100% EtOAc) yielded the title compound as a light yellow oil (184.8 mg, 52% yield, >10:1 selectivity). **^{1}H NMR** (400 MHz, $CDCl_3$) δ 8.38 (d, J = 5.8 Hz, 1H), 7.64-7.67 (m, 2H), 7.46 (d, J = 2.4 Hz, 1H), 7.14-7.38 (m, 6H), 6.93 (dd, J = 2.4 Hz, 5.8 Hz, 1H), 6.90 (td, J = 1.0 Hz, 7.4 Hz, 1H), 4.45 (m, 1H), 4.12 (m, 4H), 3.39 (dd, J = 8.8 Hz, 15.3 Hz, 1H), 2.63 (dd, J = 2.6 Hz, 15.3 Hz, 1H), 1.32 (d, J = 6.3 Hz, 3H). **^{13}C NMR** (101 MHz, d_6-DMSO) δ 160.7, 150.0, 148.8, 142.7, 141.6, 131.5, 127.8, 127.7, 127.1, 126.1, 125.9, 121.4, 111.7, 108.9, 108.2, 105.3, 64.8, 58.1, 35.8, 19.4. **HRMS** (DART) $[M+H]^+$ calcd. for $[C_{16}H_{22}NO]^+$ 359.1754, 359.1765 found. **IR** (neat, cm^{-1}) 2969, 2888, 1583, 1485, 1390, 1087, 989.

1-(2-(8-methoxy-1,4-dioxaspiro[4.5]decan-8-yl)pyridin-4-yl)indoline (40)

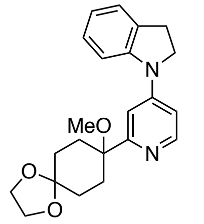

The title product was prepared according to the general procedure using 5-bromo-2-(8-methoxy-1,4-dioxaspiro[4.5]decan-8-yl)pyridine (327.0 mg, 1.0 mmol, 1.0 equiv) and indoline (141.8 mg, 1.2 mmol, 1.2 equiv). Silica gel chromatography (40% EtOAc/hexanes to 60% EtOAc/hexanes) yielded the title compound as a light brown solid (245.5 mg, 67% yield, approximately 9:1 selectivity). **Melting Point**: 113 °C – 115 °C. **^{1}H NMR** (400 MHz, $CDCl_3$) δ 8.38 (d, J = 5.8 Hz, 1H), 7.32 (d, J = 8.0 Hz, 1H), 7.28 (d, J = 2.4 Hz, 1H), 7.21 (m, 1H), 7.15 (m, 1H), 6.94 (dd, J = 5.8 Hz, 2.4 Hz, 1H), 6.87 (m, 1H), 3.94-4.02 (m, 6H), 3.12-3.19 (m, 5H), 2.26 (td, J = 13.5 Hz, 4.3 Hz, 2H), 1.92-2.10 (m, 4H), 1.69 (m, 2H). **^{13}C NMR** (101 MHz, $CDCl_3$) δ 164.9, 150.4, 149.7, 144.9, 132.5, 127.6, 125.7, 121.2, 110.8, 109.2, 108.8, 107.0, 79.1, 64.7, 64.5, 51.4, 50.9, 32.1, 30.8, 28.2. **HRMS** (DART) $[M+H]^+$ calcd. for $[C_{22}H_{27}N_2O_3]^+$ 367.2016, 367.2040 found. **IR** (neat, cm^{-1}) 2945, 2884, 1583, 1487, 1108, 1065, 941.

2-(2-phenyl-1,3-dioxolan-2-yl)pyridin-4(1*H*)-one (43)

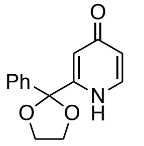

The title product was prepared according to the general procedure inside a N_2 filled glovebox. An oven-dried 4 mL vial (ThermoFisher, C4015-1) was charged with a magnetic stir bar, 5-bromo-2-(2-phenyl-1,3-dioxolan-2-yl)pyridine (76.5 mg, 0.25 mmol, 1.0 equiv), and *N*,*N*-dibenzyl-3-hydroxypropanamide (67.3 mg, 0.25 mmol, 1.0 equiv and brought into a N_2 filled glovebox. DMAc (1.5 mL, 0.17 M), KBr (14.9 mg, 0.125 mmol, 0.5 equiv), 18-crown-6 (231.3 mg, 0.875 mmol, 3.5 equiv), and KOH (42.1 mg, 0.75 mmol, 3.0 equiv) was then added in successive order. The reaction vial was sealed with a PTFE-lined screw cap (ThermoFisher, C4015-1A), removed from the glovebox and placed into a preheated 80 °C aluminum reaction block. The crude reaction solution was cooled to rt and directly loaded onto a silica gel column that had been neutralized with NEt_3 and eluted on a gradient (1% MeOH/CH_2Cl_2 to 5% MeOH/CH_2Cl_2) to yield the title compound as a light yellow solid (30.1 mg, 0.124 mmol 50% yield, >10:1 selectivity). **Melting Point**: 213 °C – 217 °C. **^{1}H NMR** (400 MHz, CD_3OD) δ 7.75 (d, J = 7.2 Hz, 1H), 7.56 (m, 2H), 7.38-7.45 (m, 3H), 6.54 (d, J = 2.6 Hz, 1H), 6.43 (dd, J = 7.2 Hz, 2.6 Hz, 1H), 4.17 (m, 4H). **^{13}C NMR** (CD_3OD, 101 MHz) δ 182.5, 154.0, 140.6, 140.5, 130.8, 130.1, 127.6, 117.6, 116.0, 108.0, 67.3. **HRMS** (DART) $[M+H]^+$ calcd. for $[C_{14}H_{14}NO_3]^+$ 244.0968, 244.0984 found. **IR** (neat, cm^{-1}) 2894, 1628, 1519, 1215, 1072, 989.

VII. Convergence of 3- and 5-Bromopyridine Isomers

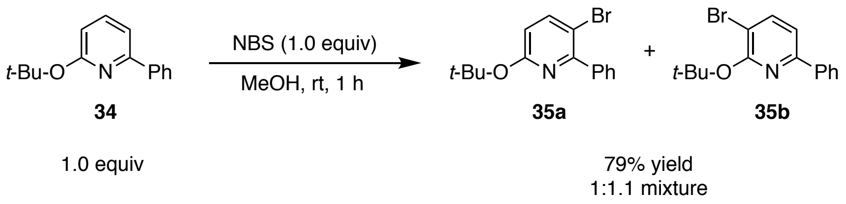

3-bromo-6-(*tert*-butoxy)-2-phenylpyridine (35a) and 3-bromo-2-(*tert*-butoxy)-6-phenylpyridine (35b) mixture: Inside a N_2 filled glovebox, an oven-dried 100 mL round bottom flask was charged with 2-(*tert*-butoxy)-6-phenylpyridine (1.32 g, 5.8 mmol, 1.0 equiv) and anhydrous MeOH (20.0 mL, 0.29 M). *N*-Bromosuccinimide (1.03 g, 5.8 mmol, 1.0 equiv) was then added slowly portionwise. The flask was sealed with a red septum (VWR, Cat. No. 89097-544) and the edges of the septum were sealed with Parafilm®. The flask was removed from the glovebox, put under positive pressure with an N_2 balloon, and stirred at rt for one hour. The reaction solution was then poured into H_2O (70 mL) in a separatory funnel and the product was extracted with EtOAc (3 x 60 mL). The organic layer was dried over Na_2SO_4. The Na_2SO_4 was filtered off and the organic layer was concentrated *in vacuo*. Silica gel chromatography (1% EtOAc/hexanes) yielded the title compounds as a 1.1:1 mixture of isomers (1.39 g, 79% yield). **Melting Point**: 37 °C – 39 °C. **^{1}H NMR** (400 MHz, $CDCl_3$) *mixture of regioisomers δ 7.98 (both isomers) (m, 2H), 7.82 (single isomer) (d, J = 8.0 Hz, 1H), 7.39-7.75 (both isomers) (m, 10H), 7.20 (single isomer) (d, J = 8.0 Hz, 1H), 6.51 (single isomer) (d, J = 8.6 Hz, 1H), 1.71 (s, 9H), 1.59 (s, 9H). **^{13}C NMR** (101 MHz, $CDCl_3$) *mixture of 3- and 5-bromoregioisomers δ 162.5, 159.5, 154.3, 153.5, 143.8, 142.3, 140.1, 138.8, 129.8, 129.3, 129.1, 128.7, 128.0, 126.9, 113.6, 113.6, 109.8, 107.8, 81.6, 80.6, 29.0, 28.9. **HRMS** (DART) $[M+H]^+$ calcd. for $[C_{15}H_{17}BrNO]^+$ 306.0488, 306.0504 found. **IR** (neat, cm^{-1}) 2968, 2360, 1566, 1424, 1322, 1164, 1120.

Note: Provided below are ^{1}H and ^{13}C NMR spectra of the obtained brominated mixture.

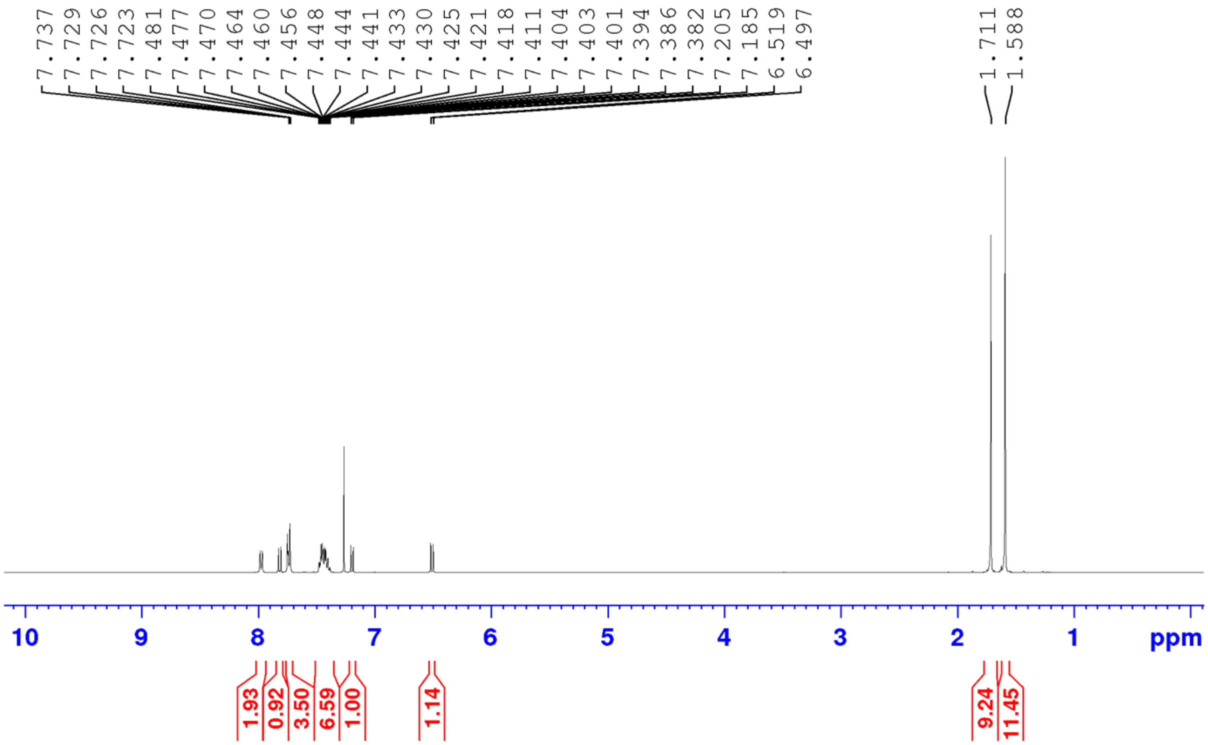

Figure S24: ^{1}H NMR spectrum (400 MHz, $CDCl_3$) of the purified 1.1:1 mixture of **35a** and **35b**.

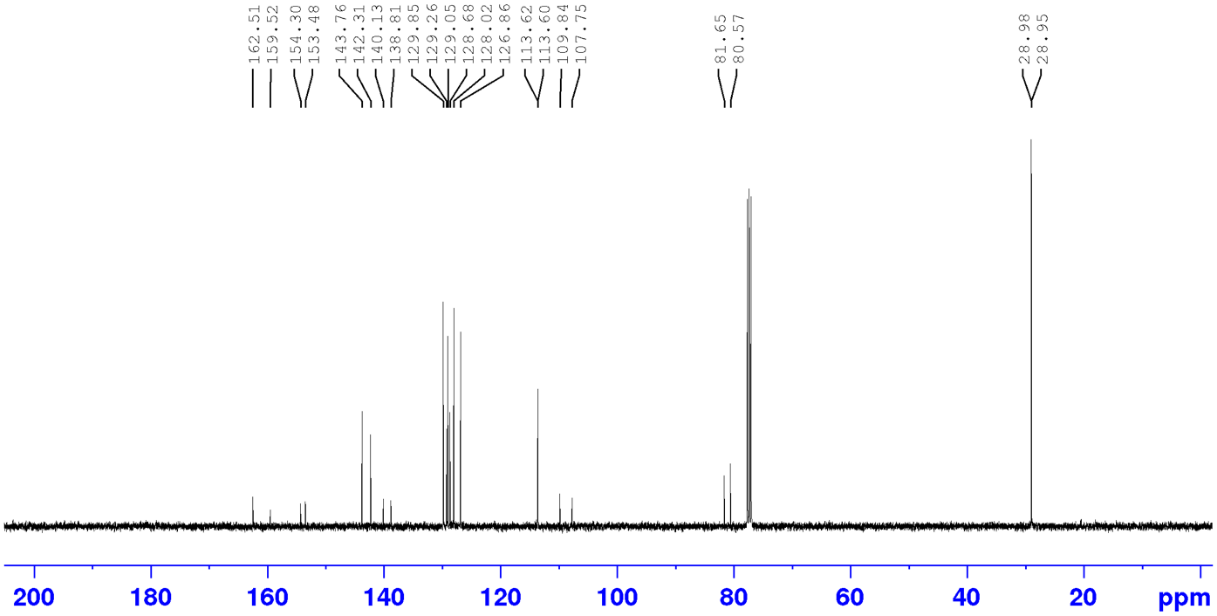

Figure S25: ^{13}C NMR spectrum (101 MHz, $CDCl_3$) of the purified 1.1:1 mixture of **35a** and **35b**.

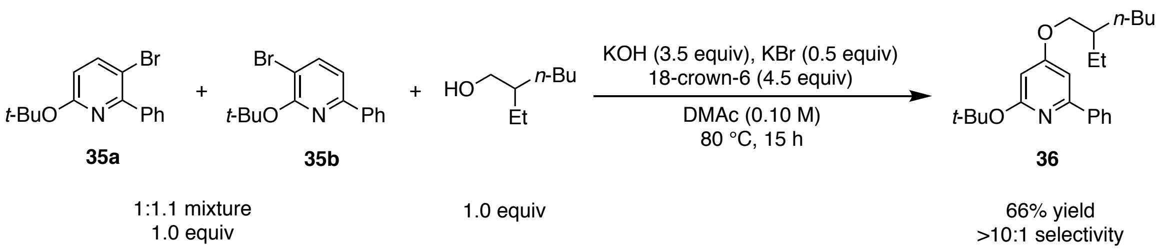

2-(*tert*-butoxy)-4-((2-ethylhexyl)oxy)-6-phenylpyridine (36)

Procedure: Inside a N_2 filled glovebox, an oven-dried 25 mL round bottom flask was charged with a magnetic stir bar, a 1.1:1 mixture of 3-bromo-2-(*tert*-butoxy)-6-phenylpyridine and 3-bromo-6-(*tert*-butoxy)-2-phenylpyridine (152.5 mg, 0.5 mmol, 1.0 equiv), 2-ethyl-1-hexanol (65.1 mg, 0.5 mmol, 1.0 equiv), DMAc (5.0 mL, 0.10 M), KBr (29.8 mg, 0.25 mmol, 0.5 equiv), 18-crown-6 (594.7 mg, 2.25 mmol, 4.5 equiv), and KOH (98.2 mg, 1.75 mmol, 3.5 equiv) in successive order. The flask was sealed with a Fisherbrand® red septum stopper (Cat. No. FB57875) and the edges of the septum were then sealed with Parafilm®. The flask was then removed from the glovebox, put under positive pressure with N_2 on a Schlenk line, and placed into a preheated 80 °C silicon oil bath. The solution was stirred at 80 °C for 15 h and then allowed to cool to rt. The selectivity was determined according to the general procedure in Section V. The solution was poured into a separatory funnel containing water (50 mL). The product was then extracted with EtOAc (3 x 50 mL). The organic layer was dried over Na_2SO_4. The Na_2SO_4 was then filtered and the solution was concentrated *in vacuo*. Silica gel chromatography (1% EtOAc/hexanes to 2% EtOAc/hexanes) yielded the title compound as a clear oil (117.6 mg, 66% yield, >10:1 selectivity). **^{1}H NMR** (400 MHz, $CDCl_3$) δ 8.00 (m, 2H), 7.45 (m, 2H), 7.38 (m, 1H), 6.92 (d, J = 1.9 Hz, 1H), 6.11 (d, J = 1.9 Hz, 1H), 3.89 (m, 2H), 1.75 (m, 1H), 1.68 (s, 9H), 1.38-1.57 (m, 4H), 1.29-1.37 (m, 4H), 0.88-0.98 (m, 6H). **^{13}C NMR** (101 MHz, $CDCl_3$) δ 168.4, 165.5, 155.3, 139.8, 128.9, 128.8, 127.0, 102.7, 95.4, 79.8, 70.7, 39.5, 30.8, 29.4, 29.2, 24.2, 23.4, 14.4, 11.4. **HRMS** (DART) $[M+H]^+$ calcd. for $[C_{23}H_{34}NO_2]^+$ 356.2584, 356.2601 found. **IR** (neat, cm^{-1}) 2960, 2928, 1596, 1571, 1418, 1151.

VIII. Preparative scale 4-selective amination of 3-bromopyridine

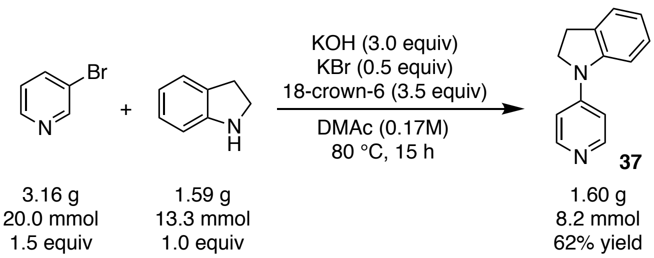

1-(pyridin-4-yl)indoline (37)

Procedure: An oven-dried 250 mL round bottom flask was charged with a magnetic stir bar, 18-crown-6 (12.30 g, 46.6 mmol, 3.5 equiv), KBr (797.4 mg, 6.7 mmol, 0.5 equiv), and KOH (2.24 g, 39.9 mmol, 3.0 equiv). The flask was sealed with a Fisherbrand® red septum stopper (Cat. No. FB57875) and the edges of the septum were then sealed with Parafilm®. The flask was evacuated and backfilled three times with N_2 and then left under positive pressure of N_2 on a Schlenk line. DMAc (68 mL) was then added and the solution was stirred at rt (***Note***: there are some insoluble salts remaining at the bottom of the flask). 3-Bromopyridine (3.16 g, 20.0 mmol, 1.5 equiv) and indoline (1.59 g, 13.3 mmol, 1.0 equiv) were then measured into a 20 mL scintillation vial and constituted in 10 mL of DMAc and sparged with N_2 for 5 min. This solution was then added dropwise via syringe to the 250 mL round bottom flask. The reaction solution was then placed into a preheated 80 °C silicon oil bath and stirred for 15 h. The solution was cooled to rt and 1,3,5-trimethoxybenzene internal standard was added and the 4-selectivity was determined using the general procedure described in Section V. The solution was then poured into a separatory funnel with H_2O (700 mL) and the product was extracted with EtOAc (3 x 250 mL). The organic layers were then pooled and poured back into the separatory funnel and washed with H_2O (1 x 200 mL) and brine (1 x 100 mL). The organic layer was then dried over Na_2SO_4. The Na_2SO_4 was filtered off and the organic layer was concentrated *in vacuo*. Purification by silica gel chromatography (50% EtOAc/hexanes to 100% EtOAc to 5% MeOH/CH_2Cl_2 to 10% MeOH/ CH_2Cl_2) yielded the title compound as a brown solid (1.60 g, 8.2 mmol, 62% yield, >10:1 selectivity). **Melting Point**: 71 °C – 76 °C. **^{1}H NMR** (400 MHz, $CDCl_3$) δ 8.39 (m, 2H), 7.33 (d, J = 8.0 Hz, 1H), 7.22 (dd, J = 0.6 Hz, 7.3 Hz, 1H), 7.15 (m, 1H), 7.03 (m, 2H), 6.89 (td, J = 0.6 Hz, 7.4 Hz, 1H), 4.00 (t, J = 8.4 Hz, 2H), 3.18 (t, J = 8.4 Hz, 2H). **^{13}C NMR** (101 MHz, $CDCl_3$) δ 150.5, 149.8, 144.7, 132.6, 127.5, 125.8, 121.5, 111.0, 110.5, 51.3, 28.2. **HRMS** (DART) $[M+H]^+$ calcd. for $[C_{13}H_{13}N_2]^+$ 197.1073, 197.1075 found. **IR** (neat, cm^{-1}) 3025, 2945, 1581, 1504, 1392, 1222.

IX. Preparation of 3- or 5-bromopyridine starting materials

(a) Preparation of simple 3-bromopyridine substrates

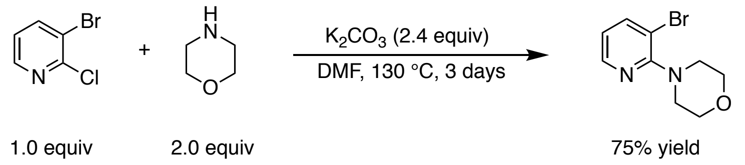

4-(3-bromopyridin-2-yl)morpholine: Title compound was prepared according to a reported procedure.[2] Spectroscopic characterization is consistent with a previous report.[2] **^{1}H NMR** (400 MHz, $CDCl_3$) δ 8.25 (dd, J = 1.6 Hz, 4.7 Hz, 1H), 7.80 (dd, J = 1.6 Hz, 7.7 Hz, 1H), 6.80 (dd, J = 4.7 Hz, 7.7 Hz, 1H), 3.87 (m, 4H), 3.35 (m, 4H).

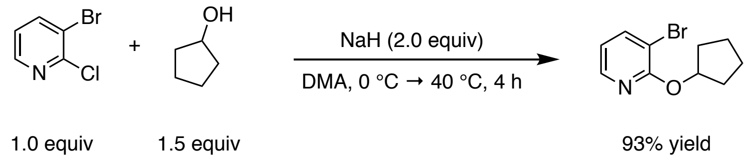

3-bromo-2-(cyclopentyloxy)pyridine: NaH (60% dispersion, 800 mg, 20.0 mmol, 2.0 equiv) was weighed into an oven-dried 100 mL round bottom flask. The flask was sealed with a septum stopper (VWR, Cat. No. 89097-544). The flask was evacuated and backfilled three times with N_2 and then left under positive pressure with a N_2 balloon. The flask was cooled to 0 °C, and then DMAc (20 mL) was added. Cyclopentanol (1.29 g, 15.0 mmol, 1.5 equiv) was then added dropwise to the slurry. The solution was allowed to stir at 0 °C for 10 min, then 3-bromo-2-chloropyridine (1.92g, 10.0 mmol, 1.0 equiv) constituted in DMAc (20 mL) was added dropwise. Upon completion of the addition, the solution was allowed to warm to rt and was then placed into a 40 °C preheated silicon oil bath. The solution was stirred at 40 °C for 4 h. The solution was then allowed to cool to rt and was quenched dropwise with H_2O (ca. 1.0 mL). The mixture was then poured into H_2O (100 mL) in a separatory funnel and the product was extracted with EtOAc (3 x 80 mL). The organic layers were dried over Na_2SO_4. The Na_2SO_4 was filtered off and the organic layer was concentrated *in vacuo*. Purification of the resultant residue by silica gel chromatography (5% EtOAc/hexanes) yielded the title compound as a clear oil (2.23 g, 9.3 mmol, 93% yield). **^{1}H NMR** (400 MHz, $CDCl_3$) δ 8.07 (dd, J = 1.7 Hz, 4.9 Hz, 1H), 7.77 (dd, J = 1.7 Hz, 7.6 Hz, 1H), 6.72 (dd, J = 4.9 Hz, 7.6 Hz, 1H), 5.45 (m, 1H), 1.90-2.04 (m, 2H), 1.77-1.90 (m, 4H), 1.59-1.70 (m, 2H). **^{13}C NMR** (101 MHz, $CDCl_3$) δ 160.1, 145.8, 141.8, 117.5, 108.2, 79.5, 33.1, 24.2. **HRMS** (DART) $[M+H]^+$ calcd. for $[C_{10}H_{13}BrNO]^+$ 242.0175, 242.0182 found. **IR** (neat, cm^{-1}) 2960, 2871, 1580, 1430, 1296, 1025, 971.

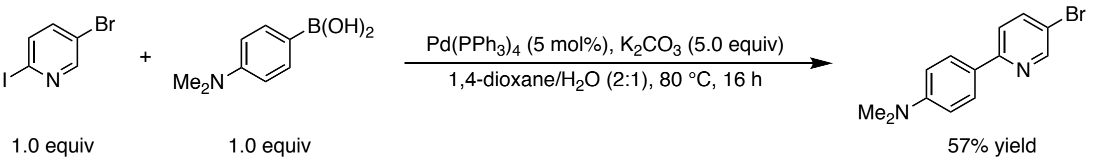

4-(5-bromopyridin-2-yl)-*N*,*N*-dimethylaniline: Inside a N_2 filled glovebox, an oven-dried 100 mL round bottom flask was charged with a magnetic stir bar, 5-bromo-2-iodopyridine (851.7 mg, 3.0 mmol, 1.0 equiv), 4-(*N*,*N*-dimethylamino)phenylboronic acid (495.0 mg, 3.0 mmol, 1.0 equiv), 1,4-dioxane (13 mL), tetrakis(triphenylphosphine)palladium(0) (173.3 mg, 0.15 mmol, 0.05 equiv), and K_2CO_3 (1.50 g, 15.0 mmol, 5.0 equiv). The flask was sealed with a red septum (VWR, Cat. No. 89097-544) and removed from the glovebox. The system was put under positive pressure with a N_2 balloon, then H_2O (7 mL) was then added via syringe. The flask was then placed into a preheated 80 °C silicon oil bath and stirred for 16 h. The solution was then allowed to cool to rt and was poured into a separatory funnel containing H_2O (70 mL). The product was extracted with EtOAc (3 x 60 mL). The organic layers were dried over Na_2SO_4. The Na_2SO_4 was filtered out and the organic layer was concentrated *in vacuo*. Silica gel chromatography (3% EtOAc/hexanes to 10% EtOAc/hexanes) yielded the title compound as a light yellow powder (470.6 mg, 1.7 mmol, 57% yield). **Melting Point**: 150 °C – 152 °C. **^{1}H NMR** (400 MHz, $CDCl_3$) δ 8.64 (d, J = 2.4 Hz, 1H), 7.88 (d, J = 9.0 Hz, 2H), 7.76 (dd, J = 2.4 Hz, 8.6 Hz, 1H), 7.53 (d, J = 8.6 Hz, 1H), 6.78 (d, J = 9.0 Hz, 2H), 3.02 (s, 6H). **^{13}C NMR** (101 MHz, $CDCl_3$) δ 156.4, 151.6, 150.6, 139.3, 128.0, 126.2, 120.5, 117.6, 112.5, 40.6. **HRMS** (DART) $[M+H]^+$ calcd. for $[C_{13}H_{14}BrN_2]^+$ 277.0335, 277.0355 found. **IR** (neat, cm^{-1}) 2799, 2360, 2342, 1606, 1454, 1325, 1091.

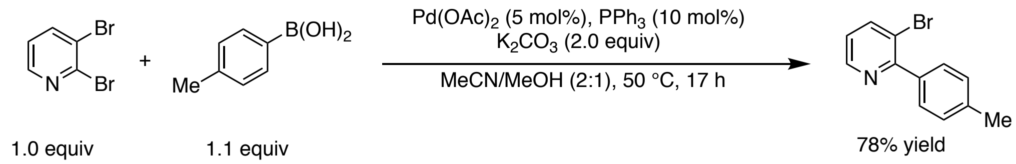

3-bromo-2-(*p*-tolyl)pyridine: Inside a N_2 filled glovebox, an oven-dried 100 mL round bottom flask was charged with a magnetic stir bar, 2,3-dibromopyridine (947.6 mg, 4.0 mmol, 1.0 equiv), *p*-tolylboronic acid (598.2 mg, 4.4 mmol, 1.1 equiv), anhydrous MeCN/MeOH (2:1, 50 mL), palladium(II) acetate (44.9 mg, 0.20 mmol, 0.05 equiv), triphenylphosphine (104.9 mg, 0.4 mmol, 0.1 equiv), and K_2CO_3 (800.9 mg, 8.0 mmol, 2.0 equiv). The flask was sealed with a red septum (VWR, Cat. No. 89097-544) and removed from the glovebox. The system was put under positive pressure with a N_2 balloon and then placed into a preheated 50 °C silicon oil bath and stirred for 17 h. The solution was then allowed to cool to rt and was poured into a separatory funnel containing H_2O (70 mL). The product was extracted with EtOAc (3 x 60 mL). The organic layers were dried over Na_2SO_4. The Na_2SO_4 was filtered out and the organic layer was concentrated *in vacuo*. Silica gel chromatography (8% EtOAc/hexanes) yielded the title compound as a clear oil (774.2 mg, 3.1 mmol, 78% yield). Spectroscopic characterization matches that of reported literature.[3] **^{1}H NMR** (400 MHz, $CDCl_3$) δ 8.62 (d, J = 4.6 Hz, 1H), 7.98 (d, J = 8.0 Hz, 1H), 7.60 (d, J = 8.0 Hz, 2H), 7.28 (d, J = 8.0 Hz, 2H), 7.13 (dd, J = 4.6 Hz, 8.0 Hz, 1H), 2.43 (s, 3H).

(b) Preparation of 3-bromopyridines where corresponding 4-halopyridine is less available

Note on the commercial availability of 3-bromopyridines vs. 4-halopyridines: For the substrates below, the 3-bromopyridine derivative is more readily available and/or cheaper than a corresponding 4-halogenated derivative. For these substrates, a comparison of the cheapest price for 1 gram of both the 3-bromopyridine and a 4-halopyridine (as reported in the online www.emolecules.com database accessed March 2, 2020) starting material is provided in Figure **S26** below. The obtained estimated times of delivery are also included. The comparison is meant to provide a general idea of the relative cost and availability of these substrates compared to their 4-halogenated or 4-hydroxylated derivatives.

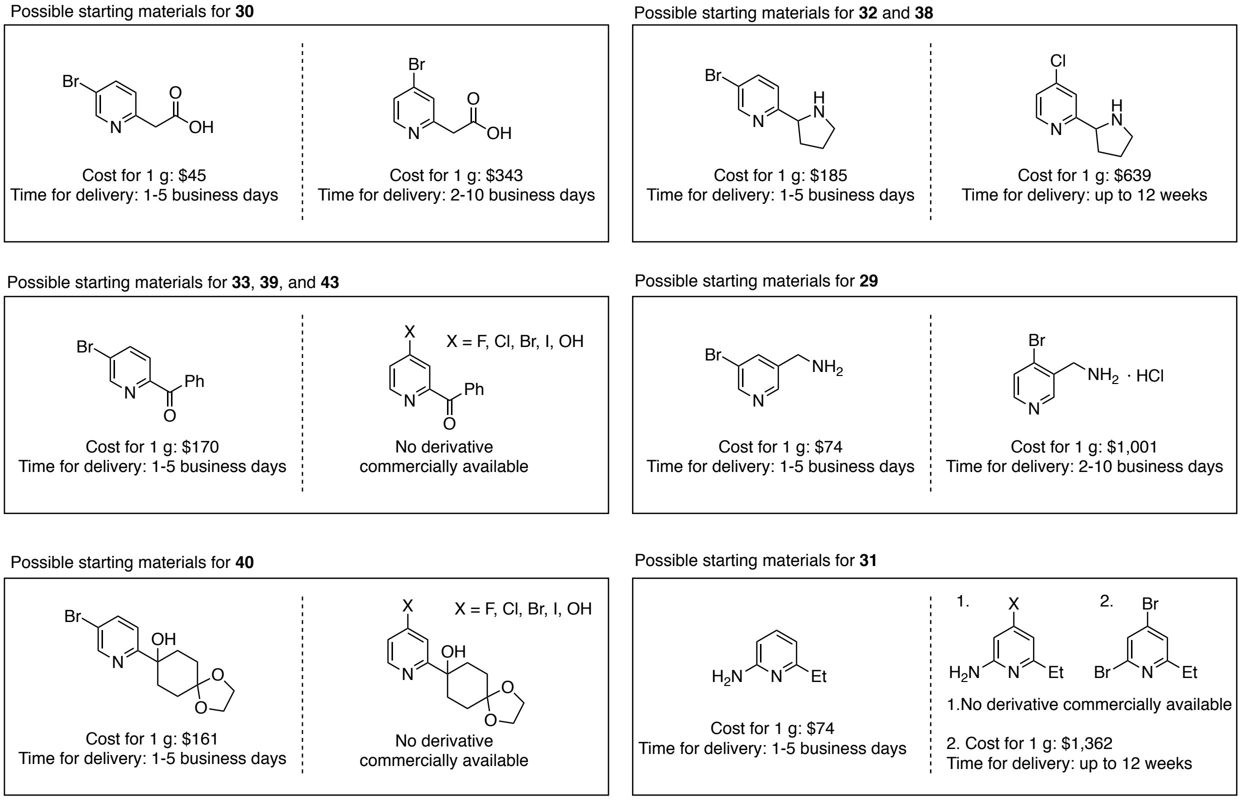

Figure S26: Comparison of commercially available 3-bromopyridine starting materials compared to corresponding 4-halogenated or 4-hydroxylated starting materials.

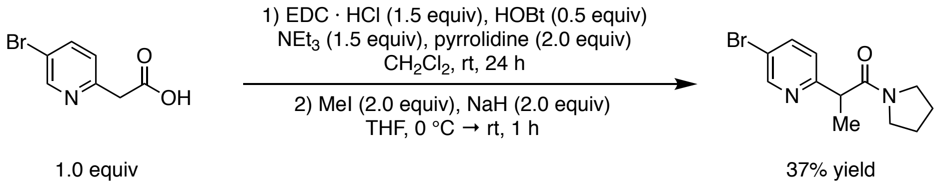

2-(5-bromopyridin-2-yl)-1-(pyrrolidin-1-yl)propan-1-one: A 250 mL round bottom flask was charged with a magnetic stir bar, *N*-(3-dimethylaminopropyl)-*N*'-ethylcarbodiimide hydrochloride (1.34 g, 7.0 mmol, 1.5 equiv), 1-hydroxybenzotriazole hydrate (310.8 mg, 2.3 mmol, 0.5 equiv), anhydrous CH_2Cl_2 (30 mL, 0.16 M), triethylamine (1.0 mL, 7.0 mmol, 1.5 equiv), 2-(5-bromopyridin-2-yl)acetic acid (1.0 g, 4.7 mmol, 1.0 equiv), and pyrrolidine (0.8 mL, 9.3 mmol, 2.0 equiv) in successive order. The solution was stirred at rt for 24 h, and then diluted with CH_2Cl_2 (50 mL). The solution was then washed with saturated aqueous NH_4Cl (1 x 50 mL), H_2O (1 x 50 mL), and brine (1 x 30 mL). The organic layer was dried over Na_2SO_4. The Na_2SO_4 was filtered out and the organic layer was concentrated *in vacuo*. Silica gel chromatography (100% CH_2Cl_2 to 5% MeOH/CH_2Cl_2) yielded 2-(5-bromopyridin-2-yl)-1-(pyrrolidin-1-yl)ethan-1-one as a yellow solid (560.0 mg, 2.1 mmol, 44% yield). **^{1}H NMR** (400 MHz, $CDCl_3$) δ 8.57 (d, J = 2.4 Hz, 1H), 7.76 (dd, J = 2.4 Hz, 8.4 Hz, 1H), 7.31 (d, J = 8.4 Hz, 1H), 3.81 (s, 2H), 3.55 (t, J = 6.7 Hz, 2H), 3.48 (t, J = 6.9 Hz, 2H), 1.95 (m, 2H), 1.86 (m, 2H).

2-(5-bromopyridin-2-yl)-1-(pyrrolidin-1-yl)ethan-1-one (560.0 mg, 2.1 mmol, 1.0 equiv) from the previous step was constituted in 5.0 mL of anhydrous THF in a 25 mL round bottom flask. The solution was cooled to 0 °C and NaH (60% dispersion, 168.0 mg, 4.2 mmol, 2.0 equiv) was added slowly. MeI (260 µl, 4.2 mmol, 2.0 equiv) was then added dropwise. The solution was warmed to rt and stirred for 1 h. The reaction solution was quenched slowly with H_2O (ca. 0.2 mL) until the solution ceased evolving H_2 gas. The solution was then loaded directly onto a silica column and eluted with 2% MeOH/CH_2Cl_2 to yield the title compound as an off-white solid (495.8 mg, 1.76 mmol, 84% yield). **Melting Point**: 62 °C – 64 °C. **^{1}H NMR** (400 MHz, $CDCl_3$) δ 8.55 (m, 1H), 7.77 (dd, J = 2.2 Hz, 8.4 Hz, 1H), 7.34 (d, J = 8.4 Hz, 1H), 4.03 (q, J = 7.0 Hz, 1H), 3.51 (m, 2H), 3.40 (m, 2H), 1.75-1.99 (m, 4H), 1.50 (d, J = 7.0 Hz, 3H). **^{13}C NMR** (101 MHz, $CDCl_3$) δ 171.4, 160.3, 150.2, 139.9, 123.4, 119.3, 47.2, 47.0, 46.4, 26.4, 24.6, 18.7. **HRMS** (DART) $[M+H]^+$ calcd. for $[C_{12}H_{16}BrN_2O]^+$ 283.0441, 283.0461 found. **IR** (neat, cm^{-1}) 2966, 2360, 2342, 1637, 1421, 1372, 1008.

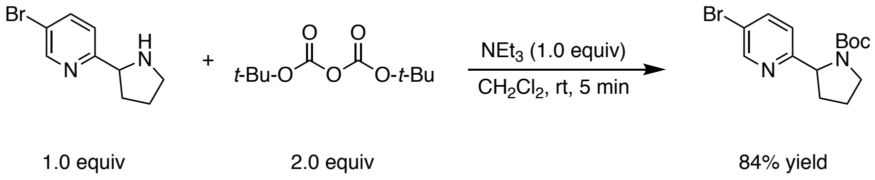

***tert*-butyl 2-(5-bromopyridin-2-yl)pyrrolidine-1-carboxylate** An oven-dried 25 mL round bottom flask was charged with a magnetic stir bar, 5-bromo-2-(pyrrolidin-2-yl)pyridine (500 mg, 2.2 mmol, 1.0 equiv), anhydrous CH_2Cl_2 (4.0 mL), di-*tert*-butyl dicarbonate (960.5 mg, 4.4 mmol, 2.0 equiv), and triethylamine (300 µl, 2.2 mmol, 1.0 equiv) in successive order. The solution was stirred at rt for 5 min and then loaded directly onto silica gel column and eluted (25% EtOAc/hexanes) to afford the title compound as a white solid (603.4 mg, 1.9 mmol, 84% yield). **Melting Point**: 37 °C – 40 °C. **^{1}H NMR** (mixture of rotamers) (400 MHz, $CDCl_3$) δ 8.58 (m, 1H), 7.74 (m, 1H), 7.08 (m, 1H), 4.87 (m, 1H), 3.46-3.68 (m, 2H), 2.34 (m, 1H), 1.81-2.06 (m, 3H), 1.15-1.51 (m, 9H). **^{13}C NMR** (101 MHz, $CDCl_3$) (mixture of rotamers) δ 162.8, 154.7, 150.6, 150.4, 139.3, 139.1, 122.1, 121.5, 118.6, 79.9, 62.6, 62.0, 47.7, 47.4, 34.5, 33.3, 28.8, 28.5, 24.2, 23.5. **HRMS** (DART) $[M+H]^+$ calcd. for $[C_{14}H_{20}BrN_2O_2]^+$ 327.0703, 327.0711 found. **IR** (neat, cm^{-1}) 2974, 2875, 1690, 1463, 1387, 1156, 1113.

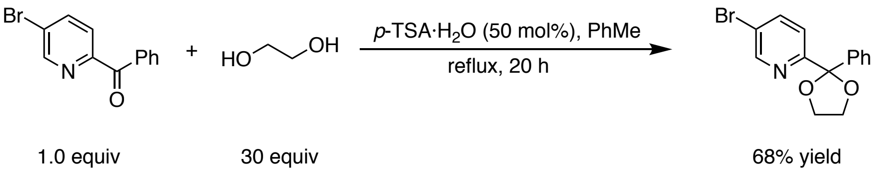

5-bromo-2-(2-phenyl-1,3-dioxolan-2-yl)pyridine: 5-Bromopyridin-2-yl)(phenyl)methanone (2.13 g, 8.2 mmol, 1.0 equiv) was added to a 100 mL round bottom flask and constituted in anhydrous toluene (25 mL, 0.33 M). *para*-Toluenesulfonic acid monohydrate (779.8 mg, 4.1 mmol, 0.5 equiv) and anhydrous ethylene glycol (14.0 mL, 246 mmol, 30 equiv) were then added. The flask was fitted with a reflux condenser and refluxed for 20 h. The solution was cooled to rt and then poured into H_2O (80 mL) in a separatory funnel. The product was extracted with EtOAc (3 x 60 mL) and the organic layers were pooled and dried over Na_2SO_4. The Na_2SO_4 was filtered out and the organic layer was concentrated *in vacuo*. Silica gel chromatography yielded the title compound as white crystals (1.69 g, 5.5 mmol, 68% yield). **Melting Point**: 112 °C – 114 °C. **^{1}H NMR** (400 MHz, $CDCl_3$) δ 8.65 (d, J = 2.3 Hz, 1H), 7.80 (dd, J = 2.3 Hz, 8.5 Hz, 1H), 7.55-7.60 (m, 3H), 7.27-7.36 (m, 3H), 4.11 (s, 4H). **^{13}C NMR** (101 MHz, $CDCl_3$) δ 159.3, 150.8, 141.0, 139.5, 128.7, 128.5, 126.3, 122.2, 120.4, 108.7, 65.5. **HRMS** (DART) $[M+H]^+$ calcd. for $[C_{14}H_{13}BrNO_2]^+$ 306.0124, 306.0140 found. **IR** (neat, cm^{-1}) 2993, 2891, 2360, 1571, 1445, 1206, 1091.

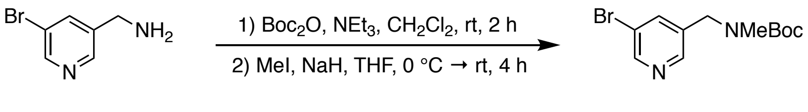

1.0 equiv 55% yield

***tert*-butyl ((5-bromopyridin-3-yl)methyl)(methyl)carbamate**: An oven-dried 4 mL vial (ThermoFisher, C4015-1) was charged with a magnetic stir bar, (5-bromopyridin-3-yl)methanamine (187.0 mg, 1.0 mmol, 1.0 equiv), anhydrous CH_2Cl_2 (1.5 mL, 0.67 M), di-*tert*-butyl dicarbonate (261.9 mg, 1.2 mmol, 1.2 equiv), and NEt_3 (210 µl, 1.5 mmol, 1.5 equiv) in successive order. The solution was stirred 2 h at rt. The solution was slowly transferred into H_2O (40 mL) in a separatory funnel and the product was extracted with CH_2Cl_2 (3 x 30 mL). The organic layers were pooled and dried over Na_2SO_4. The Na_2SO_4 was filtered off and the organic layer was concentrated *in vacuo*. Silica gel chromatography (40% EtOAc/hexanes) yielded *tert*-butyl ((5-bromopyridin-3-yl)methyl)carbamate (213.8 mg, 0.75 mmol, 75% yield). **^{1}H NMR** (400 MHz, $CDCl_3$) δ 8.59 (d, J = 2.1 Hz, 1H), 8.46 (m, 1H), 7.79 (m, 1H), 4.92 (s, 1H), 4.32 (s, 2H), 1.47 (s, 9H).

An oven-dried 25 mL round bottom flask was charged with a magnetic stir bar, *tert*-butyl ((5-bromopyridin-3-yl)methyl) (213.8 mg, 0.75 mmol, 1.0 equiv) and anhydrous THF (4.0 mL, 0.19 M). The solution was cooled to 0 °C and then NaH (60% dispersion, 36.0 mg, 0.9 mmol, 1.2 equiv) and iodomethane (51 µl, 0.83 mmol, 1.1 equiv) were added. The reaction solution was warmed to rt and stirred for 4 h. The solution was then slowly quenched with H_2O (ca. 1 mL) and then transferred to a separatory funnel containing H_2O (30 mL). The product was extracted with EtOAc (3 x 30 mL) and the organic layers were pooled and dried over Na_2SO_4. The Na_2SO_4 was filtered out and the organic layer was concentrated *in vacuo*. Silica gel chromatography (30% EtOAc/hexanes) yielded the title compound as an off-white solid (166.2 mg, 0.55 mmol, 77% step yield, 55% overall yield). **Melting Point**: 30 °C – 32 °C. **^{1}H NMR** (400 MHz, $CDCl_3$) δ 8.60 (m 1H), 8.41 (m, 1H), 7.72 (m, 1H), 4.40 (br s, 2H), 2.84 (br s, 3H), 1.47 (br s, 9H). **^{13}C NMR** (101 MHz, $CDCl_3$) 150.2, 147.5, 138.3, 137.9, 135.8, 121.2, 80.7, 50.2, 49.5, 34.6, 28.7. **HRMS** (DART) $[M+H]^+$ calcd. for $[C_{12}H_{18}BrN_2O_2]^+$ 301.0546, 301.0567 found. **IR** (neat, cm^{-1}) 3044, 2972, 1685, 1419, 1387, 1142.

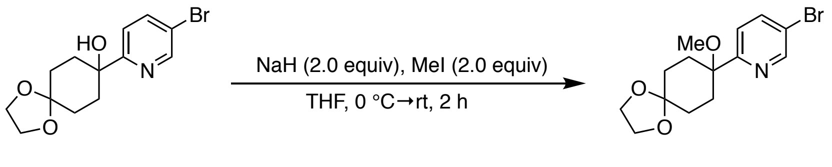

1.0 equiv 92% yield

5-bromo-2-(8-methoxy-1,4-dioxaspiro[4.5]decan-8-yl)pyridine: An oven-dried 50 mL flask was charged with a magnetic stir bar, 8-(5-bromopyridin-2-yl)-1,4-dioxaspiro[4.5]decan-8-ol (2.5 g, 8.0 mmol, 1.0 equiv), and anhydrous THF (16.0 mL, 0.5 M). The solution was cooled to 0 °C and NaH (60% dispersion, 640.0 mg, 16.0 mmol, 2.0 equiv) and iodomethane (1.0 mL, 16.0 mmol, 2.0 equiv) were added slowly. The solution was warmed to rt and stirred for 2 h. The reaction solution was quenched with H_2O (ca. 0.5 mL) and then loaded directly onto a silica gel column

and eluted (30% EtOAc/hexanes) to yield the title compound as a white powder (2.41 g, 7.4 mmol, 92% yield). **Melting Point**: 81 °C – 84 °C. **^{1}H NMR** (400 MHz, $CDCl_3$) δ 8.61 (d, J = 2.4 Hz, 1H), 7.79 (dd, J = 2.4 Hz, 8.6 Hz, 1H), 7.40 (d, J = 8.6 Hz, 1H), 3.97 (m, 4H), 3.07 (s, 3H), 2.18 (m, 2H), 1.92-2.05 (m, 4H), 1.59-1.70 (m, 2H). **^{13}C NMR** (101 MHz, $CDCl_3$) δ 163.0, 150.2, 139.3, 122.3, 119.5, 108.6, 78.8, 64.7, 64.6, 50.9, 32.0, 30.7. **HRMS** (DART) $[M+H]^+$ calcd. for $[C_{14}H_{19}BrNO_3]^+$ 328.0543, 328.0570 found. **IR** (neat, cm^{-1}) 2931, 2869, 1702, 1460, 1360, 1093, 1035.

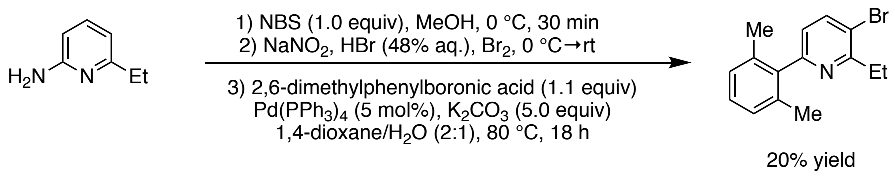

3-bromo-6-(2,6-dimethylphenyl)-2-ethylpyridine: An oven-dried 250 mL round bottom flask was charged with 2-amino-6-ethylpyridine (5.0 g, 41.0 mmol, 1.0 equiv) and anhydrous MeOH (80 mL, 0.51 M). The solution was cooled to 0 °C and *N*-bromosuccinimide (7.3 g, 41.0 mmol, 1.0 equiv) was added portion wise slowly (***Caution***: addition of *N*-bromosuccinimide is exothermic. Care should be taken to add the reagent very slowly). The solution was stirred at 0 °C for 30 min and was subsequently poured into H_2O (200 mL) in a separatory funnel. The product was extracted with EtOAc (3 x 100 mL). The organic layers were dried over Na_2SO_4. The Na_2SO_4 was filtered off and the organic layer was concentrated *in vacuo*. Purification by silica gel chromatography (20% EtOAc/hexanes to 50% EtOAc/hexanes) yielded 6-amino-3-bromo-2-ethylpyridine as a light orange solid (5.87 g, 29.4 mmol, 70% yield). **^{1}H NMR** (400 MHz, $CDCl_3$) δ 7.48 (d, J = 8.5 Hz, 1H), 6.23 (d, J = 8.5 Hz, 1H), 4.39 (br s, 2H), 2.79 (q, J = 7.6 Hz, 2H), 1.23 (t, J = 7.6 Hz, 3H).

6-amino-3-bromo-2-ethylpyridine (2.83 g, 14.2 mmol, 1.0 equiv) was added to a 50 mL round bottom flask and the flask was cooled to 0 °C; 48% aq. HBr (6.0 mL) was then added slowly and the slurry was stirred for 15 min at 0 °C. Br_2 (2.2 mL, 42.6 mmol, 3.0 equiv) was then added dropwise and the solution was stirred for an additional 15 min at 0 °C. $NaNO_2$ (2.94 g, 42.6 mmol, 3.0 equiv) constituted in H_2O (7.0 mL) was then added dropwise over approximately 10 min (***Caution***: addition of $NaNO_2$ is highly exothermic and results in the formation of a reactive diazonium salt. A blast shield should be utilized as an additional precaution). The solution was stirred for 1 h at 0 °C and 1 h at rt. The solution was cooled to 0 °C and 4 M NaOH was used to neutralize the reaction. Once the solution was basic, it was transferred to a separatory funnel and the product was extracted with Et_2O (3 x 70 mL). The organic layers were pooled and dried over Na_2SO_4. The Na_2SO_4 was filtered out and the organic layer was concentrated in vacuo. Purification by silica gel chromatography (10% CH_2Cl_2/hexanes to 15% CH_2Cl_2/hexanes) yielded 3,6-dibromo-2-ethylpyridine as a clear oil (1.73 g, 6.6 mmol, 46% yield). **^{1}H NMR** (400 MHz, $CDCl_3$) δ 7.63 (d, J = 8.2 Hz, 1H), 7.18 (d, J = 8.2 Hz, 1H), 2.94 (q, J = 7.5 Hz, 2H), 1.28 (t, J = 7.5 Hz, 3H).

Inside a N_2 filled glovebox, a 50 mL round bottom flask was charged with a magnetic stir bar, 2,6-dimethylphenylboronic acid (627.4 mg, 4.2 mmol, 1.1 equiv), potassium carbonate (1.90 g, 19.0

mmol, 5.0 equiv), palladium tetrakistriphenylphosphine (219.5 mg, 0.19 mmol, 0.05 equiv), 1,4-dioxane (10 mL), and 3,6-dibromo-2-ethylpyridine (1.0 g, 3.8 mmol, 1.0 equiv) in successive order. The flask was removed from the glovebox and put under positive pressure of N_2 on a Schlenk line. H_2O (5.0 mL) was then added via syringe. The solution was then placed into a preheated 80 °C silicon oil bath and stirred for 18 h. The solution was then allowed to cool to rt and poured into H_2O (70 mL) in a separatory funnel. The product was extracted with EtOAc (3 x 70 mL). The organic layers were dried over Na_2SO_4. The Na_2SO_4 was filtered off and the organic layer was concentrated *in vacuo*. Purification by silica gel chromatography (1% CH_2Cl_2/hexanes to 20% CH_2Cl_2/hexanes) yielded the title compound as a clear oil (669.8 mg, 2.3 mmol, 61% yield). **Melting Point**: 43 °C – 44 °C. **^{1}H NMR** (400 MHz, $CDCl_3$) δ 7.86 (d, J = 8.1 Hz, 1H), 7.18 (dd, J = 6.8 Hz, 8.3 Hz, 1H), 7.10 (m, 2H), 6.93 (d, J = 8.1 Hz, 1H), 3.04 (q, J = 7.5 Hz, 2H), 2.06 (s, 6H), 1.30 (t, J = 7.5 Hz, 3H). **^{13}C NMR** (101 MHz, $CDCl_3$) δ 161.9, 158.6, 140.7, 139.9, 136.1, 128.4, 128.0, 123.6, 119.1, 31.4, 20.6, 13.5. **HRMS** (DART) $[M+H]^+$ calcd. for $[C_{15}H_{17}BrN]^+$ 290.0539, 290.0550 found. **IR** (neat, cm^{-1}) 2975, 2172, 1973 1561, 1439, 1044.

X. References

1) Connon, S. J.; Hegarty, A. F. Substituted 3,4-pyridynes: clean cycloadditions. *J. Chem. Soc., Perkin Trans.* **2000**, *1*, 1245-1249.

2) Crawford, S. M.; Wheaton, C. A.; Mishra, V.; Stradiotto, M. Probing the effect of donor-fragment substitution in Mor-DalPhos on palladium-catalyzed C-N and C-C cross-coupling reactivity. *Canadian Journal of Chemistry* **2018**, *96*, 578-586.

3) Fan, J.; Zhang, W.; Gao, W.; Wang, T.; Duan, W.-L.; Liang, Y.; Zhang, Z. Syntheses of Benzofuranoquinolines and Analogues via Photoinduced Acceptorless Dehydrogenative Annulation of *o*-Phenylfuranylpyridines. *Org. Lett.* **2019**, *21*, 9183-9187.

XI. Copies of NMR Spectra

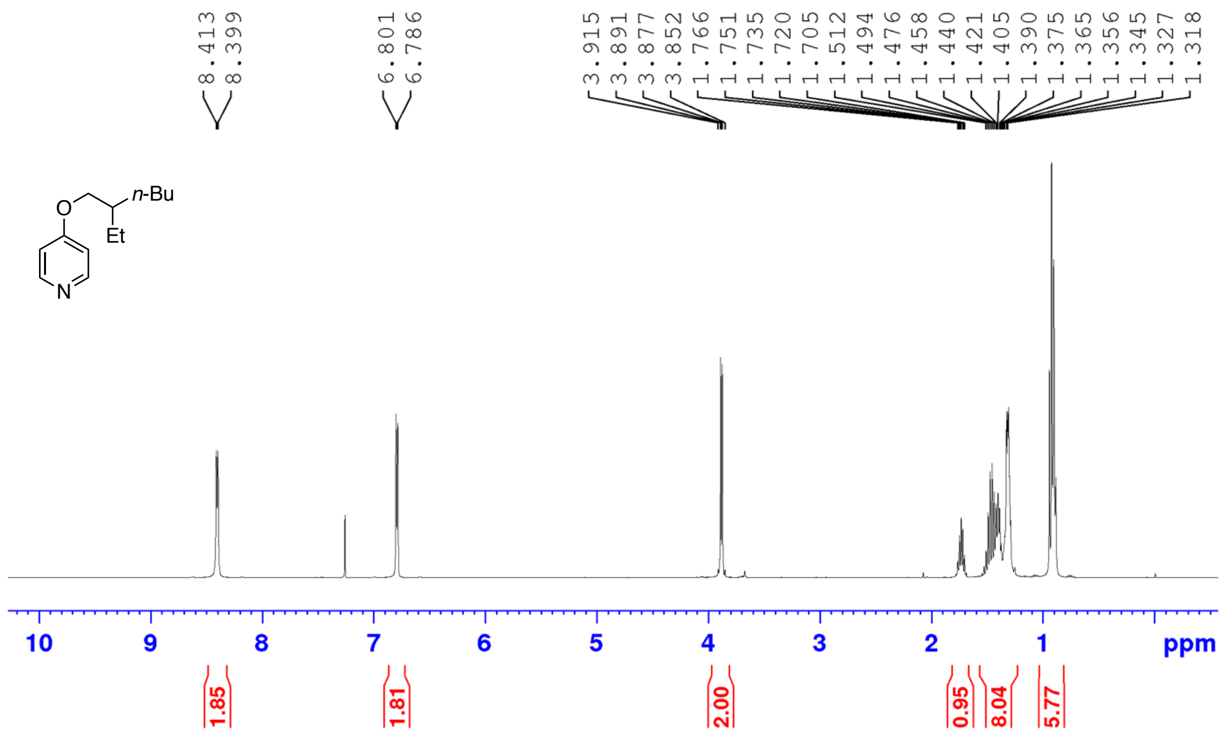

^{1}H NMR spectrum of **10** ($CDCl_3$, 400 MHz)

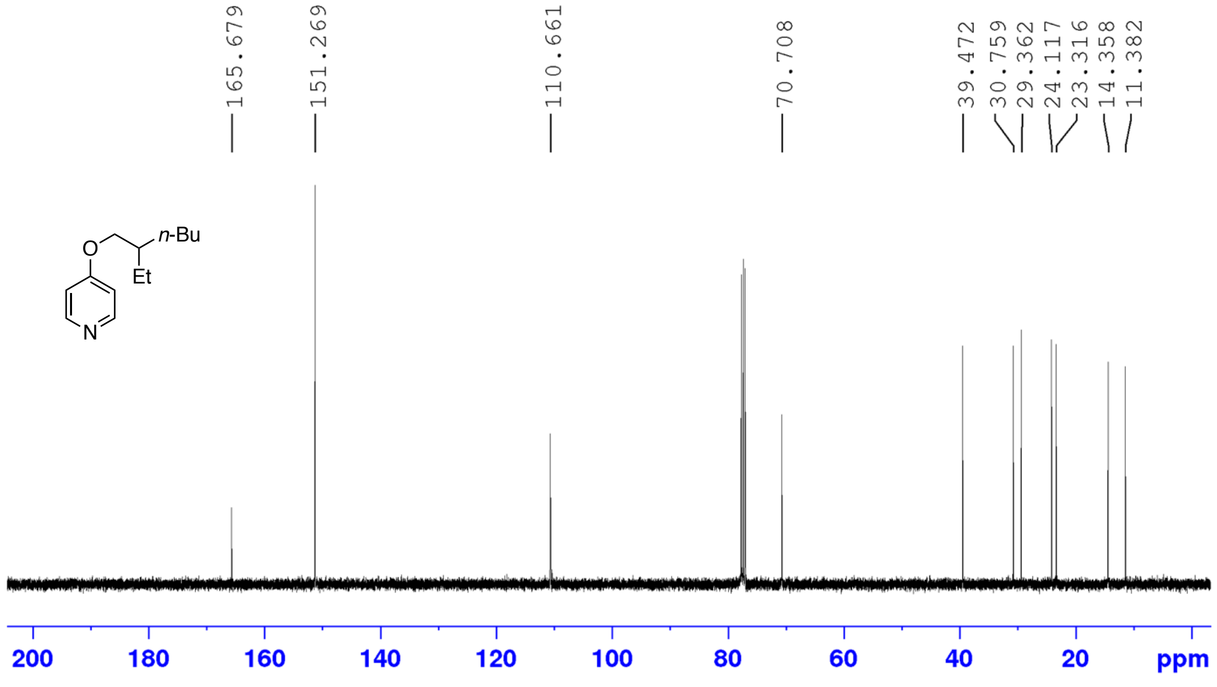

^{13}C NMR spectrum of **10** ($CDCl_3$, 101 MHz)

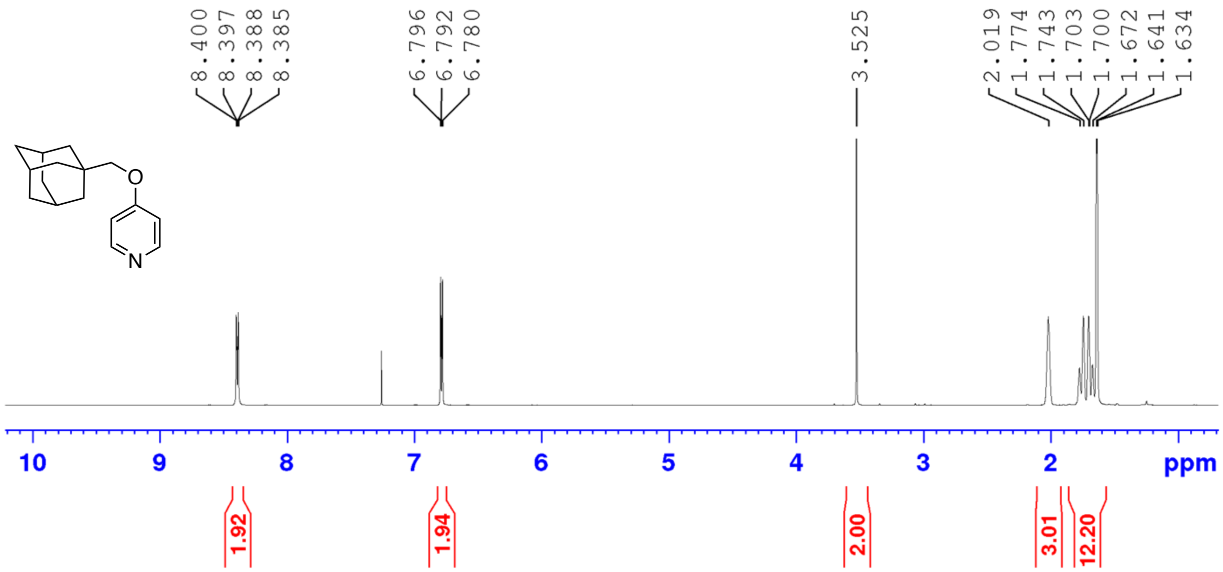

^{1}**H NMR** spectrum of **16** ($CDCl_3$, 400 MHz)

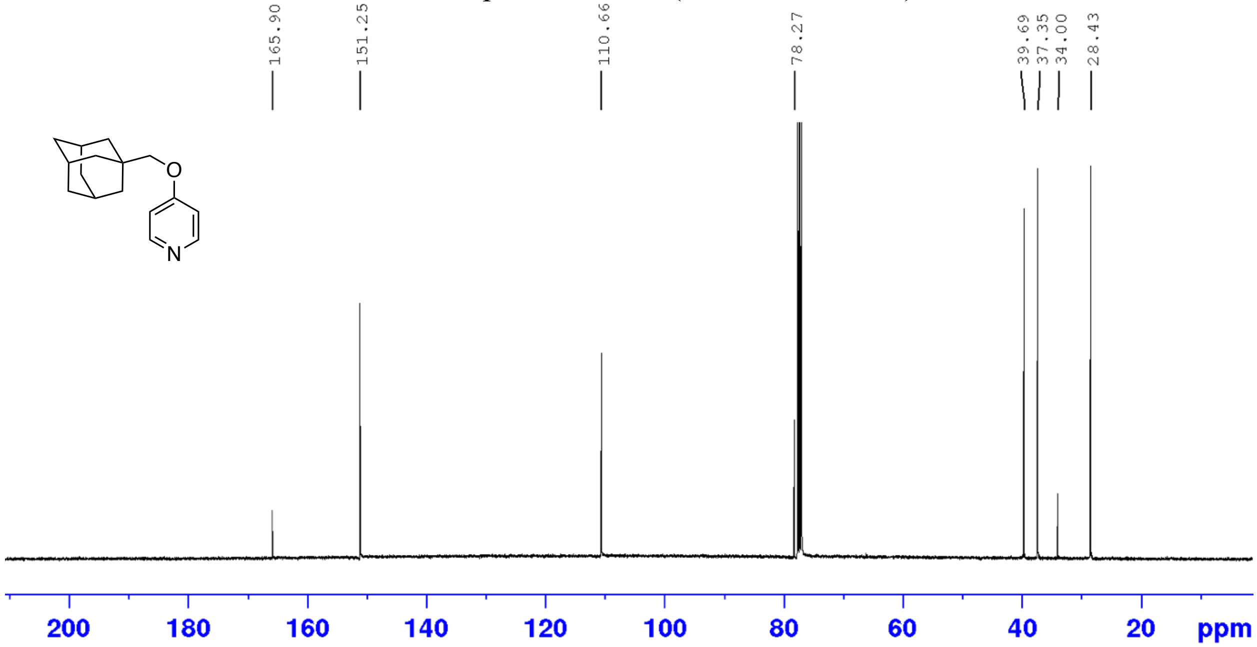

^{13}**C NMR** spectrum of **16** ($CDCl_3$, 101 MHz)

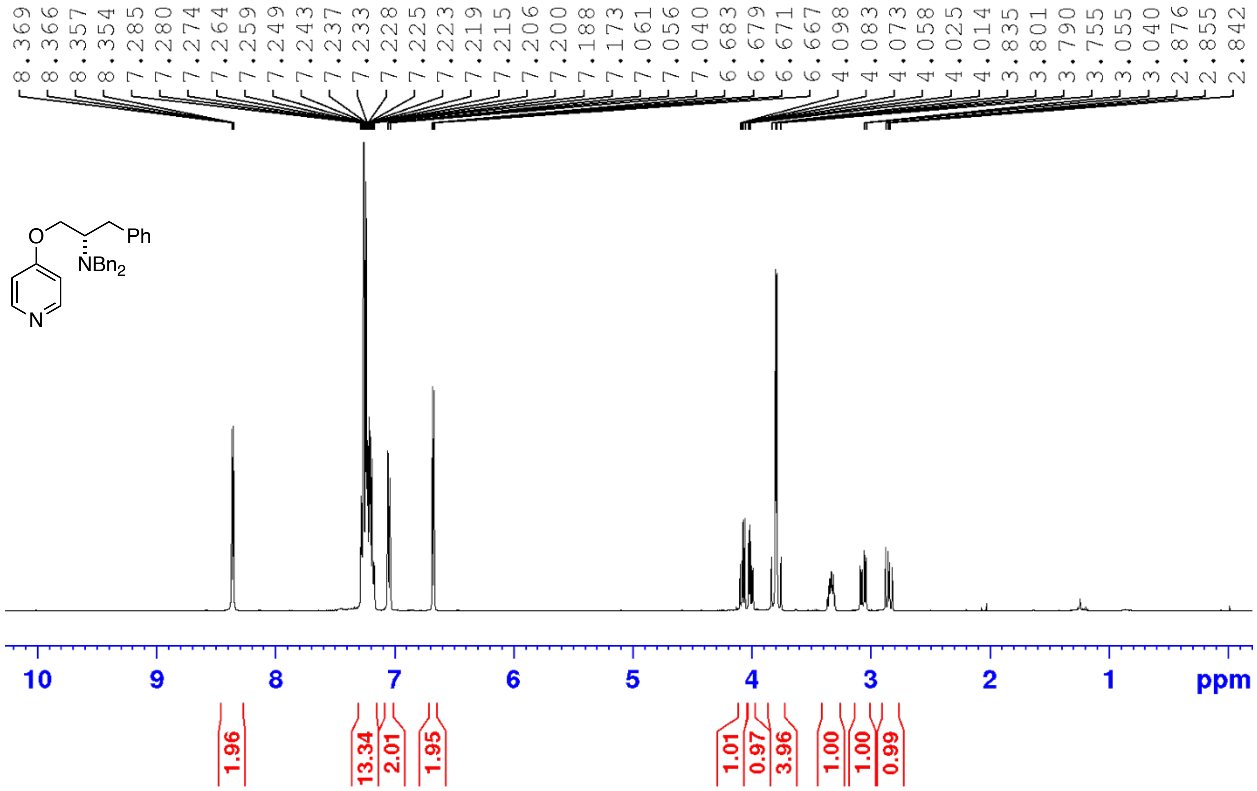

^{1}**H NMR** spectrum of **17** ($CDCl_3$, 400 MHz)

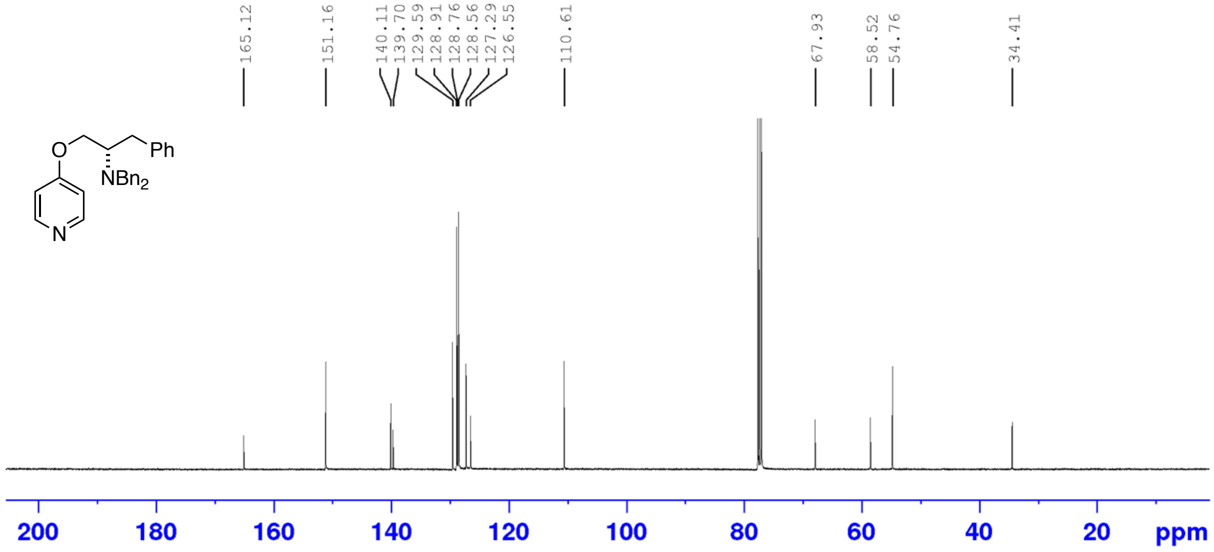

^{13}**C NMR** spectrum of **17** ($CDCl_3$, 101 MHz)

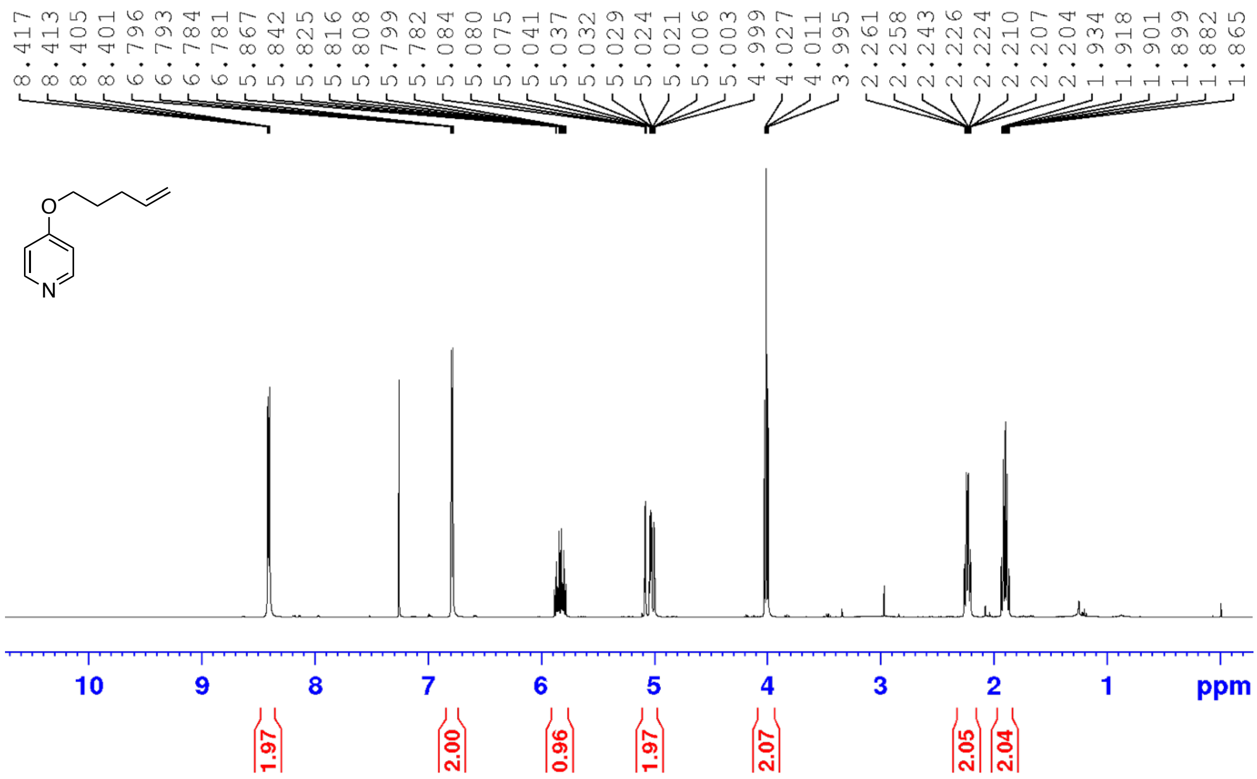

^{1}H NMR spectrum of **18** ($CDCl_3$, 400 MHz)

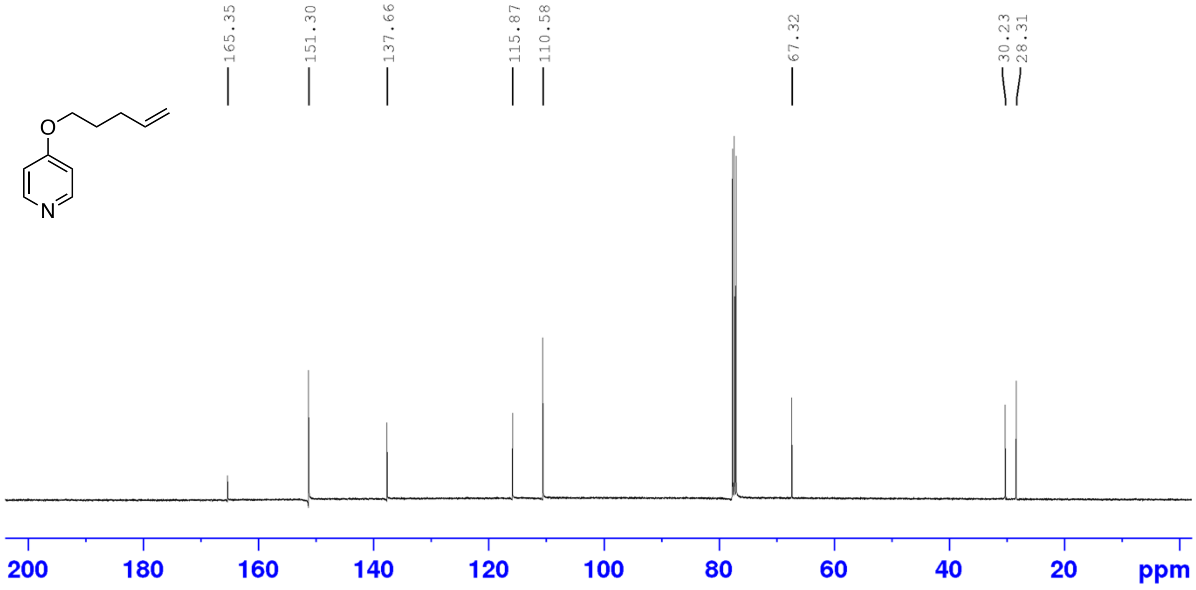

^{13}C NMR spectrum of **18** ($CDCl_3$, 101 MHz)

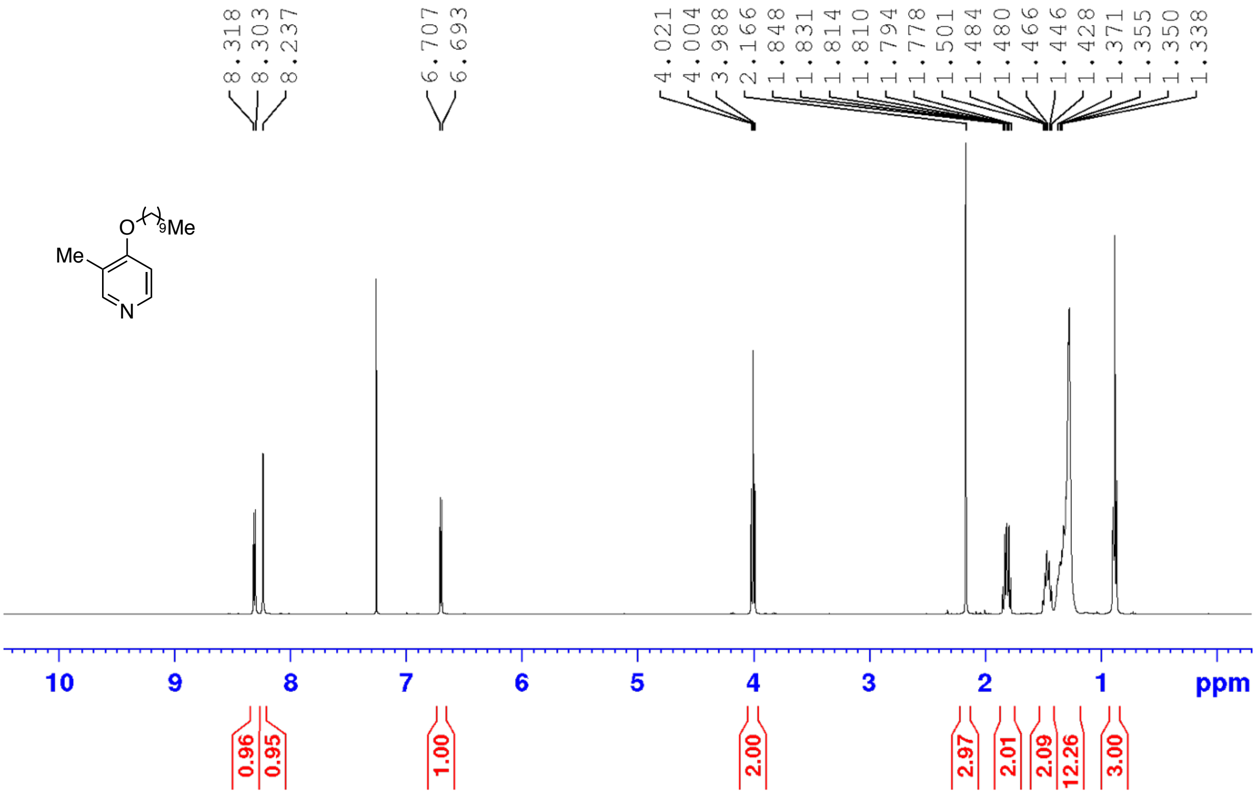

^{1}H NMR spectrum of 19 ($CDCl_3$, 400 MHz)

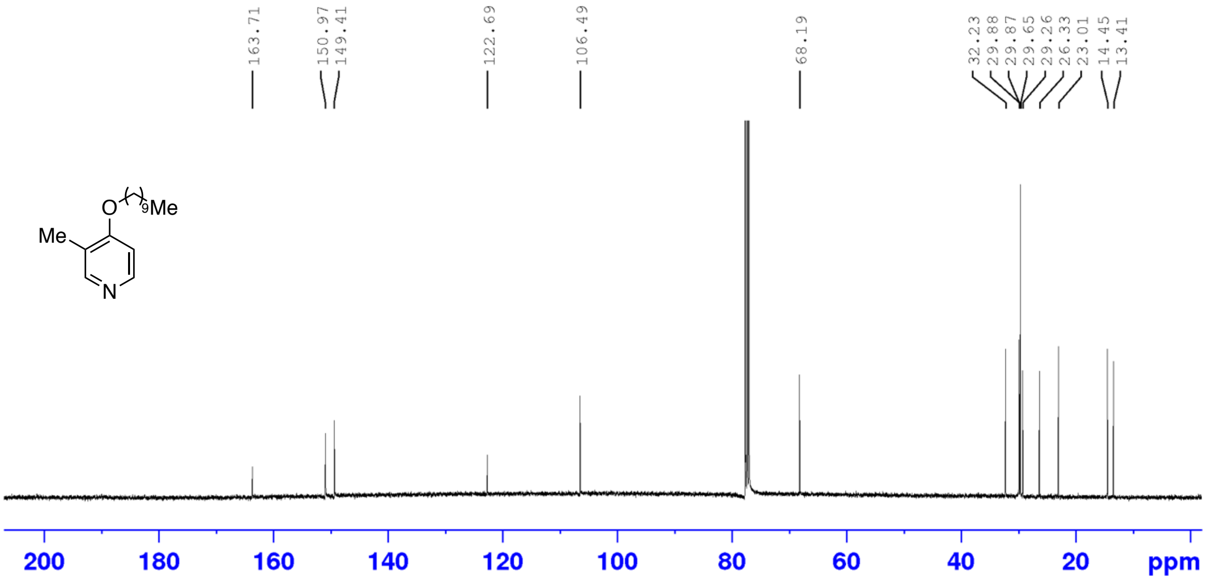

^{13}C NMR spectrum of 19 ($CDCl_3$, 101 MHz)

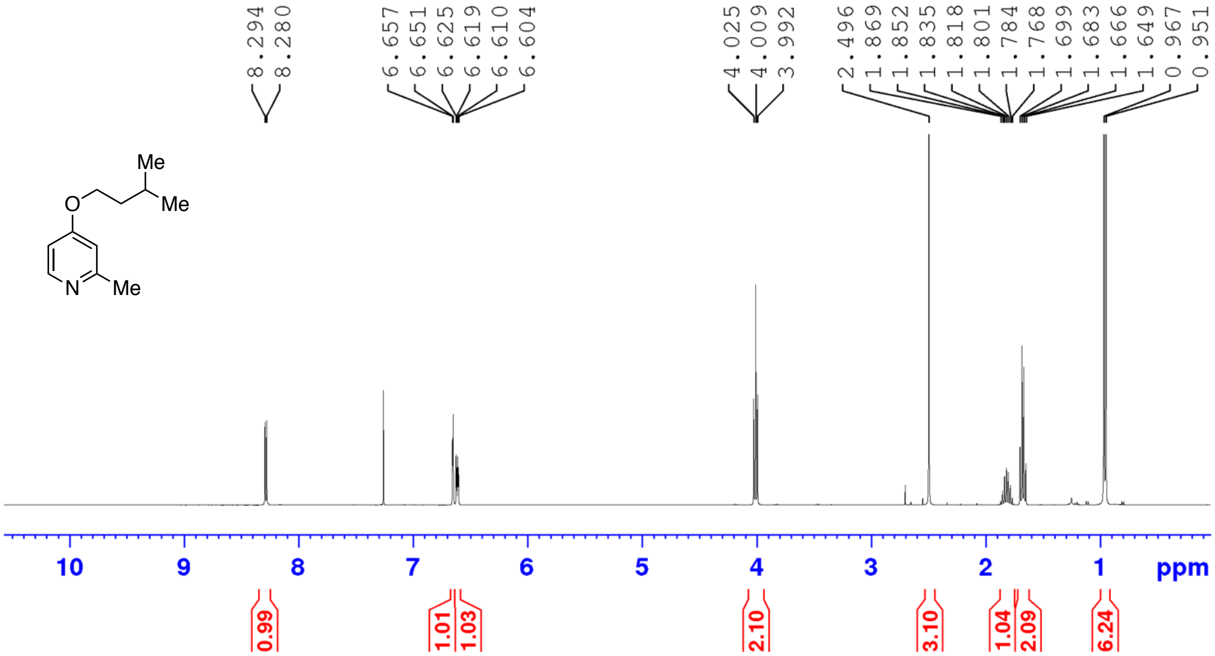

^{1}H NMR spectrum of **20** ($CDCl_3$, 400 MHz)

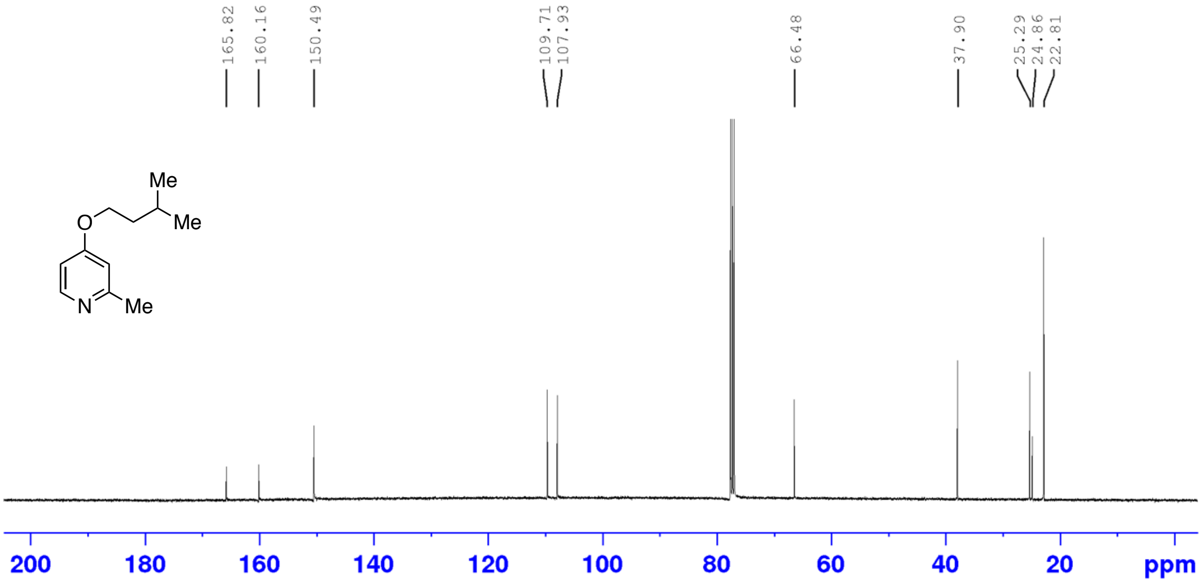

^{13}C NMR spectrum of **20** ($CDCl_3$, 101 MHz)

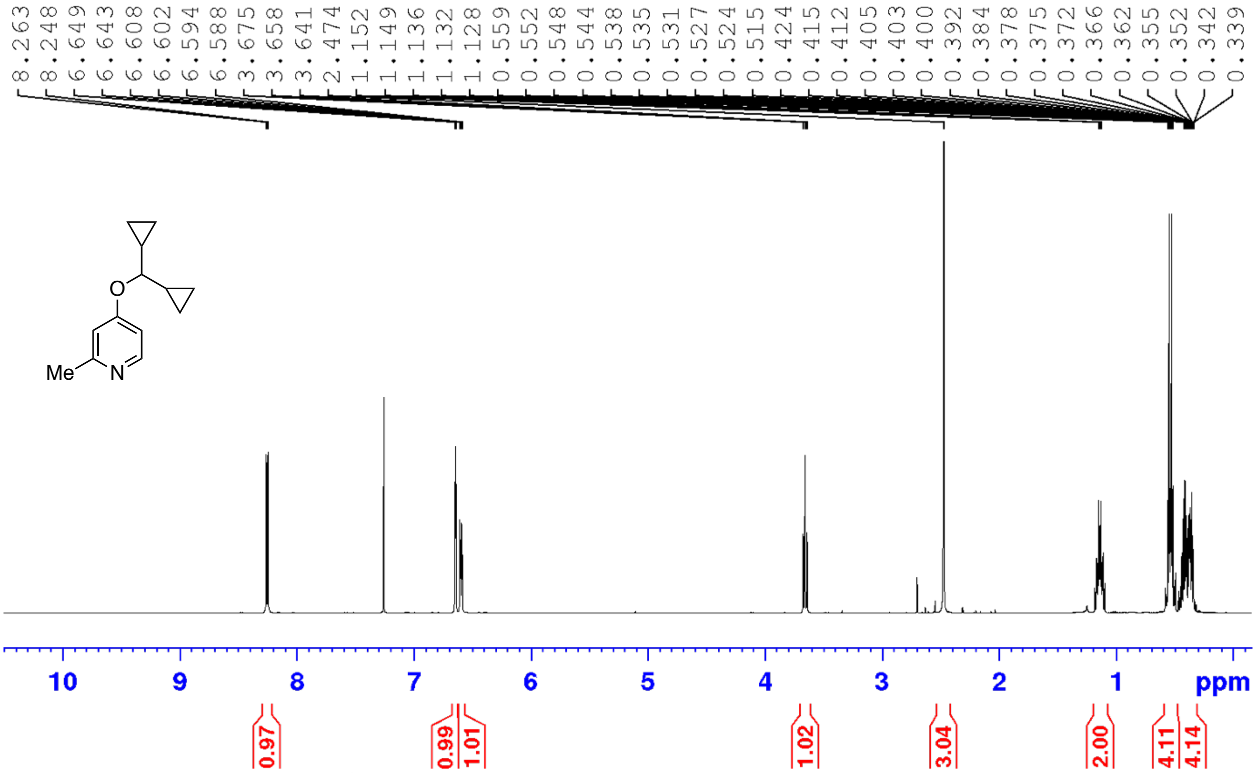

[1]**H NMR** spectrum of **21** (CDCl3, 400 MHz)

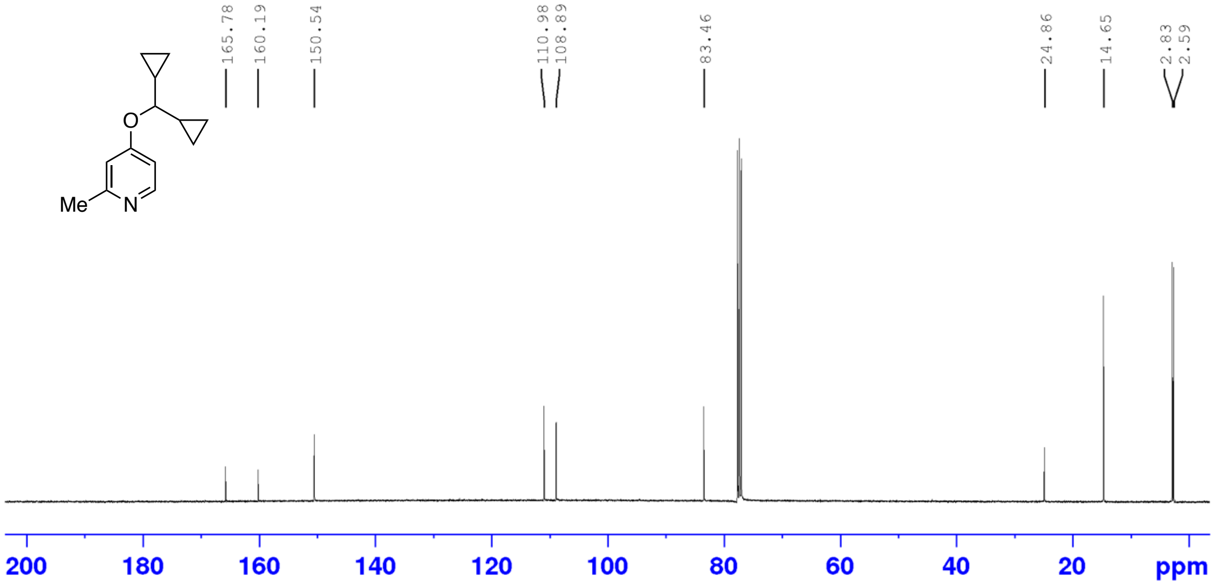

[13]**C NMR** spectrum of **21** (CDCl3, 101 MHz)

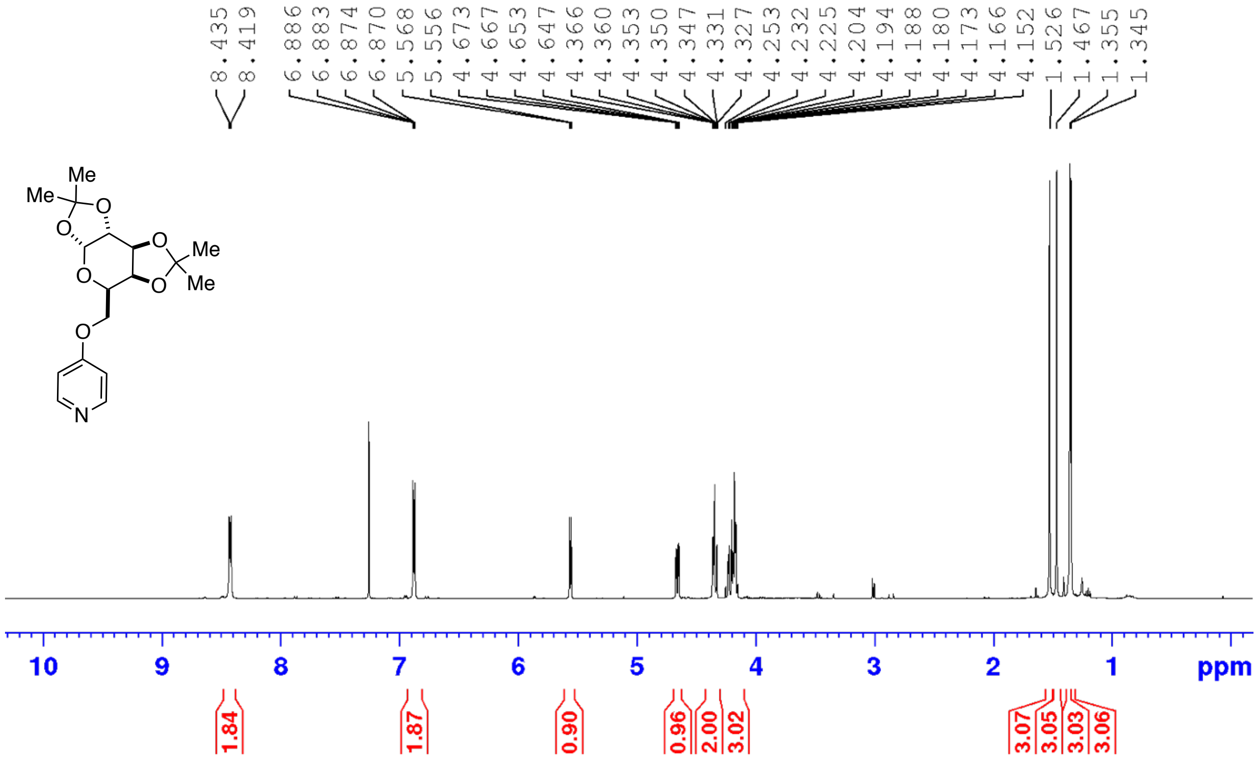

^{1}**H NMR** spectrum of **22** ($CDCl_3$, 400 MHz)

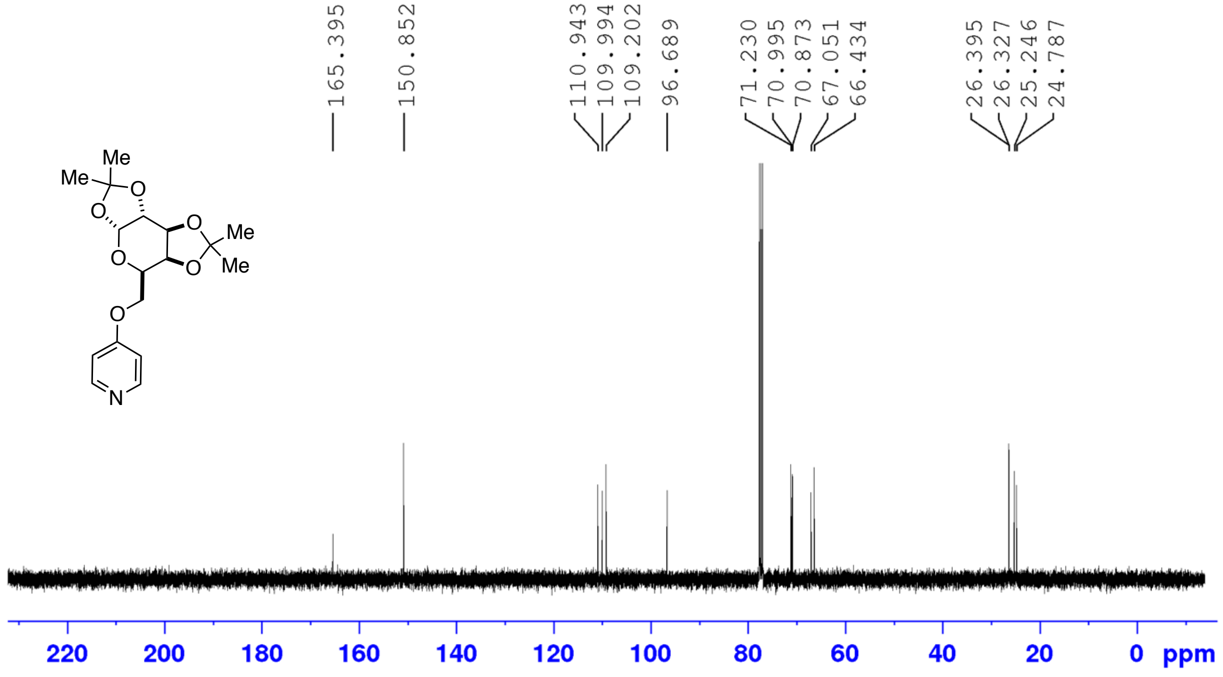

^{13}**C NMR** spectrum of **22** ($CDCl_3$, 101 MHz)

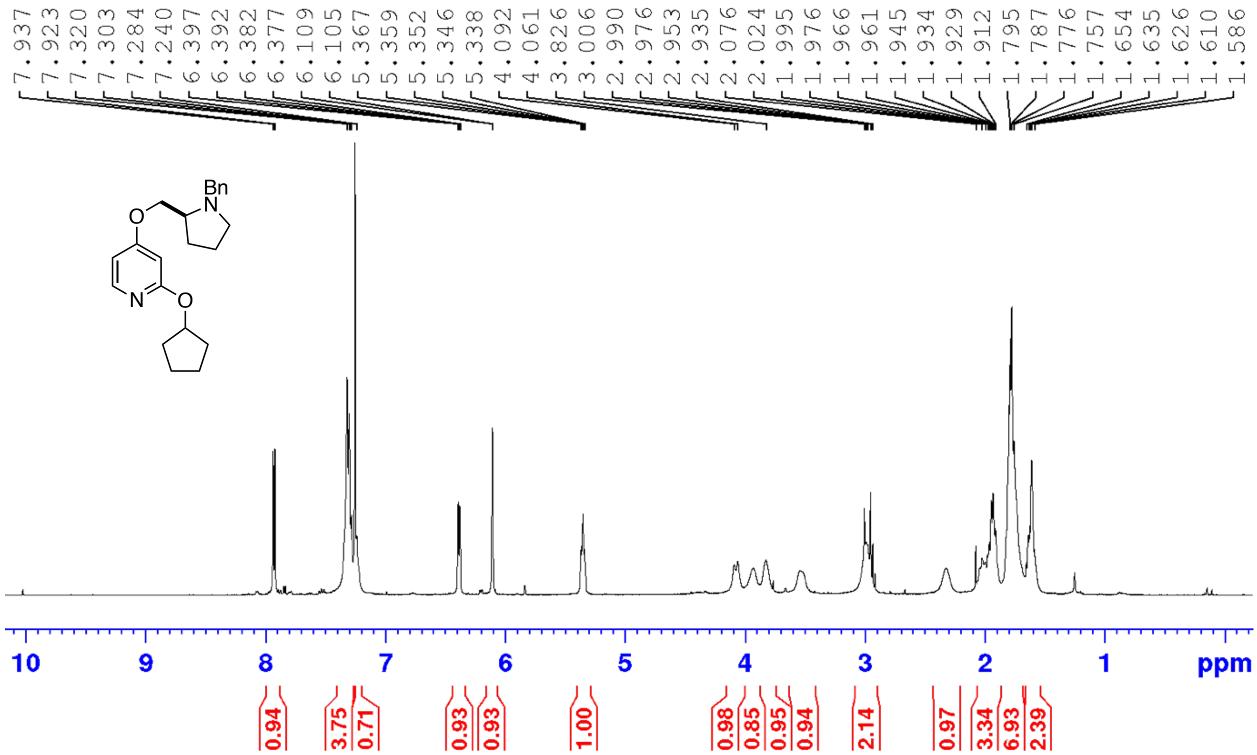

¹H NMR spectrum of **23** ($CDCl_3$, 400 MHz)

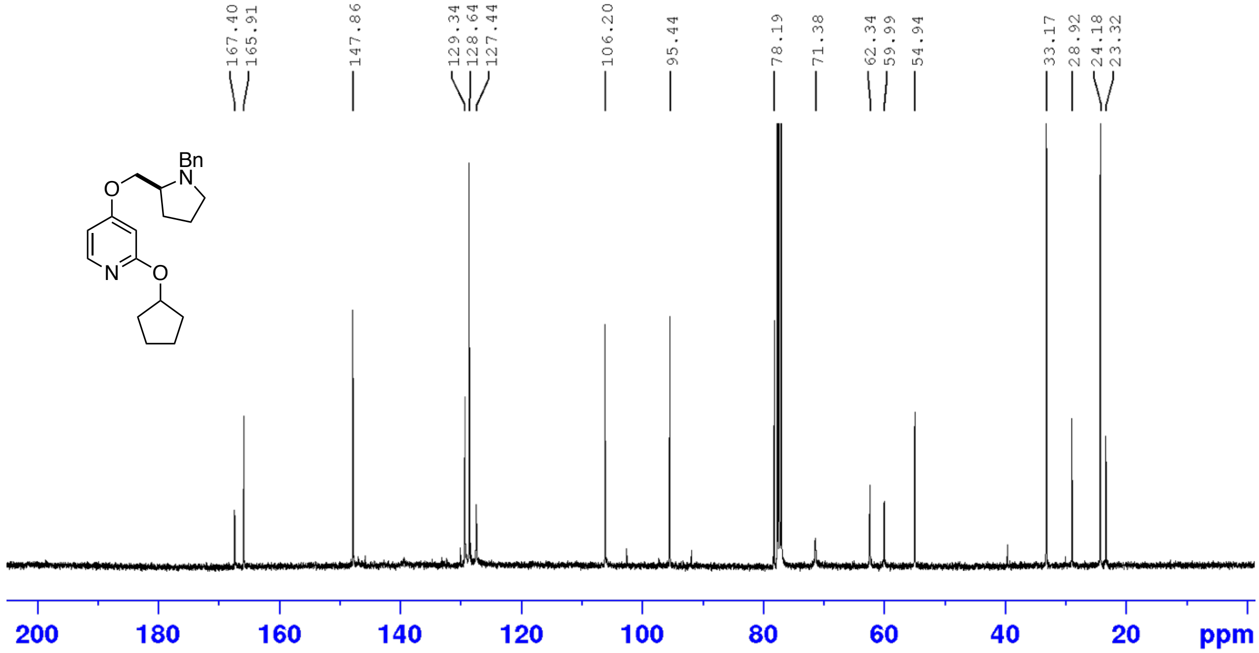

¹³C NMR spectrum of **23** ($CDCl_3$, 101 MHz)

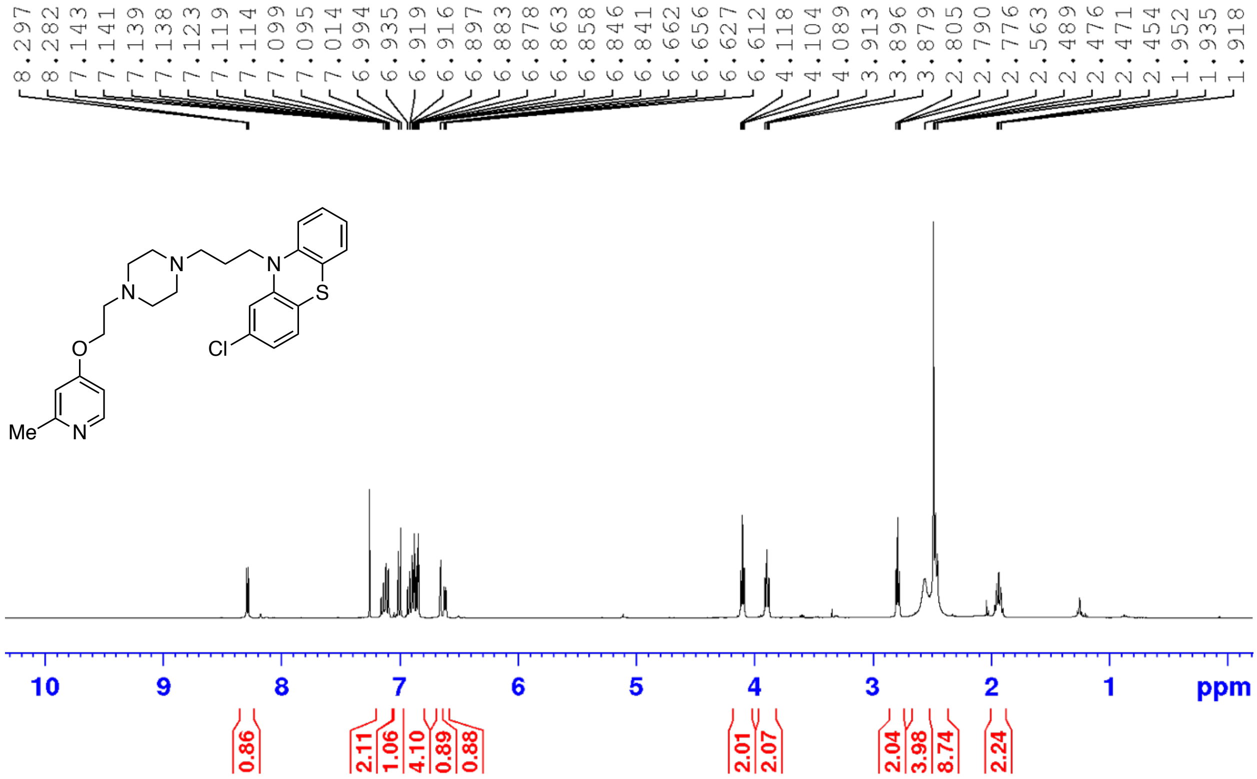

^{1}**H NMR** spectrum of **24** ($CDCl_3$, 400 MHz)

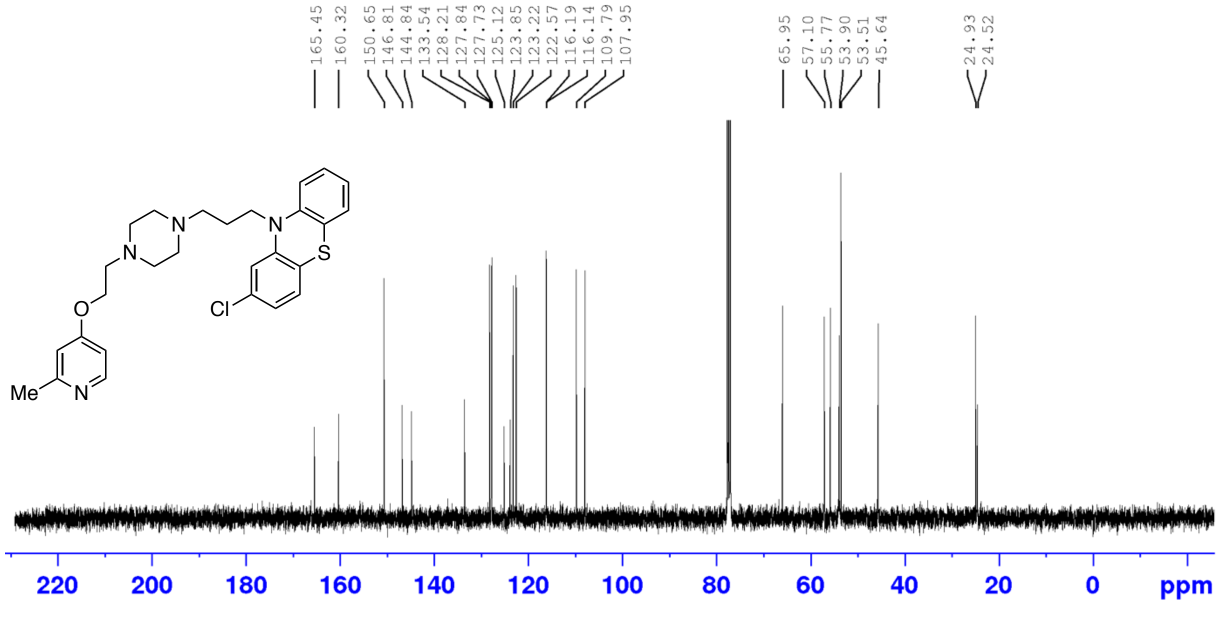

^{13}**C NMR** spectrum of **24** ($CDCl_3$, 101 MHz)

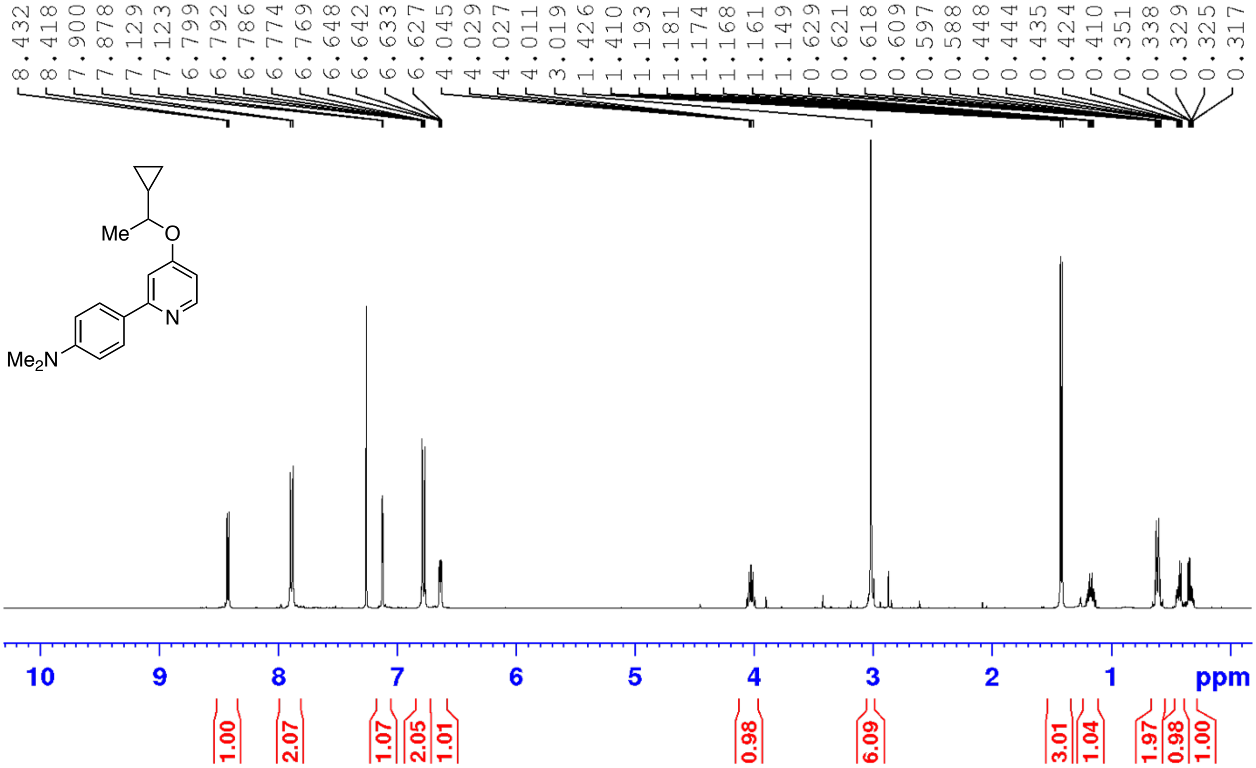

^{1}**H NMR** spectrum of **25** (400 MHz, $CDCl_3$)

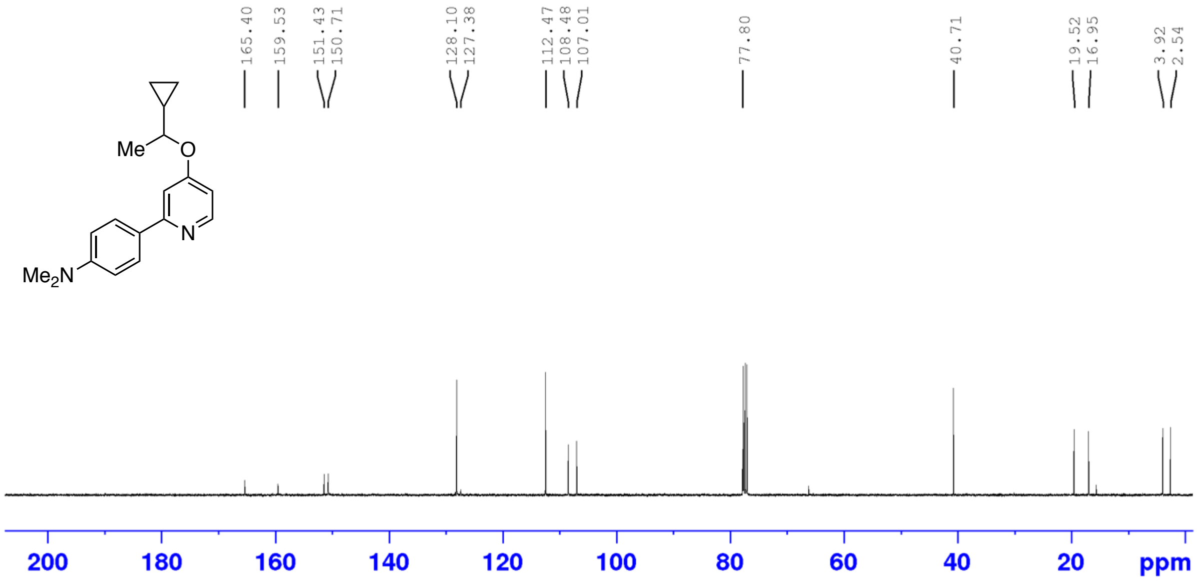

^{13}**C NMR** spectrum of **25** (101 MHz, $CDCl_3$)

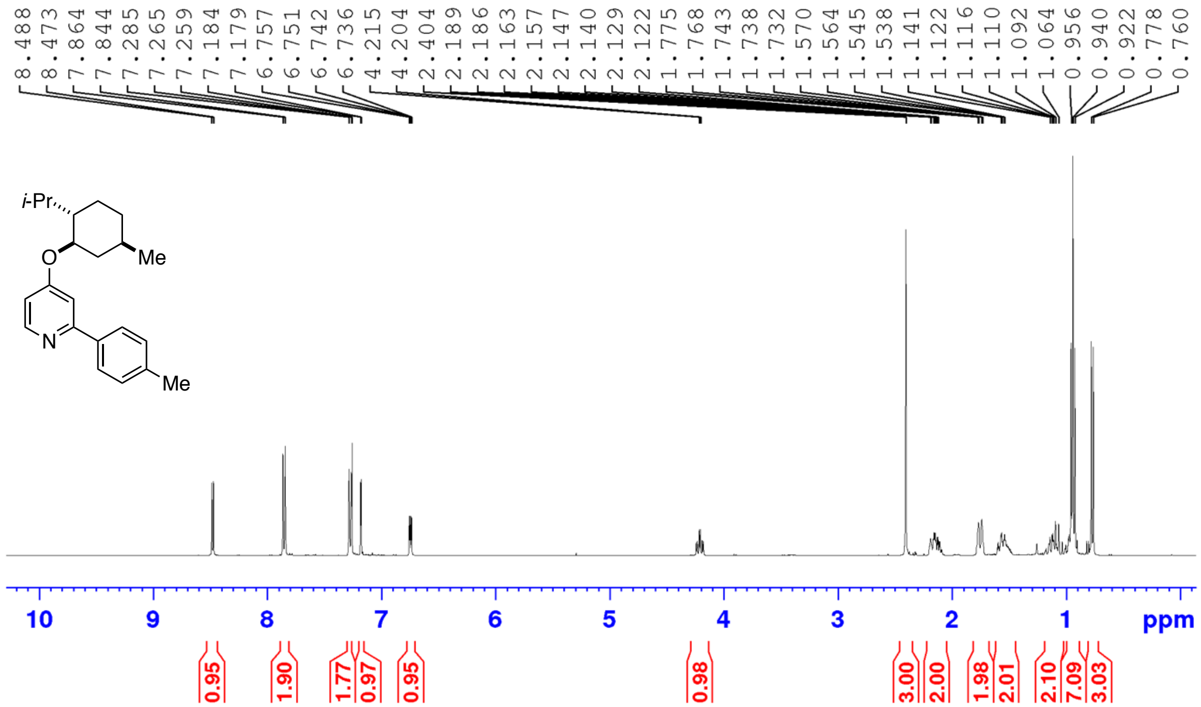

^{1}H NMR spectrum of **26** ($CDCl_3$, 400 MHz)

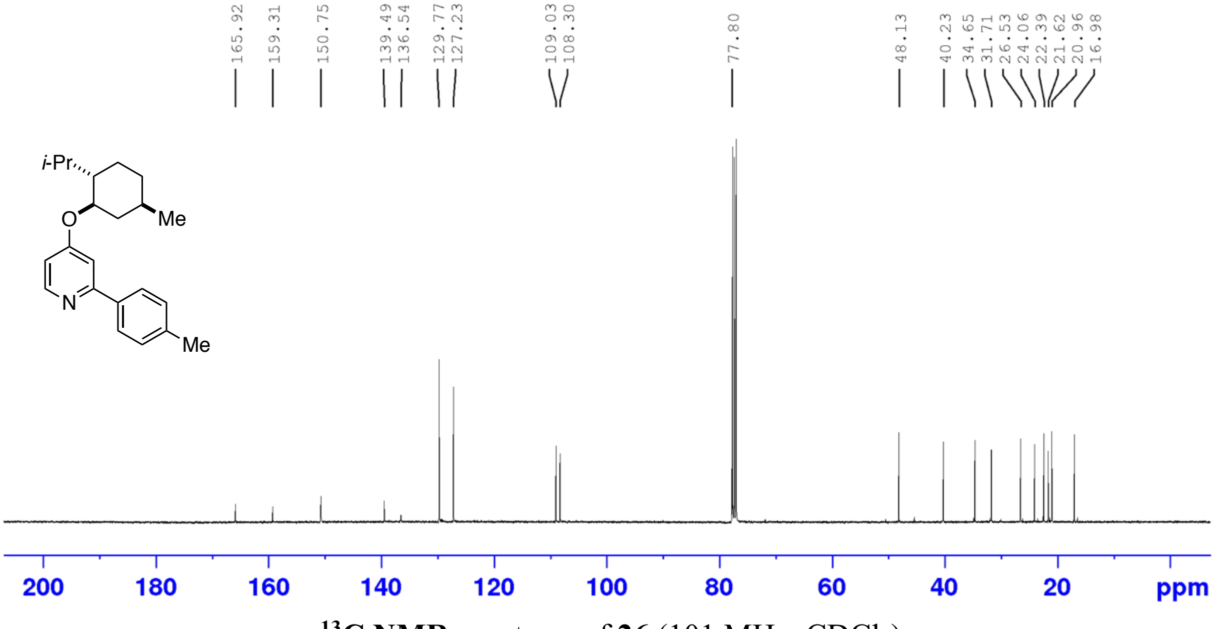

^{13}C NMR spectrum of **26** (101 MHz, $CDCl_3$)

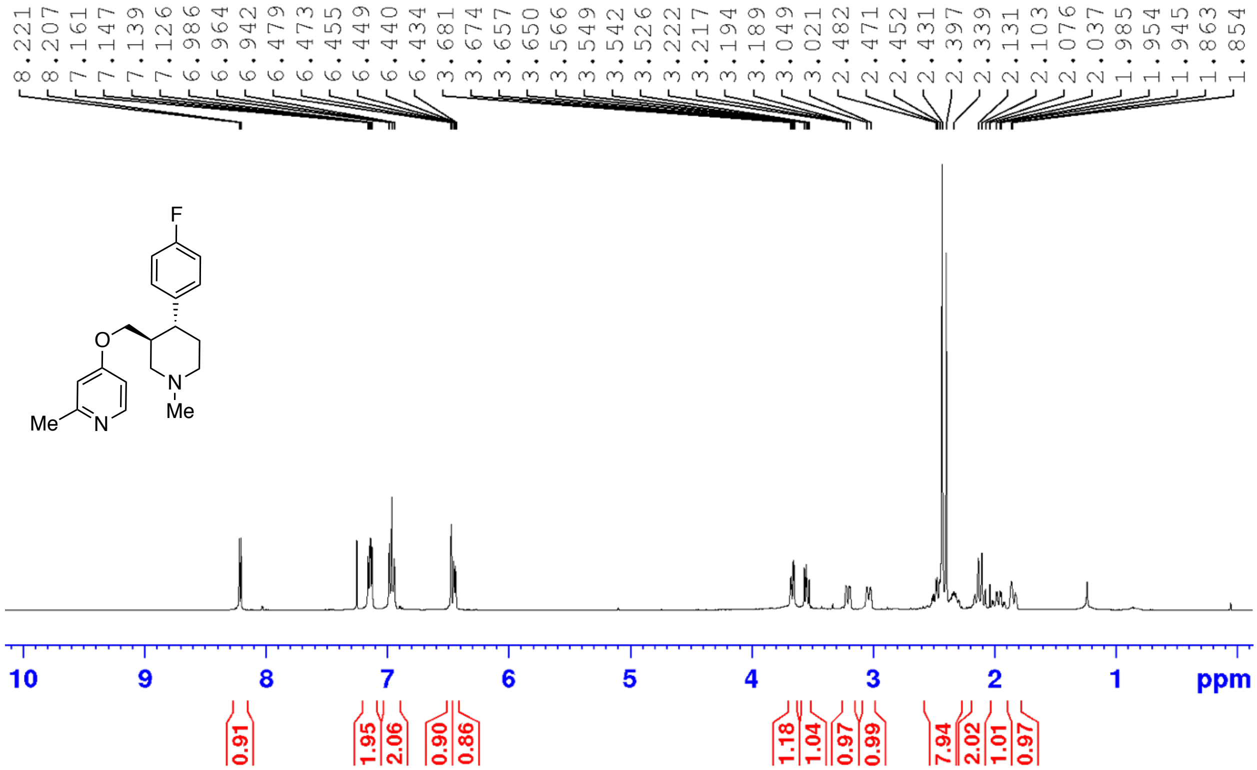

^{1}H NMR spectrum of **27** ($CDCl_3$, 400 MHz)

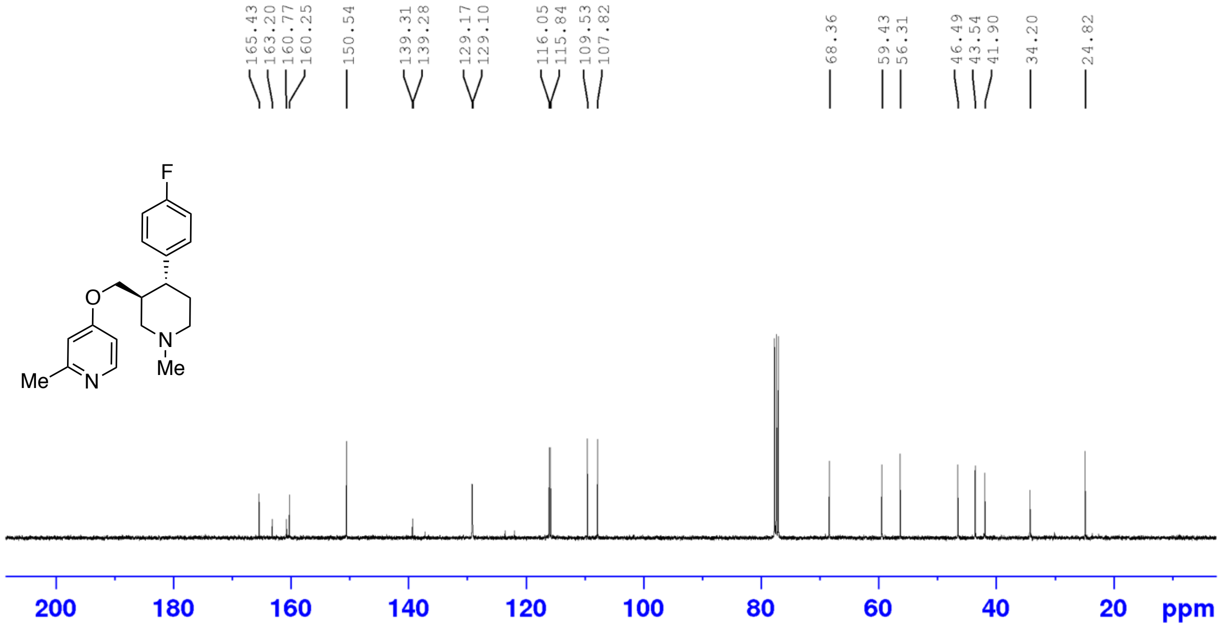

^{13}C NMR spectrum of **27** ($CDCl_3$, 101 MHz)

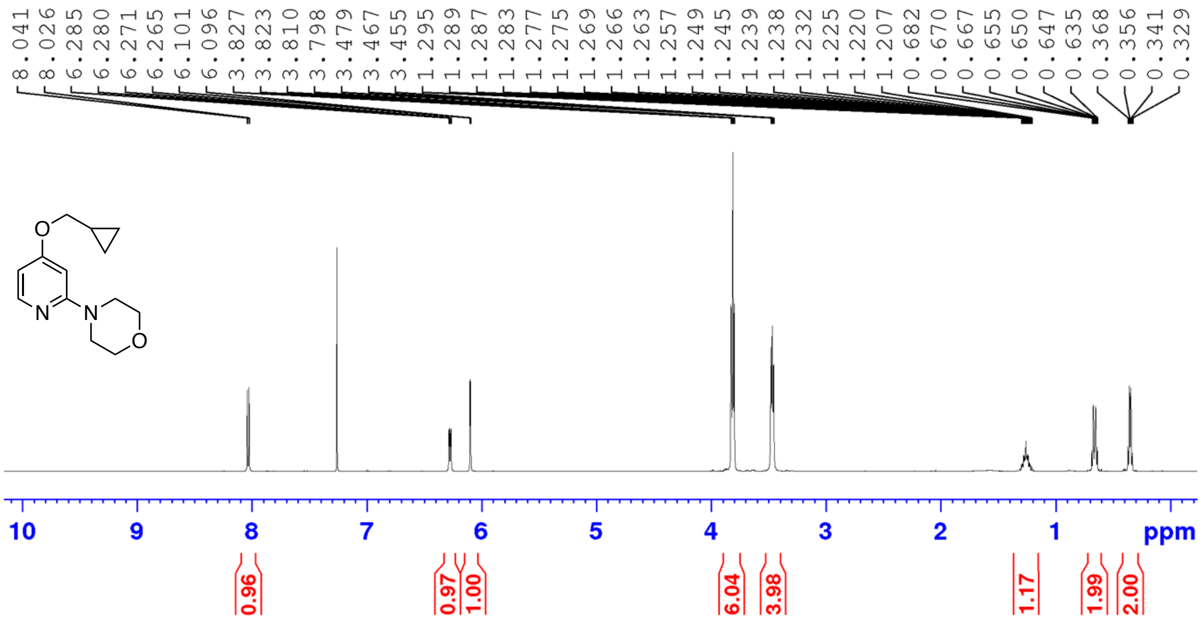

[1]**H NMR** spectrum of **28** ($CDCl_3$, 400 MHz)

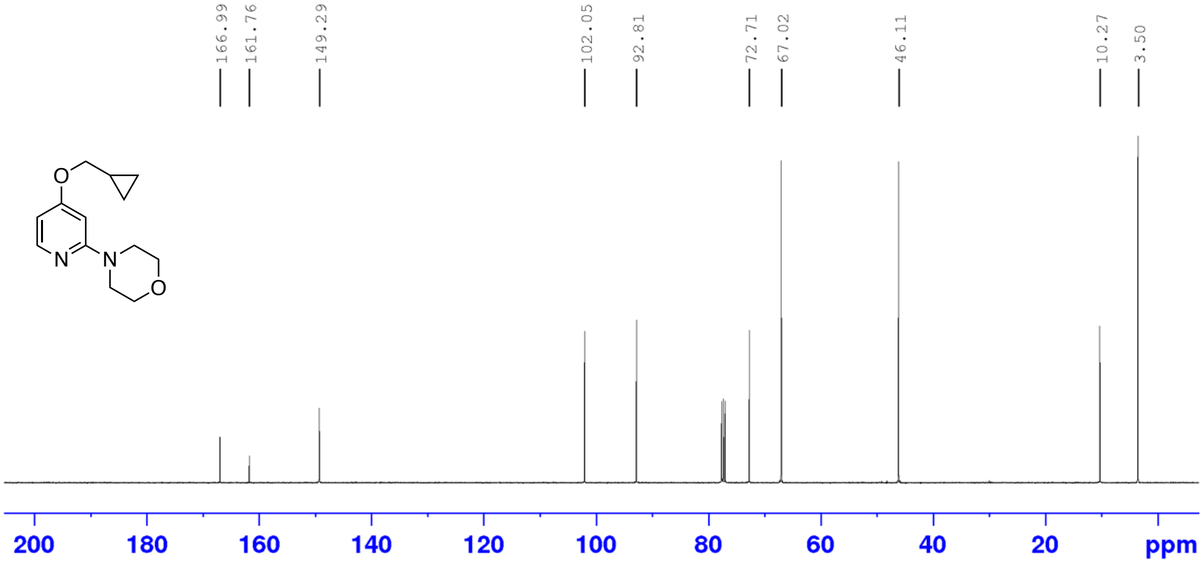

[13]**C NMR** spectrum of **28** ($CDCl_3$, 101 MHz)

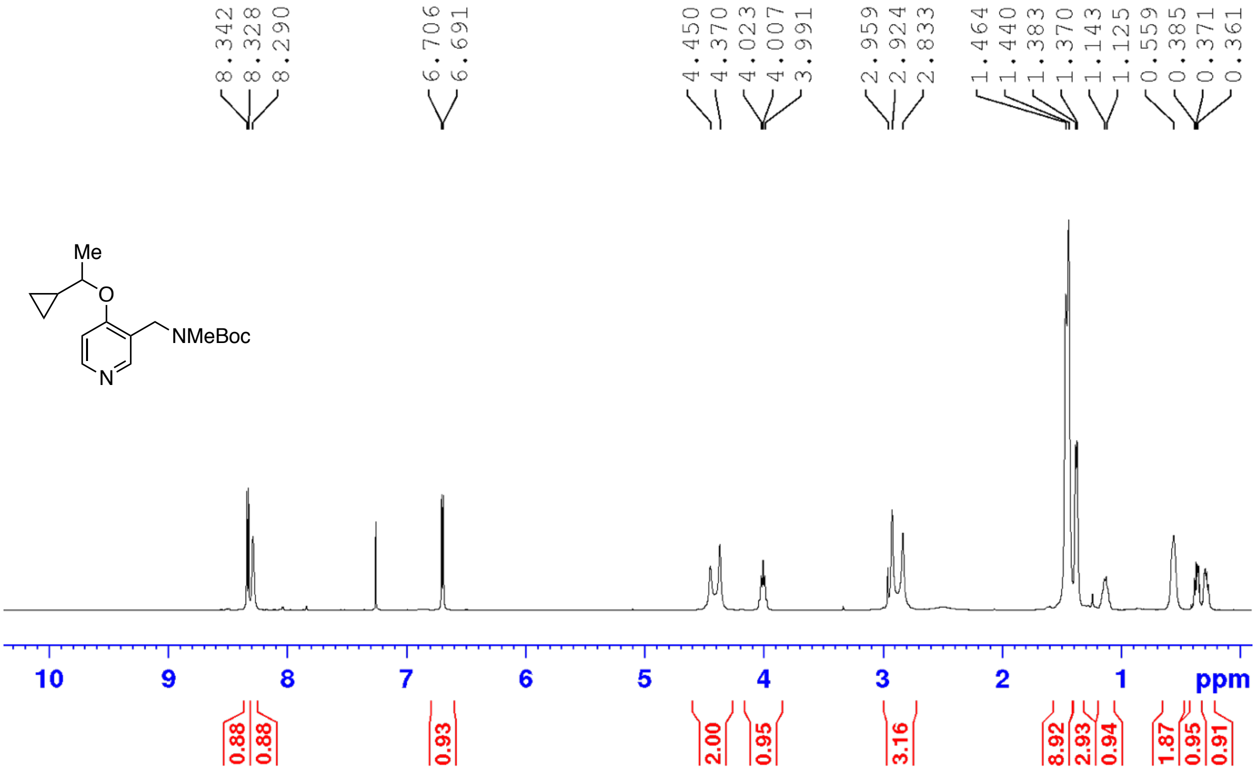

[1]**H NMR** spectrum of **29** ($CDCl_3$, 400 MHz)

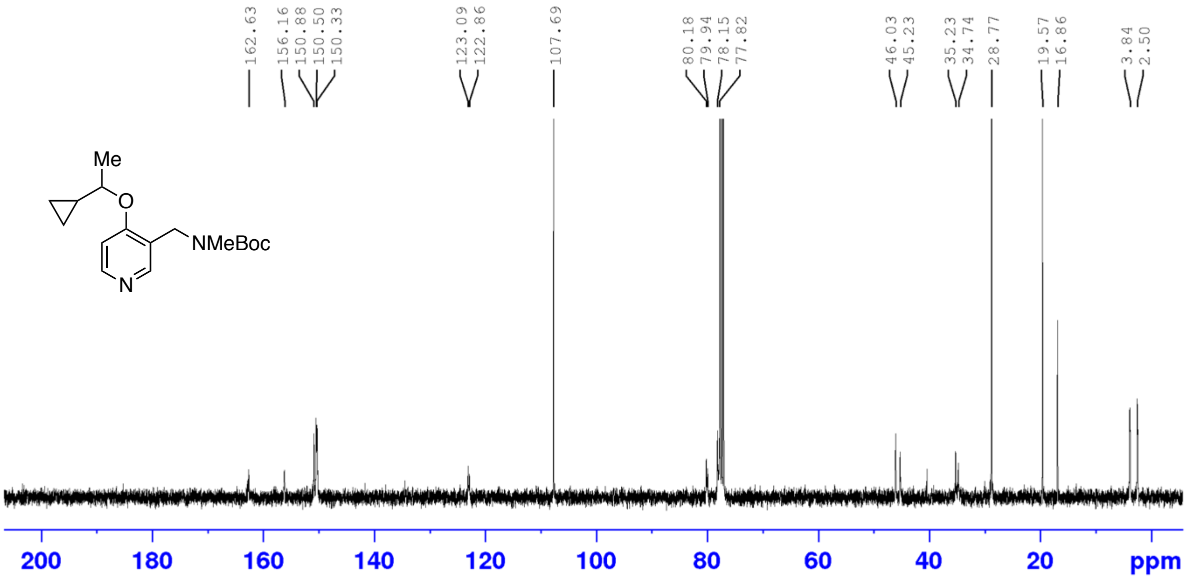

[13]**C NMR** spectrum of **29** ($CDCl_3$, 101 MHz)

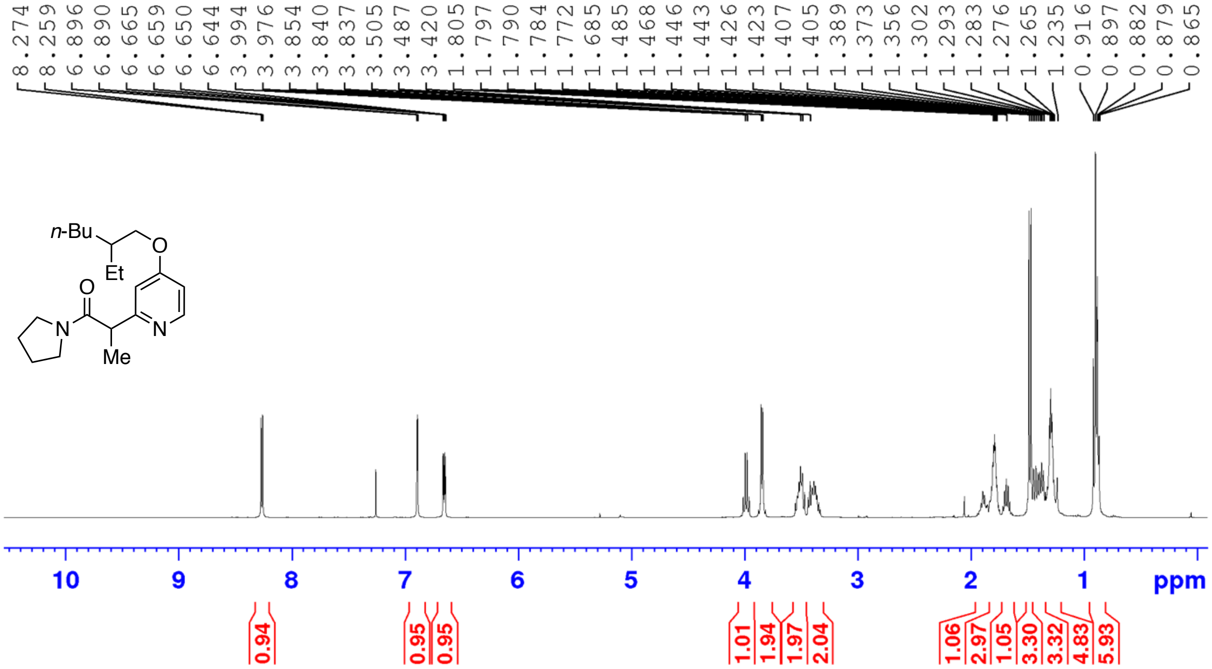

^{1}H NMR spectrum of **30** ($CDCl_3$, 400 MHz)

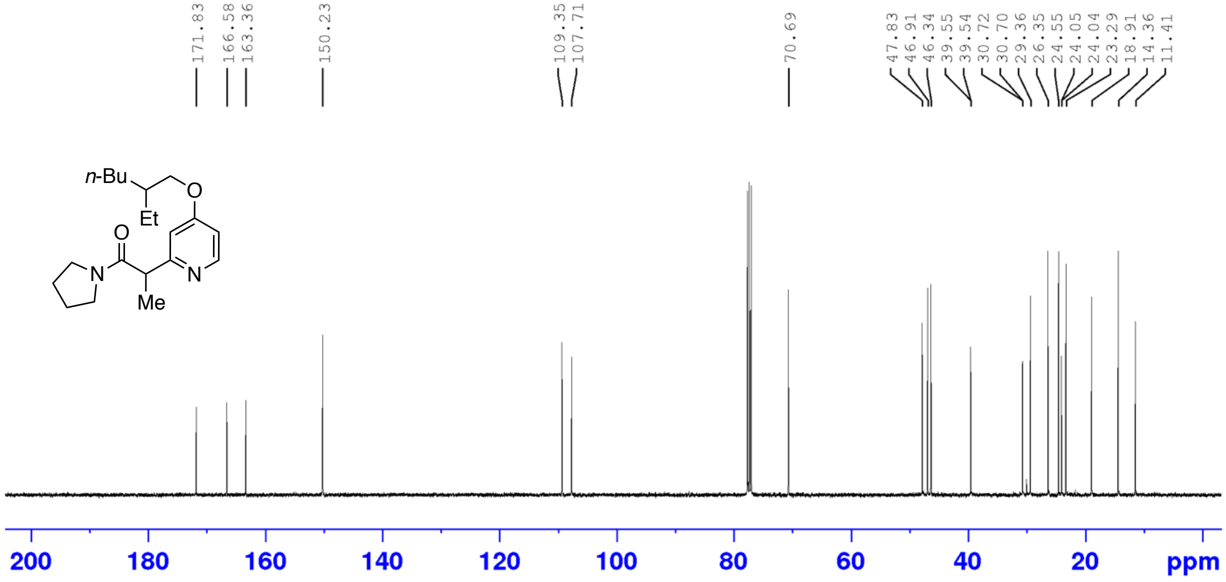

^{13}C NMR spectrum of **30** ($CDCl_3$, 101 MHz)

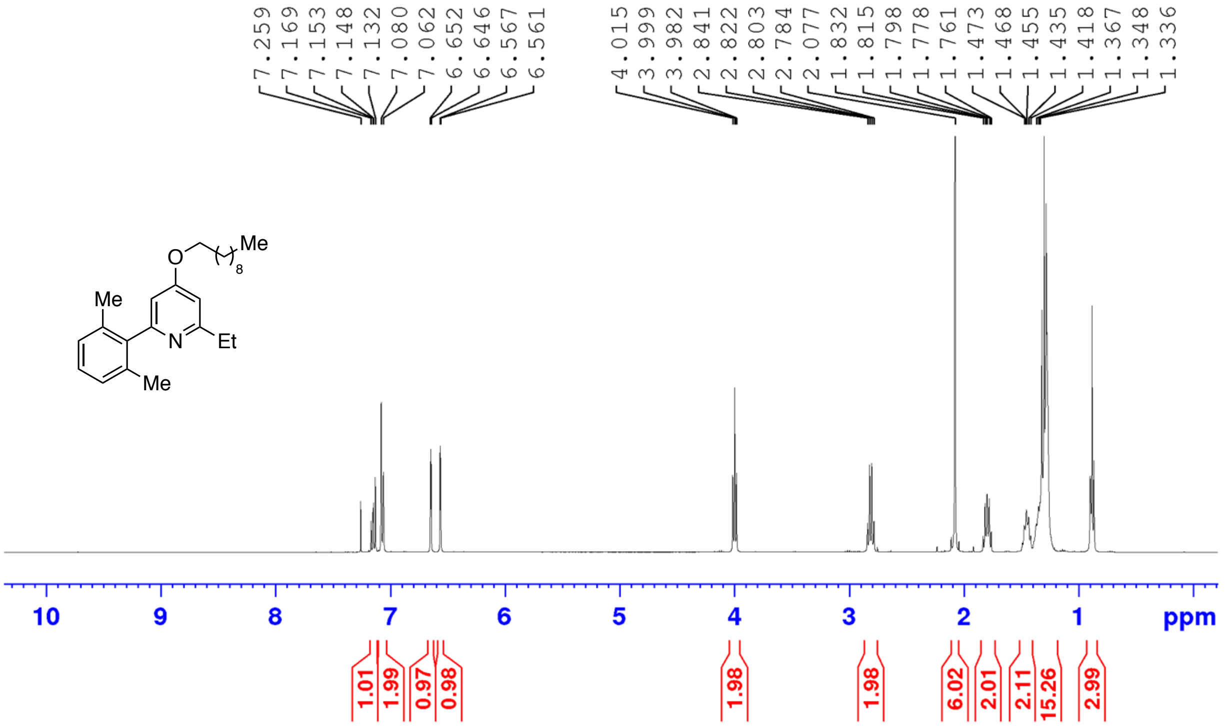

^{1}**H NMR** spectrum of **31** (400 MHz, $CDCl_3$)

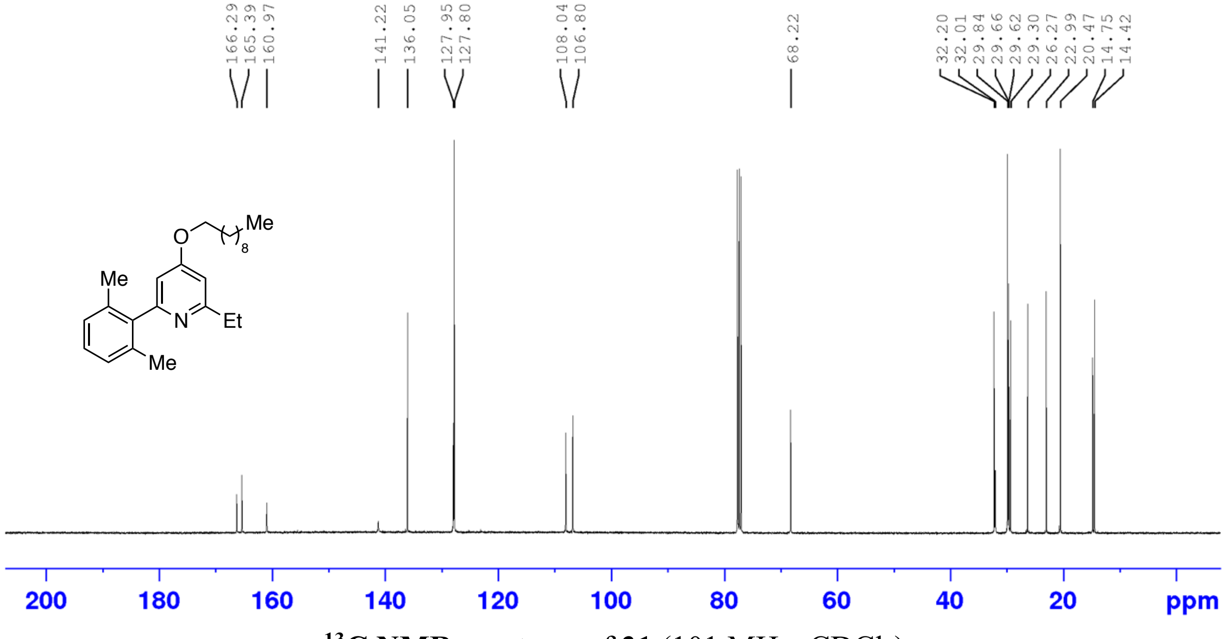

^{13}**C NMR** spectrum of **31** (101 MHz, $CDCl_3$)

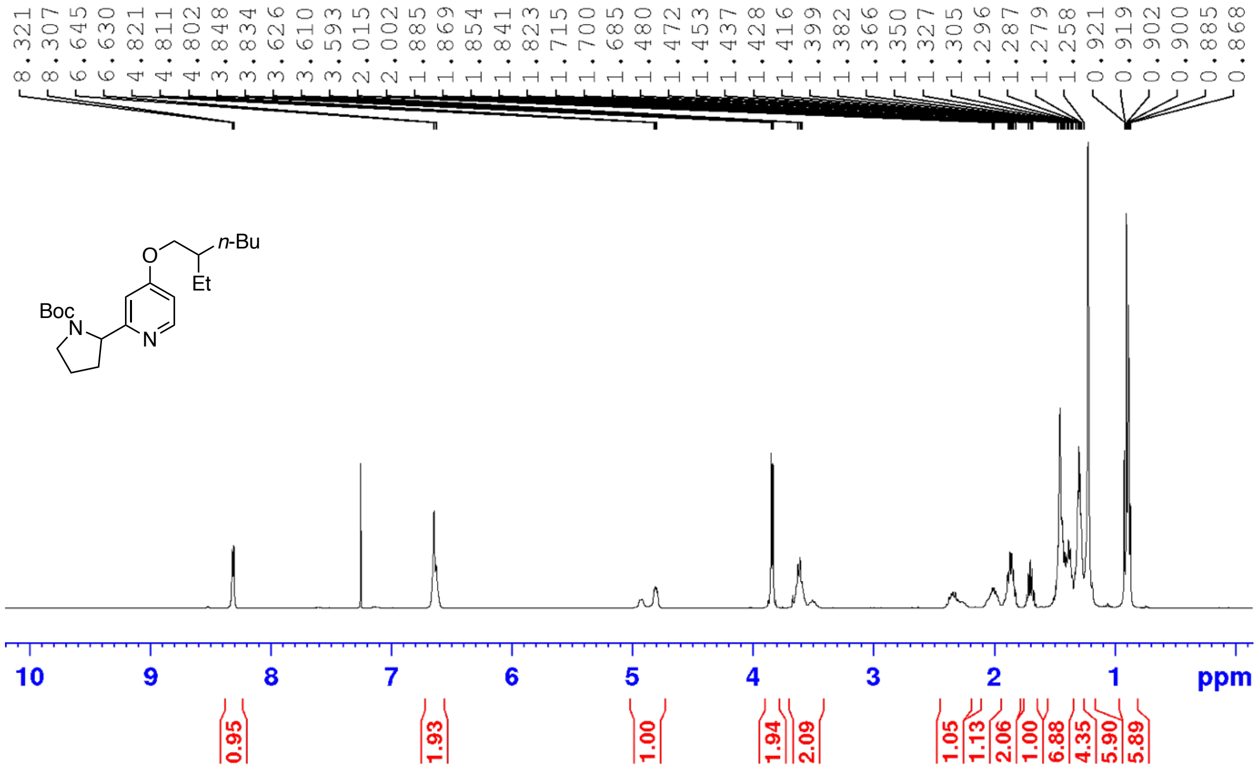

^{1}H NMR spectrum of **32** ($CDCl_3$, 400 MHz)

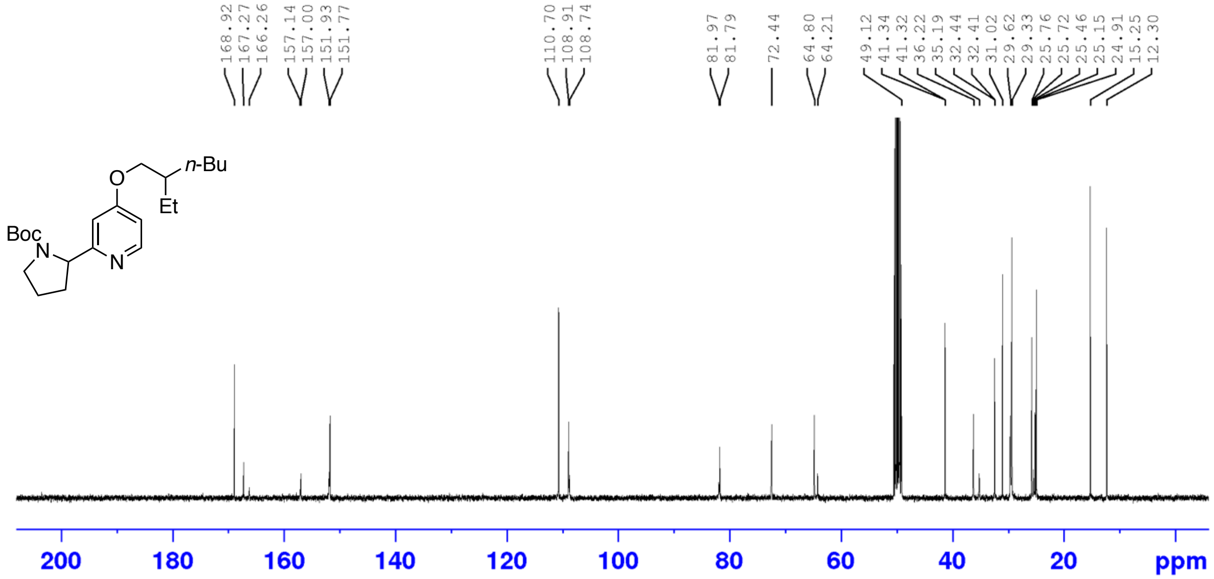

^{13}C NMR spectrum of **32** (CD_3OD, 101 MHz)

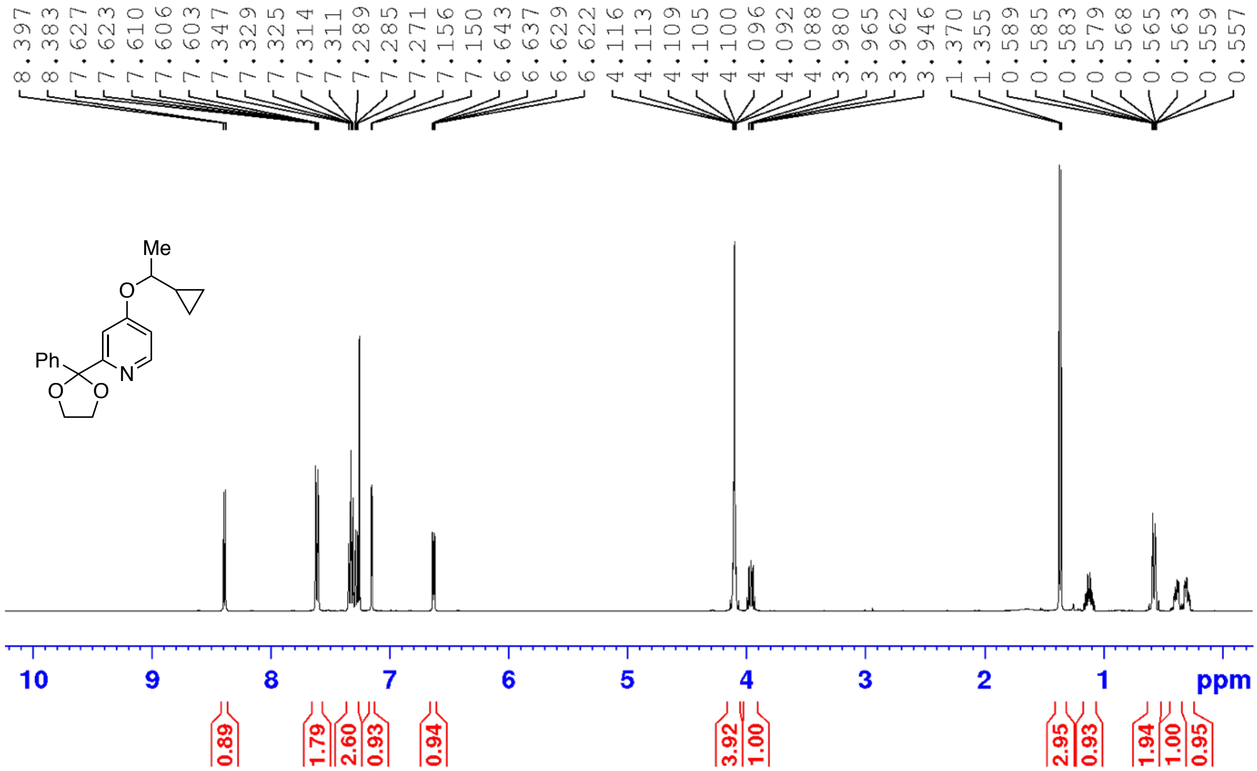

[1]**H NMR** spectrum of **33** ($CDCl_3$, 400 MHz)

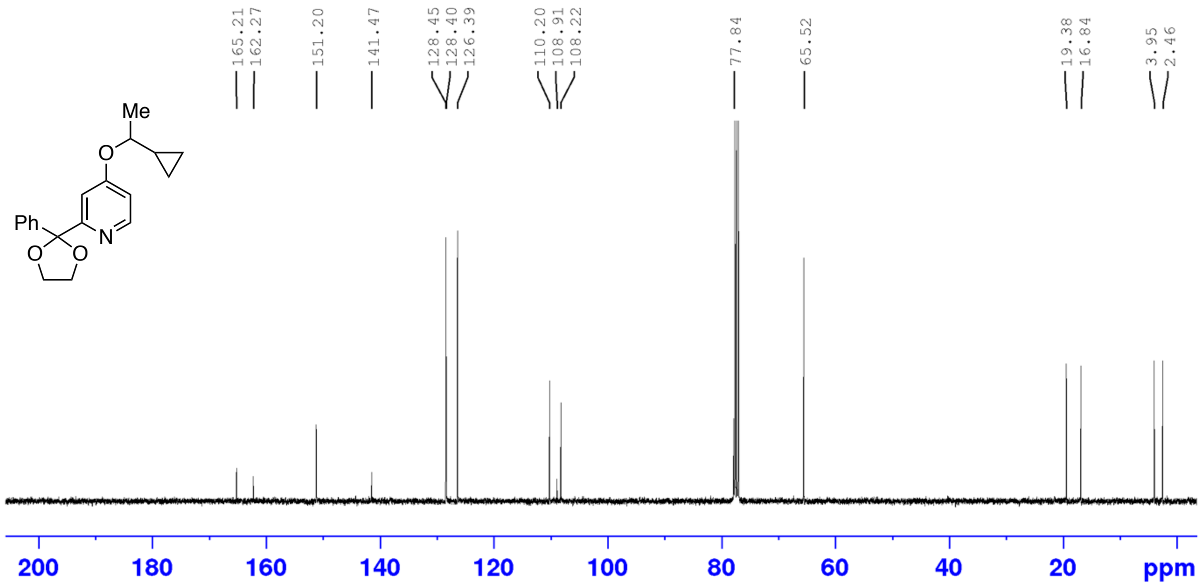

[13]**C NMR** spectrum of **33** ($CDCl_3$, 101 MHz)

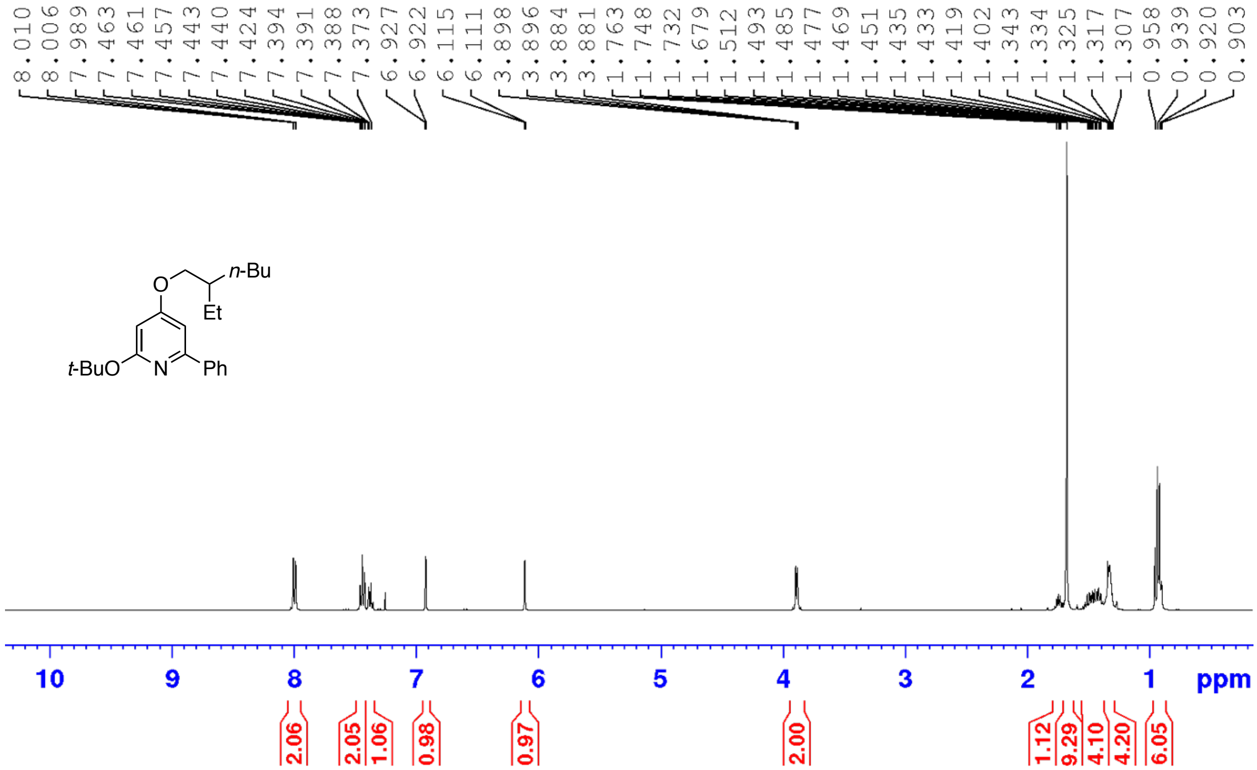

¹H NMR spectrum of **36** ($CDCl_3$, 400 MHz)

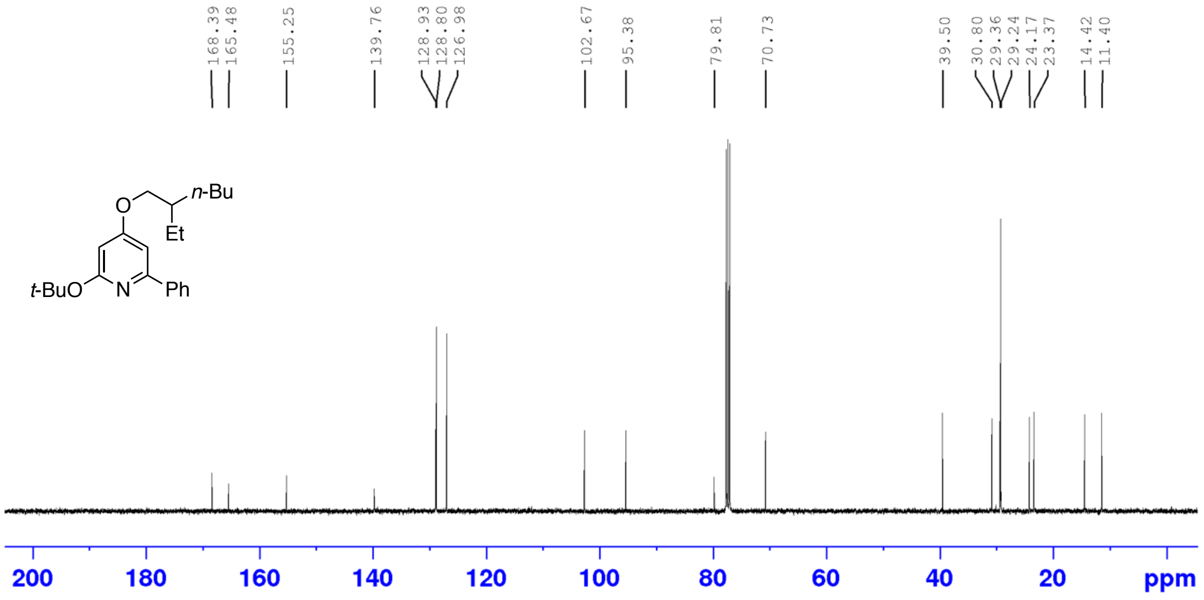

¹³C NMR spectrum of **36** ($CDCl_3$, 101 MHz)

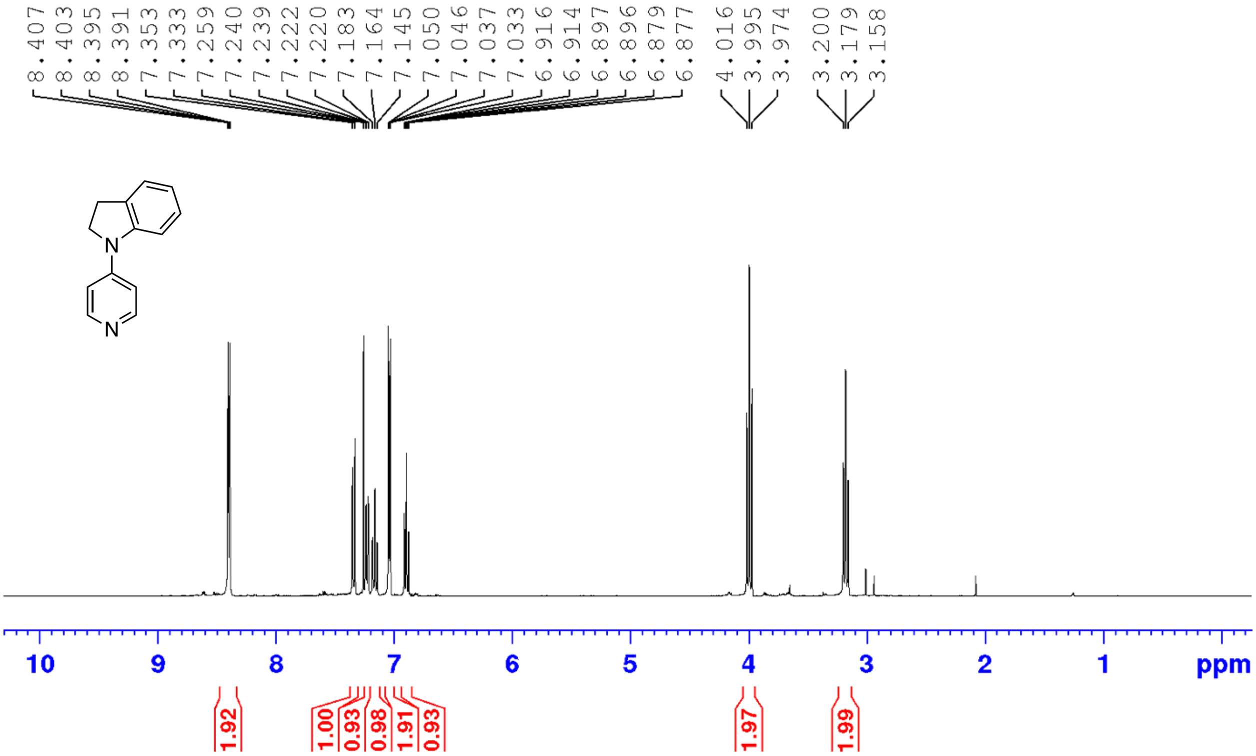

[1]**H NMR** spectrum of **37** (400 MHz, $CDCl_3$)

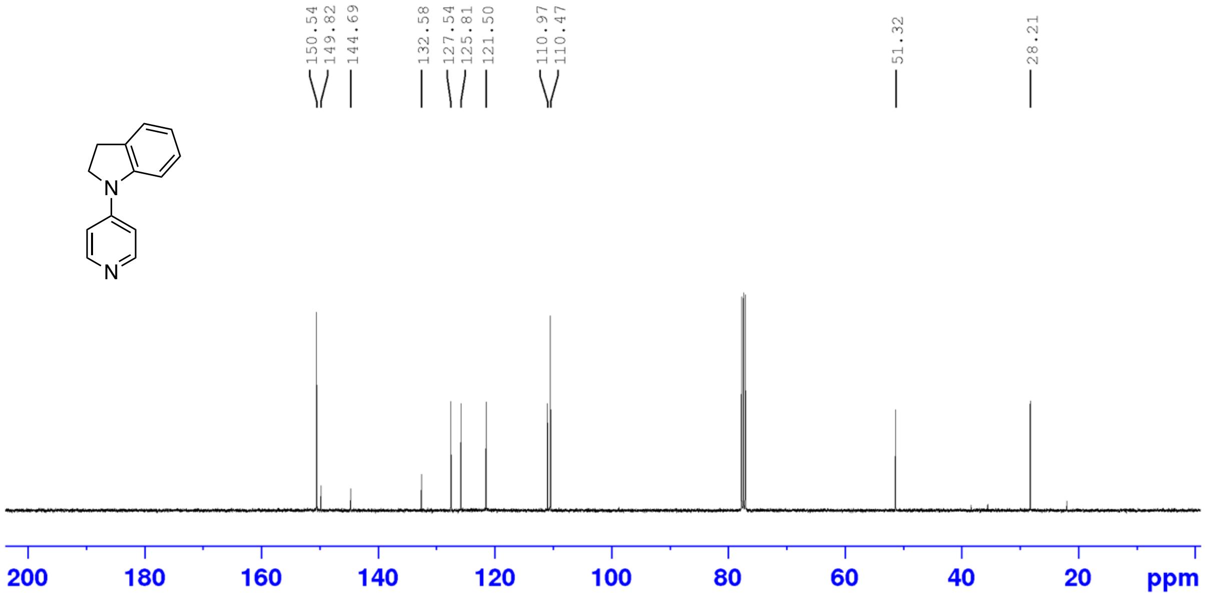

[13]**C NMR** spectrum of **37** (101 MHz, $CDCl_3$)

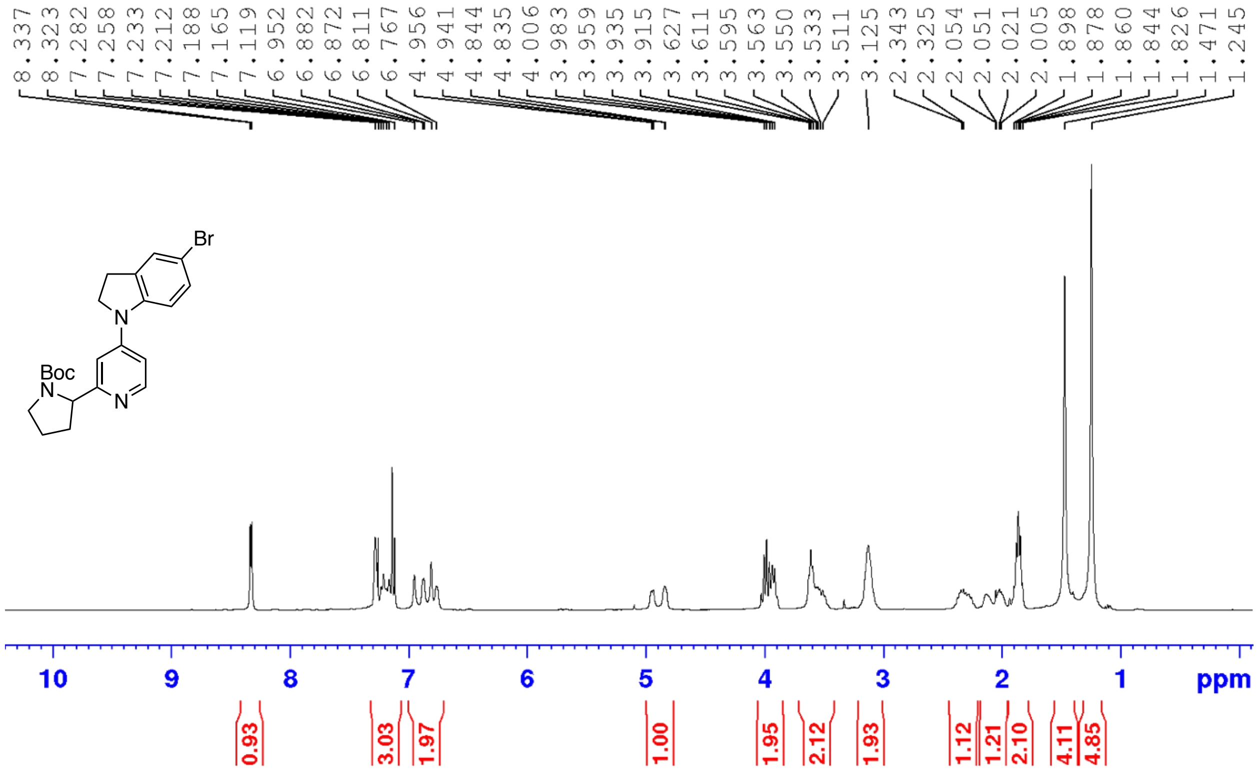

^{1}H NMR spectrum of **38** ($CDCl_3$, 400 MHz)

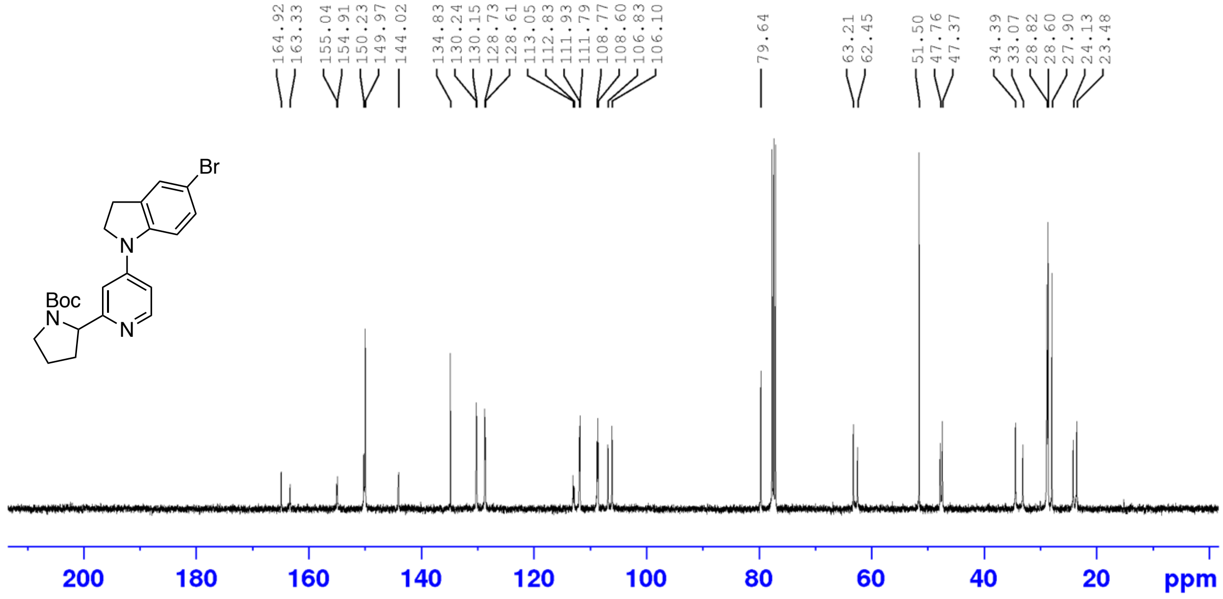

^{13}C NMR spectrum of **38** ($CDCl_3$, 101 MHz)

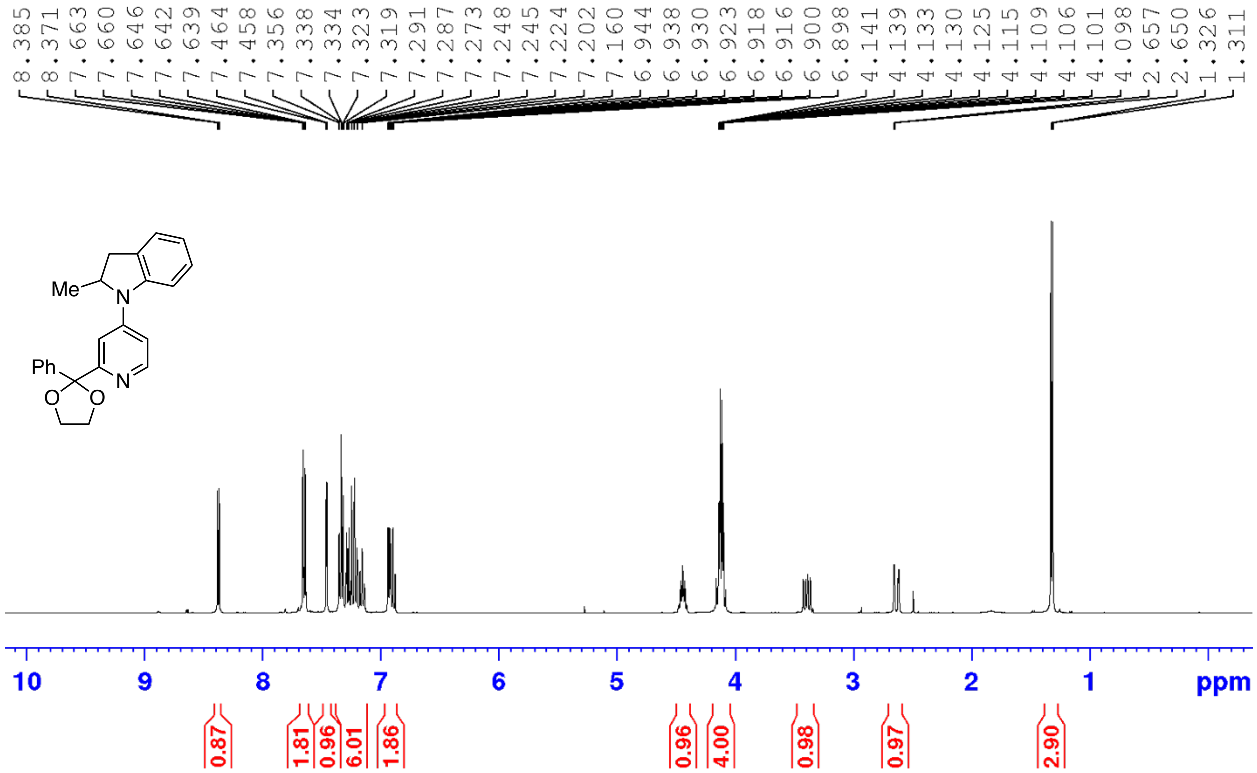

^{1}H NMR spectrum of **39** ($CDCl_3$, 400 MHz)

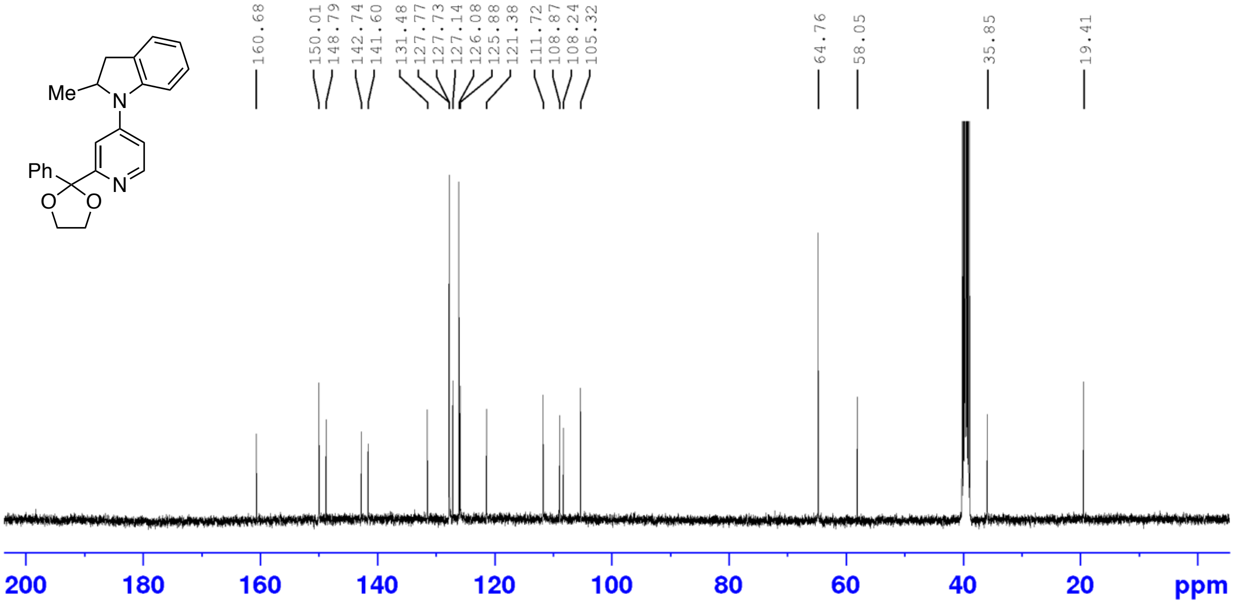

^{13}C NMR spectrum of **39** (d_6-DMSO, 101 MHz)

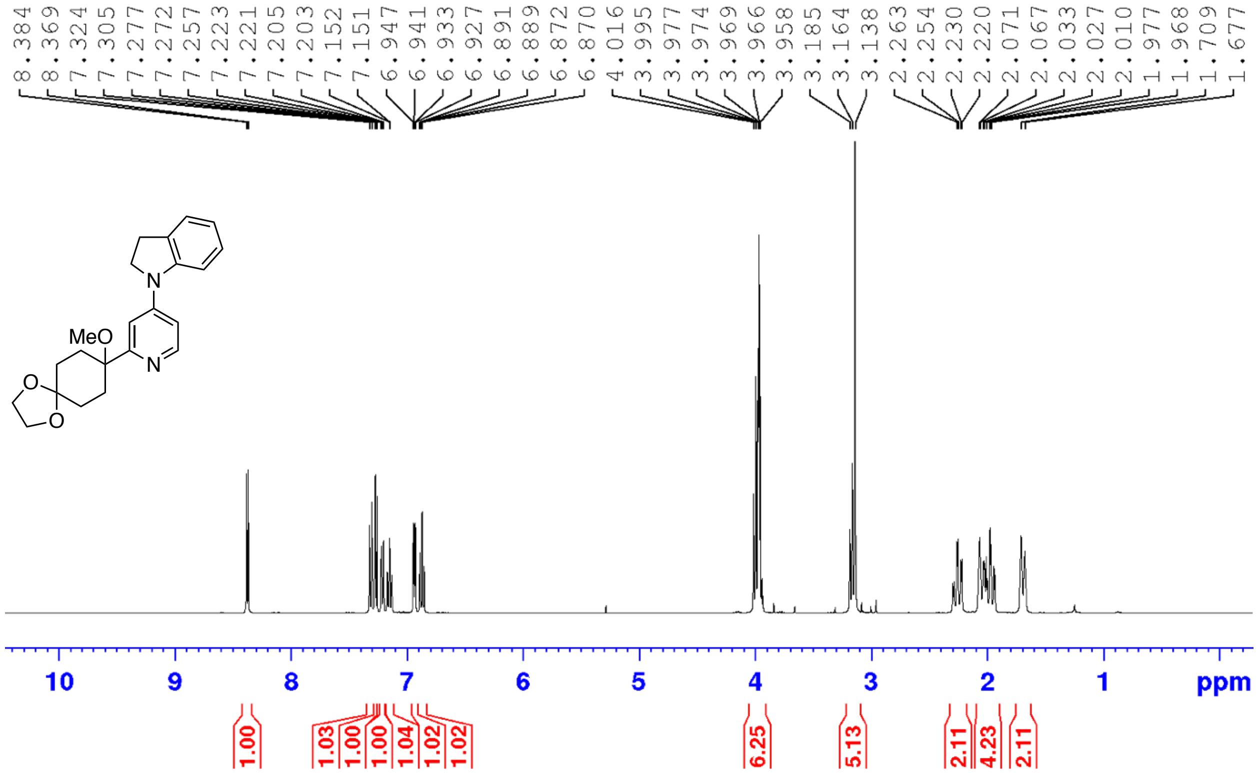

[1]**H NMR** spectrum of **40** ($CDCl_3$, 400 MHz)

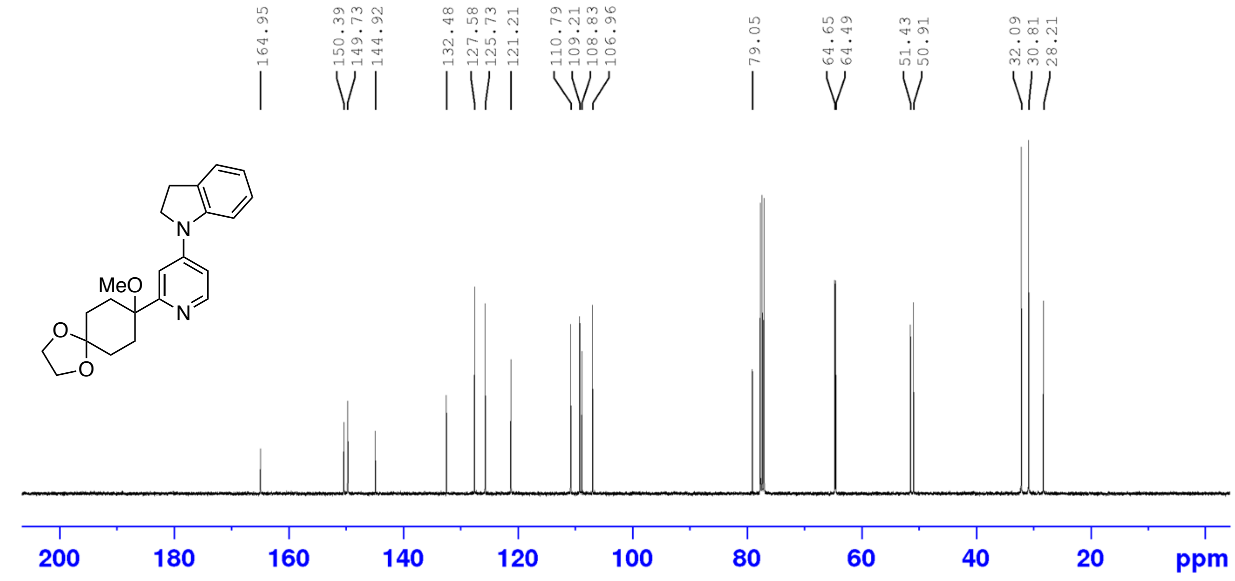

[13]**C NMR** spectrum of **40** ($CDCl_3$, 101 MHz)

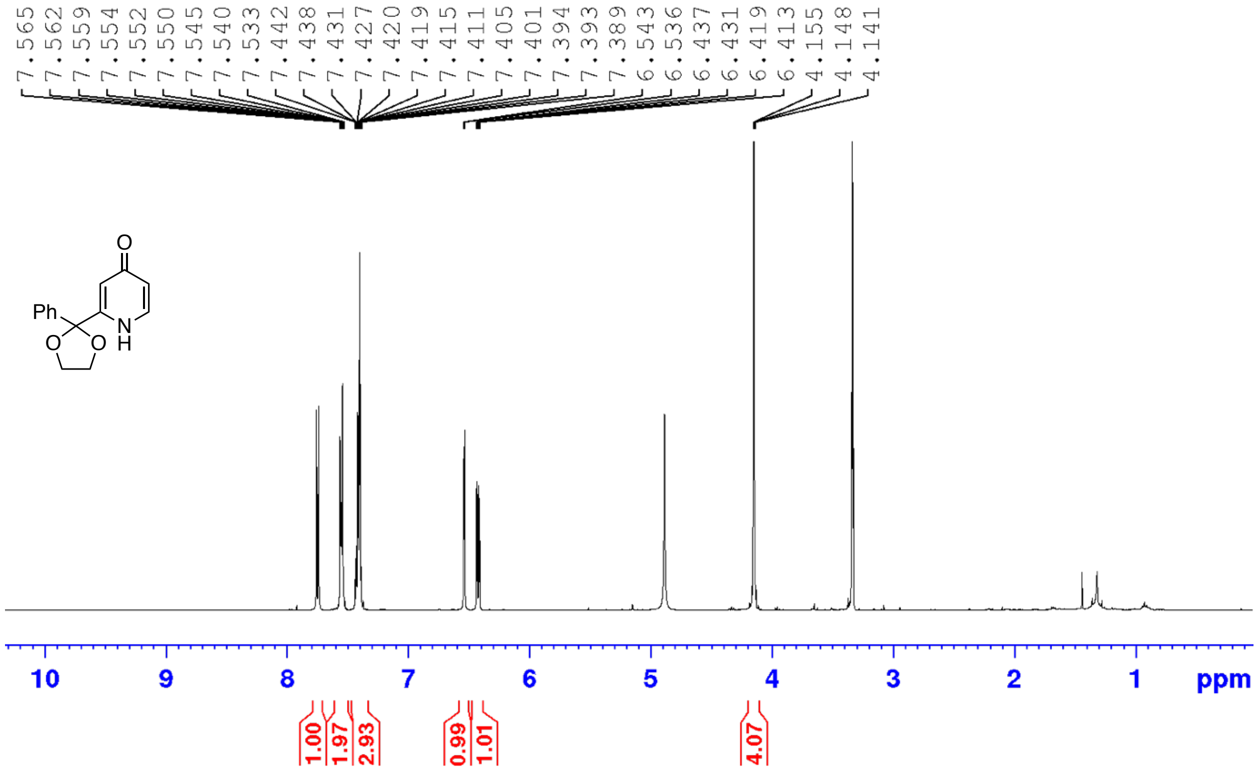

^{1}**H NMR** spectrum of **43** (CD_3OD, 400 MHz)

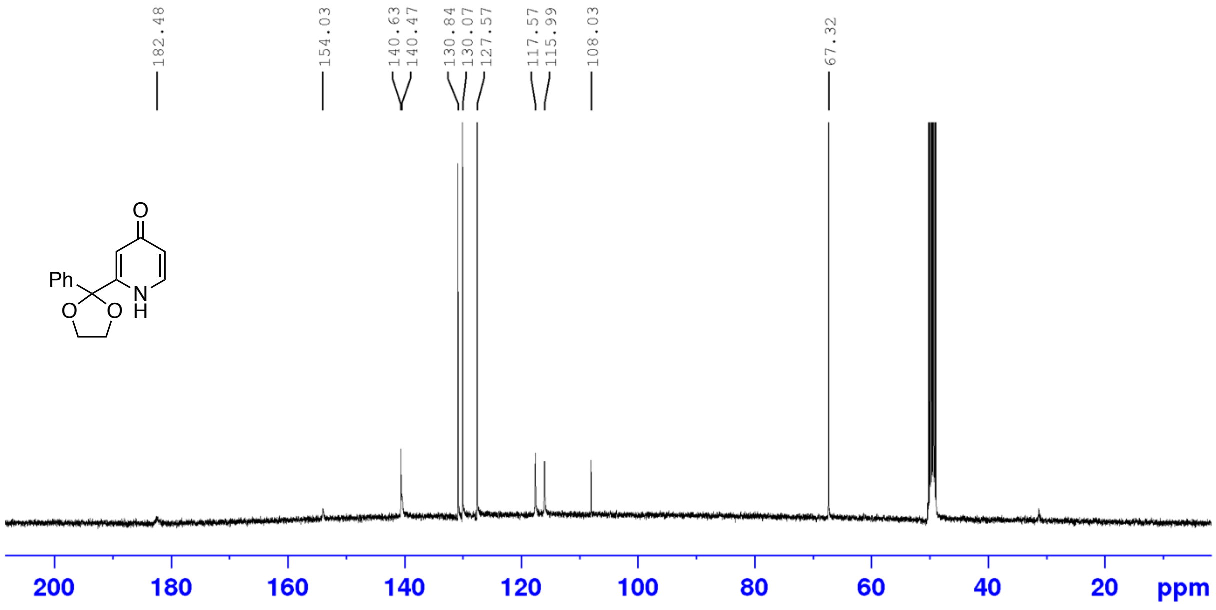

^{13}**C NMR** spectrum of **43** (CD_3OD, 101 MHz)

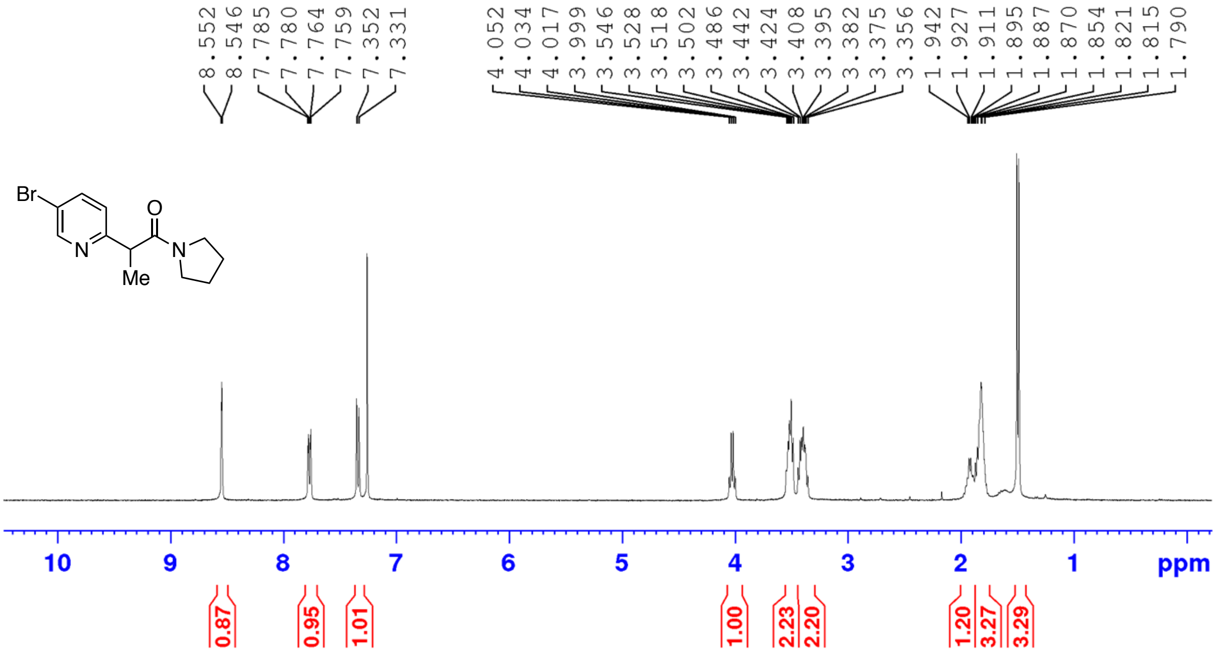

^{1}H NMR spectrum of 2-(5-bromopyridin-2-yl)-1-(pyrrolidin-1-yl)propan-1-one
($CDCl_3$, 400 MHz)

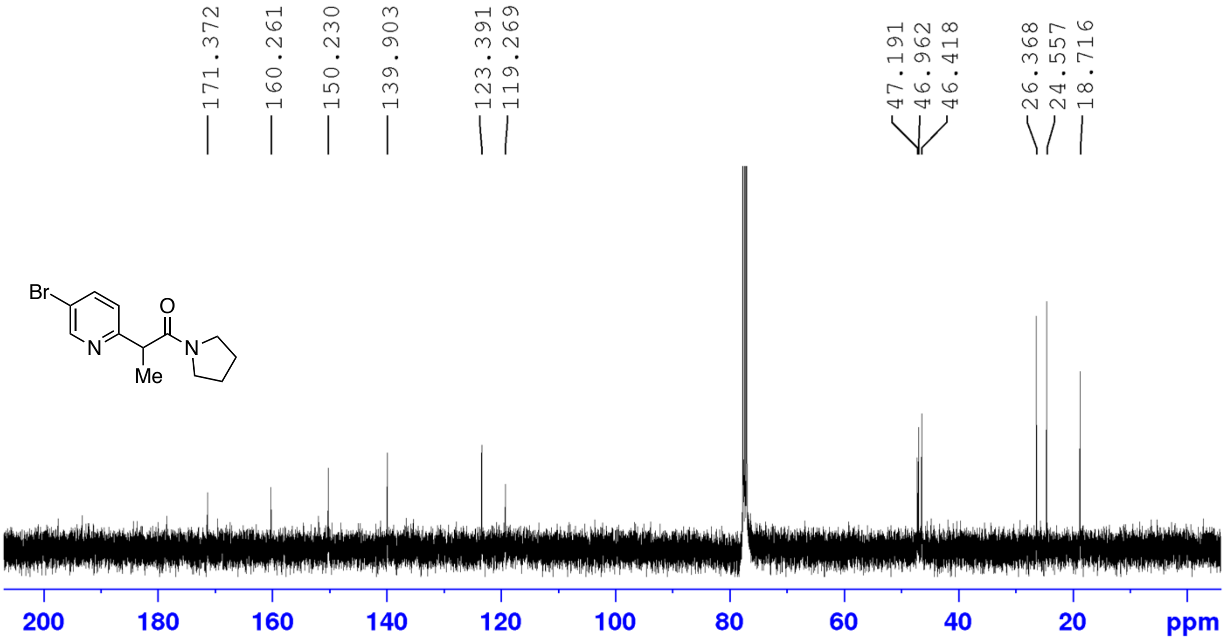

^{13}C NMR spectrum of 2-(5-bromopyridin-2-yl)-1-(pyrrolidin-1-yl)propan-1-one
($CDCl_3$, 101 MHz)

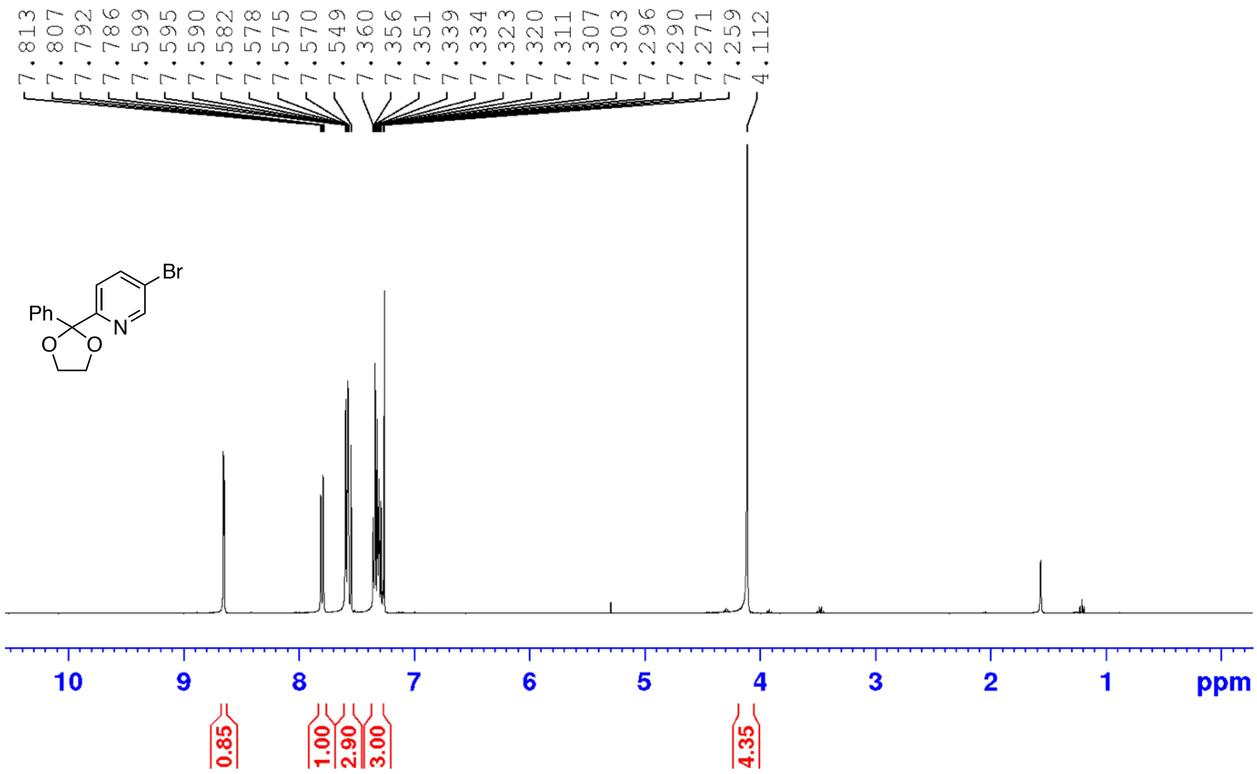

^{1}H NMR spectrum of 5-bromo-2-(2-phenyl-1,3-dioxolan-2-yl)pyridine ($CDCl_3$, 400 MHz)

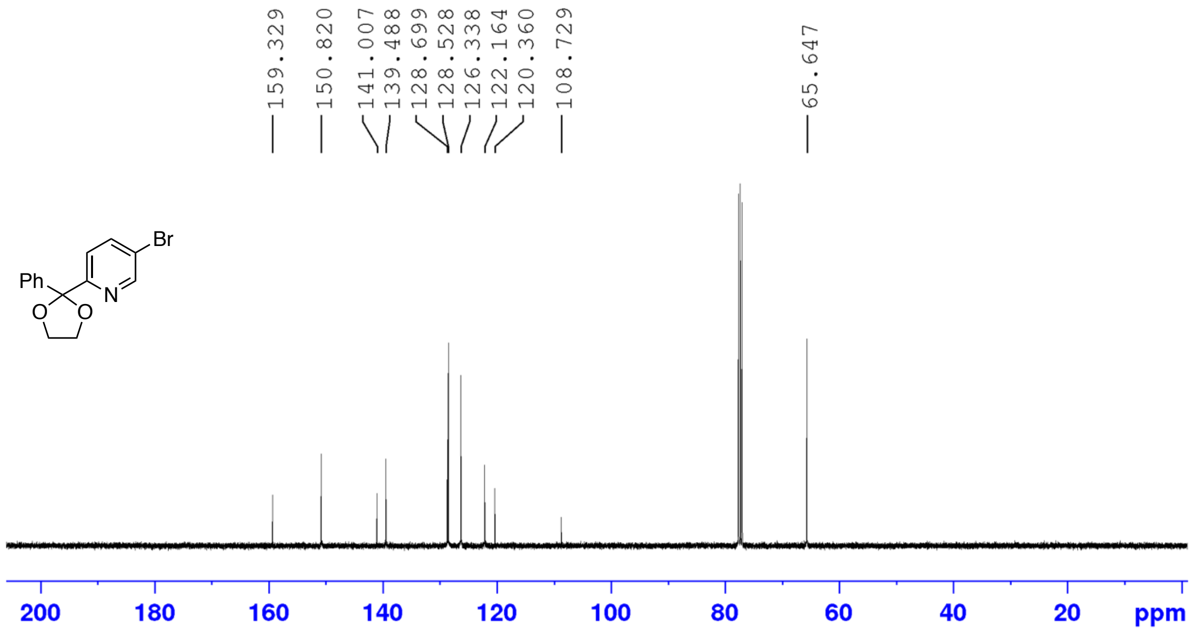

^{13}C NMR spectrum of 5-bromo-2-(2-phenyl-1,3-dioxolan-2-yl)pyridine ($CDCl_3$, 101 MHz)

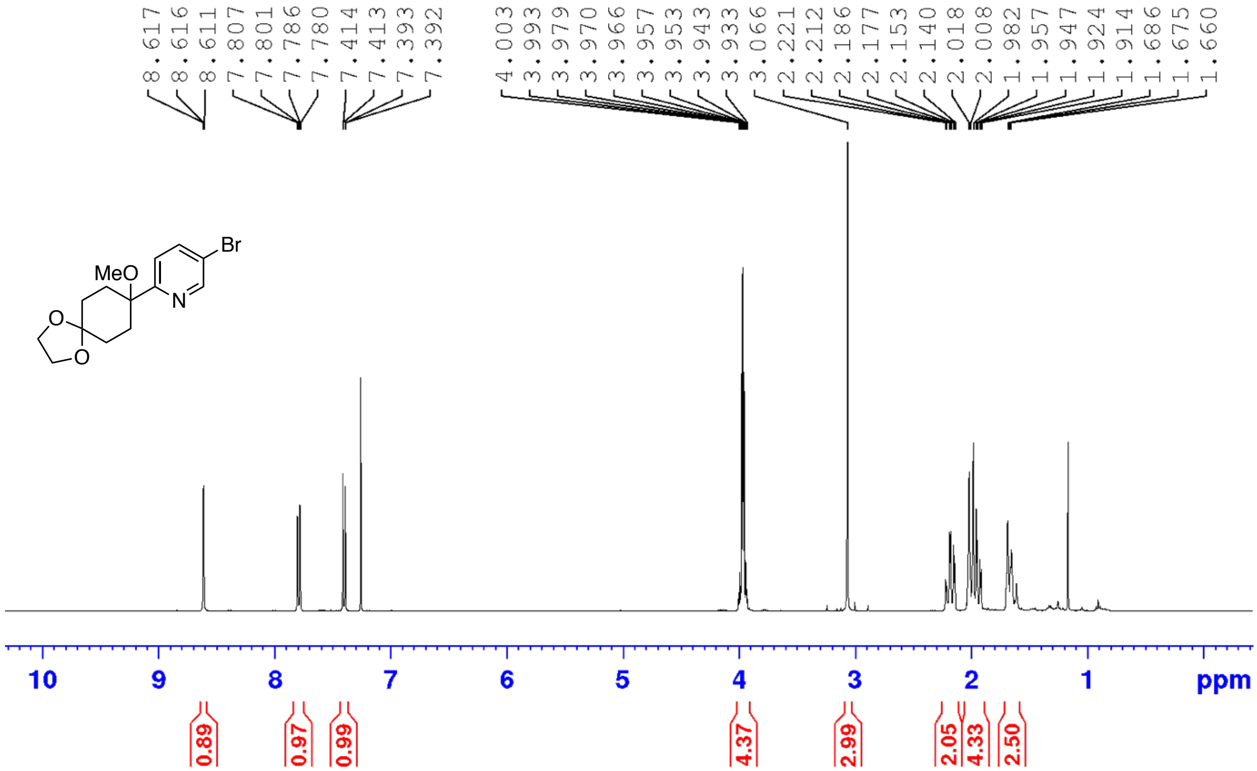

^{1}H NMR spectrum of 5-bromo-2-(8-methoxy-1,4-dioxaspiro[4.5]decan-8-yl)pyridine ($CDCl_3$, 400 MHz)

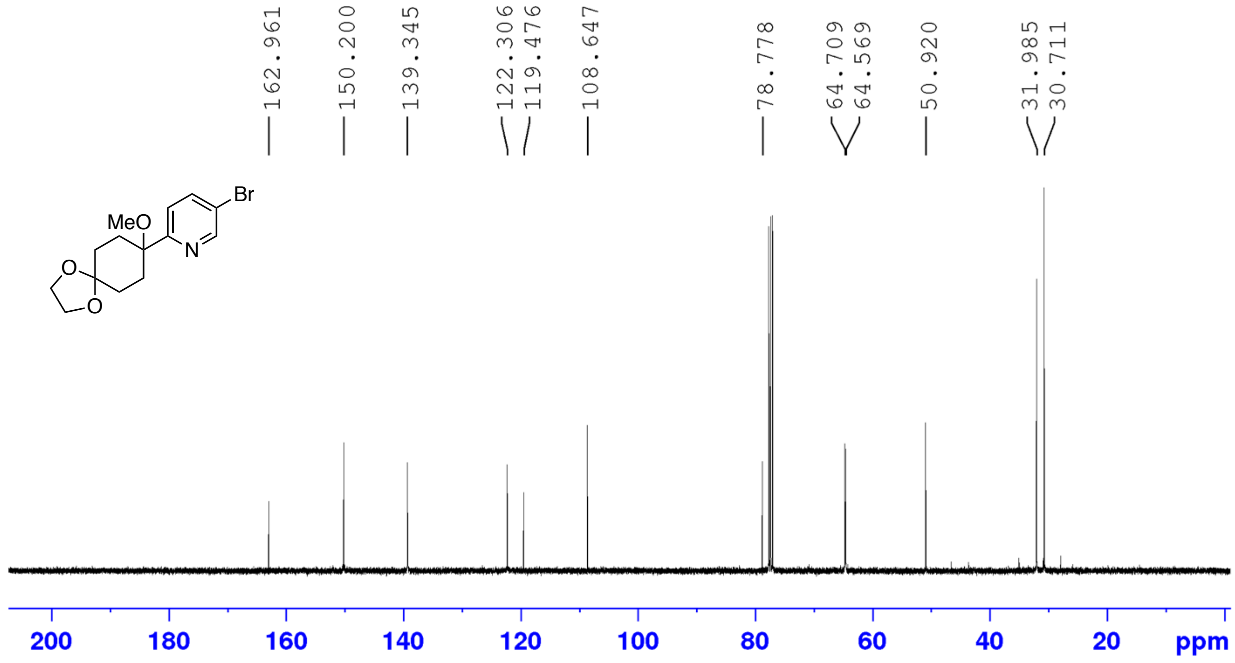

^{13}C NMR spectrum of 5-bromo-2-(8-methoxy-1,4-dioxaspiro[4.5]decan-8-yl)pyridine ($CDCl_3$, 101 MHz)

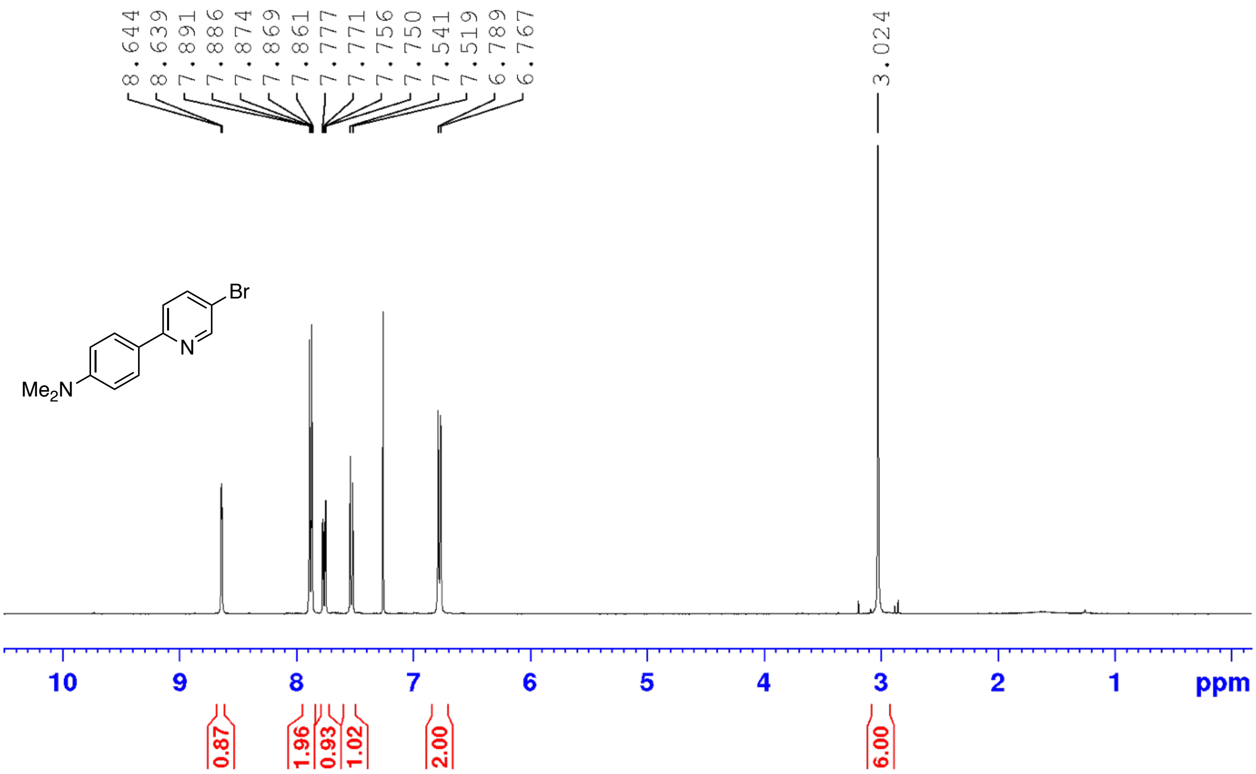

^{1}H NMR spectrum of 4-(5-bromopyridin-2-yl)-*N*,*N*-dimethylaniline ($CDCl_3$, 400 MHz)

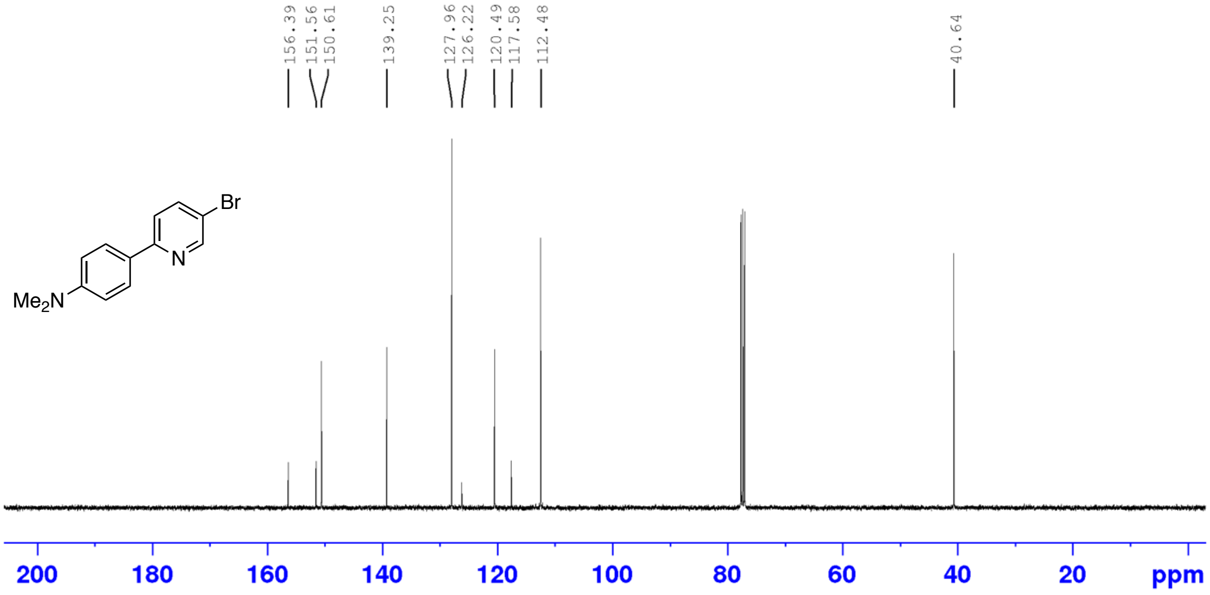

^{13}C NMR spectrum of 4-(5-bromopyridin-2-yl)-*N*,*N*-dimethylaniline ($CDCl_3$, 101 MHz)

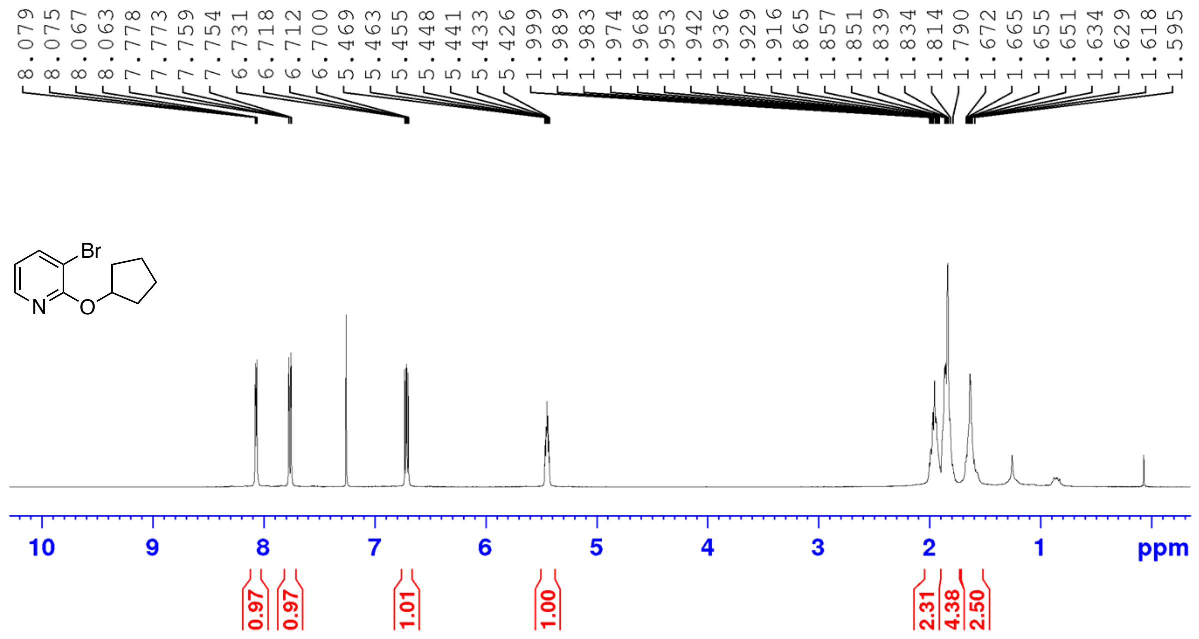

^{1}H NMR spectrum of 3-bromo-2-(cyclopentyloxy)pyridine ($CDCl_3$, 400 MHz)

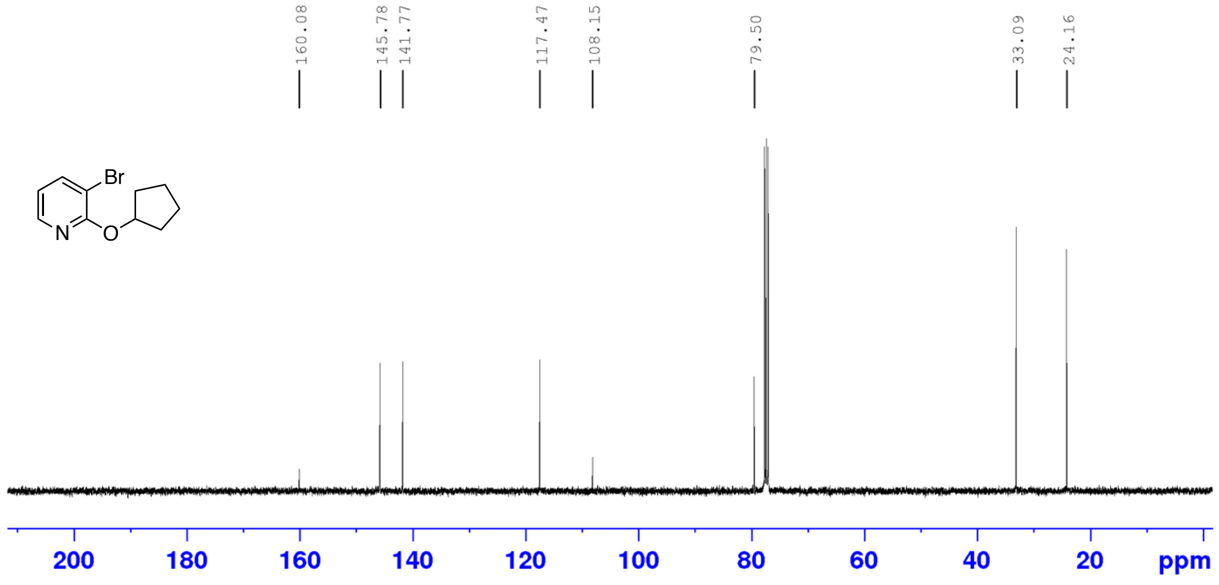

^{13}C NMR spectrum of 3-bromo-2-(cyclopentyloxy)pyridine ($CDCl_3$, 101 MHz)

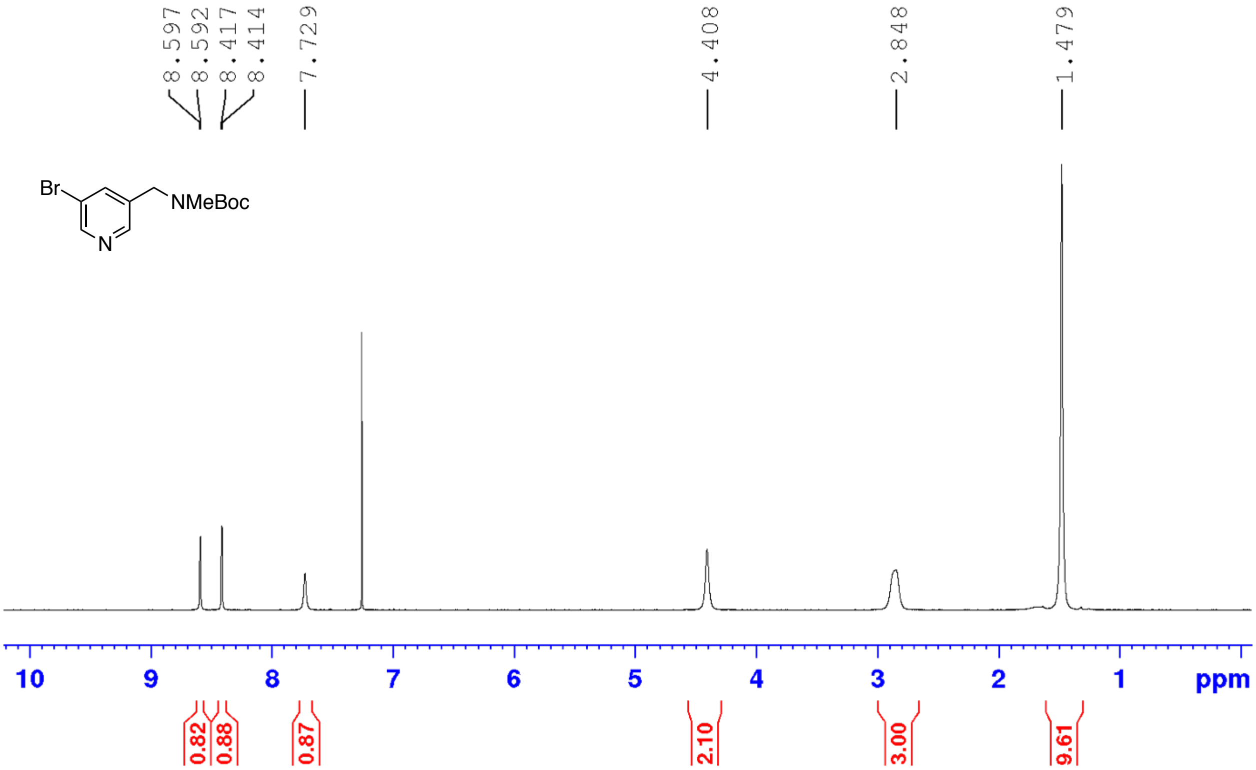

^{1}H NMR spectrum of *tert*-butyl ((5-bromopyridin-3-yl)methyl)(methyl)carbamate ($CDCl_3$, 400 MHz)

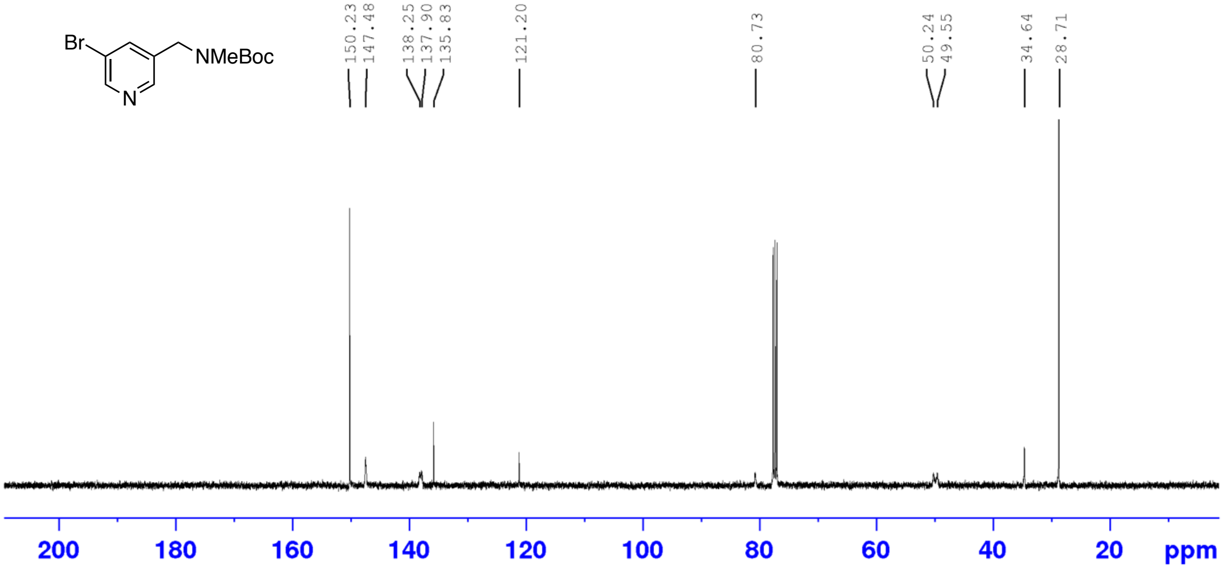

^{13}C NMR spectrum of *tert*-butyl ((5-bromopyridin-3-yl)methyl)(methyl)carbamate ($CDCl_3$, 101 MHz)

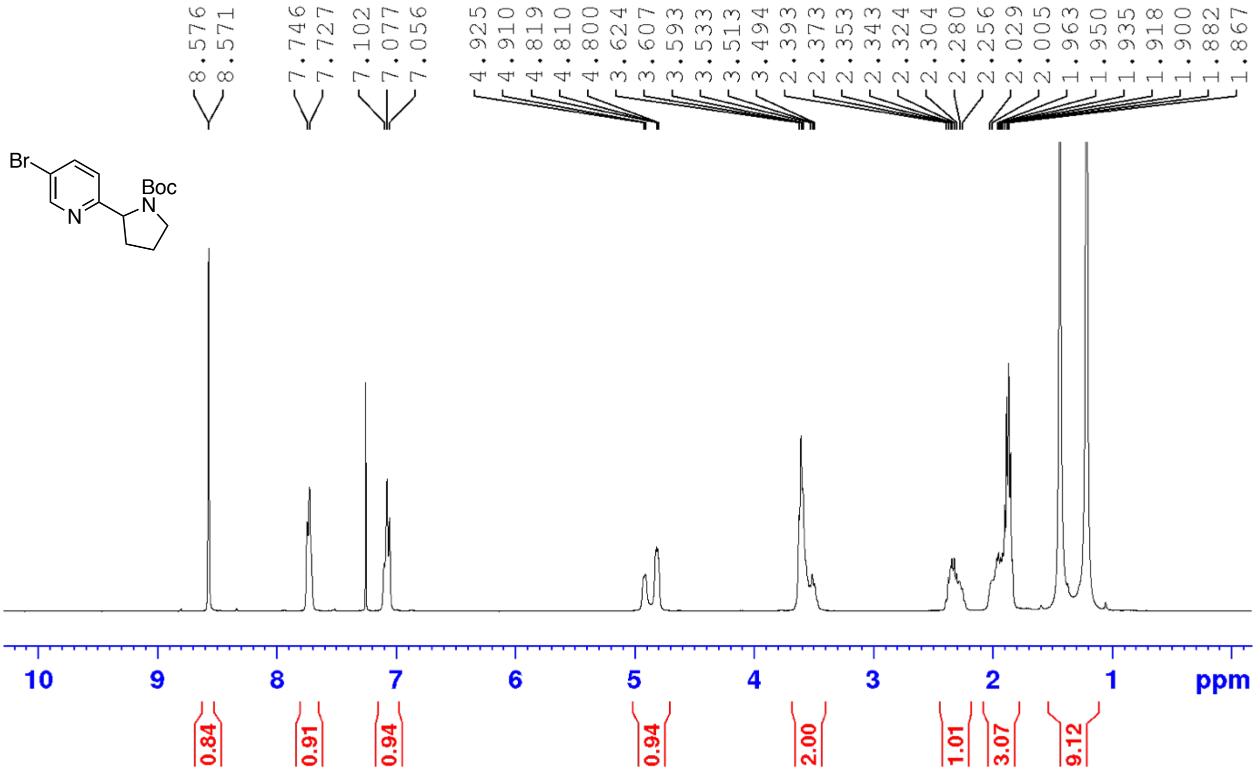

^{1}H NMR spectrum of *tert*-butyl 2-(5-bromopyridin-2-yl)pyrrolidine-1-carboxylate ($CDCl_3$, 400 MHz)

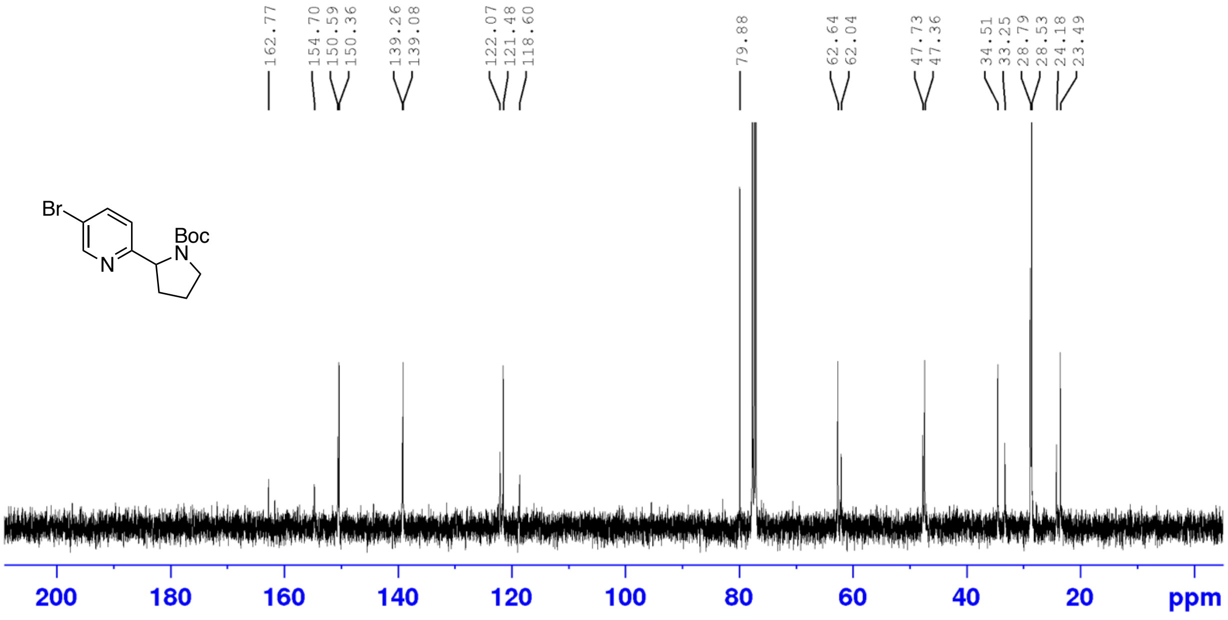

^{13}C NMR spectrum of *tert*-butyl 2-(5-bromopyridin-2-yl)pyrrolidine-1-carboxylate ($CDCl_3$, 101 MHz)

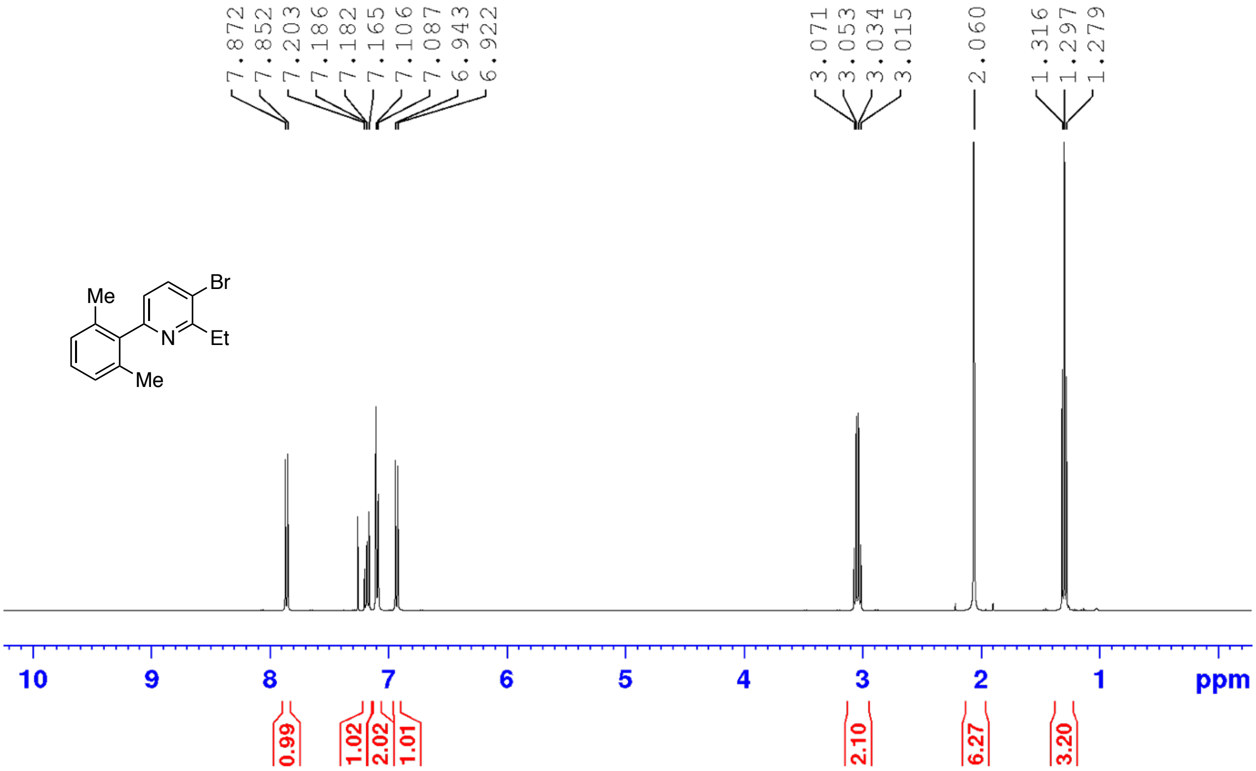

^{1}H NMR spectrum of 3-bromo-6-(2,6-dimethylphenyl)-2-ethylpyridine ($CDCl_3$, 400 MHz)

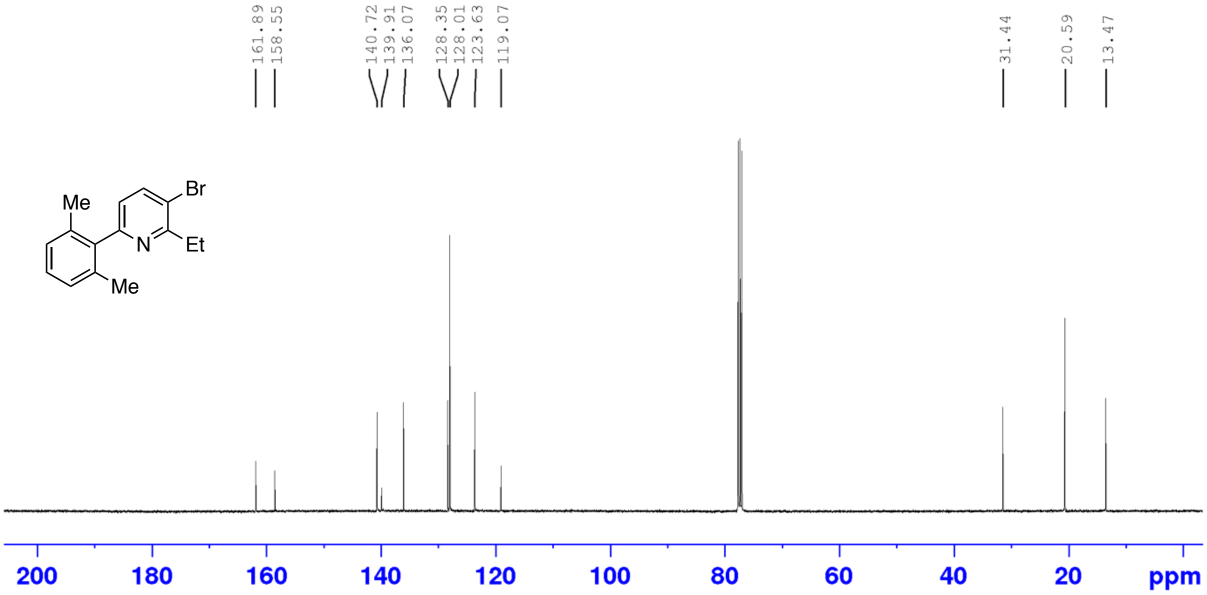

^{13}C NMR spectrum of 3-bromo-6-(2,6-dimethylphenyl)-2-ethylpyridine ($CDCl_3$, 101 MHz)
